# Patient preferences for breast cancer screening: a systematic review update to inform recommendations by the Canadian Task Force on Preventive Health Care

**DOI:** 10.1186/s13643-024-02539-8

**Published:** 2024-05-28

**Authors:** Jennifer Pillay, Samantha Guitard, Sholeh Rahman, Sabrina Saba, Ashiqur Rahman, Liza Bialy, Nicole Gehring, Maria Tan, Alex Melton, Lisa Hartling

**Affiliations:** Alberta Research Centre for Health Evidence, Faculty of Medicine and Dentistry, University of Alberta, 11405 87 Avenue NW, Edmonton, Alberta T6G 1C9 Canada

**Keywords:** Breast cancer, Screening, Patient preferences, Decision-making, Health utilities, Attitudes, Intentions, Systematic review

## Abstract

**Background:**

Different guideline panels, and individuals, may make different decisions based in part on their preferences. Preferences for or against an intervention are viewed as a consequence of the relative importance people place on the expected or experienced health outcomes it incurs. These findings can then be considered as patient input when balancing effect estimates on benefits and harms reported by empirical evidence on the clinical effectiveness of screening programs. This systematic review update examined the relative importance placed by patients on the potential benefits and harms of mammography-based breast cancer screening to inform an update to the 2018 Canadian Task Force on Preventive Health Care's guideline on screening.

**Methods:**

We screened all articles from our previous review (search December 2017) and updated our searches to June 19, 2023 in MEDLINE, PsycINFO, and CINAHL. We also screened grey literature, submissions by stakeholders, and reference lists. The target population was cisgender women and other adults assigned female at birth (including transgender men and nonbinary persons) aged ≥ 35 years and at average or moderately increased risk for breast cancer. Studies of patients with breast cancer were eligible for health-state utility data for relevant outcomes. We sought three types of data, directly through (i) disutilities of screening and curative treatment health states (measuring the impact of the outcome on one’s health-related quality of life; utilities measured on a scale of 0 [death] to 1 [perfect health]), and (ii) other preference-based data, such as outcome trade-offs, and indirectly through (iii) the relative importance of benefits versus harms inferred from attitudes, intentions, and behaviors towards screening among patients provided with estimates of the magnitudes of benefit(s) and harms(s). For screening, we used machine learning as one of the reviewers after at least 50% of studies had been reviewed in duplicate by humans; full-text selection used independent review by two humans. Data extraction and risk of bias assessments used a single reviewer with verification. Our main analysis for utilities used data from utility-based health-related quality of life tools (e.g., EQ-5D) in patients; a disutility value of about 0.04 can be considered a minimally important value for the Canadian public. When suitable, we pooled utilities and explored heterogeneity. Disutilities were calculated for screening health states and between different treatment states. Non-utility data were grouped into categories, based on outcomes compared (e.g. for trade-off data), participant age, and our judgements of the net benefit of screening portrayed by the studies. Thereafter, we compared and contrasted findings while considering sample sizes, risk of bias, subgroup findings and data on knowledge scores, and created summary statements for each data set. Certainty assessments followed GRADE guidance for patient preferences and used consensus among at least two reviewers.

**Findings:**

Eighty-two studies (38 on utilities) were included. The estimated disutilities were 0.07 for a positive screening result (moderate certainty), 0.03–0.04 for a false positive (FP; “additional testing” resolved as negative for cancer) (low certainty), and 0.08 for untreated screen-detected cancer (moderate certainty) or (low certainty) an interval cancer. At ≤12 months, disutilities of mastectomy (vs. breast-conserving therapy), chemotherapy (vs. none) (low certainty), and radiation therapy (vs. none) (moderate certainty) were 0.02–0.03, 0.02–0.04, and little-to-none, respectively, though in each case findings were somewhat limited in their applicability. Over the longer term, there was moderate certainty for little-to-no disutility from mastectomy versus breast-conserving surgery/lumpectomy with radiation and from radiation. There was moderate certainty that a majority (>50%) and possibly a large majority (>75%) of women probably accept up to six cases of overdiagnosis to prevent one breast-cancer death; there was some uncertainty because of an indication that overdiagnosis was not fully understood by participants in some cases. Low certainty evidence suggested that a large majority may accept that screening may reduce breast-cancer but not all-cause mortality, at least when presented with relatively high rates of breast-cancer mortality reductions (*n* = 2; 2 and 5 fewer per 1000 screened), and at least a majority accept that to prevent one breast-cancer death at least a few hundred patients will receive a FP result and 10–15 will have a FP resolved through biopsy. An upper limit for an acceptable number of FPs was not evaluated. When using data from studies assessing attitudes, intentions, and screening behaviors, across all age groups but most evident for women in their 40s, preferences reduced as the net benefit presented by study authors decreased in magnitude. In a relatively low net-benefit scenario, a majority of patients in their 40s may not weigh the benefits as greater than the harms from screening whereas for women in their 50s a large majority may prefer screening (low certainty evidence for both ages). There was moderate certainty that a large majority of women 50 years of age and 50 to 69 years of age, who have usually experienced screening, weigh the benefits as greater than the harms from screening in a high net-benefit scenario. A large majority of patients aged 70–71 years who have recently screened probably think the benefits outweigh the harms of continuing to screen. A majority of women in their mid-70s to early 80s may prefer to continue screening.

**Conclusions:**

Evidence across a range of data sources on how informed patients value the potential outcomes from breast-cancer screening will be useful during decision-making for recommendations. The evidence suggests that all of the outcomes examined have importance to women of any age, that there is at least some and possibly substantial (among those in their 40s) variability across and within age groups about the acceptable magnitude of effects across outcomes, and that provision of easily understandable information on the likelihood of the outcomes may be necessary to enable informed decision making. Although studies came from a wide range of countries, there were limited data from Canada and about whether findings applied well across an ethnographically and socioeconomically diverse population.

**Systematic review registration:**

Protocol available at Open Science Framework https://osf.io/xngsu/.

**Supplementary Information:**

The online version contains supplementary material available at 10.1186/s13643-024-02539-8.

## Introduction

Given similar information on the anticipated benefits and harms of interventions, guideline panels, and individuals, may make different decisions based in part by their values and preferences [1]. Recommendations aligned with patient values and preferences may be more easily accepted and implemented [2]; those that may not align well with some peoples’ values can include considerations of needs for individual or shared decision-making.

Strategies to incorporate patient/public preferences and values during guideline development may differ between guideline producers, in terms of how they collect the data (e.g., literature review and/or direct patient input), how values and preferences are defined, and how findings are incorporated within the guideline development process or recommendations [3]. For the purposes of this systematic review, we are defining preferences and values similar to the Grading of Recommendations Assessment, Development, and Evaluation (GRADE) working group, in terms of the relative importance (“weight”) placed on the benefits and harms (“outcome valuation”) of breast cancer screening [2, 4, 5]. Preferences for or against an intervention are viewed as a consequence of the relative importance people place on the expected or experienced health outcomes it incurs. These findings can then be considered as patient input when balancing the effect estimates on benefits and harms reported by empirical evidence on the clinical effectiveness of screening programs.

Outcome valuations can make use of comparisons between different health-state utility values (HSUVs) or data from other utility-based stated and revealed preference studies including contingent valuation studies, such as discrete choice experiments (DCEs), or simple ratings scales or trade-offs. HSUVs reflect preference-based health-related quality of life (HRQoL) and represent the strength of an individual’s preferences for the health outcome or health state under consideration [6]. They are measured on a scale of 0 (death) to 1 (perfect health), thus a more desirable health outcome will have a higher utility value and vice versa. Health utilities can be measured using direct choice-based utility elicitation methods such as standard gamble (SG), time tradeoff (TTO) (determining what people would be willing to risk or give up to avoid living in that health state), or indirect methods using generic multi-attribute utility instruments such as the EuroQoL 5-Dimensions (EQ-5D) with the generation of a health state and its associated utility based on tariffs from previous valuations by members of the general public. Disutilities, or decrease in utilities, can be used to assess HSUV reductions (i.e., negative impact on one’s HRQoL) compared with a person’s health state before experiencing the outcome (their “healthy state”) or with a (presumably) more desirable health state (e.g., receipt of chemotherapy vs. no chemotherapy after surgery for breast cancer). For any given health state, the HSUVs can vary depending on the method of health utility estimation, the population used to derive utility scores (patients, caregivers, health professionals, or the general public), and the context (setting, method or mode of administration, or description of health state). For a commonly used measurement tool, EQ-5D, a utility change/difference of about 0.04 can be considered a minimally important value for the Canadian general public [7].

Indirectly, the relative importance people place on the expected outcomes from an intervention can be inferred based on preferences for or against the intervention, measured through attitudes, intentions, and/or behaviors after being adequately informed about the expected outcomes [2]. Because the information provided usually covers a range of outcomes, this indirect measurement will typically only allow for the valuation of the anticipated desirable (benefits) versus undesirable (harms) outcomes, rather than for any two specific outcomes. Further, it will not often be based on peoples’ experience with the outcomes and may vary depending on the description (e.g., magnitude of possible effects) and understanding of the anticipated outcomes. The methods are considered indirect because intentions and behaviors related to an intervention are often influenced by other factors—for example, subjective norms, perceived behavioral control, accessibility, and emotional factors [8–10]—apart from a person’s outcome valuations. This review does not examine evidence or other sources of data (e.g., social media) about the general sentiment of patients or the public about breast cancer screening when it is unclear whether and what evidence-based information they are using to base their opinions.

### Objective

This systematic review update will help inform an update to the 2018 Canadian Task Force on Preventive Health Care’s (task force) guideline on breast cancer screening [11]. The findings will be considered as one form of patient input when the task force is balancing the effect estimates on benefits and harms based on empirical evidence of the clinical effectiveness of screening programs. Other forms of patient engagement are used during the development of recommendations, key messages, and knowledge dissemination tools (e.g., members of task force working group, public advisory network) [12] and can help inform this topic on preferences and other considerations related to acceptability, resource use, and feasibility that contribute to recommendations. For this review, we answered the following research question: what is the relative importance placed by patients on the potential benefits and harms of mammography-based breast cancer screening?

## Methods

This is a modified update to our previous systematic review completed in 2018 [13]. The full research plan for the evidence reviews for this guideline update and the protocols for the key questions on the benefits and harms of screening and for this review on patient preferences can be found at Open Science Framework https://osf.io/xngsu/. The scope of the review was informed by a working group consisting of task force members, clinical experts external to the task force, and patient partners (see Acknowledgements). A draft research plan was also reviewed by stakeholders throughout Canada and peer reviewers, with all comments considered by the task force and review team and modifications made as suitable. The research plan and protocol were then finalized during the pilot stages of the screening and data extraction. Any deviations to the protocol are described herein. A lay summary of the methods and findings of this review can be found on the task force's website (https://canadiantaskforce.ca/).

For this update, the eligibility criteria were broadened to include HSUVs. HSUVs and other data from preference-based (e.g., discrete choice experiments, trade-offs) or non-preference-based (e.g., relative importance of benefits versus harms inferred by intentions to screen after receiving information) studies focused on the task force’s ratings of outcomes considered important or critical (ratings of 4–6 or 7–9 on a 9-point scale, respectively) for their decision making: anticipated benefits included reductions/improvements in breast-cancer and all-cause mortality, curative treatment-related morbidity (measured indirectly through receipt of radiotherapy, chemotherapy, mastectomy [vs. breast-conserving surgery (BCS)/partial mastectomy], or axillary lymph node dissection [ALND; vs. sentinel lymph node biopsy, SLNB]), advanced-stage disease (ideally via reductions in stage III+ cancer), HRQoL, and life years gained; anticipated harms included overdiagnosis (a measure of harms from the label and treatments received for cancer that would have never caused harm, never progressed, progressed too slowly to cause symptoms or harm during a person’s remaining lifetime), false positive/alarms (resolved by any means; FPs), FPs resolved by biopsy, and interval cancers (cancers not recognized during screening [false-negative results] or clinically detected between screening rounds). Although for this review, we use the term “false positive” which is common in the scientific literature, the task force will refer to the related screening outcomes as requiring “additional imaging with or without biopsy (no cancer)” or “additional imaging and biopsy (no cancer)” to avoid any implication that the test is positive for cancer or even that the results always indicate an abnormality.

This systematic review follows methods approved by the task force [12], with the following deviations to allow expedited processes: (i) use of machine learning via DistillerAI (DistillerSR Inc., Ottawa, Canada) as one of the reviewers for reviewing titles and abstracts after at least 50% of studies (predicted > 95% of includes) had been reviewed in duplicate by humans and with a quality check to ensure accuracy, and (ii) use of verification by a senior reviewer rather than dual independent data extraction and risk of bias and certainty assessments. We report the systematic review according to the Preferred Reporting Items for Systematic Reviews and Meta-Analyses 2020 statement [14].

### Eligibility

Studies were selected according to the inclusion and exclusion criteria outlined in Table 1. The target population was cisgender women and other adults assigned female at birth (including transgender men and nonbinary persons) ≥ 35 years of age (in ≥ 80% of the sample) with average or moderately increased risk for breast cancer. As the majority of evidence reviewed defined their population as “women,” both the terms “women” and “patients” will be used throughout this review. We included women younger than 40 years of age (target age limit for the task force’s guideline) to capture individuals considering screening in the near future. For studies of HSUVs related to cancer diagnosis or treatment, participants preferentially had experience with breast cancer (currently or in the past), but if no data were found from this population for analysis, we used public samples presented with hypothetical scenarios about cancer diagnosis and/or treatment. We excluded studies reporting HSUVs based on samples of healthcare providers. If not reporting whether a new cancer was detected by screening, we included data from untreated new diagnoses detected by any means as long as < 10% of the sample had stage IV disease; this threshold for stage IV was also used for HSUVs of curative treatments. For other data, such as HSUVs related to screening health states (e.g., before screening [to capture the utility of a “healthy screen-eligible population” for calculating disutilities of other states], a positive test before diagnostic work-up, FPs, invasive procedures during diagnostic work-up) and from non-HSUV studies, we excluded studies with ≥ 20% participants at high-risk for breast cancer (Table 1).

**Table 1:** Eligibility criteria

	**Inclusion**	**Exclusion**
**Population**	Cisgender women and other adults assigned female at birth (including transgender men and nonbinary persons) aged ≥ 35 years of age* with average or moderately increased risk for breast cancer For studies of HSUVs related to a new cancer diagnosis or the impact of cancer treatments (exposures 5–7), participants will have experienced cancer or will be presented with hypothetical scenarios about cancer diagnosis and/or treatment. Specific populations: • All outcomes: age (35–39, 40–44, 45–49, 50–69, 70–79, 80+; also < 50 vs 50–60 and 60–70 vs. >70 [allowing for 5-year deviations if necessary]), ethnicity/race, risk for breast cancer (e.g., family history) • For non-HSUV (health-state utility values) studies: previous experience with screening, a false positive, or previous breast surgery • For HSUV studies (exposures 5–6; see Exposures): stage 0 vs. I–III (allowing for 0/I vs. II to III if necessary) *For age and other variables of interest (e.g., chemotherapy use), we will allow for < 20% of the sample to be ineligible)	*Except for studies measuring HSUVs on cancer diagnosis and treatment (exposures 5–7):* Adults with high risk (> 20% of the study population) of breast cancer: personal history of breast cancer or high-risk breast lesions; extensive family history of breast or ovarian cancer or significant genetic markers or syndromes (e.g., BRCA1/BRCA2, Li-Fraumeni syndrome); previously received high-dose radiation treatment to the chest (e.g., Hodgkin’s) *For HSUV studies on exposures 5*–*7*: the above applies except we will include studies of breast cancer diagnosis or treatment, unless focus is on recurrent cancer, specific treatment-related adverse effects, or studies where the breast is not the primary cancer site; *for exposures 5–6*: excluded studies with >10% metastatic/non-curative disease and not reporting data by stage or curative vs. metastatic
**Exposure(s)**	For non-HSUV studies (focus on screening): (i) exposure to information on the expected magnitude of ≥ 1 benefit and ≥ 1 harm from screening (as per task force ratings of important or critical outcomes); (ii) experience of FPs and provided with information on benefits to make decisions for future screening; or (iii) no exposure or information but values (e.g., trade-offs) for ≥ 1 benefit and ≥ 1 harm are elicited by studies For HSUV studies: 1. Prior to screening or, if necessary, negative screening result or no cancer sample within a study measuring another exposure of interest 2. Positive screening mammography (before results of diagnostic testing known) 3. False-positive result, if possible by +/− invasive testing 4. Invasive diagnostic testing (e.g., any form of biopsy or localization technique; cancer status not known) 5. True-positive result (all treatment naïve) (may include new diagnosis if not clearly screen-detected) (*post hoc added interval cancer diagnosis*) 6. Curative treatment—variables of interest include (between and within-study comparisons eligible): i) Complete mastectomy vs. partial mastectomy/lumpectomy ii) Receipt of chemotherapy (yes/no) (a) Subgroups by: anthracycline vs. no anthracycline iii) Receipt of radiotherapy (yes/no) iv) Axillary lymph node dissection vs. sentinel lymph node biopsy 7. Advanced stage: i.e., during treatment for stage 0–II vs. II+, 0–II vs III/IV, metastasized vs not; using within-study comparisons	HSUVs from second or higher line of treatment settings or from experimental/not approved treatments
**Comparators**	None If studies compare two different versions of information/decision aids or two different types of treatment, each eligible arm will be considered separately.	
**Outcomes**	Preference-based outcomes: • HSUVs, *using hierarchy*: (i) generic multi-attribute utility instruments (e.g., EuroQoL-5D, Health Utilities Index, or Short form-6D) by patients (or their proxies) (based on the current status for exposure 6 but may be through recall for other exposures]); (ii) if *N* < 100 or all studies are high risk of bias for a given health state from (i), use generic multi-attribute utility instruments in population sample (e.g., previous patients or eligible for screening) and TTO, SG (not VAS). • Estimated disutilities for each HSUV (vs. healthy eligible population for screening) using data from exposure 1 or from Canadian norms value set for females aged 40–70. • Non-HSUVs: ◦ Preference weights from contingent valuation studies for benefit and harm outcomes ◦ Relative ranking/rating or probability trade-offs between benefit and harm outcomes (e.g., ratings based on the degree of importance to screening decision-making) ◦ Others will be considered Indirect, non-preference-based relative importance of outcomes (i.e., benefits vs harms) based (inferred from) on: • Willingness to be screened, acceptability or attitudes about screening, uptake of screening, intent to return for another screen, and others will be considered	HSUVs from disease-specific HRQOL tools that are mapped to utility measurements or from disease-specific utility measures HSUVs elicited from health care providers
**Timing**	Non-HSUV studies and HSUVs in exposures 1–5: Published on or after 2000 For HSUVs in exposures 6 and 7: published in 2014 or later to account for major changes to treatment over time; two time points for analysis: (i) within 1 year of surgery/treatment initiation and (ii) ≥ 2 years after surgery; using the worst utility within each study at each time point	
**Settings and sample**	Countries having a Very High Development Index For HSUV studies, ≥ 30 study participants for each HSUV exposure reported	
**Study designs**	All quantitative studies, examples including: • Direct measurement of utilities, e.g., HSUV measurement, utility-based stated and revealed preference studies including contingent valuation studies including discrete choice experiments, willingness to pay • Surveys or other studies using questions to rate or rank outcomes (e.g., visual analogue scales or rating scales) • Studies examining decision aids or preferences for screening based on information on the magnitude of benefits and harms Studies may collect quantitative data from all participants using focus groups or other interview techniques (i.e., themes and/or other qualitative data are not eligible) Studies may be embedded within randomized controlled trials or other controlled study designs	Commentaries, opinion, editorials, case reports, and reviews Conference abstracts (contact will be made for full texts from authors if study looks eligible, e.g., does not just mention “quality of life” without indication of utilities or a specific eligible tool)
**Language**	English and French	

*HRQOL* Health-related quality of life, *HSUVs* Health-state utility values, *SG* Standard gamble, *TTO* Time trade-off, *VAS* Visual analogue scale

For non-HSUV studies, patients had to have exposure to information on the expected magnitude of ≥ 1 benefit and ≥ 1 harm from screening (as per task force ratings of important or critical outcomes), or data on benefits if previously exposed to a FP, unless there was elicitation by the authors of the trade-offs between different magnitudes of benefit(s) and harm(s). We included quantitative data only, though this could be collected by qualitative data collection methods such as focus groups.

We collected HSUV data elicited directly by TTO and SG methods, and indirectly using a utility-based generic HRQoL tool (e.g., any version of EQ-5D, Short-form (SF)-6D, 15-Dimension, Health Utilities Index [HUI]). For the treatment comparisons of interest (e.g., chemotherapy vs. none), we included studies that reported on only one of the exposures (e.g., chemotherapy) with plans to use between-study as well as within-study comparisons. For the exposure of advanced-stage disease (e.g., treated stages I–III vs. IV), we only included studies if there was a within-study comparison due to a vast amount of literature on the utilities in advanced disease. Studies needed to be reported in English or French and the country of data collection had to be considered Very Highly Developed as per the United Nations Development Programme’s Human Development Index [15]. Date of publication was limited to 2000 onwards (given the increased scrutiny about harms from screening [16, 17]), with the exception of studies reporting on HSUVs for treatment-related states (surgery, chemotherapy, and radiation) where the date was limited to 2014 onwards to capture treatments subjected to advances over time, especially related to the surgical management of the axilla.

#### Literature search

An information specialist modified our 2017 peer-reviewed search (combining terms for breast cancer screening and decision making/attitudes/intentions) to add terms for studies reporting on HSUVs for breast cancer patients and screening outcomes (e.g., need for additional imaging/false positives). Our search concept for HSUVs has been peer-reviewed for other relevant topics. The searches used both controlled vocabulary, such as the National Library of Medicine’s MeSH (Medical Subject Headings), and keywords in three databases: MEDLINE (1946–) via Ovid, CINAHL via EBSCOhost (1937–present), and PsycINFO via Ovid (1987–present). Database searches were run on June 19, 2023. Methodological filters were not applied to limit retrieval by study design. Searches were restricted by language to include full texts published in English and French, with a publication date of 2017 onwards. Supplementary file 1 contains the final search strategies. To capture eligible studies on HSUVs published between 2000 and 2017 (for utilities related to screening outcomes) or between 2014 and 2017 (for utilities from treatment-related states), we used our database searches to locate and scan systematic reviews on HSUVs in breast cancer screening or patients to assess all of the included studies against our eligibility criteria. Because the last version of this review was conducted when the task force only rated breast cancer and all-cause mortality as critical benefit outcomes, we scanned our previous review’s excluded studies lists to locate studies weighing harms against the additional outcomes rated as critical for this guideline update (e.g., weighing the importance of reduction in advanced stage vs. one or more harm).

On August 10, 2023, we also searched (past 2 years) for completed studies in clinicaltrials.gov and WHO ICTRP for non-HSUV data, and ISPOR Presentations Database, International Health Economics Association (IHEA), Congress International Society for Quality of Life Research (ISOQOL), the EQ-5D Database, and the University of Sheffield’s School of Health & Related Research Health Utilities Database (ScHARRHUD) for studies reporting HSUVs (Supplementary file 1 includes search terms and results). We also reviewed all submissions solicited by the task force from stakeholders and reference lists of included studies.

All results of the database searches were imported into an EndNote® database (Thomson Reuters, New York, NY) for reference citation, and after duplicate removal, into DistillerSR (DistillerSR Inc. Ottawa, Canada) for screening and selection procedures. Results from the grey literature searches and from scanning references of reviews and included studies were uploaded into EndNote and exported into Excel for screening and selection.

#### Study selection

To screen primary studies identified from the database searches, we applied the machine learning program DistillerAI (DistillerSR) which continually reprioritizes records during screening [18]. DistillerAI learns from human reviewers’ inclusion decisions to assign a likelihood score for each unscreened record. Further, a threshold likelihood score for inclusion can be applied, allowing DistillerAI to act as a second reviewer with high specificity and sensitivity for the remaining unscreened records [19, 20]. Until DistillerAI predicted that > 95% of included studies had been found (about 50% of citations), a single reviewer screened all titles/abstracts and another reviewer verified all excluded records. Thereafter, we used DistillerAI with a prediction score of 0.70 likelihood for exclusion to serve as the second reviewer. Quality assurance of the process was conducted; all records that were screened by DistillerAI as a second reviewer were identified (*n* = 2574) and 20% of the sample was cross-referenced in DistillerSR to ensure inclusion/exclusion criteria were met. Particular attention was placed on records with a likelihood score of ≥ 0.5. No discrepancies were identified, indicating a high specificity and sensitivity of DistillerAI as a second reviewer for this review.

One reviewer screened the grey literature and reference lists, with any potentially relevant study sent for full-text review by two reviewers. For full-text selection, a single reviewer reviewed all records, with all exclusions verified by another reviewer and the use of an arbitrator in case of disagreement. An exception was made if the abstract only mentioned the quality of life (*n* = 482 studies) where one reviewer assessed full text for use of any utility-based measurement tool; the dual review was then used for relevant studies reporting utilities. We contacted authors (by email once with one reminder) to confirm eligibility where this was unclear; this mainly occurred for non-HSUV studies when it was unclear what information, if any, was provided on the expected magnitude of outcomes from screening. Both title/abstract (100 citations) and full text (20 citations) screening were piloted by all reviewers involved. Studies were further verified for inclusion during data extraction. The flow of literature and reasons for full text exclusions were recorded.

#### Data extraction

Data extraction forms were piloted (at least five studies of various methods) by all reviewers involved in extraction. Thereafter, one reviewer independently extracted data from each newly included study; a second (senior) reviewer verified all data for accuracy and completeness. Disagreements on study and population characteristics (e.g., classification of exposure for HSUVs, details on presentation of information) and data results were resolved through discussion or consultation with a third reviewer until consensus was reached. For studies included in the previous review, we reviewed the study characteristics and results to determine if there were additional items or results to consider for this update.

Details about the population and study characteristics included the following: study design, country of origin, sample sizes, population(s) (including age, ethnicity, breast density, risk for breast cancer, screening history, and [if relevant] cancer stage and context e.g., current/previous/hypothetical health state), exposure(s) (e.g., survey/interview topics, design of decision aid, HSUV instrument including country of tariff and treatment exposures of patients), information provided to participants on potential benefits and harms and other outcomes (including all numerical information and definitions of overdiagnosis and FPs), comparator(s) as applicable, and findings, as reported by study authors. Outcomes of interest are listed in Table 1; for studies reporting non-HSUV data, we decided in a post hoc manner to also extract results on any knowledge tests given to participants, to provide information related to their understanding of the data on outcomes presented. Data reported by authors on any subgroup analysis for the specific populations of interest (age, race/ethnicity, screening history, risk for breast cancer, history of a FP) were extracted. If studies compared two different versions of information/decision aids, each eligible arm was considered separately.

For HSUV data related to cancer treatment, by type of surgery, receipt of chemo- and radiation therapy, use of axillary lymph node dissection, and by stage of disease, we extracted the worst utility value within each of two time points: within 12 months of surgery (or within 18 months of diagnosis) and at 2 years or later after surgery. For each health state of interest and for any subgroup analyses, we used an 80/20 rule; for example, if ≥ 80% of patients received a treatment all were classified as receiving, if ≤20% received the treatment none were considered as receiving, and for studies with > 20% to < 80% receiving the treatment the sample was considered “mixed” for that variable. For the HSUV of a healthy screen-eligible population (for making calculations of the disutility of the other health states), we used data from before screening or a sample of unscreened people matched to those being screened, or, if necessary, from a sample after they received a negative screening test.

Tables were created with data by study and a descriptive summary was developed to summarize all study characteristics among the two major sets of data (HSUVs and non-HSUVs).

#### Risk of bias assessments

We used items as per GRADE guidance on risk of bias in studies on patient preferences, about the choice/selection of representative participants; completeness of data (participation rate and missing data); appropriate administration and choice of instrument; and analysis and presentation of methods and results (e.g., inclusion of variance measures) [4]. Two questions related to the selection of participants, about the adequacy of the participation rate and similarity between responders and non-responders, were added to the main question about appropriateness of the study sample, which was used to be specific to whether participants were highly selected, for example asked about screening attitudes while attending screening visits. Items related to the choice of instrument included, as applicable, presentation of benefit and harms data (e.g., using absolute risk data) or quality of vignettes, comprehensiveness of data (e.g., inclusion of data/estimates of overdiagnosis), and whether testing was done by authors on patients’ understanding of tasks. For HSUVs, we assessed risk of bias for each time point reported and risk was considered high if there were concerns about missing outcome data or lack of variance measures, or moderate if there were concerns about two or more other items. For non-HSUV studies, we rated the risk of bias for the entire study and risk of bias was considered high if there was poor presentation of outcome data (i.e., only relative effects and/or no data on overdiagnosis), a potentially highly biased sample (e.g., up to 20% 18–35 aged years, recruitment during screening visits), or major concerns about missing data or measurement of outcomes (e.g., not defining “positive” intentions). If not rated as high risk but providing an inadequate description of overdiagnosis (e.g., not referring to it as cancer), the study was rated as moderate risk of bias. The risk of bias form was piloted (at least 3 studies of various methods) by all reviewers involved in the assessments. Thereafter, one reviewer performed assessments and a second verified the data used for the assessment and marked any disagreements in the ratings. The reviewers met to come to a consensus on their ratings, with arbitration by a third reviewer if necessary.

Due to a general lack of protocol availability and uncertainty about any impact from missing studies on this topic, we did not assess if there was a risk of bias within each analysis from missing results within studies or from missing studies. Any potential bias from this was limited by inclusion in our synthesis of data regardless of its suitability for analysis (e.g., lack of variance measures), consideration of several types of data in our syntheses for each outcome, and a highly comprehensive search including grey literature/unpublished sources.

#### Data analysis

##### HSUV data

We charted out the exposures of interest and HSUV elicitation methods across all studies, and for treatment exposures identified which studies had within-study comparisons which we prioritized over between-study comparisons (e.g., mean utilities from studies of chemotherapy vs. studies without). We preferred mean utility values but used medians if necessary. If variance measures were not reported we used one from a similar study [21]. We combined arms in a study if they were both exposed to the same health state of interest (e.g., BCS) but varied in other unrelated factors (e.g., differing forms of nerve block during surgery). Our main analysis relied on data from indirect measurement with utility-based HRQOL tools rather than direct methods of TTO and SG which were less common. The data from the direct measurements, for example the relative utility between different treatment states, were analyzed separately and used to compare with the indirect data or provide additional information on subgroup effects.

We considered pooling estimates if two or more studies reported on the same comparison or exposure. Data using any utility-based HRQOL were pooled, as were data from direct TTO or SG methods. Pooling used a fixed-effects model with weighting by the inverse of variance; our protocol planned for the use of a random effects model but we found that many small studies (often at higher risk of bias) within this analysis were given too much weight using the random effects model. If we were not able to use a study’s data in a meta-analysis (e.g., only p values were reported), we commented on these findings and compared them with the results of the meta-analysis. Analyses were performed using Microsoft Excel, Review Manager (version 5.3), and STATA (version 14.2 or higher).

Within each analysis, if there were more than two studies we explored heterogeneity first by sensitivity analysis removing less commonly reported methods, that is HRQOL tools apart from EQ-5D, and then by subgroup analysis by risk of bias (high [e.g., missing variance data] vs. low/moderate). If high risk of bias results led to highly significant subgroup effects (*p* < 0.01), we relied on the analysis without these studies. We also performed several pre-planned stratified analyses (using our 80/20 rule) by (i) stage 0 inclusion for exposures of a new diagnosis, surgical treatments, and advanced (vs. not) stage; (ii) stage 0/I versus II/III and I/II versus III/IV for new diagnosis and advanced stage; (iii) type of adjuvant therapy (chemotherapy [+/− radiation therapy], radiation therapy, none, or mixed) for surgery and advanced stage comparisons; and (iv) type of surgery (mastectomy, BCS, or mixed) for therapy and advanced stage comparisons. Post hoc, we added subgroups for stage of disease and receipt of radiation therapy for the chemotherapy exposure, and receipt of chemotherapy for the radiation exposure.

##### Non-HSUV data

Studies were first grouped according to whether they used preference-based methods to directly measure outcome valuations (e.g., discrete choice experiments, trade-offs, rankings) or non-preference-based methods to indirectly capture preferences through intentions/uptake/attitudes to screening based on information in decision aids or other educational materials. Preference-based studies were then grouped by the types of outcomes they compared and then by the age of the participants. Non-preference studies with data on the valuation of the benefits versus the harms more generally were grouped primarily based on age (of participants and relevancy of information on outcomes provided), but also the relative magnitude of the benefit-to-harm ratio of screening in the information presented to women and by the risk of bias of the studies. We categorized the benefit-to-harm ratio (“net benefit”) information in each study as low, moderate, or high taking into account the relevancy of the information to the targeted age category as well as the completeness of data (e.g., the inclusion of overdiagnosis as a potential harm), magnitudes of effect, and how the presentation of the data would likely influence perceptions by women (e.g., relative effect estimates portraying higher benefit than natural frequencies). Our classification of the degree of net benefit is focused on the differences across studies, in relative terms, rather than our judgments about the magnitude of the benefits or harms portrayed (e.g., the data indicating a relatively low net benefit scenario may be considered by others to provide moderate net benefit). Despite this, data used for the low net benefit scenarios are quite well aligned with the findings of previous guideline panels that have judged the effects as indicating a small net benefit or having a close balance between benefits and harms [11]. In addition, differences in the magnitudes of effects presented often reflected different time frames examined by the underpinning data sources; for instance, the high net benefit scenarios were often based on a 20 or longer year time horizon (e.g., [22, 23]). We assumed that most study participants would be focused on the numerical data provided (e.g., 1 in 1000 vs. 1 in 200 fewer deaths) rather than also considering the duration over which these effects would occur. After the studies were sorted, we compared and contrasted findings within the relevant studies, with consideration of the sample sizes, risk of bias, subgroup findings, and data on knowledge scores, to create a summary narrative statement for each data set. In terms of interpreting the proportions of patients that the findings related to, we used “all/almost all” (≥ 90%), a “large majority” (≥ 75%), and a “majority” (≥ 50%).

#### Certainty of the evidence

We used GRADE methods guidance for patient preferences to assess the certainty of evidence [4, 5]. All outcomes started at high certainty and were rated down, as applicable, if there were serious concerns about the risk of bias, inconsistency (or lack of consistency in the case of single studies), indirectness (all non-preference-based studies were rated down for this domain), or imprecision (i.e., wide confidence intervals for HSUVs, small sample sizes or uncertainty about the proportion of patients the data referred to in non-HSUV studies). In some cases, we had some but not serious concerns for one or more domains; if we had some concerns for two domains, we rated down the evidence by one level. In cases where studies of HSUVs reporting on similar exposures could not be pooled in meta-analysis, we used guidance for rating the certainty of evidence in the absence of a single estimate [24]. One reviewer initially assessed the certainty of evidence and at least two reviewers came to a consensus on final judgments.

We chose to use standard wording to describe the level of certainty of each finding. For findings of high, moderate, and low certainty evidence, we use “will,” “probably,” and “may,” respectively, in our narrative statements about the findings [25]. For very low-certainty findings, we report that the evidence is very uncertain without describing any study findings.

#### Involvement of contributors and stakeholders

The working group members, including clinical experts and patient partners external to the task force and employees of the Public Health Agency of Canada, helped inform the eligibility criteria. The task force members of the working group chose the outcomes based on ratings of their importance. The contributors were not involved in the selection of studies, extraction of data, appraisal of risk of bias, nor in the synthesis of data, but contributed to the interpretation of the findings and commented on the draft report. They were called upon to help inform judgments when assessing certainty (e.g., related to directness to Canadian practice or populations). A draft of this manuscript was sent to various stakeholders across Canada. All comments from the 31 respondents were considered and several modifications were made, most focusing on clarifying the interpretation of utility values and the methods (e.g., modifications made to risk of bias tool) or expanding on the review conclusions and limitations, but none leading to changes to the summary statements or certainty assessments (Supplementary file 1).

## Results

### Literature flow

**Fig. 1:**
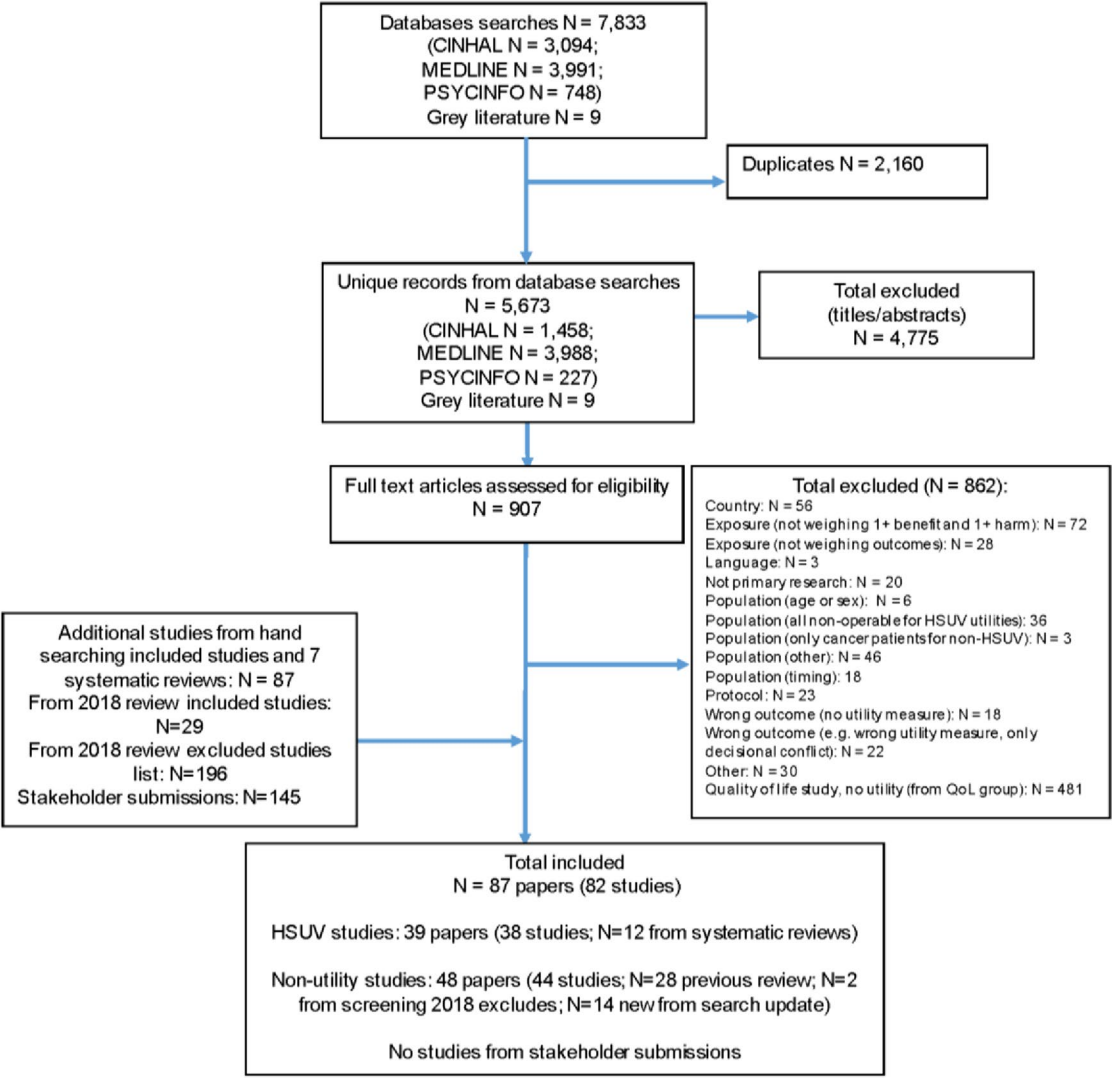
Literature flow diagram

Our database searches retrieved 5673 unique citations. Nine citations were found from grey literature sources, all of which were ineligible for inclusion. After reviewing 907 full texts from these searches, 862 studies were excluded for reasons (Supplementary file 1). 428 records were screened from seven existing systematic reviews, our previous review’s excluded studies list, and stakeholder submissions. All of the eligible studies submitted by stakeholders were also found in our searches. Overall, we included 82 studies with five additional associated publications (Figure 1); 28 of these were included in the previous review. One of the previously included studies was excluded because more than 20% of the participants were below 35 years of age which was a revised criterion for this update [26].

### Health state utility values

#### Study characteristics

For HSUVs, we included 38 studies [27–64] and one associate paper [65]. The mean age across studies was 56.7 years (≥ 70 years in 4 studies [27, 46, 53, 57]), and the total number of participants was 22,952 (range 30 to 8287). Studies came from the Netherlands [31, 39–43, 59], the USA [27, 28, 37, 55, 57, 60], South Korea [38, 44, 49, 61], Finland [51, 52, 54, 65], the UK [36, 53, 62], Australia [29, 34, 63], Japan [33, 58], Spain [45, 47], Norway [48], Italy [50], Croatia [64], Thailand [56], France [35], Greece [32], England and Wales [46], and multiple countries (UK, USA, Japan, France, Germany, Italy, and Spain) [30]. Other demographic information including race/ethnicity, comorbidities, and family history was scarce and not consistently reported. Of seven studies reporting on race/ethnicity, the percent of non-Caucasian/White participants was 0.8 to 60.7%. Recruitment strategies usually included cancer networks/centers or outpatient clinics.

Thirty studies used the EQ-5D index score with patient samples and three of four studies using direct methods (TTO and SG) used vignettes with a public sample (see Supplementary file 2 for details). One study using the EQ-5D visual analogue scale (VAS) in a public sample was included in a post hoc manner to assess the utility of interval breast cancers as this health state was not reported in any of the other included studies.

Thirteen (34.2%) of the 38 studies were at high risk of bias for at least one timepoint of interest. Nine studies solely focused on screening health states, 25 were focused on treatment health states, and four studies included health states on both screening and treatment.

Table 2 summarizes the main findings and their certainty from the primary analysis of HSUVs relying on the utility-based HRQOL tools and supported by TTO and SG findings where available. Supplementary file 2 contains tables of the study characteristics, the risk of bias ratings, and a full summary of findings tables which include all findings from the main, sensitivity, and subgroup analyses. Detailed results are presented here when the evidence was rated to have low or higher certainty.

**Table 2:** Summary of findings on health state utilities

**Number of included studies in main analysis using utility-based HRQOL tools** **Sample size** **Data from direct methods (TTO/SG), if applicable**	Certainty*		**What does the evidence say?**
**Healthy eligible comparator**			
*N* = 3 studies [60, 61, 64] *N* = 8556	⊕⊕⊕⊝ MODERATE^b^ (for individuals 40-70 years) ⊕⊕⊝⊝ LOW^b,c^ (for individuals 70+ years)		The utility value for a healthy comparator, eligible for BC screening and aged 40–70 years is probably 0.94. The utility value for a healthy comparator, eligible for BC screening and over 70 years of age may be 0.94, but there is less certainty for this value.
**Disutility of screening test process (after screening but before screening results)**			
No evidence	No evidence		No evidence
**Disutility of positive screening mammography (before diagnostic testing)**			
*N* = 3 studies [32, 59, 60] *N* = 565 participants	⊕⊕⊕⊝ MODERATE^a,b^		The disutility value for a positive screening mammography is probably 0.07.
**Disutility after biopsy (diagnostic results not known)**			
*N* = 1 study [32] *N* = 102 participants	⊕⊝⊝⊝ VERY LOW^a,b,d^		We are very uncertain about the disutility of receiving a biopsy, before the results are known.
**Disutility of knowledge of a false positive requiring imaging only or imaging plus biopsy**			
*N* = 2 studies [59, 60] *N* = 696 participants	⊕⊕⊝⊝ LOW^a,c^		The disutility value for having knowledge of a false positive requiring imaging only or imaging plus biopsy may be 0.03 to 0.04.
**Disutility of false-positive result requiring imaging plus biopsy**			
*N* = 1 study [32] *N* = 78 participants	⊕⊝⊝⊝ VERY LOW^a,b,d^		We are very uncertain about the disutility of a false positive result after invasive testing.
**Disutility of a true-positive result, before treatment**			
*N* = 9 studies [31, 35, 44–46, 52, 53, 61, 64] *N* = 6657 participants Direct methods: *N* = 1 [53] SG, *n* = 156	⊕⊕⊕⊝ MODERATE^b,c^		The disutility of a screen-detected cancer is probably on average 0.08, but may be higher for older ages and advanced stage operable cancer.
**Disutility of an interval cancer**			
*N* = 1 study (using VAS) [28] *N* = 131 participants	⊕⊕⊝⊝ LOW^a,b,d^		The disutility for interval cancer may be similar to a screen-detected cancer.
**Disutility of mastectomy vs. BCS/partial mastectomy (≤ 12 months from surgery)**			
Within study: *N* = 3 studies [29, 47, 52] *N* = 1546 participants Direct methods: *N* = 2 studies [29, 39] SG/TTO, *N* = 293	⊕⊕⊝⊝ LOW ^a,b^	⊕⊕⊝⊝ LOW^a,b^	The disutility of a mastectomy versus a BCS/partial mastectomy (all patients receiving adjuvant treatments) may be at least 0.02 to 0.03. We are very uncertain about the disutility from mastectomy without adjuvant treatment vs. BCS/partial mastectomy with radiation.
Between study: BCS, *N* = 5 studies [27, 29, 47, 52, 63] *N* = 1682 participants Mastectomy, *N* = 7 studies [29, 33, 47, 49, 52, 61, 62] *N* = 1942 participants Direct methods: *N* = 3 studies [29, 39, 56] TTO/SG, *N* = 696	⊕⊕⊝⊝ LOW^a,b^		
**Disutility of adjuvant chemotherapy vs. none (≤12 months from surgery)**			
Within study: *N* = 2 studies [36, 53] *N* = 1011 participants	⊕⊕⊝⊝ LOW^a,d^	⊕⊕⊝⊝ LOW^b,c^	The disutility of adjuvant chemotherapy may be 0.02–0.04 among a mixed surgical population.
Between study: Adjuvant chemotherapy: *N* = 7 studies [36, 43, 47, 53, 58, 61, 65] *N* = 1234 participants (1 study *N* = NR by arm, *N* = 231 overall [36]) No adjuvant chemotherapy: *N* = 5 studies [36, 38, 46, 51, 53] *N* = 2447 participants (1 study *N* = NR by arm, *N* = 231 overall [36])	⊕⊕⊝⊝ LOW^b,c^		
**Disutility of adjuvant radiation vs. none (≤12 months from surgery)**			
Within study: *N* = 4 studies [29, 36, 62, 63] *N* = 1587 participants Direct methods: *N* = 1 study [29] SG, *N* = 1 72	⊕⊕⊕⊝ MODERATE^b^	⊕⊕⊕⊝ MODERATE^b^	There is probably little-to-no disutility from adjuvant radiation, among those receiving BCS/partial mastectomy or mastectomy, where many are receiving chemotherapy.
Between study: Adjuvant radiation: *N* = 8 studies [27, 29, 36, 47, 52, 61–63] *N* = 2174 participants (1 study *N* = NR by arm, *N* = 231 overall) No adjuvant radiation: *N* = 8 studies [29, 33, 34, 36, 52, 58, 62, 63] *N* = 1547 participants Direct methods: *N* = 3 studies [29, 39, 55] TTO/SG, *N* = 449	⊕⊕⊝⊝ LOW^B^		
**Disutility of ALND vs. SLND (≤12 months from surgery)**			
Within study: No evidence	No evidence		No evidence
Between study: No evidence	No evidence		
**Disutility of advanced vs. not advanced stage (stages II–III vs. I) (≤12 months from treatment initiation)**			
* N* = 2 studies [30, 50] *N* = 1412 participants	⊕⊕⊝⊝ LOW^b,d^		There may be a disutility of 0.02, from having stage II–III versus stage I breast cancer among a mixed surgical and adjuvant treatment population.
**Disutility of advanced vs. not advanced stage (stage III vs. I–II) (≤12 months from treatment initiation)**			
*N* = 2 studies [30, 50] *N* = 1412 participants Direct methods: *N* = 1 study [39], *N* = 121	⊕⊕⊝⊝ LOW^b,d^		There may be a disutility of 0.03, from having stage III versus I–II breast cancer among a mixed surgical and adjuvant treatment population.
**Disutility of mastectomy vs. BCS/partial mastectomy (≥ 2 years from surgery)**			
Within study: *N* = 5 studies [37, 40, 41, 52, 57] *N* = 3820 participants	⊕⊕⊕⊝ MODERATE^c^	⊕⊕⊕⊝ MODERATE^c^	There is probably little-to-no disutility from mastectomy versus BCS/partial mastectomy with radiation >2 years from surgery. This may be most applicable after at least 5 years post-surgery.
Between study: BCS, *N* = 6 studies [37, 40–42, 52, 57, 61] *N* = 2017 participants Mastectomy, *N* = 6 studies [37, 40, 41, 52, 57, 62] *N* = 2702 participants	⊕⊕⊕⊝ MODERATE^c^		
**Disutility of adjuvant chemotherapy vs. none (≥ 2 years from surgery)**			
Within study: no evidence	No evidence	⊕⊝⊝⊝ VERY LOW^b,d^	We are very uncertain about the disutility of adjuvant chemotherapy versus none >2 years from surgery.
Between study: Adjuvant chemotherapy: *N* = 2 studies [54, 61] *N* = 272 participants No adjuvant chemotherapy: *N* = 1 study [57] *N* = 278 participants	⊕⊝⊝⊝ VERY LOW^b,D^		
**Disutility of adjuvant radiation vs. none (≥ 2 years from surgery)**			
Within study: *N* = 2 studies [57, 62] *N* = 1183 participants	⊕⊕⊕⊝ MODERATE^d^	⊕⊕⊕⊝ MODERATE^b^	There is probably little-to-no disutility from adjuvant radiation versus none >2 years from surgery.
Between study: Adjuvant radiation: *N* = 9 studies [37, 40–42, 48, 52, 57, 61, 62] *N* = 5646 participants No adjuvant radiation: *N* = 4 studies [37, 41, 57, 62] *N* = 838 participants	⊕⊕⊕⊝ MODERATE^b^		
**Disutility of ALND vs. SLND (≥ 2 years from surgery)**			
Within study: no evidence	No evidence		No evidence
Between study: no evidence	No evidence		
**Disutility of advanced vs. not advanced stage (> 2 years from treatment initiation)**			
No evidence	No evidence		No evidence

^*^Reasons for rating down certainty: ^a^risk of bias, ^b^inconsistency/lack of consistency, ^c^indirectness, ^d^imprecision; the use of capitals indicates there was a very serious concern for the domain; for three exposures (positive screening mammography, true positive result, interval cancer), there was only some concern for two of the domains

*ALND*, axillary lymph node dissection; *BC*, breast cancer; *BCS*, breast-conserving surgery; *HRQOL*, health-related quality of life; *SG*, standard gamble; *SLND*, sentinel lymph node dissection, *TTO*, time trade-off; *vs*, versus

#### Screening health states

Disutilities from outcomes during the breast cancer screening process were calculated in comparison with (subtracted from) the pooled utility (0.94) of a healthy screen-eligible population including individuals scheduled for genetic counseling (*n* = 33; results of genetic testing unknown), with a known negative screening result (*n* = 531), and healthy age and education matched comparators (*n* = 7992) [60, 61, 64]. This estimate had moderate certainty due to a lack of consistency from large reliance on one study [61]. When comparing results with trends observed in population norms (not all eligible for screening) for a Canadian province (Alberta) [66], we had low certainty (from indirectness to older ages) that the utility of 0.94 applies well to those over 70 years of age.

During the breast cancer screening process, the disutility of positive screening mammography (before diagnostic testing) is probably 0.07, with a rating down one level for some concerns of inconsistency and risk of bias [32, 59, 60]. Two of the studies excluded people who were later known to have cancer and the other only included women who’s screening mammography indicated low suspicion for breast cancer. After receiving imaging only or imaging plus biopsy and results of no cancer diagnosis, the disutility of knowing about a FP result may be 0.03 to 0.04 [59, 60].

The disutility of a true-positive result (screen-detected cancer) is probably on average 0.08, but may be higher for older ages and advanced stage operable breast cancer [31, 35, 44–46, 52, 53, 61, 64]. Removing two studies at high risk of bias did not impact the findings. There were some concerns about unexplained inconsistency; subgroup data based on age and stage of disease (including data from a SG; *n* = 156 [55]) was limited but suggested higher disutility with older ages and advanced-stage operable disease. There was also some indirectness because data were not specific to screen-detected cases, though concerns were not serious because the seven studies that reported stage of disease confirmed there were very few stage IV (0–0.7%) cancers which are not typically identified during screening.

Interval cancers were reported in one study using a hypothetical scenario and evaluated by VAS, where the disutility may be similar to screen-detected cancer (*n* = 131; low certainty) [28].

#### Treatment health states < 12 months from surgery

Utility values within 12 months from surgery for breast cancer were compared across different surgeries and different adjuvant therapies, to estimate the disutility of one treatment versus another. The disutility of mastectomy versus a BCS/partial mastectomy where all patients are receiving adjuvant therapy may be at least 0.02 to 0.03 [27, 29, 33, 47, 49, 52, 61–63]. The range in disutility values comes from the within-study estimate of 0.03 and the between-study estimate of 0.02 [mastectomy HSUV 0.80 vs. BCS 0.82]. All studies had at least 20% of patients receiving adjuvant therapies. Concerns included the risk of bias and unexplained inconsistency based on the types of adjuvant therapies received as well as an indication from direct measurements (in public samples) that the disutility may be higher (0.05 to 0.10) [29, 39, 56]. When looking at findings among subgroups where patients were all receiving chemotherapy or all receiving radiation therapy, thus controlling for these variables, the disutility of a mastectomy versus BCS was higher (0.04 to 0.08) but findings were limited by few studies. We are very uncertain about a major comparison of interest which was mastectomy without adjuvant therapy (no evidence) versus BCS/partial mastectomy with adjuvant radiation (a choice often provided for small node-negative estrogen-receptor-positive breast cancer, a common cancer found by screening).

The disutility of adjuvant chemotherapy versus no chemotherapy may be 0.02 to 0.04 among a mixed surgical population within 1 year from surgery, due to concerns of inconsistency and indirectness from reliance for our estimate on between-study comparison data [36, 38, 43, 46, 47, 51, 53, 54, 58, 61]. Subgroup analysis by type of adjuvant chemotherapy used was not possible due to insufficient reporting. There were no apparent outlier effects (lower/higher values) based on timepoint used in studies. The pooled estimate for no adjuvant chemotherapy relied on studies not at high risk of bias (after removing 2 studies). The findings indicated a range of disutility, with the lower estimate (0.02) from the difference in pooled effects for the between-study comparisons (HSUVs of 0.85 vs. 0.87) and the upper estimate (0.04) from comparisons controlling for radiation use and for inclusion of stage 0 (i.e., studies without patients having stage 0 were thought most relevant to chemotherapy treatment and indicated higher disutility from chemotherapy).

Comparing adjuvant radiation use versus none within 1 year from surgery, there is probably little-to-no disutility among those receiving BCS/partial mastectomy or mastectomy, where many are also receiving adjuvant chemotherapy [27, 29, 33, 34, 36, 47, 52, 58, 61–63]. The within- (disutility 0.01) and between-study (HSUVs 0.80 vs. 0.81) analyses estimates were similar. There were concerns of inconsistency across studies contributing to this disutility measure, particularly when the type of surgery differed. Findings relied mostly on within-study comparisons, though between-study comparisons had similar findings when studies where most patients (>80%) were receiving chemotherapy were removed. Removing studies with a high risk of bias did not impact any findings. Similar findings within surgical subgroups were found in studies using TTO and SG [29, 39, 55]; the scenarios in these studies did not describe any receipt of chemotherapy and findings suggested that there may be a disutility from radiotherapy among those not receiving chemotherapy.

Two studies reported within study utilities for treatment by stage [30, 50]. Neither study included patients with stage 0 or IV disease and both studies were considered to include a mixed surgical and adjuvant therapy population. Within 1 year from surgery, among mixed surgical and adjuvant treatment populations stages II–III versus stage I may have a disutility of 0.02 and stage III versus I–II may have a disutility of 0.03 [30, 50]. For these findings, there was a lack of consistency, due to one study [30] being heavily weighted in the analyses, and concern over imprecision. Neither study was at high risk for bias.

#### Treatment health states > 24 months from surgery

At more than 2 years after surgery, there is probably little-to-no disutility from mastectomy versus BCS/partial mastectomy with adjuvant radiation [37, 40–42, 52, 57, 61, 62]. The within- (disutility 0.00) and between-study (HSUVs 0.83 vs. 0.84) analyses estimates were similar. Most studies used timeframes over 5 years after surgery so the findings may be most applicable to at least 5 years post-surgery. The within- and between-study analyses agreed with each other, once the high risk of bias studies were removed from the between-study analyses. There were concerns of indirectness due to most data for mastectomy reported by patients using mixed therapies.

Findings indicated that there is probably little-to-no disutility from adjuvant radiation versus none at long-term follow-up, with moderate certainty due to concerns of inconsistency across studies [37, 40–42, 48, 52, 57, 61, 62]. The within- (disutility 0.00) and between- (HSUVs 0.80 vs. 0.81) study analyses estimates were similar; findings for the between-study analyses relied on studies not at high risk of bias (removed after sensitivity analysis). Subgroup findings based on type of surgery were consistent with little-to-no disutility in the within-study analyses but inconsistent in the between-study analysis, finding a utility from radiation among those receiving BCS and a disutility among those receiving mastectomy.

### Non-health state utility values

#### Study characteristics

We included 44 studies [67–110] and four associated papers [111–114] that examined preference-based and other information to inform the relative importance of the potential outcomes from screening. Details about the included studies are described within each of the sections below. Supplementary file 3 contains tables with the study characteristics and risk of bias ratings and more complete summary of findings tables with the associated study findings.

#### Direct comparisons between outcomes via preference-based data

Table 3 summarizes the findings and certainty of evidence from preference-based studies, by outcomes compared and relevant age group.

**Table 3:** Summary of findings from other preference-based studies, by outcomes compared and relevant age group

**Included studies** **Sample size**	Certainty*	**What does the evidence say?**
**All-cause vs. BC mortality**		
**50- to 69-year-olds**		
2 studies [74, 94] *N* = 1019 (range 106–913)	⊕⊕⊝⊝ LOW^c,d^	For patients aged 50 to 69 years, a large majority (>75%) of patients may think that reducing breast cancer mortality is beneficial even if there is no impact on all-cause mortality. The evidence was limited to relatively high rates of breast cancer mortality reductions (2 and 5 fewer per 1000).
**BC mortality vs. overdiagnosis**		
**Across all ages**		
5 studies [83, 102, 103, 106, 109] *N* = 2652 (range 50–810)	⊕⊕⊕⊝ MODERATE^c^	For patients aged 40 years or older, at least a majority (> 50%) and possibly a large majority (> 75%) of patients probably accept up to 6 cases of overdiagnoses to prevent one death from breast cancer.
** 50- to 69-year-olds**		
2 studies [82, 108] *N* = 1833	⊕⊕⊝⊝ LOW^b,c^	For patients aged 50 years and older, a large majority of women may accept at least 3 overdiagnoses to prevent one death from breast cancer though an upper limit was not examined.
**BC mortality vs. FPs**		
**Across all ages**		
3 studies [74, 100, 109] *N* = 675 (range 90–479)	⊕⊕⊝⊝ LOW^a,d^	For patients aged 40 years or older, there may be considerable variation in preferences though almost all patients may accept that 25–50 and a majority may accept that a few hundred among 1000 experience a FP result to prevent one death from breast cancer over 10 years.
**40- to 49-year-olds**		
2 studies [87, 90] *N* = 272	⊕⊕⊝⊝ LOW^b,c,d^	For patients in their 40s, at least a majority of patients probably accept at least 100 and may accept at least 300 FPs per breast cancer death prevented over 10 years.
**50- to 59-year-olds**		
3 studies [79, 82, 110] *N* = 1483	⊕⊕⊝⊝ LOW^a,b^	For patients 50–59 years of age, even in scenarios of relatively high reductions in breast cancer mortality, FP rates of 80–120 or higher per 1000 may be important information for a large minority of patients when making decisions about screening.
**BC mortality vs. FP biopsies**		
**Across ages**		
1 study [102] *N* = 812	⊕⊕⊝⊝ LOW^b,d^	For patients aged 40 years or older, a large majority of patients may accept that between 10 and 15 people experience a FP biopsy to prevent one death from breast cancer over many years. This trade-off may be an overestimate for what is acceptable over a 10-year timeframe.
**Stage distribution (reduced advanced disease) vs. FPs**		
**Across ages**		
3 studies [70, 77, 84] *N* = 2881	⊕⊕⊝⊝ LOW^a,c^	For patients aged 40 years or older, a large majority of patients may accept that at least 25 people experience a FP to prevent one advanced stage breast cancer.
**Stage distribution (reduced advanced disease) vs. FP biopsies**		
**Across ages**		
2 studies [77, 84] *N* = 2481	⊕⊕⊝⊝ LOW^a,c^	For patients aged 40 years or older, a large majority of patients may accept that at least 4 people experience a FP biopsy to prevent one advanced-stage cancer.
**Treatment-related morbidity (reduced mastectomy) vs. FP **		
**Across ages**		
1 study [70] *N* = 400	⊕⊕⊝⊝ LOW^b,d^	For patients 40 or older, avoiding mastectomy may be much more important than experiencing a FP for a majority of patients.

^*^Reasons for rating down certainty: ^a^risk of bias, ^b^inconsistency/lack of consistency, ^c^indirectness, ^d^imprecision; for BC mortality versus FPs, there were only some concerns about inconsistency and imprecision

*BC* breast cancer,* FP* False positive, *vs* Versus

##### All-cause mortality versus breast-cancer mortality

Two studies provided data to inform how women value the effects of screening on all-cause versus breast cancer mortality. One study used computer-assisted telephone interviews based on a convenience sample from primary care clinics (*n* = 106; 45–70 years; 91% previous screening) with sequential presentation of screening scenarios with information on the benefits (no harms presented) on (i) breast cancer mortality using relative terms (34% reduction), (ii) breast-cancer mortality using absolute terms (4 in 1000 screened vs. 6 in 1000 not screened over 10 years), and (iii) all-cause mortality (“screening will reduce deaths from breast cancer but will not increase your chance of living longer”) [74]. An RCT studied an online decision aid in those aged 50, at first invitation (*n* = 913; 50 years, 33% previously screened), and provided information that screening would lead to 1 fewer breast cancer death and no reduction in all-cause mortality among 200 screened for 20 years (=5 fewer among 1000 screened) versus 50 FPs (=250 per 1000 screened) and 1 overdiagnosis in 200 screened (=5 among 1000 screened) (duration of harms not noted) [94]; this study did not use preference-based methods (e.g., trade-offs, ratings) but was considered to provide relevant information directly comparing these two specific outcomes. In the interviews, women were somewhat less willing to be screened after being presented with information on all-cause mortality (definitely = 53% and probably = 31%) than after information on breast cancer mortality (definitely = 78% and probably = 14%) and only 16% of participants stated that the information on all-cause mortality should definitely be presented to women (40% stated probably), compared with 73% and 20% when asked about the absolute effects of breast cancer mortality. In the RCT, there were positive intentions to screen for 82% of participants after reading the decision aid, 83% had a positive attitude about screening, and at 3 months 65% of the women had attended the screening. Findings across these studies suggest that a large majority (> 75%) of women aged 50 to 69 years may appraise screening as beneficial even if told that it reduces breast cancer but not all-cause mortality. Certainty was low due to the indirectness of the data (i.e., one population of screeners and potential confounding from other information presented to women in the RCT and imprecision around the estimate of a large majority which may be an overestimate). Screening was portrayed as relatively beneficial for reducing breast cancer in these studies though we did not rate down further for this factor.

##### Mortality from breast cancer versus overdiagnosis

Our main analysis included three studies (*N* = 1663) of mixed age groups from community samples using (i) an online survey employing choice sets to determine a maximum acceptable ratio of overdetection to breast cancer death avoided among four treatment scenarios (Netherlands and Australia; *n* = 803; low risk of bias) [103], (ii) focus groups on acceptance of different levels of overdiagnosis given the 10-year benefits of screening across age groups (Australia; *n* = 50; low risk of bias) [83], and (iii) an online discrete choice experiment (DCE) calculating trade-offs within a screening program stopping at age 74 years (France; *n* = 810; moderate risk of bias for description of overdiagnosis only involving ductal carcinoma in situ [DCIS]) [102]. The two large studies were new to this update. In the survey study (45–75 years; mean 58.3 ± 8.9; oversampled 45–50 years), 50–57% (varying across types of treatment used for overdiagnosis) stated they would always participate in screening, even with a 1:6 ratio of breast-cancer deaths avoided to cancers overdiagnosed. There were no associations between acceptance and age, previous experience of a FP or FP biopsy, or having a friend or relative with breast cancer. Previous screening was associated with higher acceptance of overdiagnosis for all scenarios (*p* < 0.001). Only 33% of participants in this study were correct when asked to identify the definition of overdiagnosis in a knowledge test. Among the focus group participants (ages 40–79 years; 38% <50 years), 30% overdiagnosis (i.e., 11 among 38 cancers; on average 5:1 ratio compared with benefits portrayed) was acceptable and of limited impact and 50% overdiagnosis (i.e., 19 among 38 cancers; 10:1) was thought to possibly deter some women, especially younger women, or necessitate careful consideration by others. In the DCE (40- to 74-year-olds; 37% <50 years), a mean of 14.1 overdiagnoses were acceptable for preventing one death from breast cancer; a majority (>50%), a large majority (≥75%), and almost all (≥ 90%) would accept < 10:1, ≤ 6:1, and ≤ 4:1. The studies consistently found that at least a majority of patients will accept up to 6 cases of overdiagnosis to prevent one breast cancer death. Though somewhat inconsistent, the number accepting this ratio may be a large majority. There is uncertainty about whether these trade-offs would be acceptable in situations where the outcome was well understood and in view of this we rated down for indirectness (from using an uninformed exposure). The findings appear to apply across 40- to 70-year-olds.

Two other studies among mixed age groups at high risk of bias were examined but not considered to inform the main analysis: an online survey eliciting simple trade-offs (UK; *n* = 510; mean 46.9 years) [106] and a study asking about the relative importance of these outcomes when making decisions based on a decision aid (Hong Kong; *n* = 90; mean age 54 years; very few previously screened; < 20% had heard of mammography) [109]. Findings from two other studies of women between 50 and 69 years of age support the above conclusions (see Supplementary file 3) [82, 108].

##### Mortality from breast cancer versus FPs and FPs after biopsy

Three studies from the previous review reported on the relative importance between breast cancer mortality and FPs across all ages. One population-based survey conducted in 2000 in the US (*n* = 479) found that FPs were highly acceptable, with 80%, 63%, and 37% of respondents saying they would accept 100, 500, or 10,000 or more FPs per life saved over a 10-year timeframe [100]. About 20% of the sample was aged under 35 years, which caused concern about the risk of bias. During telephone interviews within a primary care clinic (*n* = 106; ages 45–70 years), a ratio of 25:1 did not change willingness to screen (78%) or positive attitudes (85%) towards screening [74]. Based on the data presented, both breast cancer mortality and FPs were very important or important for most (95% and 87%) participants to know when making decisions to screen. One other study (*n* = 90) at high risk of bias reported that information about BC mortality (20% relative risk reduction) and FPs (10%) was important for decision-making in 22% and 5% of participants, respectively [109].

Findings from two US studies (*N* = 272) among women in their 40s attending primary care clinics also suggest that a majority may accept at least a few hundred FPs to prevent one breast cancer death (Supplementary file 3) [87, 90]. An upper limit of the highest acceptable number of FPs was not evaluated. Among women 50–59 years of age, a specific trade-off could not be estimated but data from three studies (*N* = 1483) indicated that even in scenarios of relatively high reductions in breast cancer mortality, FP rates of 80–120 or higher per 1000 may be important information for a large minority of patients when making decisions about screening [79, 82, 110].

In the DCE (*n* = 810) using a mixed-age community sample also included above for comparing mortality with overdiagnosis, the mean willingness-to-accept value for FP biopsies per prevented breast-cancer death was 47.8 when screening until age 74; 95% accepted between 6.7 and 127.3 FP biopsies; 92% 10 FP biopsies, 63% 20 FP biopsies, and 48% 30 FP biopsies [102]. Those with regular screening history were willing to accept more FPs (22% higher). The estimated trade-off of 10–15 for a large majority of patients may be an overestimate for what is acceptable over a shorter timeframe.

##### Stage distribution (reduced advanced disease) versus FPs and FP biopsies

Three studies reported on the relative importance between stage distribution and FPs. In Singapore, trained interviewers administered a DCE (*n* = 400; 40- to 64-year-olds; 52.1 ± 7.3 years) with attributes including stage distribution (i.e., breast cancer survival rates of 25% [late stage], 50%, 65%, and 90% [early stage]) and FPs (5%, 15%, and 30%) [70]. Independent of the other attributes, when cancer survival rates changed from 25 to 90%, 14.5% more participants stated they would undergo screening; uptake only increased by 1.4% when the FP rate was reduced from 30 to 5% (25-unit change). Two older US studies (excluded in the last review due to no weighing of mortality) employed the same questionnaire to determine willingness to accept more FPs (15% vs. 10%) in order for the chance that if cancer is diagnosed it may be detected earlier (described as 1 in 200 cancers found vs. 1 in 300) (i.e., 50 more FPs vs. 2 cancers detected earlier per 1000) [77, 84]. Earlier detection is assumed to be interpreted as smaller and more curable cancer, but is judged as indirect for the advanced stage outcome. In one of the studies enrolling 97% Caucasians (*n* = 1570, 41% 40–49 years), the large majority (86%) agreed that this trade-off would be acceptable [77]. Subgroups found small differences (5% at most) between groups for the variables of previously screened, previous FP or invasive procedures, age (< 60 vs. ≥ 60 years), and family history of breast cancer. The other study was undertaken among an underserved and predominantly minority population (*n* = 911, 32% 40–49 years), with more White than Black and Hispanic women agreeing (76% vs. 54% and 59%) and fewer being unsure (11% vs. 27% and 24%) about the trade-off’s acceptance [84]. Both studies used clinical samples of patients aged 40 or older, and participants were recruited while attending their mammography appointment which was considered a risk of bias. This questionnaire was not tested by potential participants for its understanding. Many women in both studies greatly overestimated the chance of breast cancer being detected on one screening visit which may have further impacted their answers. These two US studies also examine early diagnosis compared with FP biopsies (data representing 8 more FP biopsies to detect 2 cancers earlier, per 1000), finding that a large majority of patients (possibly fewer in Black and Hispanic women) may accept that at least four people experience a FP biopsy to prevent one advanced stage cancer. Low certainty evidence suggested that a large majority of women may accept at least 25 FPs and 4 FP biopsies to save one life from breast cancer.

##### Treatment-related morbidity versus FPs

The DCE from Singapore (*n* = 400) also included an attribute related to type of surgery (3 levels: no change, changes in feel/appearance of breast, or lose an entire breast) for comparisons with FPs (5%, 15%, and 30%) [70]. Independent of the other attributes, compared with no change, not losing a breast increased acceptance by 4.8%, and not having a change in appearance increased acceptance by 2.1%, compared with the increased acceptance of 1.4% with a large change in FPs from 30 to 5%. Findings suggested that avoiding mastectomy may be much more important than experiencing a FP for a majority of patients.

#### Indirect comparisons of the relative importance of the potential benefits and harms

Data for this group of findings was categorized by its relevance to age and to our assessments about the benefit-to-harm ratio (net benefit) of the information provided to participants. In relation to the main outcomes considered important for decision-making by the task force, studies generally provided data about the expected effects of screening on breast cancer mortality, FPs, and overdiagnosis. Few studies commented on the possibility of lower treatment morbidity from detecting cancer earlier and avoiding some treatments such as chemotherapy. Apart from two studies of women in their 40s [89, 92], one of 50-year-old women [94], and one of 70–71-year-olds [88], studies did not provide information about all-cause mortality. False positives were usually described as abnormal findings (or suspicious of cancer) requiring more tests that show there is no cancer, without mention that in many cases the screening test result could also be incomplete or probably benign. Findings of at least low certainty for women starting (40-49 years) and stopping (70 years and older) screening are presented in detail here, but Table 4 contains all summary statements and Supplementary file 3 contains a detailed summary of findings tables for all categories and a full narrative for all data for women 50–69 years of age.

**Table 4:** Summary of findings from studies providing indirect evidence on preferences between potential benefits and harms of screening

**Included studies** **Sample size**	Certainty*	**What does the evidence say?**
**40 to 49-year-olds**		
**Relatively low net benefit scenario**		
3 studies [89, 92, 96] *N* = 459	⊕⊕⊝⊝ LOW^c,d^	In a relatively low net benefit scenario, a majority of patients in their 40s may *not* weigh the benefits as greater than the harms from screening.
**Relatively moderate net benefit scenario**		
1 study [105] *N* = 2120	⊕⊝⊝⊝ VERY LOW^b,C^ ⊕⊕⊝⊝ LOW^b,d^	In a relatively moderate net benefit scenario, it is unclear how patients in their 40s weigh the benefits as greater than the harms from screening. Information on overdiagnosis may be quite important for many women.
**Relatively high net benefit scenario**		
6 studies [75, 76, 86, 95, 97, 101] *N* = 4826	⊕⊕⊝⊝ LOW^b,c^	In a relatively high net benefit scenario, a majority but possibly not a large majority of patients in their 40s may weigh the benefits as greater than the harms from screening. Preferences may be similar for patients with different levels of breast cancer risk.
**50- to 69-year-olds**		
**Relatively low net benefit scenario**		
**Focus on 50- to 59-year-olds**		
3 studies [68, 81, 105] *N* = 2481	⊕⊕⊝⊝ LOW^b.c^	In a relatively low net benefit scenario, a large majority of 50- to 59-year-old patients may weigh the benefits as greater than the harms from screening.
**Relatively moderate net benefit scenario**		
**Focus on 50-year-olds**		
1 study [82] *N* = 879	⊕⊕⊕⊝ MODERATE^c^	In a relatively moderate net benefit scenario, a majority and possibly a large majority of patients 50 years old probably weigh the benefits as greater than the harms from screening.
**Ongoing screening in 50 to 69-year-olds**		
1 study [67] *N* = 20	⊕⊝⊝⊝ VERY LOW^a,b,c^	In a relatively moderate net benefit scenario, it is uncertain how 50- to 69-year-old patients weigh the benefits versus harms of screening.
**Relatively high net benefit scenario**		
**Focus on 50-year-olds**		
5 studies [69, 78, 93–95] *N* = 6904	⊕⊕⊕⊝ MODERATE^c^	In a relatively high net benefit scenario, a large majority of 50-year-old patients probably weigh the benefits as greater than the harms from screening.
**Ongoing screening in aged 50-69 years**		
6 studies [71, 75, 80, 85, 104, 107] *N* = 16,864 (1 RCT 16,000)	⊕⊕⊕⊝ MODERATE^c^	In a relatively high net benefit scenario, a large majority of 50- to 69-year-old patients probably weigh the benefits as greater than the harms from screening.
**70 years and older**		
**Relatively moderate-to-low net benefit scenario**		
**70 to 71-year-olds**		
1 study [88] *N* = 734	⊕⊕⊕⊝ MODERATE^c^	In a moderate-to-low net benefit scenario, a large majority of patients 70–71 years of age who have recently screened probably think the benefits outweigh the harms of continuing to screen.
**75 years and older**		
3 studies [73, 98, 99] *N* = 634	⊕⊕⊝⊝ LOW^c,d^	For patients aged 75 years to their early 80s who have recently screened, a majority but possibly not a large majority may weigh the benefits as greater than the harms for continuing to screen under a moderate-to-low net benefit scenario. It is unclear what impact life expectancy has on this preference.
**Relatively high net benefit scenario**		
**70 years and older**		
2 studies [72, 91] *N* = 73	⊕⊝⊝⊝ VERY LOW^a,c,d^	Under relatively high net benefit scenarios, it is uncertain how patients 70 years old and over weigh the benefits and harms.

^*^Reasons for rating down certainty: ^a^risk of bias, ^b^inconsistency/lack of consistency, ^c^indirectness, ^d^imprecision; the capital C for ratings among 40- to 49-year-olds in the relatively moderate net-benefit scenario indicates very serious concerns for indirectness from the mean age being 49.5 years

##### 40 to 49 years

Ten studies (*N* = 7405, range 12 to 2918) [75, 76, 86, 89, 92, 95–97, 101, 105], four new to this review (*N* = 3814) [86, 95, 97, 105], included patients in their 40s. Five studies were conducted in the USA [76, 96, 97, 101, 105], and one each was conducted in Spain [86], Italy [95], Canada [75], New Zealand [92], and Australia [89]. Three studies included a broader age range (*n* = 1001, aged 49.7 [SD 3.2] [95]; *n* = 2120 aged 49.5 [SD 7.8] [105]; *n* = 46, aged 35–59 [63% < 50] [75]) though in one of these [75] there were findings presented by age group.

Three studies (*N* = 459) from the previous review presented information indicating relatively low net benefit from screening (i.e., 0.5 breast cancer deaths prevented in 1000, 239–330 FPs and 2 to 10 overdiagnoses per 1000; 2 studies also portrayed 0.5 all-cause deaths prevented in 1000) [89, 92, 96]. Two studies [89, 92] enrolled community samples whereas the other [96] used a wide recruitment strategy across community and healthcare settings. All three studies were rated at moderate risk of bias either for some concerns about missing data and/or inadequate response rates, or for inadequate description of overdiagnosis (i.e., not described as including cancer). Ten of 11 women in a citizen’s jury in New Zealand changed their mind from being *for* to *against* public provision of screening for ages 40–49 [92]. A video intervention in a US study (*n* = 35) reduced scores about the benefits being greater than the harms (− 0.65 on a 5-point scale; [*p* < 0.001]) [96]. This intervention also lowered intentions to screen (pre: 85% intended and 6% unsure vs. post: 49% intended and 20% unsure). In the RCT from Australia (*n* = 412, 27% having previously screened), 39% had no intentions (18% were unsure) to screen in their 40s after using an online decision aid [89]. 95% of participants in this RCT were judged to have adequate conceptual and numerical knowledge after using the decision aid. Our summary that a majority of patients in their 40s may not weigh the benefits as greater than the harms from screening in a relatively low net benefit scenario is of low certainty because of concerns about indirectness and imprecision (i.e., a majority may be an overestimate).

One new study (*n* = 2120) at low risk of bias was judged to present a moderate net benefit scenario for this age group (i.e., 2 fewer breast cancer deaths, 160 FPs and 20 overdiagnoses in 1000 over 11 years) [105]. Though very low certainty for any outcome valuation, the findings suggested that information on overdiagnosis may be quite important for many women (Supplementary file 3).

Six studies (*N* = 4826; three new to this review) were judged to present relatively high net benefit scenarios [75, 76, 86, 95, 97, 101], with benefits presented only using relative effects (e.g., 20% reduction) or a natural frequency that was judged as high (e.g., 1 in 200 prevented breast cancer deaths, 12 vs. 20 in 1000), and/or not presenting any numerical information on overdiagnosis. One study was at low risk [86] and five were at high risk of bias. Four studies provided patients with their own predicted risk for breast cancer [76, 86, 97, 101]; in three, there was also the opportunity to discuss the information during a clinic visit [76, 86, 97]. One of these was focused on attitudes regarding personalized screening, but also examined overall attitudes about screening [86]. The other studies either examined subgroups based on the 5-year risk for breast cancer (i.e., above and below 1.1% and 1.7% 5-year risk), split their study population by low (< 1.5% risk) and elevated risk (≥ 1.5%; excluding high risk), or reported narratively on analysis by risk.

Positive attitudes regarding screening were reported as high (88% and 92%) in two studies, though one (*n* = 1001) [95] enrolled patients aged 45 and older with a mean age of almost 50 and the other (*n* = 387) [86] also found that there were positive attitudes (62.7%) towards personalized screening (e.g., limiting screening to higher-risk women in their 40s) and a preference (27%) for personalized versus “current” screening. Very few (1.5%) patients in the study on personalized screening demonstrated adequate conceptual and numerical knowledge of breast cancer mortality, FPs, and overdiagnosis despite that provision of the information was facilitated by a professional. Another study (*n* = 168) reported that 83% of participants strongly agreed or agreed that the potential benefits outweigh the risks [76].

When examining data on intentions to screen, the one study reporting data across a broader age range found that 98–99% (across two eligible interventions) had positive intentions [95], whereas in three other studies focused on screening in one’s 40s, fewer patients had intentions (e.g., 77% attended screening or planned to schedule a mammogram over the next 6 months [76], 19–31% would not screen during their 40s/would wait until they were 50 [75], mean score of 68 (standard deviation 40) on 0–100 intentions scale [97]). The study examining personalized screening found that intentions were high (92%) for personalized screening [86]. Further, in the Canadian study (*n* = 46) asking participants in focus groups when people should start screening, the authors assessed that 21% of 35- to 49-year-olds chose age 40 [75].

Two studies measuring screening attendance found conflicting results, with attendance at 16 ± 5.4 months of 42% in one US study of clinic patients (having a fairly diverse sample with 36% non-Caucasian) [97] and at an unknown follow-up of 84% in another study that had a large proportion of patients in their 50s [95].

Apart from the study reporting on some preference for personalized screening, three other studies reporting findings by risk group found some inconsistency but at most small differences in preferences. The largest study (*n* = 2918) reported fairly similar screening intentions between groups, with 19–24% (across four different interventions providing numerical data) of those at low risk deciding to wait until their 50s and 24–31% of those at high risk not planning to start or continue screening during their 40s [101]. In another study (*n* = 306), intentions to screen (over the next year) were lower for those at lower risk (63 to 67 on a 0–100 scale) than at higher risk (77 to 87 on a 0–100 scale) [97]. The third study (*n* = 168) reported that there was no association between the predicted breast cancer risk and actual or intended use of screening (77% across all patients) [76].

##### 70 years and older

Four new studies (*N* = 662) [72, 73, 91, 99] were added to the two (*N* = 779) [88, 98] included in the last review. Five studies included recent (within the past 1 or 2 years) screening attendees either 70–71 years of age (1 study; *n* = 734; Australia) or aged 75 years or older (4 studies *N* = 648; all US). Another US study (*n* = 59) included 70- to 92-year-olds, of which 91% had any previous screening. All studies relied on primary care or screening program lists for recruitment, with one also recruiting among community settings. The five studies focusing on decision-making about whether to continue screening in recent screeners used decision aids presenting age-specific information on the benefits and harms of screening, and about life expectancy and competing causes of death for older women [72, 73, 88, 98, 99]. Two studies were rated at high risk of bias [72, 91].

Analyses using what was judged as a moderate-to-low net benefit scenario were separated by the differing focus on age. A low risk of bias RCT (*n* = 734) in Australia compared a decision aid to a standard brochure among 70- to 71-year-old recent screeners [88]. Data on breast cancer and all-cause mortality (each with 2 lives saved per 1000 over 10 years) and harms (135 FPs, 15 overdiagnoses and 9 interval cancers) was included as well as for other outcomes (false negatives, reassurance, cancer from radiation). 95% of participants’ attitudes remained positive towards screening and 86% intended (with 5% more unsure) to continue screening. At 1-month follow-up, 6% had participated in screening and 76% indicated they were in the process of arranging to be screened. We rated the certainty at moderate, for indirectness from the use of intentions and possibly consideration by participants of other outcomes; there were some concerns about reliance on one study but because the study was large with a low risk of bias and an applicable population, we did not rate down further.

One RCT (*n* = 546) [99] and two pre-post trials (*N* = 88) [73, 98] among US primary care clinics measured screening intentions and, in two studies [98, 99], screening attendance after exposure to a decision aid for women aged 75 and older. In all studies, there was the opportunity to use the decision aid during a clinic visit. All of the aids depicted a reduction of breast cancer mortality by 1 per 1000 screened (e.g., 3 vs. 4 die in 1000) but in two the time horizon was 5 years [98, 99] whereas in the other (*n* = 43) [73] it was 10 years. The decision aids in the RCT and one other study also mentioned that 4 women in 1000 screened would avoid a large cancer. Overdiagnosis was described to occur in 11 to 13 per 1000 screened as were FPs in 100–200. The decision aid used in the RCT and one of the pre-post trials also had a statement that “doctors do not know if mammograms benefit women age 75 or older”. In the RCT, screening intentions reduced (by ≥ 1 level on a 15-point scale) for 24.5% of those viewing the decision aid and attendance over 18 months was 51%. No apparent effect modification on receipt of screening was found by patient age, educational level, life expectancy, or breast cancer risk (≥ 3 vs < 3% 5-year risk). A pre-post trial (*n* = 45) [98] by the same investigators found that 56% stated intentions to continue screening (vs. 82% at pre-test) and 63% attended screening by the 15-month follow-up. Both intentions and attendance were impacted by life expectancy in this small study, with those having < 9 years of life expectancy having lower intentions (50% vs. 63%) and attendance (52% vs. 78%). The other pre-post trial presented a slightly lower benefit-to-harm ratio (e.g., 1 life saved over 10 years vs. 200 FPs and 13 overdiagnosis) and only extracted data on intentions based on notes in 18 patient records from documented discussions with a physician; 67% of records had a note for continuing screening, 22% noted a discontinuation, and 22% noted indecision [73]. The applicability of these findings may be specific to previous screeners. There was also concern about imprecision around our estimate of the proportion preferring screening.

## Discussion

This systematic review update on the relative importance placed by patients on the potential benefits and harms of breast cancer screening was conducted to inform an update to the 2018 Canadian Task Force for Preventive Health Care’s guideline on breast cancer screening [11]. The review captured three main types of preferences, directly, through (i) HSUVs to estimate the disutility from health states related to screening (including a screen-detected cancer diagnosis), from different surgical and adjuvant treatments, and by stage of cancer during treatment, and (ii) data from other preference-based studies, such as DCEs, or simple rating scales or trade-offs between specific outcomes, and indirectly through (iii) attitudes, intentions, and/or behaviors towards screening after being informed about the expected benefits and harms (inferred as the relative importance of the potential benefits vs. harms).

### Summary and considerations of health state utility values

After determining an estimate of the utilities of a healthy screen-eligible population (0.94) and four screening states, the estimated disutilities were 0.07 for a positive screening result, before diagnostic work-up (moderate certainty); 0.03–0.04 for a FP requiring imaging and, if necessary, biopsy (low certainty); 0.08 for a true-positive result/untreated screen-detected cancer (moderate certainty); and possibly also 0.08 for an interval cancer (low certainty). These values are likely most applicable to women under 70 years of age. They may also be slightly overestimated based on comparisons between the HSUVs in the healthy screening populations (0.94) we used and the population norms (where not everyone is eligible for screening) reported for a Canadian province (Alberta) using the EQ-5D which were in the range of 0.82–0.83 (±0.15) for people 45–74 years of age [66]. Nevertheless, we chose to rely on the 0.94 estimate because it came from populations comparable to those in the studies reporting on screening states; further, the utility values for some health states examined in our review, such as chemotherapy (0.85, mostly using EQ-5D for measurement), were higher than those estimated for the population norms suggesting that the utility of a healthy screen-eligible population is higher than these population norm estimates.

At 12 months or less from surgery, the disutilities of having a mastectomy (vs. BCS), chemotherapy (vs. none), and radiation therapy (vs. none) were 0.02–0.03, 0.02–0.04, and little-to-none, respectively, though in each case the findings were limited in their applicability to patients receiving adjuvant therapies (for mastectomy vs. BCS), to a mixed surgical population (chemotherapy vs. none) and to many also receiving chemotherapy (radiation vs. none). From data on longer-term health states, there was moderate certainty for little-to-no disutility from mastectomy versus BCS with radiation (most applicable to 5 or more years after surgery) and from radiation, whereas the evidence for chemotherapy, ALND, and treated advanced versus early stage was uncertain.

Disutilities for the treatment states, particularly for receipt of chemotherapy, were lower than we anticipated. Because of this, we added post hoc additional subgroup analyses to the chemotherapy analysis, for radiation exposure and stage of disease, and to the radiation analysis for chemotherapy exposure. These additional analyses revised our estimates slightly for chemotherapy—from the pooled disutility of 0.02 (from the main analysis) to a range between 0.02 and 0.04 (from findings that the disutility may be 0.04 if controlling for radiation exposure and focusing on people with invasive cancers)—though the disutility remained fairly low. There may be some explanations for this. Generally, chemotherapy lasts a short time (i.e., 3–4 months; though can still have late/long-term effects) whereas endocrine therapy can cause low-grade yet persistent side effects over the course of 5–10 years. Endocrine therapy can cause significant physical and cognitive impairments (hot flashes, depression/anxiety, sleep disturbances, weight gain, musculoskeletal pain) which impact significantly women’s lives even if they do not receive chemotherapy. Further, some women with a very low risk of distant recurrence face challenges in deciding whether to take (daily) endocrine therapy. For example, women with very low clinical risk breast cancer can expect a 1–2% absolute survival benefit (at 5–10 years) from 5 years of endocrine therapy and must balance this small benefit with a range of harms/side effects mentioned above. This decision is especially challenging as choosing against endocrine therapy likely means accepting a certain risk of recurrence. The low disutility for mastectomy (relative to BCS) may be in part explained by women having a “false sense of security” believing that having mastectomy (or bilateral mastectomy) will mean that there is no (or limited) risk of recurrence. These women may fail to appreciate that neither surgical approach (nor radiation) decreases the risk of distant recurrence (i.e., metastatic recurrence) which if it occurred would negatively impact survival.

An additional consideration is that the utility-based tools available measure generic HRQoL, which may not adequately capture specific aspects of breast cancer treatment, such as the psychological impact of change in body image, sexuality, and vulnerability that may be captured in disease-specific HRQoL tools. When comparing HSUVs across different health states, including those during screening and treatment, there is the need to rely on similar measures of HSUV to avoid systematic bias due to different measurements rather than true differences in the health states. Across several health states, including a FP, there are possibly important psychosocial symptoms (e.g., anxiety, fear of recurrence) not captured as impacting generic HRQoL; however, these potential harms were not the focus of this review. The evidence in our review mainly came from the use of the EQ-5D instrument as this is commonly used; this tool may provide higher estimates of utilities than would other direct measurement techniques including TTO or SG, though this was difficult to verify since the use of these tools in the included studies was limited and mostly employed public versus patient samples. Further, the relative effects when comparing utilities across health states may be quite similar despite the method used.

When using disutilities to compare the importance of the relevant outcomes, the expected average duration of each health state needs to be considered. For example, while there is probably an important disutility from a positive screening result, if there is no cancer this level of disutility may only persist until the woman receives a diagnostic work-up to rule out the cancer. After this point, the patient would be expected to transition to the state of a FP result (with its associated disutility) for some duration of time that would in many cases not last as long as the health states of a cancer diagnosis and treatment. The relative importance of the outcomes may make use of a ratio of utilities, but standardization of the values to a common timeframe would be necessary (e.g., scaled to per month, one scenario may be that the importance of a treated cancer state lasting on average 24 months [24 × disutility of 0.12–0.14= 2.88–3.26] may be 13–16 times larger than the experiences of someone with a FP for whom there would be the initial positive screening test result and then the awareness of a FP health state, each lasting on average 2 months [(2 × 0.07) + (2 × 0.03–0.04) = 0.20–0.22]). Either health state may be even more important if even a very small remaining disutility lasts for many months or years (such as is the case for endocrine therapy). Another way to use this information is to compare the disutilities to a minimally important difference for the measurement instrument; one estimated MID for the EQ-5D among a general population in Canada is 0.037 [7]. From this perspective, the disutilities of a positive screening result, screen-detected cancer, FP, and surgery with or without adjuvant radiotherapy or chemotherapy all appear to be important, though likely for different and possibly varying durations.

### Summary and considerations of additional data on preferences

From preference-based data where trade-offs between specific outcomes were reported or estimated based on study data, there was moderate certainty that a majority (> 50%) and possibly a large majority (> 75%) of women across all ages probably accept up to six cases of overdiagnosis to prevent one breast-cancer death. There was some indication that almost all would accept up to three overdiagnoses. Similar to several analyses in this review, but perhaps most relevant here, there was concern about findings based on data from knowledge tests within the studies indicating that many women do not completely understand the concept of overdiagnosis despite being provided with explanations. Other evidence was of low certainty but suggested that a large majority of women aged 50–69 years may think that reducing breast cancer mortality (using relatively large rates of breast cancer mortality reduction) is beneficial even if there is no impact on all-cause mortality, and that a majority of women accept that to prevent one breast-cancer death at least a few hundred patients will receive a FP result and at least 10–15 will have a FP resolved through biopsy. Despite this high acceptance, most women of all ages may find it important to have information on the magnitude of risk for the FP outcomes prior to choosing to engage in screening. Further, a large majority of patients may accept that at least 25 people experience a FP, and/or 4 require a biopsy to resolve a FP, to prevent one advanced stage breast cancer.

When using data from studies assessing attitudes, intentions, and screening behaviors, across all age groups’ preferences reduced as the net benefit presented by study authors reduced in magnitude. This was most evident for women in their 40s where the magnitude of benefits dropped substantially from the high (e.g., 1 prevented breast cancer death in 200 screened over 10–30 years, 15–20% relative reduction, or no information on overdiagnosis) to low (i.e., 0.5 fewer deaths [all-cause and from breast-cancer] per 1000 screened over 10 years) net-benefit scenarios. In the low net-benefit scenario, evidence suggested (with low certainty) that a majority (>50%) of women in their 40s may think the harms outweigh the benefits, whereas for women in their 50s a large majority (>75%) may prefer screening. For the women in their 40s, there was an indication in the studies portraying a low net benefit that all-cause mortality would be reduced to a similar extent as breast-cancer mortality. It is unclear how these women would respond if told there may not be a reduction in all-cause mortality. There is some indication from the direct evidence in this review that information about a lack of reduction in all-cause mortality may not change preferences for screening for a large majority of women, yet this evidence came from studies portraying a relatively larger net benefit and including mostly older women. There was moderate certainty that a large majority of women aged 50 and 50–69 years, who have mostly undergone screening, weigh the benefits as greater than the harms from screening in a high net-benefit scenario. Among the five studies of women aged 50, one study [94] mentioning all-cause mortality (indicating no reduction from screening) had similar findings to the others. Further, a large majority of patients 70–71 years of age who have recently screened probably think the benefits outweigh the harms of continuing to screen. A majority of women in their mid-70s to their early 80s may prefer to continue screening; it is unclear what impact life expectancy has on this preference but women were told about other competing causes of death and indicated their preferences in the presence of a physician when there may have been discussions on this topic. The decision aids used in two of these studies, though presenting data on possible reductions in breast-cancer mortality, also had statements about the uncertainty by doctors about whether mammograms benefit women age 75 or older. Many of the women in the studies for those above 50 years had been previously screened, and though some within-study data found no impact of this experience on the preference data there is empirical support that past screening behaviors are independently predictive of future screening intentions and behaviors [10, 115, 116]. A belief perseverance seems to exist for some women, whereby the behavior persists despite being given information that may otherwise contradict their values.

### Comparison with other existing evidence

The findings of this review are fairly similar to those of our previous review. For this update, we added data on HSUVs, formally added GRADE certainty assessments, and attempted to better quantify the relative effects (e.g., trade-offs) and proportion of women each finding related to. The approach to analysis for the non-HSUV data was otherwise quite similar, though we were able to examine more data within subgroups of women in their 50s (i.e., starting to screen at 50 vs. ongoing screening during 50–59/69 years) and their 70s. We also added comparisons between non-mortality benefits (e.g., advanced-stage cancer) and possible harm outcomes. In the group of preference-based studies, we added two large studies (*n* = 1613) [102, 103] for the trade-off between overdiagnosis and breast cancer mortality, and now have more confidence and greater clarity about this data. For the group of studies measuring preferences indirectly, five of six studies related to screening in 40- to 69-year-olds presented data using a high net-benefit scenario; the relevance of this exposure will depend on the appraisal of the clinical evidence of screening effectiveness by the task force or other stakeholders. We also added four new studies [72, 73, 91, 99] to the previous two examining screening in one’s 70s and now have low certainty that at least a majority of women in their mid-70s to their early 80s may prefer to screen assuming a moderate-to-low net benefit (i.e., 1 fewer deaths vs. 100–200 FPs and 11–13 overdiagnoses per 1000) may be attained. Because most of the studies continue to avoid providing any information about estimates of reduction (or possibly lack thereof) in all-cause mortality, there remains some uncertainty about whether and how findings would differ based on different estimates of effects for this outcome.

Several other reviews including utilities in breast cancer exist, each having different eligibility criteria, exposures of interest, and methodological approaches [117–123]. The largest one that also included screening health states had fairly similar findings when comparing exposures of interest for our review [119]. On average across the ranges in utility values in the included studies, this review found that the HSUVs were slightly lower for mastectomy than BCS, and from chemotherapy. Further, their regressions across a wide range of measurement tools, samples (e.g., patients, public, healthcare professionals), and countries found no disutility (measured via coefficients; referent screening) from a noninvasive diagnostic test and quite similar values for surgery with radiation and for chemotherapy. Findings for advanced- versus early-stage cancer showed a small disutility but were not significant. These authors did not conduct study quality assessments or assessment of the certainty of their findings.

### Strengths and limitations of the review

Our review was conducted following current guidance for systematic reviews of patient preferences for guideline developers. We critically appraised all studies; carefully analyzed the data with consideration of the variability across populations, exposures, and outcome measurements; and assessed the certainty about our summary statements. There is a small chance that we missed one or more studies from applying machine learning during screening, though our quality check indicates this would be quite unlikely and our findings across numerous outcomes and comparisons are thought robust in the absence of any additional information if it exists. Our database searches were highly sensitive and peer-reviewed to avoid missing studies as much as possible. All data extractions and risk of bias assessments were verified by senior reviewers with experience in this type of review question. Despite this, our lack of duplicate work, especially for the risk of bias assessments, may have led to a few more errors than expected.

For examining preferences, we focused on empirical studies where women had either experienced the outcomes of interest (with their utility [impact on HRQoL] measured) or had been provided with information on the expected magnitude of effects. It is well documented that uninformed people overestimate the beneficial effects of screening [124] and often do not have any awareness or conceptual understanding of overdiagnosis [125, 126], such as when nonlifesaving early-stage breast cancer detection is not viewed as a harm but rather a benefit when discussed among social networks [127]. It was evident that in some cases the studies we examined had other potential biases, such as responder bias with women with beliefs supporting screening predominating the sample, and we considered this carefully in our assessment of the studies and certainty of the evidence.

We could not make any conclusions on how or whether different descriptions of FPs may impact outcome preferences, such that future studies on this topic would be beneficial. It would be valuable to know whether providing more accurate information during a recall for more testing (e.g., often not a result of a suspicious finding) would reduce the negative impacts (or anticipation of these for those who have not experienced) from a positive screening result and any remaining impacts after their negative cancer status is known (a FP) for at least some women. Few studies presented data on the possibility of lower treatment morbidity and/or avoiding some treatments such as chemotherapy, and it is unknown to what extent preferences for or against screening would change based on this information.

To keep our review manageable and because quantitative findings are considered most informative for our review question, we excluded qualitative findings that could have provided some useful information to help interpret our findings. Authors of one excluded study, with contextual applicability to the task force, of four citizen panels (*n* = 49; 96% aged ≥ 50 years; 89% of women previously screened; low net-benefit scenario presented) in Canada described findings of variability among participants in their future screening intentions and that “all highlighted the value of having this information to make a decision and the importance of individuals being able to make a decision that was best for them” [128].

Our review findings may be most applicable to settings within highly developed countries; further, there were very limited data on whether study findings applied well across an ethnographically and socioeconomically diverse population. Although some data within the studies indicated that findings would be similar across different risk groups, overall there was limited data on which to make conclusions in this regard. Studies measuring relevant HSUVs from Canada were lacking.

## Conclusions

This review examined evidence across a range of data sources on how informed patients value the potential outcomes from breast cancer screening and will be useful during decision-making for recommendations. Depending on judgments about the degree of net benefit screening offers for women within the age groups of interest, there may be differences and variability in preferences to consider. Further, regardless of the strength of any recommendations for offering screening, the evidence strongly suggests that the outcomes examined have importance to women of any age. In addition, easily understandable information about the possible magnitudes of effect across outcomes and that a personal choice is required should be provided to enable informed decision-making.

### Supplementary Information

**Supplementary Material 1.** Search strategies, list of studies excluded at full text, and responses to stakeholder comments.

**Supplementary Material 2.** Data sets for studies reporting health-state utilities.

**Supplementary Material 3.** Data sets for studies not reporting health-state utilities.

## Data Availability

The data generated during this study are available within the manuscript or its supplementary files.

## Abbreviation

BCS: Breast-conserving surgery
EQ-5D: EuroQol-5 dimension
FP: False positive
GRADE: Grading of Recommendations Assessment, Development and Evaluation
HRQOL: Health-related quality of life
HSUV: Health-state utility values
PHAC: Public Health Agency of Canada
SG: Standard gamble
TTO: Time-trade-off
VAS: Visual analogue scale

## References

[1] Schünemann HJ, Wiercioch W, Etxeandia I, Falavigna M, Santesso N, Mustafa R (2014). Guidelines 2.0: systematic development of a comprehensive checklist for a successful guideline enterprise. CMAJ.

[2] Zhang Y, Coello PA, Brożek J, Wiercioch W, Etxeandia-Ikobaltzeta I, Akl EA (2017). Using patient values and preferences to inform the importance of health outcomes in practice guideline development following the GRADE approach. Health Qual Life Outcomes.

[3] Bastemeijer CM, Voogt L, van Ewijk JP, Hazelzet JA (2017). What do patient values and preferences mean? A taxonomy based on a systematic review of qualitative papers. Patient Educ Couns.

[4] Zhang Y, Alonso-Coello P, Guyatt GH, Yepes-Nuñez JJ, Akl EA, Hazlewood G (2019). GRADE Guidelines: 19. Assessing the certainty of evidence in the importance of outcomes or values and preferences-Risk of bias and indirectness. J Clin Epidemiol..

[5] Zhang Y, Coello PA, Guyatt GH, Yepes-Nuñez JJ, Akl EA, Hazlewood G (2019). GRADE guidelines: 20. Assessing the certainty of evidence in the importance of outcomes or values and preferences-inconsistency, imprecision, and other domains. J Clin Epidemiol.

[6] Torrance GW, Feeny D (1989). Utilities and quality-adjusted life years. Int J Technol Assess Health Care.

[7] McClure NS, Sayah FA, Xie F, Luo N, Johnson JA (2017). Instrument-defined estimates of the minimally important difference for EQ-5D-5L index scores. Value Health.

[8] Marteau TM, Dormandy E, Michie S (2001). A measure of informed choice. Health Expect.

[9] Ajzen I (1991). The theory of planned behavior. Organ Behav Hum Decis Process.

[10] Griva F, Anagnostopoulos F, Madoglou S (2009). Mammography screening and the theory of planned behavior: suggestions toward an extended model of prediction. Women Health.

[11] Klarenbach S, Sims-Jones N, Lewin G, Singh H, Thériault G, Tonelli M (2018). Recommendations on screening for breast cancer in women aged 40–74 years who are not at increased risk for breast cancer. CMAJ.

[12] Canadian Task Force on Preventive Health Care. Canadian Task Force on Preventive Health Care Procedure Manual. 2022. https://canadiantaskforce.ca/methods/.

[13] Pillay J, MacGregor T, Featherstone R, Hartling L. Breast Cancer Screening: Part B. Systematic Review on Women’s Values and Preferences to Inform an Update of the Canadian Task Force on Preventive Health Care 2011 Guideline. 2018. https://canadiantaskforce.ca/wp-content/uploads/2018/11/Womens-Values-and-Preferences-on-Breast-Cancer-Screening_FINAL.pdf.

[14] Page MJ, McKenzie JE, Bossuyt PM, Boutron I, Hoffmann TC, Mulrow CD (2021). The PRISMA 2020 statement: an updated guideline for reporting systematic reviews. BMJ.

[15] United Nation’s Development Programme. Human Development Index. Human Development Reports United Nations. 2022. https://hdr.undp.org/content/human-development-report-2021-22.

[16] Olsen O, Gøtzsche PC (2001). Cochrane review on screening for breast cancer with mammography. Lancet.

[17] Gøtzsche PC, Olsen O (2000). Is screening for breast cancer with mammography justifiable?. Lancet.

[18] Distiller Inc. AI Screening. Ottawa, Canada: Evidence Partners. 2022. http://v2dis-help.evidencepartners.com/1/en/topic/ai-preview-and-rank.

[19] Burns JK, Etherington C, Cheng-Boivin O, Boet S. Using an artificial intelligence tool can be as accurate as human assessors in level one screening for a systematic review. Health Info Libr J. 2021;00:1–13. 10.1111/hir.12413. 10.1111/hir.12413 34792285

[20] Hamel C, Kelly SE, Thavorn K, Rice DB, Wells GA, Hutton B (2020). An evaluation of DistillerSR’s machine learning-based prioritization tool for title/abstract screening – impact on reviewer-relevant outcomes. BMC Med Res Methodol.

[21] Higgins JPT LT, Deeks JJ (editors). Chapter 6: Choosing effect measures and computing estimates of effect. In: Cochrane Handbook for Systematic Reviews of Interventions version 64 (updated August 2023). Edited by Higgins JPT TJ, Chandler J, Cumpston M, Li T, Page MJ, Welch VA. Cochrane; 203.

[22] Barratt A, Howard K, Irwig L, Salkeld G, Houssami N (2005). Model of outcomes of screening mammography: information to support informed choices. BMJ.

[23] Marmot MG, Altman DG, Cameron DA, Dewar JA, Thompson SG, Wilcox M (2013). The benefits and harms of breast cancer screening: an independent review. Br J Cancer.

[24] Murad MH, Mustafa RA, Schünemann HJ, Sultan S, Santesso N (2017). Rating the certainty in evidence in the absence of a single estimate of effect. Evid Based Med.

[25] Santesso N, Glenton C, Dahm P, Garner P, Akl EA, Alper B (2020). GRADE guidelines 26: informative statements to communicate the findings of systematic reviews of interventions. J Clin Epidemiol.

[26] Petrova D, Garcia-Retamero R, Cokely ET (2015). Understanding the harms and benefits of cancer screening: a model of factors that shape informed decision making. Med Decis Making.

[27] Ali AA, Xiao H, Tawk R, Campbell E, Semykina A, Montero AJ (2017). Comparison of health utility weights among elderly patients receiving breast-conserving surgery plus hormonal therapy with or without radiotherapy. Curr Med Res Opin.

[28] Bonomi AE, Boudreau DM, Fishman PA, Ludman E, Mohelnitzky A, Cannon EA (2008). Quality of life valuations of mammography screening. Qual Life Res.

[29] Bromley HL, Mann GB, Petrie D, Nickson C, Rea D, Roberts TE (2019). Valuing preferences for treating screen detected ductal carcinoma in situ. Eur J Cancer.

[30] Criscitiello C, Spurden D, Piercy J, Rider A, Williams R, Mitra D (2021). Health-related quality of life among patients with HR+/HER2- early breast cancer. Clin Ther.

[31] de Kok M, Dirksen CD, Kessels AG, van der Weijden T, van de Velde CJ, Roukema JA (2010). Cost-effectiveness of a short stay admission programme for breast cancer surgery. Acta Oncol.

[32] Domeyer PJ, Sergentanis TN, Zagouri F, Zografos GC (2010). Health-related quality of life in vacuum-assisted breast biopsy: short-term effects, long-term effects and predictors. Health Qual Life Outcomes.

[33] Fujii T, Shibata Y, Akane A, Aoki W, Sekiguchi A, Takahashi K (2019). A randomised controlled trial of pectoral nerve-2 (PECS 2) block vs. serratus plane block for chronic pain after mastectomy. Anaesthesia.

[34] Gordon LG, DiSipio T, Battistutta D, Yates P, Bashford J, Pyke C (2017). Cost-effectiveness of a pragmatic exercise intervention for women with breast cancer: results from a randomized controlled trial. Psychooncology.

[35] Haidari RE, Anota A, Dabakuyo-Yonli TS, Guillemin F, Conroy T, Velten M (2022). Utility values and its time to deterioration in breast cancer patients after diagnosis and during treatments. Qual Life Res.

[36] Hall PS, Hamilton P, Hulme CT, Meads DM, Jones H, Newsham A (2015). Costs of cancer care for use in economic evaluation: a UK analysis of patient-level routine health system data. Br J Cancer.

[37] Hanson SE, Lei X, Roubaud MS, DeSnyder SM, Caudle AS, Shaitelman SF (2022). Long-term quality of life in patients with breast cancer after breast conservation vs mastectomy and reconstruction. JAMA Surg.

[38] Kim SH, Jo MW, Lee JW, Lee HJ, Kim JK (2015). Validity and reliability of EQ-5D-3L for breast cancer patients in Korea. Health Qual Life Outcomes..

[39] Knuttel FM, van den Bosch MAAJ, Young-Afat DA, Emaus MJ, van den Bongard DHJG, Witkamp AJ, Verkooijen HM (2017). Patient preferences for minimally invasive and open locoregional treatment for early-stage breast cancer. Value Health.

[40] Kouwenberg CAE, de Ligt KM, Kranenburg LW, Rakhorst H, de Leeuw D, Siesling S, Busschbach JJ, Mureau MAM (2020). Long-term health-related qQuality of life after four common surgical treatment options for breast cancer and the effect of complications: a retrospective patient-reported survey among 1871 patients. Plast Reconstr Surg.

[41] Lagendijk M, van Egdom LSE, van Veen FEE, Vos EL, Mureau MAM, van Leeuwen N (2018). Patient-reported outcome measures may add value in breast cancer surgery. Ann Surg Onco.

[42] Lagendijk M, Vos EL, Nieboer D, Verhoef C, Corten EML, Koppert LB (2018). Evaluation of cosmetic outcome following breast-conserving therapy in trials: panel versus digitalized analysis and the role of PROMs. Breast J.

[43] May AM, Bosch MJC, Velthuis MJ, van der Wall E, Steins Bisschop CN, Los M (2017). Cost- effectiveness analysis of an 18-week exercise programme for patients with breast and colon cancer undergoing adjuvant chemotherapy: the randomised PACT study. BMJ Open.

[44] Min YH, Lee JW, Shin YW, Jo MW, Sohn G, Lee JH (2014). Daily collection of self-reporting sleep disturbance data via a smartphone app in breast cancer patients receiving chemotherapy: a feasibility study. J Med Internet Res.

[45] Miret C, Orive M, Sala M, Garcia-Gutierrez S, Sarasqueta C, Legarreta MJ (2023). Reference values of EORTC QLQ-C30, EORTC QLQ-BR23, and EQ-5D-5L for women with non- metastatic breast cancer at diagnosis and 2 years after. Qual Life Res.

[46] Morgan JL, Shrestha A, Reed MWR, Herbert E, Bradburn M, Walters SJ (2021). Bridging the age gap in breast cancer: impact of omission of breast cancer surgery in older women with oestrogen receptor-positive early breast cancer on quality-of-life outcomes. Br J Surg.

[47] Moro-Valdezate D, Buch-Villa E, Peiró S, Morales-Monsalve MD, Caballero-Gárate A, Martínez-Agulló Á (2014). Factors associated with health-related quality of life in a cohort of Spanish breast cancer patients. Breast Cancer.

[48] Moshina N, Falk RS, Botteri E, Larsen M, Akslen LA, Cairns JA (2022). Quality of life among women with symptomatic, screen-detected, and interval breast cancer, and for women without breast cancer: a retrospective cross-sectional study from Norway. Qual Life Res.

[49] Park HY, Nam KE, Lim JY, Yeo SM, Lee JI, Hwang JH (2023). Real-time interactive digital health care system for postoperative breast cancer patients: a randomized controlled trial. Telemed J E Health.

[50] Porciello G, Montagnese C, Crispo A, Grimaldi M, Libra M, Vitale S (2020). Mediterranean diet and quality of life in women treated for breast cancer: a baseline analysis of DEDiCa multicentre trial. PloS One.

[51] Rautalin M, Färkkilä N, Sintonen H, Saarto T, Taari K, Jahkola T (2018). Health-related quality of life in different states of breast cancer - comparing different instruments. Acta Oncol.

[52] Rautalin M, Jahkola T, Roine RP (2021). Surgery and health-related quality of life - a prospective follow up study on breast cancer patients in Finland. Eur J Surg Oncol.

[53] Ring A, Battisti NML, Reed MWR, Herbert E, Morgan JL, Bradburn M (2021). Bridging The Age Gap: observational cohort study of effects of chemotherapy and trastuzumab on recurrence, survival and quality of life in older women with early breast cancer. Br J Cancer.

[54] Roine E, Sintonen H, Kellokumpu-Lehtinen PL, Penttinen H, Utriainen M, Vehmanen L (2020). Health-related quality of life of breast cancer survivors attending an exercise intervention study: a five-year follow-up. In Vivo.

[55] Schleinitz MD, DePalo D, Blume J, Stein M (2006). Can differences in breast cancer utilities explain disparities in breast cancer care?. J Gen Intern Med.

[56] Songtish D, Praditsitthikorn N, Teerawattananon Y (2014). A cost-utility analysis comparing standard axillary lymph node dissection with sentinel lymph node biopsy in patients with early stage breast cancer in thailand. Value Health Reg Issues.

[57] Swanick CW, Lei X, Xu Y, Shen Y, Goodwin NA, Smith GL (2018). Long-term patient- reported outcomes in older breast cancer survivors: a population-based survey study. Int J Radiat Oncol Biol Phys.

[58] Tanaka K, Tachi T, Hori A, Osawa T, Nagaya K, Makino T (2019). Cost utility analysis of pharmacist counseling care for breast cancer chemotherapy outpatients. Pharmazie.

[59] Timmers JM, Damen JA, Pijnappel RM, Verbeek AL, den Heeten GJ, Adang EM (2014). Cost- effectiveness of non-invasive assessment in the Dutch breast cancer screening program versus usual care: a randomized controlled trial. Can J Public Health.

[60] Tosteson AN, Fryback DG, Hammond CS, Hanna LG, Grove MR, Brown M (2014). Consequences of false-positive screening mammograms. JAMA Intern Med.

[61] Tran TXM, Jung SY, Lee EG, Cho H, Cho J, Lee E (2023). Long-term trajectory of postoperative health-related quality of life in young breast cancer patients: a 15-year follow- up study. J Cancer Surviv.

[62] Velikova G, Williams LJ, Willis S, Dixon JM, Loncaster J, Hatton M (2018). Quality of life after postmastectomy radiotherapy in patients with intermediate-risk breast cancer (SUPREMO): 2-year follow-up results of a randomised controlled trial. Lancet Oncol.

[63] Youens D, Halkett G, Wright C, O'Connor M, Schofield P, Jefford M (2019). Assessing the cost-effectiveness of RT Prepare: a radiation therapist-delivered intervention for reducing psychological distress prior to radiotherapy. Psychooncology.

[64] Žigman T, Lukša I, Mihaljević G, Žarković M, Kirac I, Vrdoljak DV (2020). Defining health- related quality of life in localized and advanced stages of breast cancer - the first step towards hereditary cancer genetic counseling. Acta Clin Croat.

[65] Roine E, Sintonen H, Kellokumpu-Lehtinen PL, Penttinen H, Utriainen M, Vehmanen L (2021). Long-term health-related quality of life of breast cancer survivors remains impaired compared to the age-matched general population especially in young women. Results from the prospective controlled BREX exercise study. Breast.

[66] Alberta PROMS and EQ-5D Research and Support Unit. Alberta Population Norms for EQ- 5D-5L. 2018. Available at: https://sites.google.com/ualberta.ca/apersu/about-eq-5d/eq-5d-population-norms.

[67] Baena-Cañada JM, Luque-Ribelles V, Quilez-Cutillas A, Rosado-Varela P, Benitez- Rodriguez E, Marquez-Calderon S (2018). How a deliberative approach includes women in the decisions of screening mammography: a citizens' jury feasibility study in Andalusia, Spain. BMJ Open.

[68] Baena-Cañada JM, Rosado-Varela P, Expósito-Álvarez I, González-Guerrero M, Nieto-Vera J, Benítez-Rodríguez E (2015). Using an informed consent in mammography screening: a randomized trial. Cancer Med.

[69] Berens EM, Reder M, Razum O, Kolip P, Spallek J (2015). Informed choice in the german mammography screening program by education and migrant status: survey among first-time invitees. PLoS One.

[70] Bilger M, Özdemir S, Finkelstein EA (2020). Demand for cancer screening services: results from randomized controlled discrete choice experiments. Value Health.

[71] Bourmaud A, Soler-Michel P, Oriol M, Regnier V, Tinquaut F, Nourissat A (2016). Decision aid on breast cancer screening reduces attendance rate: results of a large-scale, randomized, controlled study by the DECIDEO group. Oncotarget.

[72] Braithwaite D, Chicaiza A, Lopez K, Lin KW, Mishori R, Karanth SD, et al. Clinician and patient perspectives on screening mammography among women age 75 and older: a pilot study of a novel decision aid. PEC Innov. 2023. 10.1016/j.pecinn.2023.100132 PMC10136373 37124453

[73] Cadet T, Aliberti G, Karamourtopoulos M, Jacobson A, Gilliam EA, Primeau S (2021). Evaluation of a mammography decision aid for women 75 and older at risk for lower health literacy in a pretest-posttest trial. Patient Educ Couns.

[74] Davey C, White V, Gattellari M, Ward JE (2005). Reconciling population benefits and women's individual autonomy in mammographic screening: in-depth interviews to explore women's views about 'informed choice'. Aust N Z J Public Health.

[75] Driedger SM, Annable G, Brouwers M, Turner D, Maier R (2017). Can you un-ring the bell? A qualitative study of how affect influences cancer screening decisions. BMC Cancer.

[76] Elkin EB, Pocus VH, Mushlin AI, Cigler T, Atoria CL, Polaneczky MM (2017). Facilitating informed decisions about breast cancer screening: development and evaluation of a web- based decision aid for women in their 40s. BMC Med Inform Decis Mak.

[77] Ganott MA, Sumkin JH, King JL, Klym AH, Catullo VJ, Cohen CS, Gur D (2006). Screening mammography: do women prefer a higher recall rate given the possibility of earlier detection of cancer?. Radiology.

[78] Gummersbach E, in der Schmitten J, Mortsiefer A, Abholz HH, Wegscheider K, Pentzek M. Willingness to participate in mammography screening: a randomized controlled questionnaire study of responses to two patient information leaflets with different factual content. Dtsch Arztebl Int. 2015;112(5):61-8. 10.3238/arztebl.2015.0061 PMC4335580 25686383

[79] Gyrd-Hansen D (2000). Cost-benefit analysis of mammography screening in Denmark based on discrete ranking data. Int J Technol Assess Health Care.

[80] Haakenson CP, Vickers KS, Cha SS, Vachon CM, Thielen JM, Kircher KJ (2006). Efficacy of a simple, low-cost educational intervention in improving knowledge about risks and benefits of screening mammography. Mayo Clin Proc.

[81] Henriksen MJ, Guassora AD, Brodersen J (2015). Preconceptions influence women's perceptions of information on breast cancer screening: a qualitative study. BMC Res Notes.

[82] Hersch J, Barratt A, Jansen J, Irwig L, McGeechan K, Jacklyn G (2015). Use of a decision aid including information on overdetection to support informed choice about breast cancer screening: a randomised controlled trial. Lancet.

[83] Hersch J, Jansen J, Barratt A, Irwig L, Houssami N, Howard K (2013). Women's views on overdiagnosis in breast cancer screening: a qualitative study. BMJ.

[84] Jafri NF, Ayyala RS, Ozonoff A, Jordan-Gray J, Slanetz PJ (2008). Screening mammography: does ethnicity influence patient preferences for higher recall rates given the potential for earlier detection of breast cancer?. Radiology.

[85] Lawrence VA, Streiner D, Hazuda HP, Naylor R, Levine M, Gafni A (2000). A cross-cultural consumer-based decision aid for screening mammography. Prev Med.

[86] Laza-Vasquez C, Martinez-Alonso M, Forne-Izquierdo C, Vilaplana-Mayoral J, Cruz-Esteve I, Sanchez-Lopez I (2022). Feasibility and accceptability of personalized breast cancer screening (DECIDO Study): a single-arm proof-of-concept trial. Int J Environ Res Public Health.

[87] Lewis CL, Pignone MP, Sheridan SL, Downs SM, Kinsinger LS (2003). A randomized trial of three videos that differ in the framing of information about mammography in women 40 to 49 years old. J Gen Intern Med.

[88] Mathieu E, Barratt A, Davey HM, McGeechan K, Howard K, Houssami N (2007). Informed choice in mammography screening: a randomized trial of a decision aid for 70-year-old women. Arch Intern Med.

[89] Mathieu E, Barratt AL, McGeechan K, Davey HM, Howard K, Houssami N (2010). Helping women make choices about mammography screening: an online randomized trial of a decision aid for 40-year-old women. Patient Educ Couns.

[90] Nekhlyudov L, Li R, Fletcher SW (2008). Informed decision making before initiating screening mammography: does it occur and does it make a difference?. Health Expect.

[91] Pappadis MR, Volk RJ, Krishnan S, Weller SC, Jaramillo E, Hoover DS (2018). Perceptions of overdetection of breast cancer among women 70 years of age and older in the USA: a mixed- methods analysis. BMJ Open.

[92] Paul C, Nicholls R, Priest P, McGee R (2008). Making policy decisions about population screening for breast cancer: the role of citizens' deliberation. Health Policy.

[93] Perez-Lacasta MJ, Martinez-Alonso M, Garcia M, Sala M, Perestelo-Perez L, Vidal C (2019). Effect of information about the benefits and harms of mammography on women's decision making: the InforMa randomised controlled trial. PloS One.

[94] Reder M, Kolip P (2017). Does a decision aid improve informed choice in mammography screening? Results from a randomised controlled trial. PloS One.

[95] Roberto A, Colombo C, Candiani G, Satolli R, Giordano L, Jaramillo L (2020). A dynamic web-based decision aid to improve informed choice in organised breast cancer screening. A pragmatic randomised trial in Italy. Br J Cancer.

[96] Saver BG, Mazor KM, Luckmann R, Cutrona SL, Hayes M, Gorodetsky T (2017). Persuasive interventions for controversial cancer screening recommendations: testing a novel approach to help patients make evidence-based decisions. Ann Fam Med.

[97] Schonberg MA, Davis RB, Karamourtopoulos MC, Pinheiro A, Sternberg SB, Jacobson AR (2020). A pre-test-post-test trial of a breast cancer risk report for women in their 40s. Am J Prev Med.

[98] Schonberg MA, Hamel MB, Davis RB, Griggs MC, Wee CC, Fagerlin A (2014). Development and evaluation of a decision aid on mammography screening for women 75 years and older. JAMA Intern Med.

[99] Schonberg MA, Kistler CE, Pinheiro A, Jacobson AR, Aliberti GM, Karamourtopoulos M (2020). Effect of a mammography screening decision aid for women 75 years and older: a cluster randomized clinical trial. JAMA Intern Med.

[100] Schwartz LM, Woloshin S, Sox HC, Fischhoff B, Welch HG (2000). US women's attitudes to false- positive mammography results and detection of ductal carcinoma in situ: cross-sectional survey. West J Med.

[101] Seitz HH, Gibson L, Skubisz C, Forquer H, Mello S, Schapira MM (2016). Effects of a risk- based online mammography intervention on accuracy of perceived risk and mammography intentions. Patient Educ Couns.

[102] Sicsic J, Pelletier-Fleury N, Moumjid N (2018). Women's benefits and harms trade-offs in breast cancer screening: results from a discrete-choice experiment. Value Health.

[103] Stiggelbout A, Copp T, Jacklyn G, Jansen J, Liefers GJ, McCaffery K, Hersch J (2020). Women's acceptance of overdetection in breast cancer screening: can we assess harm-benefit tradeoffs?. Med Decis Making.

[104] Toledo-Chávarri A, Rué M, Codern-Bové N, Carles-Lavila M, Perestelo-Pérez L, Pérez- Lacasta MJ, et al. A qualitative study on a decision aid for breast cancer screening: views from women and health professionals. Eur J Cancer Care (Engl). 2017;26(3):e12660. 10.1111/ecc.12660 28145105

[105] Valentine KD, Wegier P, Shaffer VA, Scherer LD (2022). The impact of 4 risk communication interventions on cancer screening preferences and knowledge. Med Decis Mak.

[106] Van den Bruel A, Jones C, Yang Y, Oke J, Hewitson P (2015). People's willingness to accept overdetection in cancer screening: population survey. BMJ.

[107] Waller J, Douglas E, Whitaker KL, Wardle J (2013). Women’s responses to information about overdiagnosis in the UK breast cancer screening programme: a qualitative study. BMJ Open.

[108] Waller J, Whitaker KL, Winstanley K, Power E, Wardle J (2014). A survey study of women's responses to information about overdiagnosis in breast cancer screening in Britain. Br J Cancer.

[109] Wong IO, Lam WW, Wong CN, Cowling BJ, Leung GM, Fielding R (2015). Towards informed decisions on breast cancer screening: development and pilot testing of a decision aid for Chinese women. Patient Educ Couns.

[110] Yasunaga H, Ide H, Imamura T, Ohe K (2007). Women's anxieties caused by false positives in mammography screening: a contingent valuation survey. Breast Cancer Res Treat.

[111] Cadet T, Pinheiro A, Karamourtopoulos M, Jacobson AR, Aliberti GM, Kistler CE (2021). Effects by educational attainment of a mammography screening patient decision aid for women aged 75 years and older. Cancer.

[112] Hersch J, Barratt A, McGeechan K, Jansen J, Houssami N, Dhillon H (2021). Informing women about overdetection in breast cancer screening: two-year outcomes from a randomized trial. J Natl Cancer Inst.

[113] Hersch J, McGeechan K, Barratt A, Jansen J, Irwig L, Jacklyn G (2017). How information about overdetection changes breast cancer screening decisions: a mediation analysis within a randomised controlled trial. BMJ Open.

[114] López-Panisello MB, Pérez-Lacasta MJ, Rué M, Carles-Lavila M (2023). Factors influencing intention to participate in breast cancer screening An exploratory structural model. PloS One.

[115] Norman P, Cooper Y (2011). The theory of planned behaviour and breast self-examination: assessing the impact of past behaviour, context stability and habit strength. Psychol Health.

[116] Rutter DR. Attendance and reattendance for breast cancer screening: a prospective 3-year test of the Theory of Planned Behaviour. Br J Health Psychol. 2000;5(Part 1):1-13.

[117] Bromley HL, Petrie D, Mann GB, Nickson C, Rea D, Roberts TE (2019). Valuing the health states associated with breast cancer screening programmes: a systematic review of economic measures. Soc Sci Med.

[118] Gong JR, Han J, Lee D, Bae S (2020). A meta-regression analysis of utility weights for breast cancer: the power of patients' experience. Int J Environ Res Public Health.

[119] Kaur MN, Yan J, Klassen AF, David JP, Pieris D, Sharma M (2022). A systematic literature review of health utility values in breast cancer. Med Decis Making.

[120] Li L, Severens JLH, Mandrik O (2019). Disutility associated with cancer screening programs: a systematic review. PloS One.

[121] Pourrahmat MM, Kim A, Kansal AR, Hux M, Pushkarna D, Fazeli MS (2021). Health state utility values by cancer stage: a systematic literature review. Eur J Health Econ.

[122] Wang Y, Gavan SP, Steinke D, Cheung KL, Chen LC (2022). The impact of age on health utility values for older women with early-stage breast cancer: a systematic review and meta-regression. Health Qual Life Outcomes.

[123] Yoon AY, Bozzuto L, Seto AJ, Fisher CS, Chatterjee A (2019). A systematic review of utility score assessments in the breast surgery cost-analysis literature. Ann Surg Oncol.

[124] Hoffmann TC, Del Mar C (2015). Patients’ expectations of the benefits and harms of treatments, screening, and tests: a systematic review. JAMA Internal Med.

[125] Ghanouni A, Meisel SF, Renzi C, Wardle J, Waller J (2016). Survey of public definitions of the term 'overdiagnosis' in the UK. BMJ Open.

[126] Moynihan R, Nickel B, Hersch J, Beller E, Doust J, Compton S, Barratt A, Bero L, McCaffery K (2015). Public opinions about overdiagnosis: a national community survey. PLoS One.

[127] Nowak SA, Parker AM (2014). Social network effects of nonlifesaving early-stage breast cancer detection on mammography rates. Am J Public Health.

[128] Abelson J, Tripp L, Sussman J (2018). 'I just want to be able to make a choice': results from citizen deliberations about mammography screening in Ontario Canada. Health Policy.

