# Supplementary material for: Patient preferences for breast cancer screening: a systematic review update to inform recommendations by the Canadian Task Force on Preventive Health Care
Source: Syst Rev. 2024 May 28;13:140. doi: 10.1186/s13643-024-02539-8 (PMC11134964; doi:10.1186/s13643-024-02539-8)
Supplement: Supplementary file 1 — Supplementary Material 1. Search strategies, list of studies excluded at full text, and responses to stakeholder comments. [file 13643_2024_2539_MOESM1_ESM.docx]

**Patient preferences for breast cancer screening: a systematic review update** **to inform recommendations by the Canadian Task Force on Preventive Health Care**

Jennifer Pillay, Samantha Guitard, Sholeh Rahman, Sabrina Saba, Ashiqur Rahman, Liza Bialy, Nicole Gehring, Maria Tan, Alex Melton, Lisa Hartling; Alberta Research Centre for Health Evidence, Faculty of Medicine and Dentistry, University of Alberta, Edmonton, Alberta, Canada.

**Supplementary file 1. Search strategies, list of studies excluded at full text, responses to stakeholder comments**

**CONTENTS**

**Database and grey literature search strategies p. 2**

**List of excluded studies at full text p. 28**

**Responses to stakeholder comments p.55**

**Database Search Strategies**

**MEDLINE**

Database(s): Ovid MEDLINE(R) ALL 1946

Search date: 19Jun2023

| **#** | **Searches** |
| --- | --- |
| 1 | exp Breast Neoplasms/ |
| 2 | exp Carcinoma, Intraductal, Noninfiltrating/ |
| 3 | ((adenocarcinoma* or adenoma* or cancer* or carcin* or malignan* or metasta*or neoplas* or sarcoma* or tumour* or tumor*) adj3 (breast? or mamma or mammar*)).ti,ab,kf. |
| 4 | (DCIS or (ductal carcinoma adj1 (in situ or insitu))).ti,ab,kf. |
| 5 | ((intra-ductal or intraductal) adj1 carcinoma*).ti,ab,kf. |
| 6 | ((mammogram* or mammograph*) and screening).ti. or ((mammogram* or mammograph*) and screening).ab. /freq=2 |
| 7 | or/1-6 [BREAST CA] |
| 8 | exp *Breast Neoplasms/di and screen*.mp. |
| 9 | exp Early Diagnosis/ and screen*.mp. |
| 10 | exp Magnetic Resonance Imaging/ and screen*.mp. |
| 11 | exp Mammography/ |
| 12 | exp Mass Screening/ |
| 13 | Ultrasonography, Mammary/ and screen*.mp. |
| 14 | (echograph* or echo-mammogra* or echo-tomograph* or echomammogra* or echotomograph*).ti,ab,kf. |
| 15 | (magnetic resonance imag* or magnetic resonance tomograph* or MR tomograph* or MRI or MRIs or NMRI or NMRIs).tw,kf. and screen*.mp. |
| 16 | "breast tomosynthesis".ti,ab,kf. and screen*.mp. |
| 17 | screen*.ti. |
| 18 | screen*.ab. /freq=2 |
| 19 | (sonograph* or ultra-son* or ultra-sound* or ultrason* or ultrasound*).ti,ab,kf. and screen*.mp. |
| 20 | or/8-19 [SCREENING] |
| 21 | 7 and 20 [BREAST CANCER + SCREENING] |
| 22 | attitude to health/ |
| 23 | Choice Behavior/ |
| 24 | Consumer Behavior/ |
| 25 | Decision Making/ |
| 26 | Decision Making, Shared/ |
| 27 | decision support techniques/ |
| 28 | health knowledge, attitudes, practice/ |
| 29 | "patient acceptance of health care"/ |
| 30 | patient participation/ |
| 31 | patient preference/ |
| 32 | patient satisfaction/ |
| 33 | Perception/ |
| 34 | ((acceptabilit* or acceptance or acceptable or attitude* or belief* or deliberat* or expectation* or intent* or jury or perceiv* or perception* or perspective* or prefer* or valuation* or valued or values or views or willing*) adj3 (elicit* or citizen or citizens or client* or consumer* or female* or individual* or panel or participant* or patient* or people* or person* or public* or respondent* or screenee* or stated or user* or wom#n)).ti,ab,kf. |
| 35 | ((choice* or choos*) adj2 (behavio?r* or discrete or experiment*)).ti,ab,kf. |
| 36 | (conjoint adj3 (analy* or design* or evaluation or exercise* or experiment* or studies or study or survey* or task* or valuation or value* or valuing)).ti,ab,kf. |
| 37 | (contingent adj3 (analy* or design* or evaluation or valuation or value* or valuing)).ti,ab,kf. |
| 38 | (decision* adj (board$1 or certain* or conflict* or dissatisf* or satisf* or uncertain*)).ti,ab,kf. |
| 39 | (decision* adj2 (aid$1 or mak* or needs or support* or tool*)).ti,ab,kf. |
| 40 | (decision* adj5 balance).ti,ab,kf. |
| 41 | (ranking or rating).ti,ab,kf. |
| 42 | ((mammograph* or mammogram* or screen*) adj3 (acceptabilit*or acceptance or acceptable or agree* or attitude* or behavio?r* or belief* or choice* or choos* or decid* or decision* or declin* or expectation* or inclin* or intending or intent* or perceiv* or perception* or perspective* or prefer*or propensity or pursue or values or views or willing*)).ti,ab,kf. |
| 43 | (tradeoff* or trade-off*).ti,ab,kf. |
| 44 | or/22-43 [DECISION-MAKING] |
| 45 | 21 and 44 [BREAST CA SCREENING + DECISION-MAKING] |
| 46 | *Genes, BRCA1/ or (BRCA* or gene* or hereditary).ti. |
| 47 | 45 not 46 [BREAST CA SCREENING + DECISION-MAKING - Genetic high-risk patient studies excluded] |
| 48 | exp Breast Neoplasms/ |
| 49 | exp Carcinoma, Intraductal, Noninfiltrating/ |
| 50 | ((adenocarcinoma* or adenoma* or cancer* or carcin* or malignan* or metasta*or neoplas* or sarcoma* or tumour* or tumor*) adj3 (breast? or mamma or mammar*)).ti,ab,kf. |
| 51 | (DCIS or (ductal carcinoma adj1 (in situ or insitu))).ti,ab,kf. |
| 52 | ((intra-ductal or intraductal) adj1 carcinoma*).ti,ab,kf. |
| 53 | or/48-52 [BREAST CA] |
| 54 | Diagnostic Errors/ |
| 55 | False Positive Reactions/ |
| 56 | (false*-positiv* or overdiagnosis or (diagnos* adj3 error*)).ti,ab,kf. |
| 57 | (indetermina* or suspect* or suspicious*).ti,ab,kf. |
| 58 | ("Ductal carcinoma in situ" or DCIS).ti,ab,kf. |
| 59 | or/54-58 |
| 60 | (Breast or ductal or mammogra*).hw,ti,ab,kf. |
| 61 | 59 and 60 [Non-CA] |
| 62 | 53 or 61 [BREAST CA /NOT BREAST CA] |
| 63 | Quality-Adjusted Life Years/ |
| 64 | *"Quality of Life"/ |
| 65 | "Value of Life"/ |
| 66 | (QALY or QALYS).ti,ab,kf. |
| 67 | direct*-elicitation*.ti,ab,kf. |
| 68 | (quality adj adjusted-life).ti,ab,kf. |
| 69 | ("Utility Based Questionnaire-Cancer" or UBQC or UBQC or UBQ-C or "15-Dimension questionnaire" or 15D or "Assessment of QoL" or AQoL or AQoL2 or "wellbeing index" or "well-being index" or "quality of wellbeing" or "quality of well-being" or qwb).ti,ab,kf. |
| 70 | (EuroQol5D or EuroQol or Euro-Qol or EuroQual5D or EuroQual or Euro-Qual or EQ5D or EQ-5D).ti,ab,kf. |
| 71 | (health adj2 (utilit* or disutilit*)).ti,ab,kf. |
| 72 | (health-stat$2 adj2 (cost* or estimat* or index or indice* or measure* or scale* or score* or valu* or weight*)).ti,ab,kf. |
| 73 | ((HRQOL or QOL or quality-of-life) adj3 (assess* or instrument*)).ti,ab,kf. |
| 74 | (HSUV or HUSVs).ti,ab,kf. |
| 75 | (HUI or HUI2 or HUI3).ti,ab,kf. |
| 76 | (multi-attribute or multi-criteria or multiattribute or multicriteria).ti,ab,kf. |
| 77 | ((quality or index) adj2 (wellbeing or well-being)).ti,ab,kf. |
| 78 | (sf6 or sf-6 or short-form-6 or shortform-6 or sf-six or sfsix or shortform-six or short-form-six or shortform6 or short-form6).ti,ab,kf. |
| 79 | standard gamble*.ti,ab,kf. |
| 80 | (time adj (tradeoff or trade-off)).ti,ab,kf. |
| 81 | TTO.ti,ab,kf. |
| 82 | (utility adj (cost* or estimat* or index or indice* or measure* or scale* or score* or valu* or weight*)).ti,ab,kf. |
| 83 | vignette*.ti,ab,kf. |
| 84 | or/63-83 [HSUV] |
| 85 | 62 and 84 [BREAST CA /BREAST non-CA + HSUV] |
| 86 | 47 or 85 [(BREAST CA SCREENING + DECISION-MAKING) OR (BCA/Non-CA + HSUV)] |
| 87 | (Adolescent/ or exp Child/ or exp Infant/) not exp Adult/ |
| 88 | (adolesc* or baby or babies or boy* or child* or fetus or fetal or foet* or girl* or juvenile* or kid or kids or infan* or newborn* or new-born* or neonat* or neo-nat* or paediatr* or pediatr* or preadolesc* or prepubesc* or preteen* or pubescen* or teen* or toddler* or youth*).ti,jn. |
| 89 | 86 not (87 or 88) [CHILD/ADOLESCENT ONLY EXCLUDED] |
| 90 | (exp Animals/ or Models, Animal/ or Disease Models, Animal/) not Humans/ |
| 91 | ((animal or animal-model* or animals or canine* or cat or cats or dog or dogs or feline* or hamster* or lamb or lambs or mice or monkey or monkeys or mouse or murine or pig or pigs or piglet* or porcine or primate* or rabbit* or rat or rats or rodent* or sheep* or swine or veterinar*) not (human* or patient*)).mp. |
| 92 | 89 not (90 or 91) [ANIMAL-ONLY STUDIES EXCLUDED] |
| 93 | (Case Reports.pt. or (case report? or case study or case studies).ti.) not (review* or trial*).ti,kf,hw,pt. |
| 94 | comment/ or editorial/ or (comment or editorial or news or newspaper article).ti,pt. |
| 95 | 92 not (93 or 94) [CASE STUDIES/OPINION PIECES EXCLUDED] |
| 96 | limit 95 to yr="2017 -Current" |
| 97 | limit 96 to (english or french) |
| 98 | remove duplicates from 97 |
| **#** | **Searches** |
| 1 | exp Breast Neoplasms/ |
| 2 | exp Carcinoma, Intraductal, Noninfiltrating/ |
| 3 | ((adenocarcinoma* or adenoma* or cancer* or carcin* or malignan* or metasta*or neoplas* or sarcoma* or tumour* or tumor*) adj3 (breast? or mamma or mammar*)).ti,ab,kf. |
| 4 | (DCIS or (ductal carcinoma adj1 (in situ or insitu))).ti,ab,kf. |
| 5 | ((intra-ductal or intraductal) adj1 carcinoma*).ti,ab,kf. |
| 6 | ((mammogram* or mammograph*) and screening).ti. or ((mammogram* or mammograph*) and screening).ab. /freq=2 |
| 7 | or/1-6 [BREAST CA] |
| 8 | exp *Breast Neoplasms/di and screen*.mp. |
| 9 | exp Early Diagnosis/ and screen*.mp. |
| 10 | exp Magnetic Resonance Imaging/ and screen*.mp. |
| 11 | exp Mammography/ |
| 12 | exp Mass Screening/ |
| 13 | Ultrasonography, Mammary/ and screen*.mp. |
| 14 | (echograph* or echo-mammogra* or echo-tomograph* or echomammogra* or echotomograph*).ti,ab,kf. |
| 15 | (magnetic resonance imag* or magnetic resonance tomograph* or MR tomograph* or MRI or MRIs or NMRI or NMRIs).tw,kf. and screen*.mp. |
| 16 | "breast tomosynthesis".ti,ab,kf. and screen*.mp. |
| 17 | screen*.ti. |
| 18 | screen*.ab. /freq=2 |
| 19 | (sonograph* or ultra-son* or ultra-sound* or ultrason* or ultrasound*).ti,ab,kf. and screen*.mp. |
| 20 | or/8-19 [SCREENING] |
| 21 | 7 and 20 [BREAST CANCER + SCREENING] |
| 22 | attitude to health/ |
| 23 | Choice Behavior/ |
| 24 | Consumer Behavior/ |
| 25 | Decision Making/ |
| 26 | Decision Making, Shared/ |
| 27 | decision support techniques/ |
| 28 | health knowledge, attitudes, practice/ |
| 29 | "patient acceptance of health care"/ |
| 30 | patient participation/ |
| 31 | patient preference/ |
| 32 | patient satisfaction/ |
| 33 | Perception/ |
| 34 | ((acceptabilit* or acceptance or acceptable or attitude* or belief* or deliberat* or expectation* or intent* or jury or perceiv* or perception* or perspective* or prefer* or valuation* or valued or values or views or willing*) adj3 (elicit* or citizen or citizens or client* or consumer* or female* or individual* or panel or participant* or patient* or people* or person* or public* or respondent* or screenee* or stated or user* or wom#n)).ti,ab,kf. |
| 35 | ((choice* or choos*) adj2 (behavio?r* or discrete or experiment*)).ti,ab,kf. |
| 36 | (conjoint adj3 (analy* or design* or evaluation or exercise* or experiment* or studies or study or survey* or task* or valuation or value* or valuing)).ti,ab,kf. |
| 37 | (contingent adj3 (analy* or design* or evaluation or valuation or value* or valuing)).ti,ab,kf. |
| 38 | (decision* adj (board$1 or certain* or conflict* or dissatisf* or satisf* or uncertain*)).ti,ab,kf. |
| 39 | (decision* adj2 (aid$1 or mak* or needs or support* or tool*)).ti,ab,kf. |
| 40 | (decision* adj5 balance).ti,ab,kf. |
| 41 | (ranking or rating).ti,ab,kf. |
| 42 | ((mammograph* or mammogram* or screen*) adj3 (acceptabilit*or acceptance or acceptable or agree* or attitude* or behavio?r* or belief* or choice* or choos* or decid* or decision* or declin* or expectation* or inclin* or intending or intent* or perceiv* or perception* or perspective* or prefer*or propensity or pursue or values or views or willing*)).ti,ab,kf. |
| 43 | (tradeoff* or trade-off*).ti,ab,kf. |
| 44 | or/22-43 [DECISION-MAKING] |
| 45 | 21 and 44 [BREAST CA SCREENING + DECISION-MAKING] |
| 46 | *Genes, BRCA1/ or (BRCA* or gene* or hereditary).ti. |
| 47 | 45 not 46 [BREAST CA SCREENING + DECISION-MAKING - Genetic high-risk patient studies excluded] |
| 48 | exp Breast Neoplasms/ |
| 49 | exp Carcinoma, Intraductal, Noninfiltrating/ |
| 50 | ((adenocarcinoma* or adenoma* or cancer* or carcin* or malignan* or metasta*or neoplas* or sarcoma* or tumour* or tumor*) adj3 (breast? or mamma or mammar*)).ti,ab,kf. |
| 51 | (DCIS or (ductal carcinoma adj1 (in situ or insitu))).ti,ab,kf. |
| 52 | ((intra-ductal or intraductal) adj1 carcinoma*).ti,ab,kf. |
| 53 | or/48-52 [BREAST CA] |
| 54 | Diagnostic Errors/ |
| 55 | False Positive Reactions/ |
| 56 | (false*-positiv* or overdiagnosis or (diagnos* adj3 error*)).ti,ab,kf. |
| 57 | (indetermina* or suspect* or suspicious*).ti,ab,kf. |
| 58 | ("Ductal carcinoma in situ" or DCIS).ti,ab,kf. |
| 59 | or/54-58 |
| 60 | (Breast or ductal or mammogra*).hw,ti,ab,kf. |
| 61 | 59 and 60 [Non-CA] |
| 62 | 53 or 61 [BREAST CA /NOT BREAST CA] |
| 63 | Quality-Adjusted Life Years/ |
| 64 | *"Quality of Life"/ |
| 65 | "Value of Life"/ |
| 66 | (QALY or QALYS).ti,ab,kf. |
| 67 | direct*-elicitation*.ti,ab,kf. |
| 68 | (quality adj adjusted-life).ti,ab,kf. |
| 69 | ("Utility Based Questionnaire-Cancer" or UBQC or UBQC or UBQ-C or "15-Dimension questionnaire" or 15D or "Assessment of QoL" or AQoL or AQoL2 or "wellbeing index" or "well-being index" or "quality of wellbeing" or "quality of well-being" or qwb).ti,ab,kf. |
| 70 | (EuroQol5D or EuroQol or Euro-Qol or EuroQual5D or EuroQual or Euro-Qual or EQ5D or EQ-5D).ti,ab,kf. |
| 71 | (health adj2 (utilit* or disutilit*)).ti,ab,kf. |
| 72 | (health-stat$2 adj2 (cost* or estimat* or index or indice* or measure* or scale* or score* or valu* or weight*)).ti,ab,kf. |
| 73 | ((HRQOL or QOL or quality-of-life) adj3 (assess* or instrument*)).ti,ab,kf. |
| 74 | (HSUV or HUSVs).ti,ab,kf. |
| 75 | (HUI or HUI2 or HUI3).ti,ab,kf. |
| 76 | (multi-attribute or multi-criteria or multiattribute or multicriteria).ti,ab,kf. |
| 77 | ((quality or index) adj2 (wellbeing or well-being)).ti,ab,kf. |
| 78 | (sf6 or sf-6 or short-form-6 or shortform-6 or sf-six or sfsix or shortform-six or short-form-six or shortform6 or short-form6).ti,ab,kf. |
| 79 | standard gamble*.ti,ab,kf. |
| 80 | (time adj (tradeoff or trade-off)).ti,ab,kf. |
| 81 | TTO.ti,ab,kf. |
| 82 | (utility adj (cost* or estimat* or index or indice* or measure* or scale* or score* or valu* or weight*)).ti,ab,kf. |
| 83 | vignette*.ti,ab,kf. |
| 84 | or/63-83 [HSUV] |
| 85 | 62 and 84 [BREAST CA /BREAST non-CA + HSUV] |
| 86 | 47 or 85 [(BREAST CA SCREENING + DECISION-MAKING) OR (BCA/Non-CA + HSUV)] |
| 87 | (Adolescent/ or exp Child/ or exp Infant/) not exp Adult/ |
| 88 | (adolesc* or baby or babies or boy* or child* or fetus or fetal or foet* or girl* or juvenile* or kid or kids or infan* or newborn* or new-born* or neonat* or neo-nat* or paediatr* or pediatr* or preadolesc* or prepubesc* or preteen* or pubescen* or teen* or toddler* or youth*).ti,jn. |
| 89 | 86 not (87 or 88) [CHILD/ADOLESCENT ONLY EXCLUDED] |
| 90 | (exp Animals/ or Models, Animal/ or Disease Models, Animal/) not Humans/ |
| 91 | ((animal or animal-model* or animals or canine* or cat or cats or dog or dogs or feline* or hamster* or lamb or lambs or mice or monkey or monkeys or mouse or murine or pig or pigs or piglet* or porcine or primate* or rabbit* or rat or rats or rodent* or sheep* or swine or veterinar*) not (human* or patient*)).mp. |
| 92 | 89 not (90 or 91) [ANIMAL-ONLY STUDIES EXCLUDED] |
| 93 | (Case Reports.pt. or (case report? or case study or case studies).ti.) not (review* or trial*).ti,kf,hw,pt. |
| 94 | comment/ or editorial/ or (comment or editorial or news or newspaper article).ti,pt. |
| 95 | 92 not (93 or 94) [CASE STUDIES/OPINION PIECES EXCLUDED] |
| 96 | limit 95 to yr="2017 -Current" |
| 97 | limit 96 to (english or french) |
| 98 | remove duplicates from 97 |

APA PsycInfo 1806 to June Week 2 2023

Search date: 19Jun2023

| **#** | **Searches** |
| --- | --- |
| 1 | Breast Neoplasms/ |
| 2 | ((adenocarcinoma* or adenoma* or cancer* or carcin* or malignan* or metasta*or neoplas* or sarcoma* or tumour* or tumor*) adj3 (breast? or mamma or mammar*)).ti,ab. |
| 3 | (DCIS or (ductal carcinoma adj1 (in situ or insitu))).ti,ab. |
| 4 | ((intra-ductal or intraductal) adj1 carcinoma*).ti,ab. |
| 5 | ((mammogram* or mammograph*) and screening).ti,ab. |
| 6 | or/1-5 [BREAST CA] |
| 7 | Cancer Screening/ |
| 8 | Health Screening/ |
| 9 | exp Magnetic Resonance Imaging/ and screen*.mp. |
| 10 | Mammography/ |
| 11 | (echograph* or echo-mammogra* or echo-tomograph* or echomammogra* or echotomograph*).ti,ab. |
| 12 | (magnetic resonance imag* or magnetic resonance tomograph* or MR tomograph* or MRI or MRIs or NMRI or NMRIs).ti,ab. and screen*.mp. |
| 13 | ("breast cancer" or "breast tomosynthesis").ti,ab. and screen*.mp. |
| 14 | screen*.ti. |
| 15 | screen*.ab. /freq=2 |
| 16 | (sonograph* or ultra-son* or ultra-sound* or ultrason* or ultrasound*).ti,ab. and screen*.mp. |
| 17 | or/7-16 [SCREENING] |
| 18 | 6 and 17 [BREAST CA + SCREENING] |
| 19 | *Client Attitudes/ |
| 20 | *Choice Behavior/ |
| 21 | *Consumer Behavior/ |
| 22 | Decision Making/ |
| 23 | Health attitudes/ |
| 24 | Client participation/ |
| 25 | Preferences/ |
| 26 | Client satisfaction/ |
| 27 | ((acceptabilit* or acceptance or acceptable or attitude* or belief* or deliberat* or expectation* or intent* or jury or perceiv* or perception* or perspective* or prefer* or valuation* or valued or values or views or willing*) adj4 (elicit* or citizen or citizens or client* or consumer* or female* or individual* or panel or participant* or patient* or people* or person* or public* or respondent* or screenee* or stated or user* or wom#n)).ti,ab. |
| 28 | ((choice* or choos*) adj2 (behavio?r* or discrete or experiment*)).ti,ab. |
| 29 | (conjoint adj3 (analy* or design* or evaluation or exercise* or experiment* or studies or study or survey* or task* or valuation or value* or valuing)).ti,ab. |
| 30 | (contingent adj3 (analy* or design* or evaluation or valuation or value* or valuing)).ti,ab. |
| 31 | (decision* adj (board$1 or certain* or conflict* or dissatisf* or satisf* or uncertain*)).ti,ab. |
| 32 | (decision* adj2 (aid$1 or mak* or needs or support* or tool*)).ti,ab. |
| 33 | (decision* adj5 balance).ti,ab. |
| 34 | (ranking or rating).ti,ab. |
| 35 | ((mammograph* or mammogram* or screen*) adj4 (acceptabilit*or acceptance or acceptable or agree* or attitude* or behavio?r* or belief* or choice* or choos* or decid* or decision* or declin* or expectation* or inclin* or intending or intent* or perceiv* or perception* or perspective* or prefer*or propensity or pursue or values or views or willing*)).ti,ab. |
| 36 | (tradeoff* or trade-off*).ti,ab. |
| 37 | or/19-36 [DECISION-MAKING] |
| 38 | 18 and 37 [BREAST CA SCREENING + DECISION-MAKING] |
| 39 | 38 not (BRCA* or gene* or hereditary).ti. [Genetic high-risk patient studies excluded] |
| 40 | Breast Neoplasms/ |
| 41 | ((adenocarcinoma* or adenoma* or cancer* or carcin* or malignan* or metasta*or neoplas* or sarcoma* or tumour* or tumor*) adj3 (breast? or mamma or mammar*)).ti,ab. |
| 42 | (DCIS or (ductal carcinoma adj1 (in situ or insitu))).ti,ab. |
| 43 | ((intra-ductal or intraductal) adj1 carcinoma*).ti,ab. |
| 44 | ((mammogram* or mammograph*) and screening).ti,ab. |
| 45 | or/40-44 [BREAST CA] |
| 46 | misdiagnosis/ |
| 47 | type i errors/ |
| 48 | (false*-positiv* or overdiagnosis or (diagnos* adj3 error*)).ti,ab. |
| 49 | (indetermina* or suspect* or suspicious*).ti,ab. |
| 50 | ("Ductal carcinoma in situ" or DCIS).ti,ab. |
| 51 | or/47-50 |
| 52 | (Breast or ductal or mammogra*).hw,ti,ab. |
| 53 | 51 and 52 [Non-CA] |
| 54 | 45 or 53 [BREAST CA /BREAST non-CA] |
| 55 | *"Quality of Life"/ |
| 56 | "Health Related Quality of Life"/ |
| 57 | (QALY or QALYS).ti,ab. |
| 58 | direct*-elicitation*.ti,ab. |
| 59 | (quality adj adjusted-life).ti,ab. |
| 60 | ("Utility Based Questionnaire-Cancer" or UBQC or UBQC or UBQ-C or "15-Dimension questionnaire" or 15D or "Assessment of QoL" or AQoL or AQoL2 or "wellbeing index" or "well-being index" or "quality of wellbeing" or "quality of well-being" or qwb).ti,ab. |
| 61 | (EuroQol5D or EuroQol or Euro-Qol or EuroQual5D or EuroQual or Euro-Qual or EQ5D or EQ-5D).ti,ab. |
| 62 | (health adj2 (utilit* or disutilit*)).ti,ab. |
| 63 | (health-stat$2 adj2 (cost* or estimat* or index or indice* or measure* or scale* or score* or valu* or weight*)).ti,ab. |
| 64 | ((HRQOL or QOL or quality-of-life) adj3 (assess* or instrument*)).ti,ab. |
| 65 | (HSUV or HUSVs).ti,ab. |
| 66 | (HUI or HUI2 or HUI3).ti,ab. |
| 67 | (multi-attribute or multi-criteria or multiattribute or multicriteria).ti,ab. |
| 68 | ((quality or index) adj2 (wellbeing or well-being)).ti,ab. |
| 69 | (sf6 or sf-6 or short-form-6 or shortform-6 or sf-six or sfsix or shortform-six or short-form-six or shortform6 or short-form6).ti,ab. |
| 70 | standard gamble*.ti,ab. |
| 71 | (time adj (tradeoff or trade-off)).ti,ab. |
| 72 | TTO.ti,ab. |
| 73 | (utility adj (cost* or estimat* or index or indice* or measure* or scale* or score* or valu* or weight*)).ti,ab. |
| 74 | vignette*.ti,ab. |
| 75 | or/55-74 [HSUV] |
| 76 | 54 and 75 [BREAST CA /BREAST non-CA + HSUV] |
| 77 | 39 or 76 [(BREAST CA SCREENING + DECISION-MAKING) OR (BCA/Non-CA + HSUV)] |
| 78 | 77 not (adolesc* or baby or babies or boy* or child* or fetus or fetal or foet* or girl* or juvenile* or kid or kids or infan* or newborn* or new-born* or neonat* or neo-nat* or paediatr* or pediatr* or preadolesc* or prepubesc* or preteen* or pubescen* or teen* or toddler* or youth*).ti,jx. [CHILD/ADOLESCENT ONLY EXCLUDED] |
| 79 | 78 not (animal or animal-model* or animals or canine* or cat or cats or dog or dogs or feline or felines or hamster or hamsters or mice or monkey or monkeys or mouse or pig or piglet or piglets or pigs or porcine or primate* or rabbit or rabbits or rat or rats or rodent or rodents or sheep or swine or swines).ti. [ANIMAL-ONLY STUDIES EXCLUDED] |
| 80 | (comment* or editor* or letter).ti. |
| 81 | (case report* or case stud*).ti. |
| 82 | 79 not (80 or 81) [CASE STUDIES/OPINION PIECES EXCLUDED] |
| 83 | limit 82 to yr="2017 -Current" |
| 84 | limit 83 to (english or french) |
| 85 | remove duplicates from 84 |

CINAHL Plus with Full Text (1937 to current)

Search date: 19Jun2023

| **#** | **Query** |
| --- | --- |
| S1 | (MH "Breast Neoplasms") |
| S2 | (MH "Carcinoma, Ductal, Breast") |
| S3 | ((adenocarcinoma* or adenoma* or cancer* or carcin* or malignan* or metasta*or neoplas* or sarcoma* or tumour* or tumor*) N2 (breast* or mamma or mammar*)) |
| S4 | (DCIS or ("ductal carcinoma" N0 ("in situ" or insitu"))) |
| S5 | (("intra-ductal" or intraductal) N0 carcinoma*) |
| S6 | (mammogram* or mammograph*) AND screening |
| S7 | S1 OR S2 OR S3 OR S4 OR S5 OR S6 |
| S8 | (MM "Breast Neoplasms+/DI/RA/US") and TX screen* |
| S9 | (MH "Cancer Screening") |
| S10 | (MH "Early Detection of Cancer") and TX screen* |
| S11 | (MH "Magnetic Resonance Imaging+") and TX screen* |
| S12 | (MH "Mammography") |
| S13 | (MH "Health Screening") |
| S14 | (MH "Ultrasonography") and TX screen* |
| S15 | (echograph* or "echo-mammogra*" or "echo-tomograph*" or echomammogra* or echotomograph*) |
| S16 | ("magnetic resonance imag*" or "magnetic resonance tomograph*" or "MR tomograph*" or MRI or MRIs or NMRI or NMRIs) and TX screen* |
| S17 | "breast tomosynthesis" and TX screen* |
| S18 | TI screen* |
| S19 | (sonograph* or "ultra-son*" or "ultra-sound*" or ultrason* or ultrasound*) and TX screen* |
| S20 | S8 OR S9 OR S10 OR S11 OR S12 OR S13 OR S14 OR S15 OR S16 OR S17 OR S18 OR S19 |
| S21 | S7 AND S20 |
| S22 | (MH "Attitude to Health") |
| S23 | (MH "Patient Attitudes") |
| S24 | (MH "Consumer Attitudes") |
| S25 | (MH "Consumer Participation") |
| S26 | (MH "Cooperative Behavior") |
| S27 | (MH "Patient Compliance") |
| S28 | (MH "Decision Making") |
| S29 | (MH "Decision Making, Patient") |
| S30 | (MH "Decision Making, Shared") |
| S31 | (MH "Decision Support Techniques") |
| S32 | (MH "Health Beliefs") |
| S33 | (MM "Mammography/PF") |
| S34 | (acceptabilit* or acceptance or acceptable or attitude* or belief* or deliberat* or expectation* or intent* or jury or perceiv* or perception* or perspective* or prefer* or valuation* or valued or values or views or willing*) N3 (elicit* or citizen or citizens or client* or consumer* or female* or individual* or panel or participant* or patient* or people* or person* or public* or respondent* or screenee* or stated or user* or wom?n) |
| S35 | (choice* or choos*) N1 (behavio#r* or discrete or experiment*) |
| S36 | conjoint N2 (analy* or design* or evaluation or exercise* or experiment* or studies or study or survey* or task* or valuation or value* or valuing) |
| S37 | contingent N2 (analy* or design* or evaluation or valuation or value* or valuing) |
| S38 | decision* W0 (board# or certain* or conflict* or dissatisf* or satisf* or uncertain*) |
| S39 | decision* N1 (aid# or mak* or needs or support* or tool*) |
| S40 | decision* N4 balance |
| S41 | ranking or rating |
| S42 | (mammograph* or mammogram* or screen*) N3 (acceptabilit* or acceptance or acceptable or agree* or attitude* or behavio#r* or belief* or choice* or choos* or decid* or decision* or declin* or expectation* or inclin* or intending or intent* or perceiv* or perception* or perspective* or prefer* or propensity or pursue or values or views or willing*) |
| S43 | tradeoff* or trade-off* |
| S44 | S22 OR S23 OR S24 OR S25 OR S26 OR S27 OR S28 OR S29 OR S30 OR S31 OR S32 OR S33 OR S34 OR S35 OR S36 OR S37 OR S38 OR S39 OR S40 OR S41 OR S42 OR S43 |
| S45 | S21 AND S44 |
| S46 | (MH "Breast Neoplasms") |
| S47 | (MH "Carcinoma, Ductal, Breast") |
| S48 | ((adenocarcinoma* or adenoma* or cancer* or carcin* or malignan* or metasta*or neoplas* or sarcoma* or tumour* or tumor*) N2 (breast* or mamma or mammar*)) |
| S49 | (DCIS or ("ductal carcinoma" N0 ("in situ" or insitu"))) |
| S50 | (("intra-ductal" or intraductal) N0 carcinoma*) |
| S51 | S46 OR S47 OR S48 OR S49 OR S50 |
| S52 | (MH "Diagnostic Errors+") |
| S53 | (MH "False Positive Results") |
| S54 | false*-positiv* or overdiagnosis or (diagnos* N2 error*) |
| S55 | indetermina* or suspect* or suspicious* |
| S56 | "Ductal carcinoma in situ" or DCIS |
| S57 | S52 OR S53 OR S54 OR S55 OR S56 |
| S58 | Breast or ductal or mammogra* |
| S59 | S57 AND S58 |
| S60 | S51 OR S59 |
| S61 | (MH "Quality-Adjusted Life Years") |
| S62 | (MM "Quality of Life") |
| S63 | (MH "Economic Value of Life") |
| S64 | QALY or QALYS |
| S65 | direct*-elicitation* |
| S66 | quality W0 adjusted-life |
| S67 | "Utility Based Questionnaire-Cancer" or UBQC or UBQC or UBQ-C or "15-Dimension questionnaire" or 15D or "Assessment of QoL" or AQoL or AQoL2 or "wellbeing index" or "well-being index" or "quality of wellbeing" or "quality of well-being" or qwb |
| S68 | EuroQol5D or EuroQol or Euro-Qol or EuroQual5D or EuroQual or Euro-Qual or EQ5D or EQ-5D |
| S69 | health N1 (utilit* or disutilit*) |
| S70 | (health-state OR health-states) N1 (cost* or estimat* or index or indice* or measure* or scale* or score* or valu* or weight*) |
| S71 | (HRQOL or QOL or quality-of-life) N2 (assess* or instrument*) |
| S72 | HSUV or HUSVs |
| S73 | HUI or HUI2 or HUI3 |
| S74 | multi-attribute or multi-criteria or multiattribute or multicriteria |
| S75 | (quality or index) N1 (wellbeing or well-being) |
| S76 | sf6 or sf-6 or short-form-6 or shortform-6 or sf-six or sfsix or shortform-six or short-form-six or shortform6 or short-form6 |
| S77 | time W0 (tradeoff or trade-off) |
| S78 | TTO |
| S79 | utility W0 (cost* or estimat* or index or indice* or measure* or scale* or score* or valu* or weight*) |
| S80 | TI vignette* OR AB vignette* |
| S81 | S61 OR S62 OR S63 OR S64 OR S65 OR S66 OR S67 OR S68 OR S69 OR S70 OR S71 OR S72 OR S73 OR S74 OR S75 OR S76 OR S77 OR S78 OR S79 OR S80 |
| S82 | S60 AND S81 |
| S83 | S45 OR S82 |
| S84 | ((MH "Adolescents+") OR (MH "Child+") OR (MH "Infant+")) NOT (MH "Adult+") |
| S85 | TI (adolesc* or baby or babies or boy* or child* or fetus or fetal or foet* or girl* or juvenile* or kid or kids or infan* or newborn* or "new-born*" or neonat* or "neo-nat*" or paediatr* or pediatr* or preadolesc* or prepubesc* or preteen* or pubescen* or teen* or toddler* or youth*) |
| S86 | S83 NOT (S84 OR S85) |
| S87 | ((MH "Vertebrates+") NOT MH Human) |
| S88 | (animal or animal-model* or animals or canine* or cat or cats or dog or dogs or feline* or hamster* or lamb or lambs or mice or monkey or monkeys or mouse or murine or pig or pigs or piglet* or porcine or primate* or rabbit* or rat or rats or rodent* or sheep* or swine or veterinar*) NOT (human* or patient*) |
| S89 | S86 NOT (S87 OR S88) |
| S90 | TI (comment* or editor*) |
| S91 | PT (Commentary or Editorial or Letter) |
| S92 | TI ("case report*" or "case stud*") |
| S93 | PT "Case Study" |
| S94 | S89 NOT (S90 OR S91 OR S92 OR S93) |
| S95 | S89 NOT (S90 OR S91 OR S92 OR S93) |

**Grey Literature Searches**


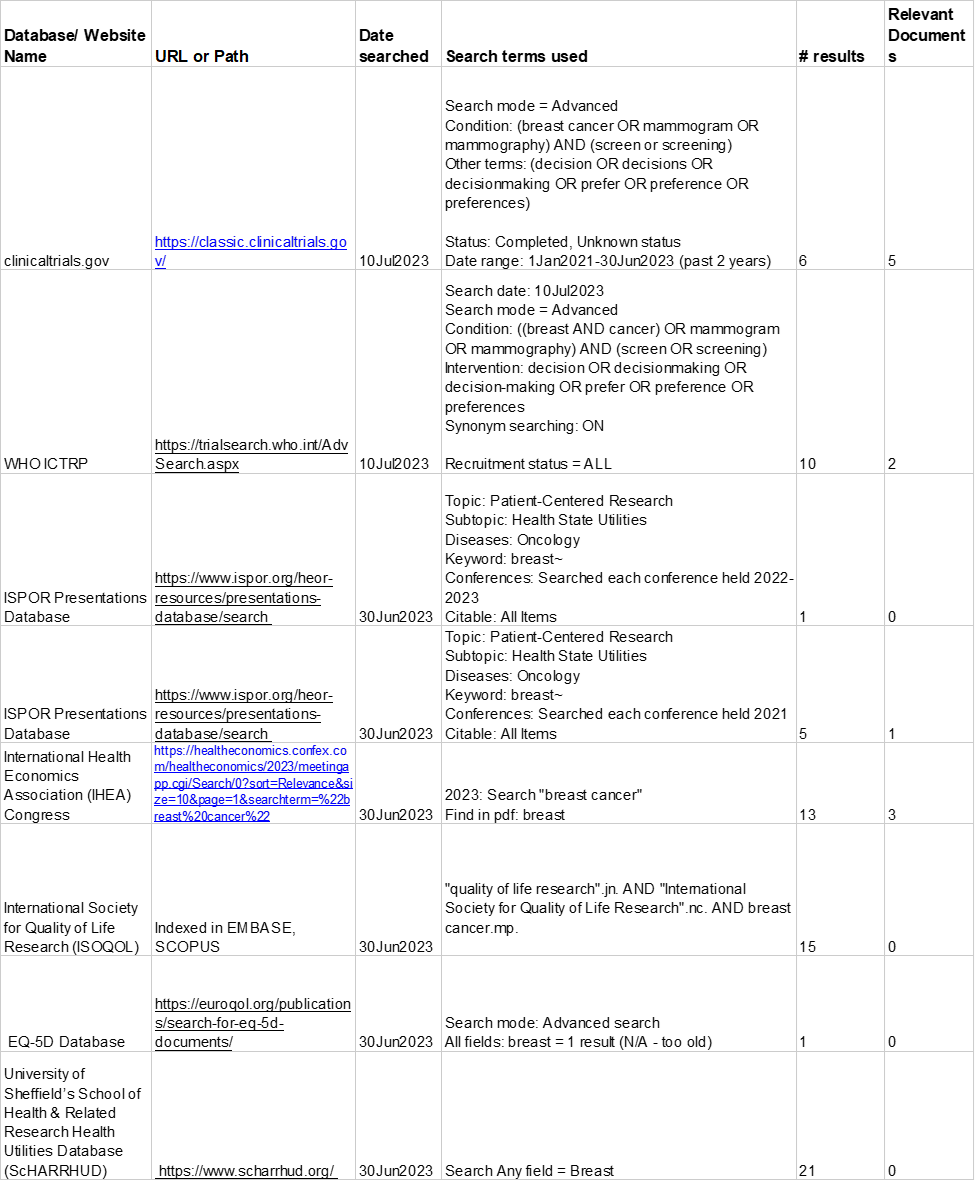


**List of Excluded Studies, by main reason**

This list *does not* include 481 full texts excluded that included “quality of life” in title/abstracts but had no health-state utility data upon full text review

Country (N=56):

1. Alizadeh-Sabeg P, Mehrabi E, Nourizadeh R, Hakimi S, Mousavi S. The effect of motivational interviewing on the change of breast cancer screening behaviors among rural Iranian women. Patient Education and Counseling. 2021;104(2):369-74.
2. ACTION Study Group. Health-related quality of life and psychological distress among cancer survivors in Southeast Asia: results from a longitudinal study in eight low- and middle-income countries. BMC Medicine. 2017;15(1):10.
3. Chen N, Yang Q, Li YF, Guo Q, Huang DY, Peng JL. Cost-utility analysis of different venous access devices in breast cancer patients: a decision-based analysis model. BMC Health Services Research. 2023;23(1):497.
4. Delaloge S, Cella D, Ye Y, Buyse M, Chan A, Barrios CH, et al. Effects of neratinib on health-related quality of life in women with HER2-positive early-stage breast cancer: longitudinal analyses from the randomized phase III ExteNET trial. Annals of Oncology. 2019;30(4):567-74.
5. Dhakal R, Adhikari C, Karki P, Neupane N, Bhandari P, Gurung A, et al. Attitude sustains longer than subjective norm and perceived behavioral control: results of breast cancer screening educational intervention. PloS One. 2023;18(2):e0281184.
6. Etikasari R, Andayani TM, Endarti D, Taroeno-Hariadi KW. Health related quality of life among postmenopausal woman with hormone responsive HER2- breast cancer in Indonesia. Journal of Basic and Clinical Physiology and Pharmacology. 2021;32(4):561-5.
7. Fathollahi-Dehkordi F, Farajzadegan Z. Health education models application by peer group for improving breast cancer screening among Iranian women with a family history of breast cancer: a randomized control trial. MJIRI. 2018;32:51.
8. Guerra RL, Dos Reis NB, Correa FDM, Fernandes MM, Ribeiro Alves Fernandes R, Cancela MDC, et al. Breast cancer quality of life and health-state utility at a Brazilian reference public cancer center. Expert Review of Pharmacoeconomics & Outcomes Research. 2020;20(2):185-91.
9. Gupta N, Pandey AK, Dimri K, Jyani G, Goyal A, Prinja S. Health-related quality of life among breast cancer patients in India. Supportive Care in Cancer. 2022;30(12):9983-90.
10. Karimabadi A, Pourahmadi E, Bafandeh Imandoust S, Nikoukar A, Aryafar M. Knowledge, practice, preferences and willingness-to-pay for mammographic screening tests among Iranian women: a contingent valuation method. APJCP. 2022;23(4):1207-13.
11. Kathrikolly TR, Shetty RS, Nair S. Opportunities and barriers to breast cancer screening in a rural community in coastal karnataka, India: a qualitative analysis. APJCP. 2020;21(9):2569-75.
12. Khadela A, Bhikadiya V, Vyas B. Impact of oncology pharmacist services on humanistic outcome in patients with breast cancer. Journal of Oncology Pharmacy Practice. 2022;28(2):302-9.
13. Khani Jeihooni A, Moayedi ZS, Momenabadi V, Ghalegolab F, Afzali Harsini P. Effect of educational intervention based on theory of planned behavior (TPB) on doing breast self-examination in a sample of Iranian women. Breast Cancer: Basic and Clinical Research. 2023;17:11782234221145417.
14. Khani Jeihooni A, Darvishi N, Harsini PA. The effect of educational intervention based on the theory of planned behavior on mammography screening in Iranian women. Journal of Cancer Education. 2020;35(2):264-73.
15. Kohler RE, Miller AR, Gutnik L, Lee CN, Gopal S. Experiences and perceptions regarding clinical breast exam screening by trained laywomen in Malawi. CCC. 2017;28(2):137-43.
16. Li S, Wang M, Liu L, Chen G. Which approach is better in eliciting health state utilities from breast cancer patients? Evidence from mainland China. European Journal of Cancer Care. 2019;28(2):e12965.
17. Liu L, Li S, Wang M, Chen G. Comparison of EQ-5D-5L health state utilities using four country-specific tariffs on a breast cancer patient sample in mainland China. Patient Preference and Adherence. 2017;11:1049-56.
18. Liu T, Li S, Wang M, Sun Q, Chen G. Mapping the Chinese version of the EORTC QLQ-BR53 onto the EQ-5D-5L and SF-6D utility scores. The Patient: Patient-Centered Outcomes Research. 2020;13(5):537-55.
19. Mahalakshmi S, Suresh S. Barriers to cancer screening uptake in women: a qualitative study from Tamil Nadu, India. APJCP. 2020;21(4):1081-7.
20. Mirmoammadi A, Parsa P, Khodakarami B, Roshanaei G. Effect of consultation on adherence to clinical breast examination and mammography in Iranian women: a randomized control trial. APJCP. 2018;19(12):3443-9.
21. Nahvijou A, Safari H, Ameri H. Psychometric properties of the SF-6Dv2 in an Iranian breast cancer population. Breast Cancer. 2021;28(4):937-43.
22. Nahvijou A, Safari H, Yousefi M, Rajabi M, Arab-Zozani M, Ameri H. Mapping the cancer-specific FACT-B onto the generic SF-6Dv2. Breast Cancer. 2021;28(1):130-6.
23. Naserian N, Ansari S, Abedi P. Comparison of training via short messages and group training on level of knowledge and practice of middle-aged women about breast cancer screening tests. Journal of Cancer Education. 2018;33(5):1036-42.
24. Ngan TT, Mai VQ, Van Minh H, Donnelly M, O'Neill C. Health-related quality of life among breast cancer patients compared to cancer survivors and age-matched women in the general population in Vietnam. Quality of Life Research. 2022;31(3):777-87.
25. Pirzadeh A, Ansari S, Golshiri P. The effects of educational intervention on breast self-examination and mammography behavior: application of an integrated model. Journal of Education and Health Promotion. 2021;10(1):196.
26. Sabermahani A, Mohammad Taghizade S, Goodarzi R. A comparative study on willingness to pay for breast cancer and osteoporosis screening in Kerman, Southeastern Iran. Iranian Journal of Public Health. 2017;46(5):693-8.
27. Safizade H, Amirzadeh N, Mangolian Shahrbabaki P. Motivational factors for breast cancer screening behaviors in Iranian women: a qualitative study. APJCP. 2020;21(10):3109-14.
28. Sarker R, Islam MS, Moonajilin MS, Rahman M, Gesesew HA, Ward PR. Effectiveness of educational intervention on breast cancer knowledge and breast self-examination among female university students in Bangladesh: a pre-post quasi-experimental study. BMC Cancer. 2022;22(1):199.
29. Setyowibowo H, Hunfeld JAM, Iskandarsyah A, Yudiana W, Passchier J, Sadarjoen SS, et al. A self-help intervention for reducing time to diagnosis in Indonesian women with breast cancer symptoms. Psycho-Oncology. 2020;29(4):696-702.
30. Setyowibowo H, Purba FD, Hunfeld JAM, Iskandarsyah A, Sadarjoen SS, Passchier J, et al. Quality of life and health status of Indonesian women with breast cancer symptoms before the definitive diagnosis: a comparison with Indonesian women in general. PloS One. 2018;13(7):e0200966.
31. Shen L, Zhou J, Chen Y, Ding J, Wei H, Liu J, et al. Treatment patterns, effectiveness, and patient-reported outcomes of palbociclib therapy in Chinese patients with advanced breast cancer: a multicenter ambispective real-world study. Cancer Medicine. 2022;11(22):4157-68.
32. Sibhat SG, Fenta TG, Sander B, Gebretekle GB. Health-related quality of life and its predictors among patients with breast cancer at Tikur Anbessa Specialized Hospital, Addis Ababa, Ethiopia. Health and Quality of Life Outcomes. 2019;17(1):165.
33. Su M, Hua X, Wang J, Yao N, Zhao D, Liu W, et al. Health-related quality of life among cancer survivors in rural China. Quality of Life Research. 2019;28(3):695-702.
34. Tang L, Pang Y, He Y, Shi Q, Han X, Li Z, et al. Longitudinal study of symptom burden in outpatients with advanced cancers based on electronic Patient-Reported Outcome (ePRO) platform: a single institution, prospective study protocol. BMJ Open. 2020;10(11):e038223.
35. Tumeh IBRG, Bergerot CD, Lee D, Philip EJ, Freitas-Junior R. MHealth program for patients with advanced cancer receiving treatment in a public health hospital in Brazil. Psycho-Oncology. 2023;32(1):125-32.
36. Wadasadawala T, Mohanty SK, Sen S, Khan PK, Pimple S, Mane JV, et al. Health-related quality of life (HRQoL) using EQ-5D-5L: value set derived for Indian breast cancer cohort. APJCP. 2023;24(4):1199-207.
37. Wang L, Shi J-F, Zhu J, Huang H-Y, Bai Y-N, Liu G-X, et al. Health-related quality of life and utility scores of patients with breast neoplasms in China: a multicenter cross-sectional survey. Breast. 2018;39:53-62.
38. Wu T-Y, Hoffman JL. Breast cancer early detection: an academic-community partnership in the Philippines. Clinical Journal of Oncology Nursing. 2019;23(5):547-51.
39. Wu T-Y, Lee J. Promoting breast cancer awareness and screening practices for early detection in low-resource settings. European Journal of Breast Health. 2019;15(1):18-25.
40. Yang Q, Yu X, Zhang W. Health variations among breast-cancer patients from different disease states: evidence from China. BMC Health Services Research. 2020;20(1):1033.
41. Yang Q, Yu XX, Zhang W, Li H. Mapping function from FACT-B to EQ-5D-5 L using multiple modelling approaches: data from breast cancer patients in China. Health and Quality of Life Outcomes. 2019;17(1):153.
42. Ye M, Lu J, Yang F, Wu B. Economic evaluation of letrozole for early breast cancer in a health resource-limited setting. BioMed Research International. 2018;2018:9282646.
43. Yousefi M, Nahvijou A, Sari AA, Ameri H. Mapping QLQ-C30 onto EQ-5D-5L and SF-6D-V2 in patients with colorectal and breast cancer from a developing country. Value in Health Regional Issues. 2021;24:57-66.
44. Zhu J, Yan X-X, Liu C-C, Wang H, Wang L, Cao S-M, et al. Comparing EQ-5D-3L and EQ-5D-5L performance in common cancers: suggestions for instrument choosing. Quality of Life Research. 2021;30(3):841-54.
45. Zhu J, Wang L, Huang H-Y, Bai F-Z, Li J, Fang Y, et al. Short-term impact of breast cancer screening intervention on health-related quality of life in China: a multicentre cross-sectional survey. Psycho-Oncology. 2019;28(9):1836-44.
46. Nosratnejad S, Rahmani S, Yousefi M, Moeeni M. Women's willingness to pay for mammography screening under two medical information scenarios. European Journal of Cancer Care. 2023:1-5.
47. Meshkani Z, Moradi N, Aboutorabi A, Jafari A, Shams R. Subjective valuation of Iranian women for screening for gene-related diseases: a case of breast cancer. BMC Public Health. 2023;23(1):1-9.
48. Sardasht FG, Irani M, Najmabadi KM, Hadiababd SFN, Fasanghari M. Breast cancer screening behaviors based on health belief model. Journal of Holistic Nursing & Midwifery. 2022;32(2):89-97.
49. Jeyapaul S, Oommen AM, Cherian AG, Marcus TA, Malini T, Prasad JH, et al. Feasibility, uptake and real-life challenges of a rural cervical and breast cancer screening program in Vellore, Tamil Nadu, South India. Indian Journal of Cancer. 2021;58(3):417-24.
50. Zeng X, Sui M, Liu B, Yang H, Liu R, Tan RL-Y, et al. Measurement properties of the EQ-5D-5L and EQ-5D-3L in six commonly diagnosed cancers. Patient. 2021;14(2):209-22.
51. Siebers C, Appelman L, Appelman P, Go S, Van Oirsouw M, Broeders M, et al. 384 Poster - Patients' experience with mammography and attitude towards targeted breast ultrasound as initial imaging technique for the evaluation of focal breast complaints...12th European Breast Cancer Conference (EBCC-12), October 2-3, 2020 (Virtual Conference). European Journal of Cancer. 2020;138:S98-S.
52. Xie S, Wu J, He X, Chen G, Brazier JE. Do discrete choice experiments approaches perform better than time trade-off in eliciting health state utilities? Evidence from SF-6Dv2 in China. Value in Health. 2020;23(10):1391-9.
53. Nisha B, Murali R. Impact of health education intervention on breast cancer awareness among rural women of Tamil Nadu. Indian Journal of Community Medicine. 2020;45(2):149-53.
54. Zolfaghari Z, Rezaee N, Shakiba M, Navidian A. The effect of motivational interviewing-based training compared with conventional training on the frequency of breast cancer screening tests in female teachers: a quasi-experimental study. Medical-Surgical Nursing Journal. 2018;7(4):1-7.
55. Cruz TSP. Breast cancer risk perception, knowledge, attitudes, beliefs and screening behaviors of Chamorro women in Guam. The University of Arizona. 2017:1-1.
56. Gusain S, Koul P, Mala YM. Perception of women regarding breast and cervical screening and its association with demographic variables. International Journal of Nursing Education. 2017;9(1):30-5.

Exposure - Not weighing 1+ benefit and 1+ harm (N=72):

1. Abbas MO, Baig M. Knowledge and practice concerning breast cancer risk factors and screening among females in UAE. APJCP. 2023;24(2):479-87.
2. Abelson J, Tripp L, Brouwers MC, Pond G, Sussman J. Uncertain times: a survey of Canadian women's perspectives toward mammography screening. Preventive Medicine. 2018;112:209-15.
3. Acheampong T, Rodriguez CB, O'Neill SC, Agovino M, Argov EJL, Tehranifar P. Scientific uncertainty and perceived mammography benefits in women screened for breast cancer. CCC. 2023;34(7):611-9.
4. Alameer A, Mahfouz MS, Alamir Y, Ali N, Darraj A. Effect of health education on female teachers' knowledge and practices regarding early breast cancer detection and screening in the Jazan area: a quasi-experimental study. Journal of Cancer Education. 2019;34(5):865-70.
5. Alizadeh Sabeg P, Mehrabi E, Nourizadeh R, Poursharifi H, Mousavi S. The effect of counseling on breast cancer awareness in rural Iranian women: a randomized controlled clinical trial. Journal of Cancer Education. 2019;34(6):1083-91.
6. Aminawung JA, Hoag JR, Kyanko KA, Xu X, Richman IB, Busch SH, Gross CP. Breast cancer supplemental screening: women's knowledge and utilization in the era of dense breast legislation. Cancer Medicine. 2020;9(15):5662-71.
7. Bashirian S, Barati M, Mohammadi Y, MoaddabShoar L, Dogonchi M. Evaluation of an intervention program for promoting breast self-examination behavior in employed women in Iran. Breast Cancer. 2021;15:1178223421989657.
8. Biederman E, Baltic R, Katz ML, Rawl S, Vachon E, Monahan PO, et al. Increasing breast, cervical, and colorectal cancer screening among rural women: baseline characteristics of a randomized control trial. Contemporary Clinical Trials. 2022;123:106986.
9. Borrayo EA, Rosales M, Gonzalez P. Entertainment-education narrative versus nonnarrative interventions to educate and motivate latinas to engage in mammography screening. Health Education & Behavior. 2017;44(3):394-402.
10. Bowen DJ, Robbins R, Bush N, Meischke H, Ludwig A, Wooldridge J. Effects of a web-based intervention on women's breast health behaviors. Translational Behavioral Medicine. 2017;7(2):309-19.
11. Bucher A, Blazek ES, West AB. Feasibility of a reinforcement learning-enabled digital health intervention to promote mammograms: retrospective, single-arm, observational study. JMIR Formative Research. 2022;6(11):e42343.
12. Chapman BM, Yang J-C, Gonzalez JM, Havrilesky L, Reed SD, Hwang ES. Patient preferences for outcomes following DCIS management strategies: a discrete choice experiment. JCO Oncology Practice. 2021;17(11):e1639-e48.
13. Colon-Lopez V, Gonzalez D, Velez C, Fernandez-Espada N, Feldman-Soler A, Ayala-Escobar K, et al. Community-academic partnership to implement a breast and cervical cancer screening education program in Puerto Rico. Puerto Rico Health Sciences Journal. 2017;36(4):191-7.
14. Cooper J, Jones G, Carlin R. Lightning can strike twice: a national partnership promoting breast health among women with disabilities. Progress in Community Health Partnerships: Research, Education, and Action. 2022;16(4):599-606.
15. Cumberland WG, Berman BA, Zazove P, Sadler GR, Jo A, Booth H, et al. A breast cancer education program for D/deaf women. American Annals of the Deaf. 2018;163(2):90-115.
16. Duarte F. Encouraging mammograms using behavioral economics: a randomized controlled trial in Chile. Value in Health. 2021;24(10):1463-9.
17. DuBenske LL, Schrager S, McDowell H, Wilke LG, Trentham-Dietz A, Burnside ES. Mammography screening: gaps in patient's and physician's needs for shared decision-making. The Breast Journal. 2017;23(2):210-4.
18. Ghanouni A, Waller J, Stoffel ST, Vlaev I, von Wagner C. Acceptability of risk-stratified breast screening: effect of the order of presenting risk and benefit information. Journal of Medical Screening. 2020;27(1):52-6.
19. Han H-R, Song Y, Kim M, Hedlin HK, Kim K, Ben Lee H, Roter D. Breast and cervical cancer screening literacy among Korean American women: a community health worker-led intervention. American Journal of Public Health. 2017;107(1):159-65.
20. Haynes D, Hughes K, Haas M, Richards GL, Robinson B. Breast Cancer Champions: a peer-to-peer education and mobile mammography program improving breast cancer screening rates for women of African heritage. CCC. 2023;34(7):625-33.
21. Hernandez-Leal MJ, Perez-Lacasta MJ, Cardona-Cardona A, Codern-Bove N, Vidal-Lancis C, Rue M, et al. Women's preference to apply shared decision-making in breast cancer screening: a discrete choice experiment. BMJ Open. 2022;12(11):e064488.
22. Hoover DS, Pappadis MR, Housten AJ, Krishnan S, Weller SC, Giordano SH, et al. Preferences for communicating about breast cancer screening among racially/ethnically diverse older women. Health Communication. 2019;34(7):702-6.
23. Housten AJ, Hoover DS, Britton M, Bevers TB, Street RL, McNeill LH, et al. Perceptions of conflicting breast cancer screening recommendations among racially/ethnically diverse women: a multimethod study. Journal of General Internal Medicine. 2022;37(5):1145-54.
24. Huerta EE, Weeks-Coulthurst P, Williams C, Swain SM. Take care of your neighborhood. Breast Cancer Research and Treatment. 2018;167(1):225-34.
25. Klippert H, Schaper A. Using Facebook to communicate mammography messages to rural audiences. Public Health Nursing. 2019;36(2):164-71.
26. Kocaoz S, Ozcelik H, Talas MS, Akkaya F, Ozkul F, Kurtulus A, Unlu F. The effect of education on the early diagnosis of breast and cervix cancer on the women's attitudes and behaviors regarding participating in screening programs. Journal of Cancer Education. 2018;33(4):821-32.
27. Kregting LM, van Ravesteyn NT, Spijker W, Dierks T, Aitken CA, Geuzinge HA, Korfage IJ. Effects of a leaflet on breast cancer screening knowledge, explicit attitudes, and implicit associations. Patient Education and Counseling. 2020.
28. Lee EE, Brecht M-L, Park H, Lee J, Oh KM. Web-based study for improving mammography among Korean American women. Journal of Cancer Education. 2017;32(2):257-63.
29. Lipscomb J, Escoffery C, Gillespie TW, Henley SJ, Smith RA, Chociemski T, et al. Improving screening uptake among breast cancer survivors and their first-degree relatives at elevated risk to breast cancer: results and implications of a randomized study in the state of Georgia. International Journal of Environmental Research and Public Health. 2020;17(3).
30. Litaker JR, Tamez N, Durkalski W, Taylor R. A cue-to-action pilot project to increase screening mammography. AJMC. 2021;27(2):e48-e53.
31. Lofters A, Jain A, Siu W, Kyte M, Lee-Foon N, Scott F, Nnorom O. Ko-Pamoja: the feasibility of a lay health educator-led breast and cervical screening program for Black women in Ontario, Canada (short report). CCC. 2017;28(11):1207-18.
32. Mandrik O, Yaumenenka A, Herrero R, Jonker MF. Population preferences for breast cancer screening policies: discrete choice experiment in Belarus. PloS One. 2019;14(11):e0224667.
33. Margulies IG, Zwillenberg J, Chadda A, Gissel H, Lettera M, Bender S, et al. Monitoring and developing a volunteer patient navigation intervention to improve mammography compliance in a safety net hospital. Journal of Oncology Practice. 2019;15(4):e389-e98.
34. McBride K, Gesink D. Increasing cancer screening among old order Anabaptist women through specialized women's health and integrated cancer screening interventions. Journal of Immigrant and Minority Health. 2018;20(2):465-78.
35. Molina Y, San Miguel LG, Tamayo L, Pichardo C, Torres P, Robledo C, et al. Comparing different interventions' effects on Latinas’ screening mammography attainment and participant-driven information diffusion. Health Education & Behavior. 2021;48(6):818-30.
36. Molina Y, San Miguel LG, Tamayo L, Robledo C, Diaz CS, Lucio A, et al. The "Empowering Latinas to Obtain Breast Cancer Screenings" study: rationale and design. Contemporary Clinical Trials. 2018;71:1-8.
37. Molina Y, Kim SJ, Berrios N, Glassgow AE, San Miguel Y, Darnell JS, et al. Patient navigation improves subsequent breast cancer screening after a noncancerous result: evidence from the patient navigation in medically underserved areas study. Journal of Women's Health. 2018;27(3):317-23.
38. Nagler RH, Franklin Fowler E, Gollust SE. Women's awareness of and responses to messages about breast cancer overdiagnosis and overtreatment: results from a 2016 national survey. Medical Care. 2017;55(10):879-85.
39. Nolan TS, Tan A, Williams KP. The ties that bind: Cancer history, communication, and screening intention associations among diverse families. Journal of Medical Screening. 2021;28(2):108-13.
40. Padela AI, Malik S, Ally SA, Quinn M, Hall S, Peek M. Reducing Muslim mammography disparities: outcomes from a religiously tailored mosque-based intervention. Health Education & Behavior. 2018;45(6):1025-35.
41. Pratt R, Mohamed S, Dirie W, Ahmed N, Lee S, VanKeulen M, Carlson S. Testing a religiously tailored intervention with Somali American Muslim women and Somali American Imams to increase participation in breast and cervical cancer screening. Journal of Immigrant and Minority Health. 2020;22(1):87-95.
42. Qin X, Nagler RH, Fowler EF, Gollust SE. U.S. women's perceived importance of the harms and benefits of mammograms and associations with screening ambivalence: results from a national survey. Preventive Medicine. 2019;123:130-7.
43. Ritchie D, Van Hal G, Van den Broucke S. Factors affecting intention to screen after being informed of benefits and harms of breast cancer screening: a study in 5 European countries in 2021. Archives of Public Health. 2022;80(1):143.
44. Rossell E-L, Bo A, Gronborg TK, Kristiansen IS, Borgquist S, Scherer LD, Stovring H. Danish women want to participate in a hypothetical breast cancer screening with harms and no reduction in mortality: a cross-sectional survey. Medical Decision Making. 2023;43(4):403-16.
45. Salinas JJ, Byrd T, Martin C, Dwivedi AK, Alomari A, Salaiz R, Shokar NK. Change in breast cancer screening knowledge is associated with change in mammogram intention in Mexican-Origin women after an educational intervention. Breast Cancer: Basic and Clinical Research. 2018;12:1178223418782904.
46. Schoenborn NL, Nagler RH, Schonberg MA, Pollack CE, Boyd CM, Xue Q-L, Gollust SE. Willingness to stop screening mammograms among older women in the United States: results from a national survey. Journal of General Internal Medicine. 2023;38(4):1091-3.
47. Schonberg MA, Karamourtopoulos M, Jacobson AR, Aliberti GM, Pinheiro A, Smith AK, et al. A strategy to prepare primary care clinicians for discussing stopping cancer screening with adults older than 75 years. Innovation in Aging. 2020;4(4):igaa027.
48. Schrager S, Evaristo C, Little T, DuBenske L, Burnside ES. Patient and clinician characteristics that predict breast cancer screening behavior in 40-49-year-old women. Journal of Patient-centered Research and Reviews. 2021;8(4):331-5.
49. Sinicrope PS, Bauer MC, Patten CA, Austin-Garrison M, Garcia L, Hughes CA, et al. Development and evaluation of a cancer literacy intervention to promote mammography screening among Navajo women: a pilot study. AJHP. 2020;34(6):681-5.
50. Smith J, Dodd RH, Hersch J, Cvejic E, McCaffery K, Jansen J. Effect of different communication strategies about stopping cancer screening on screening intention and cancer anxiety: a randomised online trial of older adults in Australia. BMJ Open. 2020;10(6):e034061.
51. Spalluto LB, Audet CM, Murry VM, Barajas CP, Beard KR, Campbell TT, et al. Group versus individual educational sessions with a Promotora and Hispanic/Latina women's satisfaction with care in the screening mammography setting: a randomized controlled trial. AJR. 2019;213(5):1029-36.
52. Vass CM, Rigby D, Payne K. Investigating the heterogeneity in women's preferences for breast screening: does the communication of risk matter? Value in Health. 2018;21(2):219-28.
53. Vieira RAdC, Silveira SFdS, Silva DRd, Tramonte MS, de Oliveira-Junior I, Lattari MCT, et al. Knowledge and attitudes about breast cancer care in female inmates in Sao Paulo State/Brazil. The Breast Journal. 2018;24(4):686-7.
54. Warner EL, Martel L, Ou JY, Nam GE, Carbajal-Salisbury S, Fuentes V, et al. A workplace-based intervention to improve awareness, knowledge, and utilization of breast, cervical, and colorectal cancer screenings among Latino service and manual labor employees in Utah. Journal of Community Health. 2019;44(2):256-64.
55. Wegwarth O, Widschwendter M, Cibula D, Sundstrom K, Portuesi R, Lein I, Rebitschek FG. What do European women know about their female cancer risks and cancer screening? A cross-sectional online intervention survey in five European countries. BMJ Open. 2018;8(12):e023789.
56. Weinstein L, LaNoue M, Hurley K, Payton C, Sifri R, Myers R. Feasibility pilot outcomes of a mammography decision support and navigation intervention for women with serious mental illness living in supportive housing settings. Journal of Primary Care & Community Health. 2019;10:2150132719867587.
57. Woodard N, Chen C, Huq MR, He X, Knott CL. Prior health promotion experience and intervention outcomes in a lay health advisor intervention. Health Education Research. 2022;37(4):266-77.
58. Yusuf A, Iskandar YHP, Hadi ISA, Nasution A, Lean Keng S. Breast awareness mobile apps for health education and promotion for breast cancer. Frontiers in Public Health. 2022;10:951641.
59. Champion VL, Paskett ED, Stump TE, Biederman EB, Vachon E, Katz ML, et al. Comparative effectiveness of 2 interventions to increase breast, cervical, and colorectal cancer screening among women in the rural US: a randomized clinical trial. JAMA Network Open. 2023;6(4):e2311004-e.
60. Champion VL, Monahan PO, Stump TE, Biederman EB, Vachon E, Katz ML, et al. The effect of two interventions to increase breast cancer screening in rural women. Cancers. 2022;14(18):N.PAG-N.PAG.
61. Stewart de Ramirez S, McGarvey J, Lotz A, McGee M, Oderwald T, Floess K, et al. Closing the gap: a comparison of engagement interventions to achieve equitable breast cancer screening in rural Illinois. Population Health Management. 2022;25(2):244-53.
62. Darcey E, Hunt EJ, Keogh L, McLean K, Saunders C, Thompson S, et al. Post‐mammographic screening behaviour: a survey investigating what women do after being told they have dense breasts. Health Promotion Journal of Australia. 2021;32:29-39.
63. McDermott AK, McDermott AJ, Osbaldiston R, Lennon RP. Improving breast and cervical cancer screening compliance through direct physician contact in a military treatment facility: a non-randomized pilot study. Military Medicine. 2021;186(5/6):e480-e5.
64. Champion VL, Christy SM, Rakowski W, Lairson DR, Monahan PO, Gathirua-Mwangi WG, et al. An RCT to increase breast and colorectal cancer screening. American Journal of Preventive Medicine. 2020;59(2):e69-e78.
65. Schoenborn NL, Massare J, Park R, Boyd CM, Choi Y, Pollack CE. Assessment of clinician decision-making on cancer screening cessation in older adults with limited life expectancy. JAMA Network Open. 2020;3(6):e206772-e.
66. Costanza ME, Luckmann R, Frisard C, White MJ, Cranos C. Comparing telephone counseling with reminding to promote on-time repeated mammography: a randomized trial in a cohort with 4 years follow-up. Health Education & Behavior. 2020;47(1):37-46.
67. Hsiang EY, Mehta SJ, Small DS, Rareshide CAL, Snider CK, Day SC, Patel MS. Association of an active choice intervention in the electronic health record directed to medical assistants with clinician ordering and patient completion of breast and colorectal cancer screening tests. JAMA Network Open. 2019;2(11):e1915619-e.
68. Jain AG, Guan J, FaisalUddin M, Manoucheri M, Fang C. Improving breast cancer screening rates in a primary care setting. Breast Journal. 2019;25(5):963-6.
69. Freund A, Cohen M, Azaiza F. A culturally tailored intervention for promoting breast cancer screening among women from faith-based communities in Israel: a randomized controlled study. Research on Social Work Practice. 2019;29(4):375-88.
70. Khaliq W, Landis R, Wright SM. Improving breast cancer screening adherence among hospitalized women. Journal of Women's Health (15409996). 2017;26(10):1094-8.
71. Muhrer JC. Improving breast cancer screening in a federally qualified health center with a team of nursing leaders. Nurse Practitioner. 2017;42(1):12-6.
72. Hae-Ra H, Youngshin S, Miyong K, Hedlin HK, Kyounghae K, Hochang Ben L, Roter D. Breast and cervical cancer screening literacy among Korean American women: a community health worker–led intervention. American Journal of Public Health. 2017;107(1):159-65.

Exposure - Not weighing outcomes (N=28):

1. Avci E, Yilmaz M. Educational material for social marketing and behaviours linked to early detection of breast cancer. British Journal of Nursing (Mark Allen Publishing). 2023;32(5):S24-S32.
2. Bensenhaver JM, Perez Martinez AP, Albert PG, Hawley ST, Petersen LF, Newman LA. Trends in mammography use among women aged 40 to 49 years with a history of breast cancer. JAMA Surgery. 2018;153(12):1153-4.
3. Burnside ES, Schrager S, DuBenske L, Keevil J, Little T, Trentham-Dietz A, et al. Team science principles enhance cancer care delivery quality improvement: interdisciplinary implementation of breast cancer screening shared decision making. JCO Oncology Practice. 2023;19(1):e1-e7.
4. Croes KD, Jones NR, DuBenske LL, Schrager SB, Mahoney JE, Little TA, Burnside ES. Core elements of shared decision-making for women considering breast cancer screening: results of a modified Delphi survey. Journal of General Internal Medicine. 2020;35(6):1668-77.
5. Djulbegovic M, Aminawung J, Hoag JR, Kyanko KA, Xu X, Busch SH, Gross CP. Current attitudes and practices around screening mammography among women in the United States: results of a national survey. Journal of General Internal Medicine. 2021;36(6):1802-4.
6. Driedger SM, Annable G, Brouwers M, Turner D, Maier R. Can you un-ring the bell? A qualitative study of how affect influences cancer screening decisions. BMC Cancer. 2017;17(1):647.
7. Eibich P, Goldzahl L. Health information provision, health knowledge and health behaviours: evidence from breast cancer screening. Social Science & Medicine (1982). 2020;265:113505.
8. Froicu M, Mani KL, Coughlin B. Satisfaction with same-day-read baseline mammography. Journal of the American College of Radiology: JACR. 2019;16(3):321-6.
9. Goto Y, Tsugawa K, Furuya Y, Maezato M, Tagami Y, Ogawa Y, et al. Behavior of Japanese women after being informed about the benefits and disadvantages of breast cancer screening: a questionnaire survey. Breast Cancer. 2020;27(4):739-47.
10. Huang H-C, Chang P-C, Li S-F, Wang C-Y, Huang W-T, Chen W, Fan S-Y. The significant predictors for breast, cervical, colorectal, or oral cancer screening intention and behavior in Taiwan. Cancer Nursing. 2022.
11. Ivanova A, Kvalem IL. Psychological predictors of intention and avoidance of attending organized mammography screening in Norway: applying the Extended Parallel Process Model. BMC Women's Health. 2021;21(1):67.
12. Janssen EM, Pollack CE, Boyd C, Bridges JFP, Xue Q-L, Wolff AC, Schoenborn NL. How do older adults consider age, life expectancy, quality of life, and physician recommendations when making cancer screening decisions? Results from a national survey using a discrete choice experiment. Medical Decision Making. 2019;39(6):621-31.
13. Kotwal AA, Walter LC, Lee SJ, Dale W. Are we choosing wisely? Older adults' cancer screening intentions and recalled discussions with physicians about stopping. Journal of General Internal Medicine. 2019;34(8):1538-45.
14. Lang Y, Wu B, Liu X. Economic evaluation of trastuzumab deruxtecan in previously treated HER2-low advanced breast cancer in the United States. Breast Cancer (Dove Medical Press). 2022;14:453-63.
15. Li H, Huang M, Yang Y, Tang J, Ye Y. The practice and willingness of women towards opportunistic screening for breast and cervical cancers in Sichuan province, China: a cross-sectional study. Risk Management and Healthcare Policy. 2023;16:169-83.
16. Lin L, Koh WL, Huang Q, Lee JK. Breast cancer information behaviours and needs among Singapore women: a qualitative study. APJCP. 2021;22(6):1767-74.
17. Liow JJK, Lim ZL, Sim TMY, Ho PJ, Goh S-A, Choy SD, et al. "It Will Lead You to Make Better Decisions about Your Health"-A focus group and survey study on women's attitudes towards risk-based breast cancer screening and personalised risk assessments. Current Oncology. 2022;29(12):9181-98.
18. Ntiri SO, Swanson M, Klyushnenkova EN. Text messaging as a communication modality to promote screening mammography in low-income African American women. Journal of Medical Systems. 2022;46(5):28.
19. Percefull J, Butler J. Improving mammography through effective screening, brief intervention, and referral to treatment at a rural health center. Journal of the American Association of Nurse Practitioners. 2020;33(4):324-30.
20. Petrocchi S, Ludolph R, Labrie NHM, Schulz P. Application of the theory of regulatory fit to promote adherence to evidence-based breast cancer screening recommendations: experimental versus longitudinal evidence. BMJ Open. 2020;10(11):e037748.
21. Salinas-Martinez AM, Castaneda-Vasquez DE, Garcia-Morales NG, Oliva-Sosa NE, de-la-Garza-Salinas LH, Nunez-Rocha GM, Ramirez-Aranda JM. Stages of change for mammography among Mexican women and a decisional balance comparison across countries. Journal of Cancer Education. 2018;33(6):1230-8.
22. Schapira MM, Hubbard RA, Seitz HH, Conant EF, Schnall M, Cappella JN, et al. The impact of a risk-based breast cancer screening decision aid on initiation of mammography among younger women: report of a randomized trial. MDM Policy & Practice. 2019;4(1):2381468318812889.
23. Shi W, Nagler RH, Fowler EF, Gollust SE. Predictors of women's awareness of the benefits and harms of mammography screening and associations with confusion, ambivalence, and information seeking. Health Communication. 2021;36(3):303-14.
24. Yurt S, Saglam Aksut R, Kadioglu H. The effect of peer education on health beliefs about breast cancer screening. International Nursing Review. 2019;66(4):498-505.
25. Augestad LA, Rand K, Luo N, Barra M. Using the choice sequence in time trade-off as discrete choices: do the two stories match? Value in Health. 2020;23(4):487-94.
26. Pullenayegum EM, Pickard AS, Xie F. Latent class models reveal poor agreement between discrete-choice and time tradeoff preferences. Medical Decision Making. 2019;39(4):421-36.
27. Okuhara T, Ishikawa H, Goto E, Okada M, Kato M, Kiuchi T. Processing fluency effect of a leaflet for breast and cervical cancer screening: a randomized controlled study in Japan. Psychology, Health & Medicine. 2018;23(10):1250-60.
28. Tsorng-Yeh L, Grace H, Pilkington FB. The effect of an educational program on breast cancer screening knowledge and attitude among Chinese immigrant women in Toronto: a pilot study. Annual Worldwide Nursing Conference. 2018:240-4.

Language (N=3):

1. Perea AH, Rosselli D. Immediate versus delayed breast reconstruction in breast cancer patients in Colombia: A costutility analysis. Biomedica: revista del Instituto Nacional de Salud. 2018;38(3):363-78.
2. Filippou F, Rokka S, Sivvas G, Pitsi A, Bebetsos E, Koupani A, et al. Greek traditional dance teaching programs: effects on the quality of life of women who survived breast cancer. Nosileftiki. 2020;59(1):78-85.
3. Chyi L, Fang-Zi L, Shu-Ling C. Symptom experiences of breast cancer patients. Journal of Nursing. 2017;64(2):19-27.

Not primary research (N=20):

1. Agarwal S, Chang DW. Breast reconstruction in the patient with stable, metastatic breast cancer. The Breast Journal. 2020;26(2):335-6.
2. Almeida GRd, Silvinato A, Bernardo WM. Systemic treatment and surgery versus systemic treatment alone for metastatic breast cancer. Revista da Associacao Medica Brasileira. 2020;66(6):710-9.
3. AlSaleh KA. Efficacy of breast cancer screening program in Kingdom of Saudi Arabia. Saudi Medical Journal. 2022;43(4):428-30.
4. Anonymous. Mammogram screening for breast cancer. American Family Physician. 2021;103(1):Online.
5. Bartholomew T, Colleoni M, Schmidt H. Financial incentives for breast cancer screening undermine informed choice. BMJ (Clinical research ed). 2022;376:e065726.
6. Byers L, Michell K, McCullough K. Awareness, acceptability and access to screening mammography for remote Aboriginal women. Health Promotion Journal of Australia. 2018;29(3):366-7.
7. Grimm LJ, Avery CS, Hendrick E, Baker JA. Benefits and risks of mammography screening in women ages 40 to 49 years. Journal of Primary Care & Community Health. 2022;13:21501327211058322.
8. Howie LJ, Singh H, Bloomquist E, Wedam S, Amiri-Kordestani L, Tang S, et al. Outcomes of older women with hormone receptor-positive, human epidermal growth factor receptor-negative metastatic breast cancer treated with a CDK4/6 inhibitor and an aromatase inhibitor: an FDA pooled analysis. Journal of Clinical Oncology. 2019;37(36):3475-83.
9. Keating NL, Pace LE. Breast Cancer Screening in 2018: time for shared decision making. JAMA. 2018;319(17):1814-5.
10. Kouwenberg CAE, Mureau MAM, Kranenburg LW, Rakhorst H, de Leeuw D, Klem TMAL, et al. Cost-utility analysis of four common surgical treatment pathways for breast cancer. European Journal of Surgical Oncology. 2021;47(6):1299-308.
11. Marsh RM, Silosky MS. The potential cost of patient satisfaction: a mammography experience. JACR. 2018;15(5):776-7.
12. Perez LG, Elder JP, Haughton J, Martinez ME, Arredondo EM. Erratum to: Socio-demographic moderators of associations between psychological factors and Latinas’ breast cancer screening behaviors. Journal of Immigrant and Minority Health. 2018;20(4):831.
13. Roberto A, Colombo C, Candiani G, Satolli R, Giordano L, Jaramillo L, et al. Correction: A dynamic web-based decision aid to improve informed choice in organised breast cancer screening. A pragmatic randomized trial in Italy. British Journal of Cancer. 2021;125(1):146-7.
14. Stellato D, Thabane ME, Park J, Chandiwana D, Delea TE. Cost Effectiveness of ribociclib in combination with fulvestrant for the treatment of postmenopausal women with HR+/HER2- advanced breast cancer who have received no or only one prior line of endocrine therapy: a Canadian healthcare perspective. PharmacoEconomics. 2021;39(9):1045-58.
15. Su TT, Donnelly M. Improving breast and colorectal cancer screening uptake in Malaysia. European Journal of Cancer Care. 2022;31(5):e13593.
16. Takumoto Y, Shiroiwa T, Shimozuma K, Iwata H, Takahashi M, Baba S, et al. Cost-effectiveness of trastuzumab with or without chemotherapy as adjuvant therapy in HER2-positive elderly breast cancer patients: a randomized, open-label clinical trial, the RESPECT trial. Clinical Drug Investigation. 2022;42(3):253-62.
17. Zahl P-H, Kalager M, Suhrke P, Nord E. Quality-of-life effects of screening mammography in Norway. International Journal of Cancer. 2020;146(8):2104-12.
18. Giorgi Rossi P. What a woman needs to know about overdiagnosis to decide about breast cancer screening. European Journal of Cancer. 2022;173:327-8.
19. Oluwasanjo A. Vital organs: honoring a patient's bodily autonomy and decision-making. ASCO Connection. 2018:34-6.
20. Wrigley A. Breast cancer screening - an informed choice? Journal of Holistic Healthcare. 2017;14(2):8-13.

Population - Age or sex (N=6):

1. Blankensteijn LL, Egeler SA, Sinno HH, Ibrahim AMS, Izadpanah A, Vorstenbosch J, et al. Analysis of utility assessment scores to objectify the health burden caused by breast conservation therapy. Plastic Surgery (Oakville, Ont). 2020;28(2):77-82.
2. Eber MR, Sunstein CR, Hammitt JK, Yeh JM. The modest effects of fact boxes on cancer screening. Journal of Risk and Uncertainty. 2021;62(1):29-54.
3. Grayek E, Yang Y, Fischhoff B, Schifferdecker KE, Woloshin S, Kerlikowske K, et al. A procedure for eliciting women's preferences for breast cancer screening frequency. Medical Decision Making. 2022;42(6):783-94.
4. Jensen MD, Hansen KM, Siersma V, Brodersen J. Using a deliberative poll on breast cancer screening to assess and improve the decision quality of laypeople. PloS One. 2021;16(10):e0258869.
5. Kim J, Paik Y, Park S. Cancer screening program delivered by community health workers for Chinese married immigrant women in Korea. International Journal of Environmental Research and Public Health. 2022;19(11).
6. Kim S-H, Jo M-W, Ock M, Lee H-J, Lee J-W. Estimation of health state utilities in breast cancer. Patient Preference and Adherence. 2017;11:531-6.

Population - All non-operable for HSUV studies (N=36):

1. Ameri H, Safari H, Poder T. Exploring the consistency of the SF-6Dv2 in a breast cancer population. Expert Review of Pharmacoeconomics & Outcomes Research. 2021;21(5):1017-24.
2. Bahl A, Wilson W, Ball J, Renninson E, Dubey S, Bravo A, et al. Concept: a randomised multicentre trial of first line chemotherapy comparing three weekly cabazitaxel versus weekly paclitaxel in HER2 negative metastatic breast cancer. Breast. 2022;66:69-76.
3. Brusniak K, Feisst M, Sebesteny L, Hartkopf A, Graf J, Engler T, et al. Measuring the time to deterioration for health-related quality of life in patients with metastatic breast cancer using a web-based monitoring application: longitudinal cohort study. JMIR Cancer. 2021;7(4):e25776.
4. Chin S, Cavadino A, Akroyd A, Tennant G, Dobson R, Gautier A, Reynolds L. An investigation of virtual reality nature experiences in patients with metastatic breast cancer: secondary analysis of a randomized controlled trial. JMIR Cancer. 2022;8(3):e38300.
5. Chou T-C, Chiang S-C, Ko Y. Health state utilities for metastatic breast cancer in Taiwan. Breast. 2020;51:57-64.
6. Claessens AKM, Ramaekers BLT, Lobbezoo DJA, van Kampen RJW, de Boer M, van de Wouw AJ, et al. Quality of life in a real-world cohort of advanced breast cancer patients: a study of the SONABRE Registry. Quality of Life Research. 2020;29(12):3363-74.
7. Curigliano G, Dunton K, Rosenlund M, Janek M, Cathcart J, Liu Y, et al. Patient-reported outcomes and hospitalization data in patients with HER2-positive metastatic breast cancer receiving trastuzumab deruxtecan or trastuzumab emtansine in the phase III DESTINY-Breast03 study. Annals of Oncology. 2023.
8. Deutsch TM, Pfob A, Brusniak K, Riedel F, Bauer A, Dijkstra T, et al. Machine learning and patient-reported outcomes for longitudinal monitoring of disease progression in metastatic breast cancer: a multicenter, retrospective analysis. European Journal of Cancer. 2023;188:111-21.
9. Hagiwara Y, Shiroiwa T, Shimozuma K, Kawahara T, Uemura Y, Watanabe T, et al. Impact of adverse events on health utility and health-related quality of life in patients receiving first-line chemotherapy for metastatic breast cancer: results from the SELECT BC study. PharmacoEconomics. 2018;36(2):215-23.
10. Hara H, Sakai Y, Kawamoto T, Fukase N, Kawakami Y, Takemori T, et al. Surgical outcomes of metastatic bone tumors in the extremities (Surgical outcomes of bone metastases). Journal of Bone Oncology. 2021;27:100352.
11. Hernandez Alava M, Wailoo A, Pudney S, Gray L, Manca A. Mapping clinical outcomes to generic preference-based outcome measures: development and comparison of methods. Health Technology Assessment. 2020;24(34):1-68.
12. Huang M, O'Shaughnessy J, Haiderali A, Pan W, Hu P, Chaudhuri M, et al. Q-TWiST analysis of pembrolizumab combined with chemotherapy as first-line treatment of metastatic triple-negative breast cancer that expresses PD-L1. European Journal of Cancer. 2022;177:45-52.
13. Im S-A, Mukai H, Park IH, Masuda N, Shimizu C, Kim S-B, et al. Palbociclib plus letrozole as first-line therapy in postmenopausal Asian women with metastatic breast cancer: results from the phase III, randomized PALOMA-2 study. Journal of Global Oncology. 2019;5:1-19.
14. Iwatani T, Inoue E, Tsugawa K. Validation of the predictive accuracy of health-state utility values based on the Lloyd model for metastatic or recurrent breast cancer in Japan. BMJ Open. 2021;11(12):e046273.
15. Kahan Z, Gil-Gil M, Ruiz-Borrego M, Carrasco E, Ciruelos E, Munoz M, et al. Health-related quality of life with palbociclib plus endocrine therapy versus capecitabine in postmenopausal patients with hormone receptor-positive metastatic breast cancer: patient-reported outcomes in the PEARL study. European Journal of Cancer. 2021;156:70-82.
16. Lambert-Obry V, Gouault-Laliberte A, Castonguay A, Zanotti G, Tran T, Mates M, et al. Real-world patient- and caregiver-reported outcomes in advanced breast cancer. Current Oncology. 2018;25(4):e282-e90.
17. Leo S, Arnoldi E, Repetto L, Coccorullo Z, Cinieri S, Fedele P, et al. Eribulin mesylate as third or subsequent line chemotherapy for elderly patients with locally recurrent or metastatic breast cancer: a multicentric observational study of GIOGer (Italian Group of Geriatric Oncology)-ERIBE. The Oncologist. 2019;24(6):e232-e40.
18. Maximiano C, Lopez I, Martin C, Zugazabeitia L, Marti-Ciriquian JL, Nunez MA, et al. An exploratory, large-scale study of pain and quality of life outcomes in cancer patients with moderate or severe pain, and variables predicting improvement. PloS One. 2018;13(4):e0193233.
19. Mendoza TR, Dueck AC, Shi Q, Ma H, Zhang J, Qian Y, Cleeland CS. The contribution of pain in determining the health status of cancer patients with bone metastases: a secondary analysis of data from three Phase III registration trials. European Journal of Pain. 2018;22(3):565-71.
20. Mueller V, Wardley A, Paplomata E, Hamilton E, Zelnak A, Fehrenbacher L, et al. Preservation of quality of life in patients with human epidermal growth factor receptor 2-positive metastatic breast cancer treated with tucatinib or placebo when added to trastuzumab and capecitabine (HER2CLIMB trial). European Journal of Cancer. 2021;153:223-33.
21. Nater A, Chuang J, Liu K, Quraishi NA, Pasku D, Wilson JR, Fehlings MG. A personalized medicine approach for the management of spinal metastases with cord compression: development of a novel clinical prediction model for postoperative survival and quality of life. World Neurosurgery. 2020;140:654-63.e13.
22. Nater A, Tetreault LA, Kopjar B, Arnold PM, Dekutoski MB, Finkelstein JA, et al. Predictive factors of survival in a surgical series of metastatic epidural spinal cord compression and complete external validation of 8 multivariate models of survival in a prospective North American multicenter study. Cancer. 2018;124(17):3536-50.
23. Ogorevc M, Murovec N, Fernandez NB, Rupel VP. Questioning the differences between general public vs. patient based preferences towards EQ-5D-5L defined hypothetical health states. Health Policy. 2019;123(2):166-72.
24. Rugo HS, Dieras V, Gelmon KA, Finn RS, Slamon DJ, Martin M, et al. Impact of palbociclib plus letrozole on patient-reported health-related quality of life: results from the PALOMA-2 trial. Annals of Oncology. 2018;29(4):888-94.
25. Shiroiwa T, Fukuda T, Shimozuma K, Mouri M, Hagiwara Y, Kawahara T, et al. Cost-effectiveness analysis of the introduction of S-1 therapy for first-line metastatic breast cancer treatment in Japan: results from the randomized phase III SELECT BC trial. BMC Cancer. 2017;17(1):773.
26. Shiroiwa T, Fukuda T, Shimozuma K, Mouri M, Hagiwara Y, Doihara H, et al. Long-term health status as measured by EQ-5D among patients with metastatic breast cancer: comparison of first-line oral S-1 and taxane therapies in the randomized phase III SELECT BC trial. Quality of life research. 2017;26(2):445-53.
27. Smith JS, Line B, Bess S, Shaffrey CI, Kim HJ, Mundis G, et al. The health impact of adult cervical deformity in patients presenting for surgical treatment: comparison to united states population norms and chronic disease states based on the EuroQuol-5 dimensions questionnaire. Neurosurgery. 2017;80(5):716-25.
28. Taira N, Kashiwabara K, Tsurutani J, Kitada M, Takahashi M, Kato H, et al. Quality of life in a randomized phase II study to determine the optimal dose of 3-week cycle nab-paclitaxel in patients with metastatic breast cancer. Breast Cancer. 2022;29(1):131-43.
29. Todo M, Ueda S, Osaki S, Sugitani I, Takahashi T, Takahashi M, et al. Improvement of treatment outcomes after implementation of comprehensive pharmaceutical care in breast cancer patients receiving everolimus and exemestane. Die Pharmazie. 2018;73(2):110-4.
30. Wallwiener M, Heindl F, Brucker SY, Taran F-A, Hartkopf A, Overkamp F, et al. Implementation and feasibility of electronic Patient-Reported Outcome (ePRO) data entry in the PRAEGNANT real-time advanced and metastatic breast cancer registry. Geburtshilfe und Frauenheilkunde. 2017;77(8):870-8.
31. Westermann L, Eysel P, Luge H, Olivier A, Oikonomidis S, Baschera D, Zarghooni K. Quality of life and functional outcomes after surgery for spinal metastases: results of a cohort study. Technology and Health Care. 2020;28(3):303-14.
32. Wood R, Mitra D, de Courcy J, Iyer S. Patient-reported pain severity, pain interference and health status in HR+/HER2- advanced/metastatic breast cancer. ESMO Open. 2017;2(3):e000227.
33. Rugo HS, Umanzor GA, Barrios FJ, Vasallo RH, Chivalan MA, Bejarano S, et al. Open-label, randomized, multicenter, phase III study comparing oral paclitaxel plus encequidar versus intravenous paclitaxel in patients with metastatic breast cancer. Journal of Clinical Oncology. 2023;41(1):65-74.
34. Nalley C. Phase III ASCENT Study: Health-related quality of life in TNBC patients. Oncology Times. 2021;43(22):21-.
35. Kahan Z, Gil-Gil M, Ruiz-Borrego M, Carrasco E, Ciruelos E, Muñoz M, et al. Health-related quality of life with palbociclib plus endocrine therapy versus capecitabine in postmenopausal patients with hormone receptor–positive metastatic breast cancer: patient-reported outcomes in the PEARL study. European Journal of Cancer. 2021;156:70-82.
36. Mueller V, Wardley A, Paplomata E, Hamilton E, Zelnak A, Fehrenbacher L, et al. Preservation of quality of life in patients with human epidermal growth factor receptor 2–positive metastatic breast cancer treated with tucatinib or placebo when added to trastuzumab and capecitabine (HER2CLIMB trial). European Journal of Cancer. 2021;153:223-33.

Population - Only cancer patients for non-HSUV studies (N=3):

1. Battisti NML, Reed MWR, Herbert E, Morgan JL, Collins KA, Ward SE, et al. Bridging the Age Gap in breast cancer: impact of chemotherapy on quality of life in older women with early breast cancer. European Journal of Cancer. 2021;144:269-80.
2. Freedman RA, Revette AC, Gagnon H, Perilla-Glen A, Kokoski M, Hussein SO, et al. Acceptability of a companion patient guide to support expert consensus guidelines on surveillance mammography in older breast cancer survivors. Breast Cancer Research and Treatment. 2022;195(2):141-52.
3. Parkinson B, Sherman KA, Brown P, Shaw L-KE, Boyages J, Cameron LD, et al. Cost-effectiveness of the BRECONDA decision aid for women with breast cancer: results from a randomized controlled trial. Psycho-Oncology. 2018;27(6):1589-96.

Population - Other (N=46):

1. Absolom K, Warrington L, Hudson E, Hewison J, Morris C, Holch P, et al. Phase III randomized controlled trial of eRAPID: eHealth intervention during chemotherapy. Journal of Clinical Oncology. 2021;39(7):734-47.
2. Asensio-Garcia MDR, Tomas-Rodriguez MI, Palazon-Bru A, Hernandez-Sanchez S, Nouni-Garcia R, Romero-Aledo AL, Gil-Guillen VF. Effect of rowing on mobility, functionality, and quality of life in women with and without breast cancer: a 4-month intervention. Supportive Care in Cancer. 2021;29(5):2639-44.
3. Brady LA, Tumiel-Berhalter LM, Schad LA, Bentham A, Vitale K, Norton A, et al. Increasing breast, cervical, and colorectal cancer screenings: a qualitative assessment of barriers and promoters in safety-net practices. Journal of Patient-Centered Research and Reviews. 2021;8(4):323-30.
4. Bruce J, Mazuquin B, Mistry P, Rees S, Canaway A, Hossain A, et al. Exercise to prevent shoulder problems after breast cancer surgery: the PROSPER RCT. Health Technology Assessment. 2022;26(15):1-124.
5. Chan A, Ruiz-Borrego M, Marx G, Chien AJ, Rugo HS, Brufsky A, et al. Final findings from the CONTROL trial: strategies to reduce the incidence and severity of neratinib-associated diarrhea in patients with HER2-positive early-stage breast cancer. Breast. 2023;67:94-101.
6. Chang Y-C, Lin G-M, Yeh T-L, Chang Y-M, Yang C-H, Lo C, et al. Impact of mindfulness-based stress reduction on female sexual function and mental health in patients with breast cancer. Supportive Care in Cancer. 2022;30(5):4315-25.
7. Che Bakri NA, Kwasnicki RM, Dhillon K, Khan N, Ghandour O, Cairns A, et al. Objective assessment of postoperative morbidity after breast cancer treatments with wearable activity monitors: the "BRACELET" study. Annals of Surgical Oncology. 2021;28(10):5597-609.
8. Chu C, Yoder J, Smolkin M, Hollen PJ, Dengel LT. A decision aid for patients with minimally suspicious screening mammograms: a pilot study. Oncology Nursing Forum. 2022;49(5):471-9.
9. Clarijs ME, Oemrawsingh A, Broker MEE, Verhoef C, Lingsma H, Koppert LB. Quality of life of caregivers of breast cancer patients: a cross-sectional evaluation. Health and Quality of Life Outcomes. 2022;20(1):29.
10. Ding H, Mao A, Lin J, Wong MCS, Dong P, Qiu W. Using a Chinese time trade-off approach to explore the health utility level and quality of life of cancer patients in urban China: a multicentre cross-sectional study. Supportive Care in Cancer. 2021;29(4):2215-23.
11. Efthymiadou O, Mossman J, Kanavos P. Health related quality of life aspects not captured by EQ-5D-5L: Results from an international survey of patients. Health Policy. 2019;123(2):159-65.
12. Efthymiadou O, Mossman J, Kanavos P. Differentiation of health-related quality of life outcomes between five disease areas: results from an international survey of patients. International Journal of Technology Assessment in Health Care. 2018;34(5):498-506.
13. Enblom A, Lindquist H, Bergmark K. Participation in water-exercising long-term after breast cancer surgery: experiences of significant factors for continuing exercising as a part of cancer rehabilitation. European Journal of Cancer Care. 2018;27(1).
14. Esplen MJ, Leszcz M, Hunter J, Wong J, Leung YW, Toner B, et al. A randomized controlled trial of a supportive expressive group intervention for women with a family history of breast cancer. Psycho-Oncology. 2018;27(11):2645-53.
15. Filipe MD, Simons JM, Moeliker L, Waaijer L, Vriens MR, van Diest PJ, Witkamp AJ. Patient-reported outcomes of ductoscopy procedures for pathologic nipple discharge. Breast Cancer. 2021;28(2):471-7.
16. Georgiou IT, Pappa ED, Coclami TE, Kelessis NG. Stereotactic vs open biopsy for non-palpable breast lesions. Their impact on short term quality of life. Psychology, Health & Medicine. 2023;28(5):1126-32.
17. Hallberg H, Elander A, Kolby L, Hansson E. A biological or a synthetic mesh in immediate breast reconstruction? A cohort-study of long-term Health related Quality of Life (HrQoL). European Journal of Surgical Oncology. 2019;45(10):1812-6.
18. Harbeck N, Franke F, Villanueva-Vazquez R, Lu Y-S, Tripathy D, Chow L, et al. Health-related quality of life in premenopausal women with hormone-receptor-positive, HER2-negative advanced breast cancer treated with ribociclib plus endocrine therapy: results from a phase III randomized clinical trial (MONALEESA-7). Therapeutic Advances in Medical Oncology. 2020;12:1758835920943065.
19. Ichimura T, Nomura H, Shimizu H. Community pharmacists' measurement of health-related quality of life for breast cancer with positive hormone receptors: a prospective observational study. SAGE Open Medicine. 2023;11:20503121231164491.
20. Jang CE, Jung MS, Sohn EH, Kim M, Yoo H-S, Bae K, et al. The evaluation of changes in peripheral neuropathy and quality-of-life using low-frequency electrostimulation in patients treated with chemotherapy for breast cancer: A study protocol. Trials. 2018;19(1):526.
21. Kleijburg A, Braal CL, Westenberg JD, Jager A, Koolen SLW, Mathijssen RHJ, et al. Health-related quality of life and productivity costs in breast cancer patients treated with tamoxifen in the Netherlands. Biomedicine & Pharmacotherapy. 2023;158:114158.
22. Koskinen J-P, Farkkila N, Sintonen H, Saarto T, Taari K, Roine RP. The association of financial difficulties and out-of-pocket payments with health-related quality of life among breast, prostate and colorectal cancer patients. Acta Oncologica. 2019;58(7):1062-8.
23. Loeffler S, Poehlmann K, Hornemann B. Finding meaning in suffering? Meaning making and psychological adjustment over the course of a breast cancer disease. European Journal of Cancer Care. 2018;27(3):e12841.
24. Lux MP, Lewis K, Rider A, Niyazov A. Treatment patterns, safety, and patient reported outcomes among adult women with human epidermal growth factor receptor 2-negative advanced breast cancer with or without, or with unknown, BRCA1/2 mutation(s): results of a Real-world study from the United States, United Kingdom, and four EU countries. Breast Care. 2022;17(5):460-9.
25. Mahal AR, Cramer LD, Wang EH, Wang S, Davidoff AJ, Gross CP, Yu JB. Did quality of life for older cancer survivors improve with the turn of the century in the United States? Journal of Geriatric Oncology. 2021;12(1):102-5.
26. Mahtani R, Niyazov A, Arondekar B, Lewis K, Rider A, Massey L, Lux MP. Real-world patient-reported outcomes and physician satisfaction with poly (ADP-ribose) polymerase inhibitors versus chemotherapy in patients with germline BRCA1/2-mutated human epidermal growth factor receptor 2-negative advanced breast cancer from the United States, Europe, and Israel. BMC Cancer. 2022;22(1):1343.
27. Nappi RE, Palacios S, Bruyniks N, Particco M, Panay N. The European Vulvovaginal Epidemiological Survey (EVES). Impact of history of breast cancer on prevalence, symptoms, sexual function and quality of life related to vulvovaginal atrophy. Gynecological Endocrinology. 2021;37(1):78-82.
28. Negenborn VL, Smit JM, Dikmans REG, Winters HAH, Twisk JWR, Ruhe PQ, et al. Short-term cost-effectiveness of one-stage implant-based breast reconstruction with an acellular dermal matrix versus two-stage expander-implant reconstruction from a multicentre randomized clinical trial. The British Journal of Surgery. 2019;106(5):586-95.
29. Ohno S, Chen Y, Sakamaki H, Matsumaru N, Tsukamoto K. A population-based study of the humanistic burden among cancer patients in Japan. Journal of Medical Economics. 2020;23(5):429-41.
30. Ou H-T, Chung W-P, Su P-F, Lin T-H, Lin J-Y, Wen Y-C, Fang W-T. Health-related quality of life associated with different cancer treatments in Chinese breast cancer survivors in Taiwan. European Journal of Cancer Care. 2019;28(4):e13069.
31. Rainey L, van der Waal D, Broeders MJM. Dutch women's intended participation in a risk-based breast cancer screening and prevention programme: a survey study identifying preferences, facilitators and barriers. BMC Cancer. 2020;20(1):965.
32. Rim CH, Ahn S-J, Kim JH, Yoon WS, Chun M, Yang DS, et al. An assessment of quality of life for early phase after adjuvant radiotherapy in breast cancer survivors: a Korean multicenter survey (KROG 14-09). Health and Quality of Life Outcomes. 2017;15(1):96.
33. Salo JTK, Repo JP, Roine RP, Sintonen H, Tukiainen EJ. Health-related quality of life after oncological resection and reconstruction of the chest wall. JPRAS. 2019;72(11):1776-84.
34. Schumacher JR, Stankowski-Drengler TJ, Tucholka JL, Poore SO, Wilke LG, Greenberg CC, Neuman HB. Utility of health services regions in examining socioeconomic disparities in receipt of breast reconstruction. The Breast Journal. 2020;26(9):1895-7.
35. Seferina SC, Ramaekers BLT, de Boer M, Dercksen MW, van den Berkmortel F, van Kampen RJW, et al. Cost and cost-effectiveness of adjuvant trastuzumab in the real world setting: a study of the Southeast Netherlands Breast Cancer Consortium. Oncotarget. 2017;8(45):79223-33.
36. Seguiti C, Salvo PF, Di Stasio E, Lamonica S, Fedele AL, Manfrida S, et al. Health-related quality of life (HRQoL) from HIV patients' perspective: comparison of patient-reported outcome (PRO) measures among people living with HIV (PLWH) and other chronic clinical conditions. Journal of Patient-Reported Outcomes. 2022;6(1):27.
37. Siebers CCN, Appelman L, van Oirsouw MCJ, Appelman PTM, Go S, Mann RM. the effect of targeted ultrasound as primary imaging modality on quality of life in women with focal breast complaints: a comparative cohort study. Journal of Women's Health. 2023;32(1):71-7.
38. Tanaka K, Tachi T, Hori A, Osawa T, Nagaya K, Makino T, et al. Cost utility analysis of pharmacist counseling care for breast cancer chemotherapy outpatients. Die Pharmazie. 2019;74(7):439-42.
39. Tripp L, Abelson J. Supporting women at average risk to make informed decisions about mammography when there is no "right" answer: a qualitative citizen deliberation study. CMAJ Open. 2019;7(4):E730-E7.
40. van den Broek JJ, Schechter CB, van Ravesteyn NT, Janssens ACJW, Wolfson MC, Trentham-Dietz A, et al. Personalizing breast cancer screening based on polygenic risk and family history. Journal of the National Cancer Institute. 2021;113(4):434-42.
41. Verrill M, Wardley AM, Retzler J, Smith AB, Bottomley C, Ni Dhochartaigh S, et al. Health-related quality of life and work productivity in UK patients with HER2-positive breast cancer: a cross-sectional study evaluating the relationships between disease and treatment stage. Health and Quality of Life Outcomes. 2020;18(1):353.
42. Williams AE, Rapport F, Russell IT, Hutchings HA. Psychometric development of the Upper Limb Lymphedema Quality of Life Questionnaire demonstrated the patient-reported outcome measure to be a robust measure for breast cancer-related lymphedema. Journal of Clinical Epidemiology. 2018;100:61-70.
43. Ax A-K, Husberg M, Johansson B, Demmelmaier I, Berntsen S, Sjövall K, et al. Cost-effectiveness of different exercise intensities during oncological treatment in the Phys-Can RCT. Acta Oncologica. 2023;62(4):414-21.
44. Stewart E, Tavabie S, White N, Appleyard S, Bass S, Gilbert D, et al. A short report examining the introduction of routine use of patient-reported outcome measures in a mixed oncology population. Clinical Oncology. 2022;34(4):241-6.
45. Karin MR, He H, Posternak VR, Bowler A, Nguyen D. Prevention of postoperative lymphedema for breast cancer surgery with axillary lymph node dissection combined with lympha and lymphatic preservation: patient selection and outcomes. Journal of the American College of Surgeons. 2021;233(5):e17-e8.
46. Myoung-Hee K. Factors affecting health-related quality of life for subjects with breast cancer. Medico-Legal Update. 2020;20(1):2258-64.

Population - Timing (N=18):

1. Abu Farha NH, Khatib MT, Salameh H, Zyoud SeH. Cancer-related post-treatment pain and its impact on health-related quality of life in breast cancer patients: a cross sectional study in Palestine. Asia Pacific Family Medicine. 2017;16:7.
2. Advani PG, Lei X, Swanick CW, Xu Y, Shen Y, Goodwin NA, et al. Local therapy decisional regret in older women with breast cancer: a population-based study. International Journal of Radiation Oncology, Biology, Physics. 2019;104(2):383-91.
3. Ahn S-J, Kim JH, Chun M, Yoon WS, Rim CH, Yang DS, et al. Physical activity status in relation to quality of life and dietary habits in breast cancer survivors: subset analyses of KROG 14-09 nationwide questionnaire study. Quality of Life Research. 2020;29(12):3353-61.
4. Chae BJ, Lee J, Lee SK, Shin H-J, Jung S-Y, Lee JW, et al. Unmet needs and related factors of Korean breast cancer survivors: a multicenter, cross-sectional study. BMC Cancer. 2019;19(1):839.
5. Galactionova K, Loibl S, Salari P, Marme F, Martin M, Untch M, et al. Cost-effectiveness of palbociclib in early breast cancer patients with a high risk of relapse: results from the PENELOPE-B trial. Frontiers in Oncology. 2022;12:886831.
6. Garrison LP, Jr., Babigumira J, Tournier C, Goertz H-P, Lubinga SJ, Perez EA. Cost-effectiveness analysis of pertuzumab with trastuzumab and chemotherapy compared to trastuzumab and chemotherapy in the adjuvant treatment of her2-positive breast cancer in the United States. Value in Health. 2019;22(4):408-15.
7. Huang M, A Fasching P, Haiderali A, Xue W, Yang C, Pan W, et al. Cost-effectiveness of neoadjuvant pembrolizumab plus chemotherapy followed by adjuvant single-agent pembrolizumab for high-risk early-stage triple-negative breast cancer in the United States. Advances in Therapy. 2023;40(3):1153-70.
8. Iwatani T, Noto S, Tsugawa K. Changes in health state utility values in Japanese Patients with end-stage breast cancer. Current Oncology. 2021;28(5):4203-12.
9. Kim J, Jo M-W, Lee H-J, Ahn S-H, Son BH, Lee JW, Lee SB. Validity and reliability of the Health-Related Quality of Life Instrument with 8 Items (HINT-8) in Korean breast cancer patients. Osong Public Health and Research Perspectives. 2021;12(4):254-63.
10. Konara Mudiyanselage SP, Wu Y-L, Kukreti S, Chen C-C, Lin C-N, Tsai Y-T, et al. Dynamic changes in quality of life, psychological status, and body image in women who underwent a mastectomy as compared with breast reconstruction: an 8-year follow up. Breast Cancer. 2023;30(2):226-40.
11. Kouwenberg CAE, Kranenburg LW, Visser MS, Busschbach JJ, Mureau MAM. The validity of the EQ-5D-5L in measuring quality of life benefits of breast reconstruction. JPRAS. 2019;72(1):52-61.
12. Lee CF, Ng R, Luo N, Cheung YB. Development of conversion functions mapping the FACT-B total score to the EQ-5D-5L utility value by three linking methods and comparison with the ordinary least square method. Applied Health Economics and Health Policy. 2018;16(5):685-95.
13. Peck SS, Esmaeilzadeh M, Rankin K, Shalmon T, Fan C-PS, Somerset E, et al. Self-reported physical activity, QoL, cardiac function, and cardiorespiratory fitness in women with HER2+ breast cancer. JACC CardioOncology. 2022;4(3):387-400.
14. Rosenberg SM, O'Neill A, Sepucha K, Miller KD, Dang CT, Northfelt DW, et al. Quality of life following receipt of adjuvant chemotherapy with and without bevacizumab in patients with lymph node-positive and high-risk lymph node-negative breast cancer. JAMA Network Open. 2022;5(2):e220254.
15. Thorarinsson A, Frojd V, Kolby L, Ljungdal J, Taft C, Mark H. Long-term health-related quality of life after breast reconstruction: comparing 4 different methods of reconstruction. Plastic and Reconstructive Surgery Global Open. 2017;5(6):e1316.
16. Yu J, Son W-S, Lee SB, Chung IY, Son BH, Ahn SH, et al. Uneven recovery patterns of compromised health-related quality of life (EQ-5D-3 L) domains for breast Cancer survivors: a comparative study. Health and Quality of Life Outcomes. 2018;16(1):143.
17. Yusoff J, Ismail A, Abd Manaf MR, Ismail F, Abdullah N, Muhammad R, et al. Quality of life of women with breast cancer in a tertiary referral university hospital. Health and Quality of Life Outcomes. 2022;20(1):15.
18. Ou HT, Chung WP, Su PF, Lin TH, Lin JY, Wen YC, Fang WT. Health‐related quality of life associated with different cancer treatments in Chinese breast cancer survivors in Taiwan. European Journal of Cancer Care. 2019;28(4):N.PAG-N.PAG.

Protocol (N=23):

1. Armin JS, Williamson HJ, Rothers J, Lee MS, Baldwin JA. An adapted cancer screening education program for native american women with intellectual and developmental disabilities and their caregivers: protocol for feasibility and acceptability testing. JMIR Research Protocols. 2023;12:e37801.
2. Baek SY, Lee SB, Lee Y, Chung S, Choi C-M, Lee HJ, et al. Effects of mobile healthcare applications on the lifestyle of patients with breast cancer: a protocol for a randomized clinical trial. Journal of Breast Cancer. 2022;25(5):425-35.
3. Carles M, Martinez-Alonso M, Pons A, Perez-Lacasta MJ, Perestelo-Perez L, Sala M, et al. The effect of information about the benefits and harms of mammography on women's decision-making: study protocol for a randomized controlled trial. Trials. 2017;18(1):426.
4. Catsman CJLM, Beek MA, Voogd AC, Mulder PGH, Luiten EJT. The COSMAM TRIAL a prospective cohort study of quality of life and cosmetic outcome in patients undergoing breast conserving surgery. BMC Cancer. 2018;18(1):456.
5. Foster C, Calman L, Richardson A, May CR, Rogers A, Smith PW. HORIZONS protocol: a UK prospective cohort study to explore recovery of health and well-being in adults diagnosed with cancer. BMJ Open. 2019;9(7):e029662.
6. Fowler NR, Schonberg MA, Sachs GA, Schwartz PH, Gao S, Lane KA, et al. Supporting breast cancer screening decisions for caregivers of older women with dementia: study protocol for a randomized controlled trial. Trials. 2018;19(1):678.
7. Garcia-Gutierrez S, Orive M, Sarasqueta C, Legarreta MJ, Gonzalez N, Redondo M, et al. Health services research in patients with breast cancer (CAMISS-prospective): study protocol for an observational prospective study. BMC Cancer. 2018;18(1):54.
8. Goyal A, Mann GB, Fallowfield L, Duley L, Reed M, Dodwell D, et al. POSNOC-POsitive Sentinel NOde: adjuvant therapy alone versus adjuvant therapy plus Clearance or axillary radiotherapy: a randomised controlled trial of axillary treatment in women with early-stage breast cancer who have metastases in one or two sentinel nodes. BMJ Open. 2021;11(12):e054365.
9. Harcourt D, Paraskeva N, White P, Powell J, Clarke A. A study protocol of the effectiveness of PEGASUS: a multi-centred study comparing an intervention to promote shared decision making about breast reconstruction with treatment as usual. BMC Medical Informatics and Decision Making. 2017;17(1):143.
10. Hild S, Teigne D, Ferrat E, Banaszuk A-S, Berquet K, Lebon A, et al. Breast cancer: a randomized controlled trial assessing the effect of a decision aid on mammography screening uptake: study protocol. Frontiers in Oncology. 2023;13:1128467.
11. Johnson L, Holcombe C, O'Donoghue JM, Jeevan R, Browne J, Fairbrother P, et al. Protocolfor a national cohort study to explore thelong-termclinical and patient-reported outcomes and cost-effectiveness of implant-based and autologousbreast reconstruction after mastectomy for breast cancer: the brighter study. BMJ Open. 2021;11(8):e054055.
12. Kaur M, Pusic AL, Cano SJ, Xie F, Bordeleau L, Zhong T, Klassen A. International phase 1 study protocol to develop a health state classification system for a preference-based measure for women with breast cancer: the BREAST-Q Utility module. BMJ Open. 2020;10(1):e034451.
13. Knerr S, Wernli KJ, Leppig K, Ehrlich K, Graham AL, Farrell D, et al. A web-based personalized risk communication and decision-making tool for women with dense breasts: Design and methods of a randomized controlled trial within an integrated health care system. Contemporary Clinical Trials. 2017;56:25-33.
14. Lagendijk M, Vos EL, Koning AHJ, Hunink MGM, Pignol JP, Corten EML, et al. TUmor-volume to breast-volume RAtio for improving COSmetic results in breast cancer patients (TURACOS); a randomized controlled trial. BMC Cancer. 2017;17(1):336.
15. Park H-Y, Nam KE, Lim J-Y, Yeo SM, In Lee J, Hwang JH. Real-time interactive digital healthcare system for post-operative breast cancer patients: study protocol for a randomized controlled trial. Trials. 2021;22(1):549.
16. Patel BK, Ridgeway JL, Ghosh K, Rhodes DJ, Borah B, Jenkins S, et al. Behavioral and psychological impact of returning breast density results to Latinas: study protocol for a randomized clinical trial. Trials. 2019;20(1):744.
17. Poder TG, Carrier N, McFadden N, Pavic M. Health utilities in cancer patients: A study protocol for a prospective, longitudinal cohort using online survey. Medicine. 2019;98(9):e14647.
18. Pons-Rodriguez A, Forne Izquierdo C, Vilaplana-Mayoral J, Cruz-Esteve I, Sanchez-Lopez I, Rene-Rene M, et al. Feasibility and acceptability of personalised breast cancer screening (DECIDO study): protocol of a single-arm proof-of-concept trial. BMJ Open. 2020;10(12):e044597.
19. Roberto A, Colombo C, Candiani G, Giordano L, Mantellini P, Paci E, et al. Personalised informed choice on evidence and controversy on mammography screening: study protocol for a randomized controlled trial. BMC Cancer. 2017;17(1):429.
20. Touillaud M, Fournier B, Perol O, Delrieu L, Maire A, Belladame E, et al. Connected device and therapeutic patient education to promote physical activity among women with localised breast cancer (DISCO trial): protocol for a multicentre 2x2 factorial randomised controlled trial. BMJ Open. 2021;11(9):e045448.
21. van Beusekom M, Cameron J, Bedi C, Banks E, Kelsey T, Humphris G. Development, acceptability and feasibility of a communication skills training package for therapeutic radiographers to reduce fear of recurrence development in breast cancer patients (FORECAST2). Pilot and Feasibility Studies. 2018;4:148.
22. Wang CC, Geraghty S, Fox-Harding C, Wang C. Effects of a nurse-led Tai Chi programme on improving quality of life, mental wellbeing, and physical function of women with breast cancer: protocol for a randomized controlled trial. Women's Health (17455057). 2022;18:1-7.
23. Roose E, Leysen L, Lahousse A, van Wilgen P, Bults R, Huysmans E, et al. The effect of perceived injustice targeted pain neuroscience education among breast cancer survivors: a protocol for a randomized controlled trial...Pain Science in Motion IV Congress 2022, May 19-20th, Maastricht, Netherlands. Pain Practice. 2022;22:44-.

Wrong outcome - QoL no utility measure (N=18):

1. Abu-Helalah M, Mustafa H, Alshraideh H, Alsuhail AI, A Almousily O, Al-Abdallah R, et al. Quality of life and psychological wellbeing of breast cancer survivors in the Kingdom of Saudi Arabia. APJCP. 2022;23(7):2291-7.
2. Bennedsgaard K, Ventzel L, Themistocleous AC, Bennett DL, Jensen AB, Jensen AR, et al. Long-term symptoms of polyneuropathy in breast and colorectal cancer patients treated with and without adjuvant chemotherapy. Cancer Medicine. 2020;9(14):5114-23.
3. Chu Y-R, Kung P-T, Liu L-C, Lin C-Y, Ou-Yang F, Yue C-H, et al. Comparison of quality of life between breast cancer patients treated with and without adjunctive traditional Chinese medicine in Taiwan. Integrative Cancer Therapies. 2023;22:15347354221150907.
4. Hao B, Feng Y. The effect of targeted psychological nursing intervention on postoperative pain and quality of life in patients with radical mastectomy. Minerva Medica. 2022;113(3):579-81.
5. Heiman J, Onerup A, Bock D, Haglind E, Olofsson Bagge R. The effect of nonsupervised physical activity before and after breast cancer surgery on quality of life: results from a randomized controlled trial (PhysSURG-B). SJS. 2022;111(4):75-82.
6. Herrera de la Muela M, Garcia Lopez E, Frias Aldeguer L, Gomez-Campelo P. Protocol for the BRECAR study: a prospective cohort follow-up on the impact of breast reconstruction timing on health-related quality of life in women with breast cancer. BMJ Open. 2017;7(12):e018108.
7. Lee JM, Lowry KP, Cott Chubiz JE, Swan JS, Motazedi T, Halpern EF, et al. Breast cancer risk, worry, and anxiety: effect on patient perceptions of false-positive screening results. Breast. 2020;50:104-12.
8. Lopes-Conceicao L, Brandao M, Araujo N, Severo M, Dias T, Peleteiro B, et al. Quality of life trajectories in breast cancer patients: an updated analysis 5 years after diagnosis. Journal of Public Health. 2021;43(1):e133-e4.
9. Roberson PNE, Cortez G, Lenger K, Bell C, Freeman T, Heidel R, Lloyd J. Quality of life fluctuations before and after breast surgery for estrogen-positive breast cancer patients living in South-Central Appalachia: a prospective pilot study. The Breast Journal. 2021;27(4):400-2.
10. van Waart H, van Dongen JM, van Harten WH, Stuiver MM, Huijsmans R, Hellendoorn-van Vreeswijk JAJH, et al. Cost-utility and cost-effectiveness of physical exercise during adjuvant chemotherapy. HEPAC. 2018;19(6):893-904.
11. Verbeek JGE, Atema V, Mewes JC, van Leeuwen M, Oldenburg HSA, van Beurden M, et al. Cost-utility, cost-effectiveness, and budget impact of Internet-based cognitive behavioral therapy for breast cancer survivors with treatment-induced menopausal symptoms. Breast Cancer Research and Treatment. 2019;178(3):573-85.
12. Gil GOB, Andrade WP, Costa Diniz PH, Cantidio FS, Vieira Gil ML, Queiroz IN, et al. A phase II randomized clinical trial to assess toxicity and quality of life of patients with breast cancer with hypofractionated versus conventional fractionation radiotherapy with regional nodal irradiation. Journal of Clinical Oncology. 2023;41:588-.
13. Kuan CC, Kuo WH, Chang SH, Sun HF. A longitudinal study on the changes in the self‐efficacy of breast cancer patients during adjuvant chemotherapy. Nursing Open. 2023;10(5):2912-9.
14. Doege D, Thong MSY, Koch-Gallenkamp L, Betrram H, Eberle A, Holleczek B, et al. 171 (PB-084) Poster - Clinical and sociodemographic determinants of disease-specific health-related quality of life in long-term breast cancer survivors...13th European Breast Cancer Conference (EBCC-13), November 16-18, 2022, Barcelona, Spain. European Journal of Cancer. 2022;175:S55-S.
15. Grusdat NP, Stäuber A, Tolkmitt M, Schnabel J, Schubotz B, Wright PR, et al. Cancer treatment regimens and their impact on the patient-reported outcome measures health-related quality of life and perceived cognitive function. Journal of Patient-Reported Outcomes. 2022;6(1):1-13.
16. Wyld L, Reed M, Collins K, Burton M, Lifford K, Edwards A, et al. 8B Oral - Cluster randomised trial to evaluate the clinical benefits of decision support interventions for older women with operable breast cancer...12th European Breast Cancer Conference (EBCC-12), October 2-3, 2020 (Virtual Conference). European Journal of Cancer. 2020;138:S7-S.
17. Tantawy SA, Abdelbasset WK, Nambi G, Kamel DM. Comparative study between the effects of kinesio taping and pressure garment on secondary upper extremity lymphedema and quality of life following mastectomy: a randomized controlled trial. Integrative Cancer Therapies. 2019;18:1-10.
18. Andersen MR, Sweet E, Hager S, Gaul M, Dowd F, Standish LJ. Effects of vitamin D use on health-related quality of life of breast cancer patients in early survivorship. Integrative Cancer Therapies. 2019;18:1-12.

Wrong outcome (e.g wrong utility measure, only decisional conflict) (N=22):

1. Abelson J, Tripp L, Sussman J. 'I just want to be able to make a choice': results from citizen deliberations about mammography screening in Ontario, Canada. Health Policy. 2018;122(12):1364-71.
2. Brorson F, Elander A, Thorarinsson A, Hansson E. Patient reported outcome and quality of life after delayed breast reconstruction - an rct comparing different reconstructive methods in radiated and non-radiated patients. Clinical Breast Cancer. 2022;22(8):753-61.
3. Cadet T, Aliberti G, Karamourtopoulos M, Jacobson A, Siska M, Schonberg MA. Modifying a mammography decision aid for older adult women with risk factors for low health literacy. Health Literacy Research and Practice. 2021;5(2):e78-e90.
4. DuBenske L, Ovsepyan V, Little T, Schrager S, Burnside E. Preliminary evaluation of a breast cancer screening shared decision-making aid utilized within the primary care clinical encounter. Journal of Patient Experience. 2021;8:23743735211034039.
5. Eden KB, Ivlev I, Bensching KL, Franta G, Hersh AR, Case J, et al. Use of an online breast cancer risk assessment and patient decision aid in primary care practices. Journal of Women's Health (2002). 2020;29(6):763-9.
6. Fernandez-Feito A, Canga-Gutierrez C, Paz-Zulueta M. A mixed-methods study to evaluate the acceptability of information leaflets for breast cancer screening. Journal of Clinical Nursing. 2021;30(11-12):1760-72.
7. Gunn CM, Maschke A, Paasche-Orlow MK, Housten AJ, Kressin NR, Schonberg MA, Battaglia TA. Using mixed methods with multiple stakeholders to inform development of a breast cancer screening decision aid for women with limited health literacy. MDM Policy & Practice. 2021;6(2):23814683211033249.
8. Liu Y, Kornfield R, Yang EF, Burnside E, Keevil J, Shah DV. Patient-provider communication while using a clinical decision support tool: explaining satisfaction with shared decision making for mammography screening. BMC Medical Informatics and Decision Making. 2022;22(1):323.
9. Norfjord Van Zyl M, Akhavan S, Tillgren P, Asp M. Experiences and perceptions about undergoing mammographic screening: a qualitative study involving women from a county in Sweden. International Journal of Qualitative Studies on Health and Well-Being. 2018;13(1):1521256.
10. Quinlan A, O'Brien KK, Galvin R, Hardy C, McDonnell R, Joyce D, et al. Quantifying patient preferences for symptomatic breast clinic referral: a decision analysis study. BMJ Open. 2018;8(5):e017286.
11. Reder M, Soellner R, Kolip P. Do women with high ehealth literacy profit more from a decision aid on mammography screening? testing the moderation effect of the eHEALS in a randomized controlled trial. Frontiers in Public Health. 2019;7:46.
12. Salzman B, Bistline A, Cunningham A, Silverio A, Sifri R. Breast cancer screening shared decision-making in older African-American women. Journal of the National Medical Association. 2020;112(5):556-60.
13. Sattar S, Alibhai SMH, Brennenstuhl S, Kulik M, MacDonald ME, McWatters K, et al. Health status, emergency department visits, and oncologists' feedback: an analysis of secondary endpoints from a randomized phase II geriatric assessment trial. Journal of Geriatric Oncology. 2019;10(1):169-74.
14. Schonberg MA, Jacobson AR, Karamourtopoulos M, Aliberti GM, Pinheiro A, Smith AK, et al. Scripts and strategies for discussing stopping cancer screening with adults > 75 years: a qualitative study. Journal of General Internal Medicine. 2020;35(7):2076-83.
15. Seitz HH, Schapira MM, Gibson LA, Skubisz C, Mello S, Armstrong K, Cappella JN. Explaining the effects of a decision intervention on mammography intentions: the roles of worry, fear and perceived susceptibility to breast cancer. Psychology & Health. 2018;33(5):682-700.
16. Skandarajah AR, Lisy K, Ward A, Bishop J, Lacey K, Mann B, Jefford M. Patient-reported outcomes in survivors of breast cancer one, three, and five years post-diagnosis: a cancer registry-based feasibility study. Quality of Life Research. 2021;30(2):385-94.
17. Toledo-Chavarri A, Rue M, Codern-Bove N, Carles-Lavila M, Perestelo-Perez L, Perez-Lacasta MJ, Feijoo-Cid M. A qualitative study on a decision aid for breast cancer screening: views from women and health professionals. European Journal of Cancer Care. 2017;26(3).
18. Toure M, Pavic M, Poder TG. Second version of the short form 6-dimension value set elicited from patients with breast and colorectal cancer: a hybrid approach. Medical Care. 2023.
19. Woolf SH, Krist AH, Lafata JE, Jones RM, Lehman RR, Hochheimer CJ, et al. Engaging patients in decisions about cancer screening: exploring the decision journey through the use of a patient portal. American Journal of Preventive Medicine. 2018;54(2):237-47.
20. Yang EF, Shah DV, Burnside ES, Little TA, Garino N, Campbell CE. Framing the clinical encounter: shared decision-making, mammography screening, and decision satisfaction. Journal of Health Communication. 2020;25(9):681-91.
21. Fernández‐Feito A, Canga‐Gutiérrez C, Paz‐Zulueta M. A mixed‐methods study to evaluate the acceptability of information leaflets for breast cancer screening. Journal of Clinical Nursing (John Wiley & Sons, Inc). 2021;30(11/12):1760-72.
22. Ryu M, Hwang JI. Cancer site differences in the health‐related quality of life of Korean cancer survivors: results from a population‐based survey. Public Health Nursing. 2019;36(2):144-54.

Other (N=30):

1. Chapman CH, Schechter CB, Cadham CJ, Trentham-Dietz A, Gangnon RE, Jagsi R, Mandelblatt JS. Identifying equitable screening mammography strategies for black women in the United States using simulation modeling. Annals of Internal Medicine. 2021;174(12):1637-46.
2. Davidovic M, Zielonke N, Lansdorp-Vogelaar I, Segnan N, de Koning HJ, Heijnsdijk EA. Disability-adjusted life years averted versus quality-adjusted life years gained: a model analysis for breast cancer screening. Value in Health. 2021;24(3):353-60.
3. Elkin EB, Pocus VH, Mushlin AI, Cigler T, Atoria CL, Polaneczky MM. Facilitating informed decisions about breast cancer screening: development and evaluation of a web-based decision aid for women in their 40s. BMC medical informatics and decision making. 2017;17(1):29.
4. Froelich MF, Kaiser CG. Cost-effectiveness of MR-mammography as a solitary imaging technique in women with dense breasts: an economic evaluation of the prospective TK-Study. European Radiology. 2021;31(2):967-74.
5. Hernandez Alava M, Pudney SE, Wailoo AJ. Does EQ-5D tell the whole story? Statistical methods for comparing the thematic coverage of clinical and generic outcome measures, with application to breast cancer. Value in Health. 2023.
6. Jerusalem G, Delea TE, Martin M, De Laurentiis M, Nusch A, Beck JT, et al. Quality-adjusted survival with ribociclib plus fulvestrant versus placebo plus fulvestrant in postmenopausal women with HR+/-HER2- advanced breast cancer in the MONALEESA-3 trial. Clinical Breast Cancer. 2022;22(4):326-35.
7. Kaur MN, Klassen AF, Xie F, Bordeleau L, Zhong T, Cano SJ, et al. An international mixed methods study to develop a new preference-based measure for women with breast cancer: the BREAST-Q Utility module. BMC Women's Health. 2021;21(1):8.
8. Klapproth CP, Fischer F, Rose M, Karsten MM. Health state utility differed systematically in breast cancer patients between the EORTC QLU-C10D and the PROMIS Preference Score. Journal of Clinical Epidemiology. 2022;152:101-9.
9. Li Y, Reed SD, Winger JG, Hyland KA, Fisher HM, Kelleher SA, et al. Cost-effectiveness analysis evaluating delivery strategies for pain coping skills training in women with breast cancer. The Journal of Pain. 2023.
10. Mambourg F, Kohn L. [Breast cancer screening: a tool for informed decision making]. Depistage du cancer du sein: un outil pour aider les femmes a prendre une decision eclairee. Revue Medicale de Bruxelles. 2018;39(4):410-5.
11. Martin J, Garcia S, Anton-Ladislao A, Ferreiro J, Martin M, Padierna A, Quintana JM. Variables related to health-related quality of life among breast cancer survivors after participation in an interdisciplinary treatment combining mindfulness and physiotherapy. Cancer Medicine. 2023.
12. Mittmann N, Stout NK, Tosteson ANA, Trentham-Dietz A, Alagoz O, Yaffe MJ. Cost-effectiveness of mammography from a publicly funded health care system perspective. CMAJ Open. 2018;6(1):E77-E86.
13. Montagnese C, Porciello G, Vitale S, Palumbo E, Crispo A, Grimaldi M, et al. Quality of life in women diagnosed with breast cancer after a 12-month treatment of lifestyle modifications. Nutrients. 2020;13(1).
14. Noman S, Shahar HK, Abdul Rahman H, Ismail S. Effectiveness of an educational intervention of breast cancer screening practices uptake, knowledge, and beliefs among Yemeni female school teachers in Klang Valley, Malaysia: a study protocol for a cluster-randomized controlled trial. International Journal of Environmental Research and Public Health. 2020;17(4).
15. Rautalin M, Jahkola T, Roine RP. The cost of breast cancer surgery - Is the money spent reflected on health-related quality of life? In Vivo. 2022;36(5):2279-86.
16. Rautalin M, Jahkola T, Roine RP. Breast reconstruction-prospective follow up on breast cancer patients' health-related quality of life. World Journal of Surgery. 2022;46(4):836-44.
17. Saver BG, Mazor KM, Luckmann R, Cutrona SL, Hayes M, Gorodetsky T, et al. Persuasive interventions for controversial cancer screening recommendations: testing a novel approach to help patients make evidence-based decisions. Annals of Family Medicine. 2017;15(1):48-55.
18. Schousboe JT, Sprague BL, Abraham L, O'Meara ES, Onega T, Advani S, et al. Cost-effectiveness of screening mammography beyond age 75 years: a cost-effectiveness analysis. Annals of Internal Medicine. 2022;175(1):11-9.
19. Sicsic J, Pelletier-Fleury N, Carretier J, Moumjid N. Preferences des femmes vis-a-vis du depistage du cancer du sein. 2020;2(HS2):7-17.
20. Sicsic J, Pelletier-Fleury N, Carretier J, Moumjid N. [Eliciting women's preferences for breast cancer screening]. Preferences des femmes vis-a-vis du depistage du cancer du sein. 2019;S2(HS2):7-17.
21. Sun L, Sadique Z, Dos-Santos-Silva I, Yang L, Legood R. Cost-effectiveness of breast cancer screening programme for women in rural China. International Journal of Cancer. 2019;144(10):2596-604.
22. Sun L, Legood R, Sadique Z, Dos-Santos-Silva I, Yang L. Cost-effectiveness of risk-based breast cancer screening programme, China. Bulletin of the World Health Organization. 2018;96(8):568-77.
23. Thavorn K, Coyle D, Hoch JS, Vandermeer L, Mazzarello S, Wang Z, et al. A cost-utility analysis of risk model-guided versus physician's choice antiemetic prophylaxis in patients receiving chemotherapy for early-stage breast cancer: a net benefit regression approach. Supportive Care in Cancer. 2017;25(8):2505-13.
24. Tina Shih Y-C, Dong W, Xu Y, Shen Y. Assessing the cost-effectiveness of updated breast cancer screening guidelines for average-risk women. Value in Health. 2019;22(2):185-93.
25. Tollens F, Baltzer PAT, Dietzel M, Schnitzer ML, Kunz WG, Rink J, et al. Cost-effectiveness of mr-mammography in breast cancer screening of women with extremely dense breasts after two rounds of screening. Frontiers in Oncology. 2021;11:724543.
26. van Ravesteyn NT, Schechter CB, Hampton JM, Alagoz O, van den Broek JJ, Kerlikowske K, et al. Trade-offs between harms and benefits of different breast cancer screening intervals among low-risk women. Journal of the National Cancer Institute. 2021;113(8):1017-26.
27. Vos E, Koppert L, van Lankeren W, Verhoef C, Koerkamp BG, Hunink M. A preliminary prediction model for potentially guiding patient choices between breast conserving surgery and mastectomy in early breast cancer patients; a Dutch experience. Quality of Life Research. 2018;27(2):545-53.
28. Williamson SZ, Johnson R, Sandhu HK, Parsons N, Jenkins J, Casey M, et al. Communicating benign biopsy results by telephone in the NHS Breast Screening Programme: a protocol for a cluster randomised crossover trial. BMJ Open. 2019;9(8):e028679.
29. Zhao J, Qiao G, Liang Y, Li J, Hu W, Zuo X, et al. Cost-effectiveness analysis of PEG-rhG-CSF as primary prophylaxis to chemotherapy-induced neutropenia in women with breast cancer in China: results based on real-world data. Frontiers in Pharmacology. 2021;12:754366.
30. Jerusalem G, Delea TE, Martin M, Laurentiis MD, Nusch A, Beck JT, et al. Quality-adjusted survival with ribociclib plus fulvestrant versus placebo plus fulvestrant in postmenopausal women with HR±HER2- advanced breast cancer in the MONALEESA-3 trial. Clinical Breast Cancer. 2022;22(4):326-35.

Responses to stakeholder comments

| **Stakeholder** | **Comment** | **Response** |
| --- | --- | --- |
| Question 1 Are the objectives and methods of this evidence review clear? | | |
| Howard Tracer, USPSTF | Yes | Thank you. No changes requested. |
| Somto Ibezi, Black Physicians Canada | Yes | Thank you. No changes requested. |
| Kathryn Boyd, Nova Scotia Health | Yes. Objectives clearly stated with clinical question identified. Methods were extensive and clearly defined including criteria, processes used and aim/goal of research. | Thank you. No changes requested. |
| Charlotte Yong-Hing, BC Cancer Agency | Yes. | Thank you. No changes requested. |
| Dee Anne Carol, Alberta Health | Yes | Thank you. No changes requested. |
| Julie Brunet, Québec INESSS (Institut national d’excellence en santé et en services sociaux) | No. The Background in the Abstract should be more elaborate if this paper is published as a stand-alone paper like the previous version in 2018 (Background and Purpose)  Objective in the main section is clear.  Methods in Abstract section need to be clarified.  Please add a sentence to increase comprehension for non-familiar user of Health State Utility assessment and Health State utility values (HSUVs) literature type, since this kind of information was not in your previous report of 2018.  ex: Utilities are measured on a scale of 0 to 1, in which 0 represents a health state equivalent to death and 1 represents a health state of perfect health.  Ref:Chang et al. Explaining Health State Utility Assessment  JAMA. 2020;323(11):1085-1086. doi:10.1001/jama.2020.0656  This will allow everybody to understand the numbers presented in the FINDINGS section of the Abstract.  Main section  Eligibility (p7)  Table 1. Inclusion and exclusion criteria: why Intervention in the standard PICO was changes for Exposure?  Under table 1 Eligibility criteria  TTO, tine tradeoff?-time tradeoff?  Literature Search (p10)  Searches were restricted by language to include full texts published in English and French, with a publication date of 2017 onwards. …. To capture studies on HSUVs published between 2000 and 2017 (for utilities related to screening outcomes) or between 2014 to 2017.  Should be: 2000-current and 2014- current or 2000-2023 and 2014-2023? since the previous documents (2018) indicates that the search for the review of women’s values and preferences was updated on December 2017?  The explanation in this section doesn’t match the information presented in the Methods section-Eligibility criteria TABLE 1-for timing (Non-HSUV studies & HSUVs in exposures 1-5: Published on or after 2000/ For HSUVs in exposures 6 & 7: published 2014 or later)  Risk of Bias Assessments (p14)  The validated tool used to assess the risk of bias is not clearly stated. GRADE guidance is normally used for systematic review not primary research evaluation? You refer to Zhang et al 2019 for the RoB assessment, but there is 9 questions in supplement 2&3 (table S2.7 & S3.7) and 7 in Zhang et al article. Also, there is no confounding factors question in your évaluation…  Please elaborate on this way to perform a RoB analysis.  Since you have several RCT in your selection, the use of a specific validated tool such as RoB2 should be more appropriate?  Data analysis (p15)  HSUV data  "If variance measures were not reported we used one from a similar study"  This strategy seems strange to me, but I'm not familiar with HSUV studies...  Please clarify this decision. | Thank you for the excellent comments/suggestions.  We have expanded the background in the abstract as suggested.  We added into the abstract and introduction information about interpreting utility values and what could be used as a minimally importance difference to the public.  For our PICOs, we used exposure vs. intervention because for this review we are not interested in the effects of an intervention (e.g. effectiveness of a decision aid), but rather data on how an exposure to an outcome (anticipated or experienced) is valued.  We corrected our typo for time trade-off.  Our search update used a revised search to add terms for HSUVs but would have only found those published 2017 onwards, so we also searched references lists in systematic reviews on utilities for previously published studies from either 2000 (screening outcomes) or 2014 (for treatment related utilities). We have added some clarity around this.  We have clarified that there is no published validated risk of bias tool for these questions, and how we modified the questions proposed in the GRADE guidance. Because we were not evaluating the effects between groups that could differ by potential confounders, we did not ask a question specific to this, though we did rate as a risk selected populations (all attending screening), and considered age differences and findings for at-risk groups during the synthesis.  We’ve followed guidance from the Cochrane handbook for dealing with missing data (eg imputing data from similar studies), as long as not very many studies required this, and added this citation and mention that this was used during subgroup analysis (for risk of bias) and findings removed if they differed from others. |
| Davina Gallagher, BC Cancer Agency | Yes | Thank you. No changes requested. |
| Modupe Tunde-Byass, Black Physicians Canada | Yes. The objectives and methods were clearly stated , the review examined the relative importance placed by women age >/=35years on the potential outcomes of breast-cancer screening. Traditionally, women <50 years were not routinely screened. The results of the review revealed a population of women who would not have appreciated the importance of screening. However, such women could be getting their information from other “trusted” sources like social media or communities to make informed decisions.  The review in addition to the well-known databases, screened submissions by stakeholders and reference list. These may not capture the preferences of women between age 35 to 50. | Thank you. We found several studies enrolling women aged 35-50 and for this review it is not considered a limitation that the women haven’t experienced the intervention since this has been shown to lead to belief perseverance, whereby the behavior/attitudes persist despite being given information that may otherwise contradict their values. The studies were all conducted in the context of making a decision about screening. |
| Heather Bryant, Canadian Partnership Against Cancer | Yes. The objective is very succinctly described, and it is of direct relevance to the current Task Force review. Selection of the sample is well-described, as is the search process and the methods of data analysis. It was reassuring to see that the sample included data from a wide range of countries and settings, as subjective weightings of perceived risks and benefits may be influenced by public health or media messages; the fact that the results reflect so many contexts adds to the robustness of the findings. | Thank you. No changes requested. |
| Jennifer Payne, Nova Scotia Health | Not selected: From the background section: ‘This systematic review update examined the relative importance placed by patients aged ≥ 35 years on the potential outcomes of breast-cancer screening.’  From the conclusion section: ‘Evidence across a range of data sources on how informed patients value the potential outcomes from breast-cancer screening will be useful during decision-making for recommendations. Further, the evidence supports providing easily understandable information on possible magnitudes of effects to enable informed decision-making.’  These two statements are not consistent. If the purpose was to determine the relative importance, and to separately determine the value of that type of information in decision-making, then the objective should indicate so.  Given the multi-disciplinary nature of the audiences interested in this work, and presumably the ‘stand-alone’ nature of the document in question, it would help if the methods statement elaborated on the approach (utilities) so that when the reader moves to the results section, there is some basis for interpreting the numerical values (suggestion: insert a sentence prior to the sentence ‘Our main analysis for utilities).  I’ve said previously I have concerns with the lower age threshold in the 30s given that these individuals are not currently eligible for average risk screening anywhere in the country so their insight is less helpful (ie it’s theoretical). Similarly, the results for individuals in their 80s is also less of interest. | Thanks for your comments.  We agree that our objective did not include assessment of use/effectiveness of information provision during decision making. We have revised our conclusions to state that findings support that all outcomes are important and that information on the likelihood of their occurrence may be necessary to enable informed decision making.  We have added details to the abstract and main text to help with interpretation of utility values.  We valued your previous input about age of eligibility and considered this during finalization of our protocol. We kept 35-40 year olds (and 80+) as eligible for the review since we were not wishing to impose any limits on what could be practiced elsewhere and because we wanted to include studies looking at attitudes/intentions about starting to screen in ones 40s for which there is an interest in evaluating. For the studies among people older than 70 we differentiated between those 70-71, 70-79 and 75%+ to make sure any differences were examined. |
| Margo Wilson, Society of Rural Physicians of Canada | From email reply: Overall I really enjoyed this review of such an important topic. My criticism of the paper is that it seemed to extend beyond the primary objective of the paper by including patient preferences regarding cancer treatment. While this is an extremely important topic, in my mind it was beyond the scope of this paper and would be better treated in a separate discussion.  Yes. 1) The objectives of the paper were clearly stated, however, the paper went beyond the stated objectives (examination of HSUVs of various treatment modalities in patients being treated for cancer). 2) The overview of the methods was not clear to the lay-reader, although the full document delineated some of the measures more clearly. 3) Inclusion of search strategies (specific terms) in the main body of the paper would be useful. 4) I’m interested in why no qualitative data was included especially as this study examines patient preferences. | Thanks for reviewing.  When assessing preference/valuation of potential outcomes (e.g. experience of early vs advanced stage of disease, need for chemotherapy, as a marker of more treatment morbidity) using utility values from patients is informative to provide an accurate indication on the impact on quality life (i.e. utility/value) of the health state/outcome (considered a preference in this context). We have elaborated on this in the manuscript. HSUVs from experiencing the different outcomes are a major component of patient preferences as per the GRADE guidance for guideline developers.  We have revised the abstract to try to provide better understanding of the concepts. We will create an accessible lay summary of the review, which will be cited in the manuscript.  We have added a few key concepts about our search into the methods section.  This review did not include qualitative data because we were trying to quantify preferences about the outcomes. If we were examining reasons for decision making, barriers etc we would have done so. |
| Anna Chiarelli, Samantha Fienberg, Jonathan Isenberg, Bronwen McCurdy, Ayesha Salleh, Erin Svara, Rebecca Truscot, Meghan Walker, Ontario Health | Yes. • This was a well done systematic review • Objectives and methods were clear | Thank you. No changes requested. |
| Melissa Coté, Québec INESSS (Institut national d’excellence en santé et en services sociaux) | Yes. The objective is clearly defined.  The methodology is really well written. I appreciated the specification of some of the terms used (all/almost all" = 90%, a "large majority" = 75%, and a "majority" = 50%) which may sometimes seem obvious but which testify to the transparency and rigor behind your evaluation. | Thank you. No changes requested. |
| Jennie Dale, Dense Breasts Canada | No see comments in 4. | Please see responses in 4 |
| MJ DeCoteau, Rethink Breast Cancer | Yes | Thank you. No changes requested. |
| Ariana Del Bianco, Canadian Cancer Society | No. Objective is clearly stated. Introduction section would benefit from plainer language or clearer explanation of complex concepts and study measures, especially for patients this will impact and others referencing this work. For example, providing an example of how to interpret a utility measure within the context of breast cancer screening. | Thank you. We have provided explanation in the abstract and introduction on how to interpret a utility value. We will also create an accessible lay summary of the review and cite this in the manuscript. |
| Paula Gordon, Dense Breasts Canada | No. Comments:  I have been asked to review a draft done by the Alberta Research Centre for Health Evidence, based on 82 studies of the relative importance placed by patients aged ≥ 35 years on the potential outcomes (benefits vs risks) of breast-cancer screening.  In the letter requesting my input, it said that “the findings will be considered as one form of patient input when the task force is balancing the effect estimates on benefits and harms…” It does NOT state what other forms of input will be used. That’s a significant omission, given the limitations of the studies that were included. It would have been helpful for reviewers to be told more about the other forms of patient input, including their conclusion(s). | Thank you for your review. The guideline will certainly have this information, but we appreciate the request and have added details into this manuscript’s abstract and objective, e.g. “Other forms of patient engagement are used during the development of recommendations, key messages, and knowledge dissemination tools (e.g., members of task force working group, public advisory network) (cited TF manual) and can help inform this topic on preferences and other considerations related to acceptability, resource use, and feasibility that contribute to recommendations. |
| Carol McClure, PEI Cancer Registry | No. Comments:  I struggled with the disutility measurement. I would like a bit more description of it and how it is calculated (as shown in the supplemental table). I would love to see an example of how it calculated and what the difference in magnitude means. Most disutility measures in the manuscript were <0.10. Should I be interpreting a disutility of 0.8 as a huge difference in preferences? Whereas a disutility of 0.01 is relatively small and not preferentially meaningful? | Thanks very much. We have added information into the abstract and introduction on the range of data (0-1) for utilities and what can be considered important to the Canadian public (about 0.04 or higher). |
| Leah Palmer, Saskatchewan Cancer Agency | Yes | Thank you. No changes requested. |
| Sandy Sehdev, Canadian Breast Cancer Network | Yes. Very clear for a professional audience, less so for the general public | Thank you. We have added some clarification to help interpret some results, such as utilities, and will create an accessible lay summary. |
| Cheryl White, Dense Breasts Canada | No. I did not find this document to be focused on a single concise question. We are interested in attitudes and preferences of women, however some of the included studies did not seem to mention what women were told with respect to risks and benefits prior to responding to the surveys. Perhaps in some cases women were not informed of their risk of missed diagnosis or that the risk of overdiagnosis is mostly described as anxiety rather than a physical harm such as, for example, burns or bruises.  In many studies an upper limit of number of secondary screenings required to save one life was not identified. Did the study authors consider that such a limit might be considerably higher than the magnitudes discussed in these studies? Is there a single study that states how many false positive screens would be too many in exchange for a life saved? It is possible the number would be quite high.  Finally, this seems like a bit of circular argument. If we ask women if they would like to be screened and then choose to recommend or not recommend screening based on those opinions then what would be the purpose of recommendations? | Thank you for your review and comments.  We have described the main data sources used to answer our question, one of which is the relative importance of the potential outcomes *as inferred* from attitudes/intentions (which can incorporate other considerations such as beliefs, cultural expectations). For these studies, we only included studies that provided participants with an estimate of the magnitude of effects for at least one benefit and one harm. We have clarified that these considerations, and our synthesis, was with respect to the outcomes rated by the working group as important or critical. Our study characteristics tables describe all of the data provided to women and we used this information (and the associated limitations about lacking data on outcomes such as overdiagnosis) during our synthesis. We used clinical input from the working group to decipher what an adequate description of overdiagnosis should look like, for example with its inclusion of cancer and not just “precancers”. Any description of how women would feel about overdiagnosis was not necessary for eligibility, and may have been interpreted as potentially biased as there are many potential consequences of being overdiagnosed (e.g., investigations, all treatments, labelling, stigma, psychological impact of being told one has breast cancer, financial).  We agree that more data on elicited trade-offs between additional imaging and breast cancer mortality would have be useful. As described in the results, one study provided a very large range of numbers to select from (up to 10,000), but was otherwise considered at high risk of bias. Our low certainty about the findings for this comparison reflects this. We added a statement to indicate that an upper limit of the highest acceptable number of FPs (for preventing one life saved) was not evaluated. Other studies of women in their 40s suggest that the number may be variable and (for some) not that high; findings suggested that a majority (>50%) of women may decide against screening at about 5-600 recalls vs deaths averted (study information: 0.5 breast-cancer deaths prevented in 1000, 239-330 FPs and 2 to 10 overdiagnoses per 1000).  This review is seeking to gain better appreciation of what magnitudes of effects are acceptable to women, as well as how much variability between people there may be. This could help with deciding about both the direction of recommendations as well as whether or to what degree informed decision making is recommended. Using a systematic review to understand this from an informed perspective is thought to be highly valuable. |
| Jamie-Lynne Bell, Department of Health and Wellness, PEI | Yes | Thank you. No changes requested. |
| Trina Buick, Canadian Association of Nurses in Oncology | Yes. Comprehensive overview of methods and is well structured.  Provides a detailed explanation of the complex eligibility criteria and decisions made | Thank you. No changes requested. |
| Rene Wittmer (peer reviewer), Médecin de famille, Universite de Montreal | Yes. Very clear, nothing to add. | Thank you. No changes requested. |
| Question 2 Were the results clearly stated? | | |
| Howard | Yes. I suggest highlighting in the abstract, when discussing overdiagnosis, that only 33% of women correctly identified/ defined overdiagnosis on acknowledge test. It’s an important caveat and limitation to that evidence. | Thank you. We added a comment to this effect in the abstract. |
| Ibezi | Yes. | Thank you. No changes requested. |
| Boyd | Yes. Results were clearly stated/summarized with clear identification of exclusions and why, including how that process took place and who was involved. | Thank you. No changes requested. |
| YongHing | Yes | Thank you. No changes requested. |
| Carol | Yes | Thank you. No changes requested. |
| Brunet | No.  ABSTRACT  Without further explanation in the Method abstract section, it’s not clear what disutilities numbers means in the FINDINGS abstract section for people not aware with the concept of health-state utility values.  RESULTS (MAIN SECTION)  Literature flow (p18)  I don’t understand if this document is an update why 28/82 included studies were included in the previous review? (and one of the previously included studies was excluded in this review…)  More clarity is need.  Table 2: add BCS to the acronym list under the table p23 | Thank you for your comments.  We have elaborated in the abstract and introduction how to interpret utility values.  When we update reviews we include all studies previously reviewed that meet our eligibility criteria, to ensure our synthesis captures all relevant data for our research question. We have made this more explicit, and explained that the one excluded study was because of revised eligibility about age of participants in this update.  Thank you for pointing out our omission of describing this abbreviation in our footnotes; we have corrected this. |
| Gallagher | Yes | Thank you. No changes requested. |
| TundeByass | Yes | Thank you. No changes requested. |
| Bryant | Yes. The results are exceptionally clearly stated. The addition of the column “What does the evidence say?” is a brilliant addition to the results, as it allows the reader to understand the outcome for each element that will be considered in the final interpretation of how women view the risks and benefits of screening. | Thank you. No changes requested. |
| Payne | Not selected: Had there been an elaboration of the term utility (and how to interpret it in the methods statement), then the results statement as is would be fine. Right now, something is missing.  As I quickly scanned the tables, I am a bit concerned that in the second table of supplementary material, there are a couple of references to samples of individuals who were attending genetic testing centres. This is concerning given that individuals undergoing genetic screening may be at significantly higher breast cancer risk than an average risk population, and these people may consider information re breast cancer very differently – I don’t think they should be included. | Thank you.  We have elaborated in the abstract and introduction how to interpret utility values.  One study of participants attending a genetic testing centre (n 33 of >8000 in the analysis) met our eligibility criteria, because it was determined that fewer than 20% were considered at high risk for breast cancer. Studies with people at moderately increased risk were eligible. |
| Wilson | No.  See overall commentary below.  1) The authors noted that patient recruitment included cancer centers and outpatient centers – I would be interested if this excluded a portion of the populations, especially more rural residents. Are there any studies that address the preferences of rural patients as screening for them may involve the additional considerations of travel and extra cost.  2) Table 3 to me really summarized the initially identified objectives of the review.  3) It was not clear to me how disutility on treatment options affected one’s opinions on screening – perhaps an area for further discussion? This seemed outside of the intended scope of this paper.  4) Table 2 initially looks at screening and diagnostics for false positive screens, but then proceeds to discuss treatment. I’m not sure how this relates to the initial stated objectives or if the scope needs to be broadened to reflect the data included. There is also some discussion of patients who were not detected by screening, which is again not entirely related to patient preferences on screening.  5) Table 4 lays out the evidence, but it seems to contradict the discussion outlined in the body of the text. Is there a way to visually or thematically display the weight of the evidence more effectively? | Thank you for your review and comments.   1. Our protocol did not specify geographical residence as a major potential confounder for our review which focus on the importance of outcomes to individuals rather than possible inequities in screening effects from accessibility. 2. When assessing preference/valuation of potential outcomes (e.g. experience of early vs advanced stage of disease, need for chemotherapy, as a marker of more treatment morbidity) using utility values from patients is informative to provide an accurate indication on the impact on quality life (ie disutility) of the health state/outcome (considered a preference in this context). We have elaborated on this in the manuscript. We have revised the abstract to try to provide better understanding of the concepts. We agree though that the data in Table 3 is very relevant and perhaps easiest to use for decision making. 3. Table 2 examines the value to people of all of the outcomes examined by the task force for this update. The task force was interested in the value to patients of reducing late stage disease or being able to avoid treatments, such as chemotherapy, that could cause more serious side effects. 4. The summary statements include in table 4 are our main conclusions, supported by the narrative and the tables in the supplement 3. It is our assessment that the summary statements capture what was reported across the relevant studies quite well (that is, are not contradictory to what the studies reported). Because we had no other comments about the information in this table we did not make any changes. |
| Chiarelli Fienberg, Isenberg, McCurdy, Salleh, Svara, Truscot, Walker | Yes. • Results are clearly stated in the tables, however may be difficult to understand for individuals without this expertise/not working in this area.  • Suggest providing an example of how to interpret utility/disutility findings, to ensure the audience understands the results (e.g, Table 2. Summary of findings on health state utilities). | Thank you. We are making an accessible lay summary to cite in the manuscript. We have also added in several places more information on how to interpret utility values. |
| Coté | Yes. I appreciate the tabular presentation of the results, which gives a quick look at the overall picture. However, I would have liked to have the references written with the author name and the year of publication (table 2), as in appendix 2, to facilitate identification of the studies selected. I understand, however, that this would significantly lengthen the table. | Thank you for the feedback We appreciate that tables cannot include all information that may be useful to all readers, but need to ensure the tables are concise and follow journal formatting style. We hope that the supplemental files assist those that require more information. |
| Dale | No see comments in 4. | Please see responses in 4. |
| DeCoteau | No. While the objectives and methods of the review were clear, particularly to lay people, the findings should have a plain language version to facilitate clear communication and understanding among the general population. If the intent of this evidence review is to inform public-facing guidelines, then the evidence review should be accessible and framed in plain language that can be understood by the public. | Thank you we are preparing a lay summary to cite in the manuscript. |
| DelBianco | Yes | Thank you. No changes requested. |
| Gordon | No. Comments:  The stated purpose of this systematic review as to examine the relative importance placed by patients aged ≥ 35 years on the potential outcomes of breast-cancer screening. The conclusion stated that “how patients value the potential outcomes from breast-cancer screening will be useful during decision-making for recommendations.”  Based on the results of most of the included studies, a large majority of women prioritize early detection of cancer through screening, are not concerned about recalls, and are willing to accept a large percentage of overdiagnosis. THAT should have been the conclusion. | Thank you. We have revisited our conclusion statements and made some minor edits. The data across all datasets (e.g. important disutility of a diagnosis of cancer and a FP, majority in 40s possibly not accepting screening if offering low net benefit) does not strongly support your proposed conclusions. We are confident we have described all results accurately and reached sound conclusions with careful consideration of the study limitations and how we used the findings of each study to answer our research questions. |
| Isenberg | Yes. • Results are clearly stated in the tables, however may be difficult to understand for individuals without this expertise/not working in this area.  • Suggest providing an example of how to interpret utility/disutility findings, to ensure the audience understands the results (e.g, Table 2. Summary of findings on health state utilities). | Thank you we have added clarity about interpreting utility values. |
| McClure | Yes. | Thank you. No changes requested. |
| Palmer | Yes | Thank you. No changes requested. |
| Sehdev | Yes. Quality of evidence, risk of bias and magnitude of conclusions were clearly indicated. | Thank you. No changes requested. |
| White | No. This document does not do a good job of summarizing topics and including the key points in the conclusion. A summary should be added. | Thank you. We have revised our conclusions to be more comprehensive and have prepared a lay summary. |
| Bell | Yes | Thank you. No changes requested. |
| Buick | Yes. Presentation of tables was valuable. | Thank you. No changes requested. |
| Wittmer (peer reviewer) | Yes. Very clear, nothing to add. | Thank you. No changes requested. |
| Question 3 Are the conclusions in the review supported by the data that were reviewed? | | |
| Howard | Yes. I agree with all the points you make in the discussion section. I was surprised by the low disutilities for several treatments. I found it interesting that given the same low net-benefit scenario, women in their 40s tended to decide against screening, while women in their 50s prefer screening. Your statement about “belief perseverance” may be relevant here. I think there may be an effect due societal norms or preexisting bias or expectation on the part of women in their 50s, knowing they should be screened, or perhaps their personal experience with friends who’ve been diagnosed with breast cancer, leading them to discount the benefits/ harms data that is presented.  Patient preferences are very important in shared decision making for breast cancer screening, but it’s important to understand that these preferences can be affected by external factors including context and prior patient expectations. | Thank you. No changes requested. |
| Ibezi | Yes | Thank you. No changes requested. |
| Boyd | Yes | Thank you. No changes requested. |
| YongHing | Yes | Thank you. No changes requested. |
| Carol | Yes | Thank you. No changes requested. |
| Brunet | Yes. ABSTRACT  One sentence could be added to Abstract Conclusions, after the first sentence in other to be more precis? ex: the evidence strongly suggests that the outcomes examined have importance to women of any age. | Thank you we agree and have revised. |
| Gallagher | Yes | Thank you. No changes requested. |
| TundeByass | Yes | Thank you. No changes requested. |
| Bryant | Yes. The conclusions relate well back to the huge volume of data that is presented, and there is a good explanation of the interpretation. It is especially helpful that the authors are quite transparent about the areas about which they are uncertain, and to some extent, to the degree of that uncertainty. | Thank you. No changes requested. |
| Payne | Yes. The results section (which is beautifully laid out in the accompanying tables in excruciating detail which is appreciated) is a bit overwhelming to review, but as best as I can tell, yes. | Thank you. No changes requested. |
| Wilson | Yes. | Thank you. No changes requested. |
| Chiarelli, Fienberg, Isenberg, McCurdy, Salleh, Svara, Truscot, Walker | Not selected. • Suggest adding implications of these results to screening programs in the conclusion | Thank you for the suggestion. We have revised to add “that provision of information on the likelihood of the outcomes may be necessary to enable informed decision making”. |
| Coté | Yes. The conclusions are relevant to the results of the literature review. Your synthesis of the results is very representative.  I appreciate the fact that you have tried to explain certain unexpected results and that you have formulated hypotheses. | Thank you. No changes requested. |
| Dale | No see comments in 4. | Please see responses in 4 |
| DeCoteau | Yes. The conclusions in the review are reflective of the data; however, as a patient group, our concern is two-fold:  - There is a need to have lay-person friendly communications to share the conclusion and an overview of the data.  - Does the data reflect and address considerations around a risk-based screening model that considers factors other than age? | Thank you. We are preparing an accessible lay summary. We have added a note in the limitations that there was some but limited data to indicate findings may be similar across risk groups within age groups. We did not access views on different screening approaches based on risk, though the review on evidence of screening effectiveness will do this and other information will be examined about this by the task force. |
| DelBianco | Yes | Thank you. No changes requested. |
| Gordon | No. Comments:  There were no conclusions clearly stated. Moreover, the majority of the studies were rated “Low” by GRADE. So even though a large majority of women prioritize early detection of cancer through screening, are not concerned about recalls, and are willing to accept a large percentage of overdiagnosis, the risk is that the Task Force will ignore women’s preferences. | Kindly also see our response to your comment in question 2. The task force finds patients preferences (and the level of certainty about the findings) quite important when deciding on the strength and direction of their recommendations. |
| McClure | Yes. Comments: Yes, but this goes back to my lack of understanding of the magnitude of the disutility measurement. What magnitude would make me want to absorb patient preference into my BCS guidelines? | Thank you. Hopefully our addition of information to help interpret the utility data is helpful for you and others. |
| Palmer | Yes | Thank you. No changes requested. |
| Sehdev | Yes. | Thank you. No changes requested. |
| White | No. Again, the conclusion is poorly written. | Thank you. We have revised our conclusions to be more comprehensive and have prepared a lay summary |
| Bell | Yes | Thank you. No changes requested. |
| Buick | Yes. Limitations were succinctly addressed. The challenges to the definition/conceptualization and variability of FP (ie viewed as a benefit and not a harm) could be expanded in the discussion (in terms of bias/women’s understanding).  While qualitative evidence wasn’t examined – it could have supported and added additional insight to address some of the limitations raised.  Conclusions supported that the outcomes examined have importance to women of any age and that personal choice is vital to enable informed decision-making. | Thank you. We have reviewed our discussion of the limitations and think we’ve captured the first two points sufficiently for the purposes of this review. We have expanded on our conclusions and incorporated your suggestions. |
| Wittmer (peer reviewer) | Yes. The results are nuanced, and the terminology aligns well with the data that were reviewed. The entire process is very transparent and easy to understand. | Thank you. No changes requested. |
| Question 4 Do you have any additional comments? | | |
| Howard | No response | No changes requested. |
| Ibezi | I note the paucity of race-related data in the studies. | We agree and have added this as a limitation in the abstract and manuscript conclusions. |
| Boyd | Extensive research provided with clear objective and intentions. | Thank you. No changes requested. |
| YongHing | Task force recommendations should not be based on low certainty evidence. | Thank you. No changes requested. |
| Carol | It was an interesting read. I do not have anything substantial to contribute, but I’m providing my completed checklist.  The only consideration I had was for future research – namely, how some populations’ growing mistrust of health care, coupled with limited relationships with healthcare providers due to lack of access, impact the patient’s ability to objectively make a decision on the benefits/harms of an intervention. Individuals’ personal trust in healthcare was out of scope for this research, however not everyone makes objective decisions solely based on advice from their HCP, particularly if they don’t trust them. It’s hard to sever personal feelings about a health care intervention, so we should consider this when researching informed decisions and consent and keep it in mind for BC screening guidelines.  I didn’t include this in my comments because, as I said, it was stated as out of scope | Thank you for the additional comments. Your comments help support that the evidence from decision aids and other informational materials asking women to decide whether or not to screen (ie data in Table 4) is indirect for the purposes of this review which tries to emulate rational decision-making (based on the effects on outcomes) which is only one aspect of many people’s decision making. We have decided to not add this as a major research objective since it was not the focus of our review (we did not examine all the evidence on factors influencing screening decisions) but we think this aspect has been described in a sufficient manner in our text and Table 1. |
| Brunet | DRAFT-KQ3 Supplementary file 2:  Table S2.7. Summary of Risk of Bias,  The reference of the tool use to perform this assessment should be indicate in the bottom of the table?  DRAFT-KQ3 Supplementary file 3:  Tables S3.7. Summary of Rias of Bias Across Studies  Same comment + correction | Thank you we have added reference to the tool in these supplements. |
| Gallagher | No. | Thank you. No changes requested. |
| TundeByass | It is clear for the most part that informed women value the potentials of breast screening above potential risks. Therefore, an informed decision making process based on personal circumstances and preferences is key in this review and subsequent recommendations. | Thank you. No changes requested. |
| Bryant | It is refreshing to see such a clear and quantitative approach to a complex issue. It will undoubtedly be a helpful lens through which to look once the evidence on effectiveness of screening itself is collated. | Thank you. No changes requested. |
| Payne | Blank | No changes requested. |
| Wilson | Additional comments include:  1) Is there any information given to women about the harms of biopsy (bleeding infection death versus the harms of a cancer found at a later stage?) and how this affects patient decision making  2) The paper notes: “There is uncertainty about whether these trade- offs would be acceptable in situations where the outcome was well understood and in view of this we rated down for indirectness. The findings appear to apply across 40 to 70-year-olds.”  The authors discuss “rating down” for indirectness, but this is a little vague. To me this is the crux of the paper – what are patient’s preferences for screening and acceptance of interventions to rule out false positives, versus treatment preferences for identified cases - and it should be explored further. Also I wonder why there was concern about the outcome not being well-understood – was there discussion or evidence of this in the literature? If so, could it be discussed further?  3) Was there any discussion about differences of values in younger populations? I would presume that preventing a cancer related death or having an early diagnosis may carry more weight in your forties than in your sixties.  4) There were some interesting studies that involved that use of a decision aid – was there any evidence of whether patients preferred the use of a standardized decision aid? Or what kinds of decision aids are the most useful (those delivered in person versus a self-administered one online?)  5) Pg 46 - The discussion addresses that people overestimate the benefits of screening, but in previous discussion and analysis patients are willing to tolerate a high level of false positives in order to identify a cancer diagnosis. I think the discussion around this could be strengthened by inclusion of the latter. | Thank you for the additional comments.   1. No, none of the studies examined this. 2. This review focused on informed preferences, as much as possible, so when data from knowledge tests (as described in the text and reported in detail in the supplementary files) indicated that outcomes such as overdiagnosis were not very well understood in some studies this reduced our certainty since the exposure of interest (information on the benefits and harms) was not well understood. We added a description to the results section to help justify why this was viewed as indirectness. 3. Much of our analysis focused on findings by age and our judgements about the net benefit of screening presented in the studies took into account the larger beneficial effects in those (older) people with higher baseline rates of cancer. 4. Our review did not examine the effectiveness (e.g. use for decision making) or preferences about decision aids. 5. Our discussion about the overestimation of benefits from screening was to justify why we only used studies that provided participants with descriptions and magnitudes of effects, and for why we rated studies at higher risk of bias if they only provided relative effects. The findings of a relatively large number of acceptable false positives is a valid finding of this review and considered a different topic. |
| Chiarelli, Fienberg, Isenberg, McCurdy, Salleh, Svara, Truscot, Walker | • Many people using this systematic review may not have expertise in this specific area. Additional information on how to use the results and interpret these findings would be beneficial. | Thank you, we have added some clarification to help interpret the data and are preparing an accessible lay summary. |
| Coté | General comments:  The document is very well written. Your assessment is very thorough. Congratulations on your excellent work.  Although I am not familiar with HSUV-type evaluations, the methodology allowed me to fully understand and interpret the results. I really appreciated that you added this type of study to your evaluation compared to your 2018 publication.  Breast cancer screening:  The selected studies come from all over the world: Netherlands, USA, South Korea, Finland, the United Kingdom, Australia, Japan, Spain, Norway, Italy, Croatia, Thailand, France, Greece, England, Wales and Germany. Considering that screening is not carried out in the same way everywhere, certain particularities could have an impact on women's perspectives. Here are just a few that came to my mind:  - Biennial vs. annual screening;  - Independent double reading vs. single reading (impact on response time and recall rate);  - Costs of screening incurred by the healthcare system vs. women screened;  - Accessibility of screening.  Without adding sub-analyses to your evaluation, I believe that mentioning these particularities would make it possible to be transparent about the risk of bias in the transferability of results to our Canadian context.  Communication:  Although you mentioned it, it would have been relevant to know women's main sources of information about breast cancer screening, in order to better reach the target audience with the new recommendations. | Thank you for your additional comments.  While it is likely that studies on the effects of screening (actual recall rates, mortality reductions) may be influenced by many of the factors you mention, we think it is less relevant to the data we examined which is focused on responses to the relative magnitudes of the outcomes from screening as presented to women for which studies were quite consistent because of using reported effects based on systematic reviews. We did take into account during our analysis the potential bias/limited applicability from studies conducted in countries where screening is not routine (e.g. Hong Kong).  Our review did not include data on or examine information needs or sources of information for women. Hopefully the dissemination tools created with this task force recommendation will reach the target audience. |
| Dale | As a patient advocate and cofounder of an organization advocating for optimal breast cancer screening, I appreciate the chance to review the Patient Preferences Review Update. In 2018, I was a reviewer of the overall draft guideline. Once again, it is clear from the evidence review that women place higher weight on the benefits as compared to the harms of breast cancer screening. It is clear that they are willing to experience “false positives and “overdiagnosis” to reduce the chance of dying from breast cancer. And yet, similar to 2018, it is inferred that women did not understand what they were being asked. The conclusion is patronizing. As well, the review itself is seen to have a number of issues.  1. The terminology used to assess values and preferences is problematic. The term false positives is pejorative. The review mentions that the Task Force is recommending the use of another term, but false positives is still the term used in the studies. As well, the term overdiagnosis is misleading. Screening does not result in overdiagnosis- only pathology does. The Task Force has continued to overestimate the rate of overdiagnosis due to its use of the compromised CNBSS.  2. In addition to the terminology issue, the time framing is an issue. The 10-year window used to assess benefit is too short a time frame to be used, particularly for a woman in her 40s or 50s. This short time frame may have resulted in some benefits of screening as being presented as very low.  3. Most of the studies used in the review were small. The confidence in the was rated as low. We have seen in previous reviews that some evidence is rated very low. It is not clear what mechanisms are in place to ensure different evidence reviews rate the evidence quality in a consistent manner.  4. The continued focus on All Cause Mortality (ACM) in the review is misleading. ACM is not a useful measure of the impact of breast cancer screening, because deaths from breast cancer represent a relatively small fraction of ACM. Attempts to measure ACM will be impaired by lack of statistical power as well as the challenges of follow-up.  5. The evidence surrounding disutility is questionable. The amount of time disutility is experienced also needs to be taken into account.  Overall, the studies showed that most women are accepting of overdiagnosis and call backs. They are willing to trade a decrease in mortality against an increase in so called harms. These are the same findings which were ignored in 2018. It is hoped that in 2024 women’s preferences will not continue to be dismissed. | Thank you for these additional comments.  We did not intend to be patronizing to any extent and many of the studies we reviewed that examined/tested knowledge made clear statements to this effect, that is, the difficulty to understand the concept of overdiagnosis.   1. We have indicated that false positive is not the best term (and the recommendations will avoid this term) but decided to keep this term in our review since the research widely uses this term. We also carefully examined how studies described this outcome and have added a discussion about this in the manuscript. By definition, in this context, overdiagnosis can only happen within the screened people. 2. We have reported the timeframe used by the studies and interpreted the data in light of this. We are not judging whether the effects are accurate/valid in the studies but rather differentiating between results based on these variations. 3. We have carefully followed internationally accepted guidance when assessing the certainty of the findings, and have justified our rationale for each rating. Though not all reviewers would come to the exact same conclusions, as per GRADE guidance we have been transparent with our assessments and provide all the data to support these. 4. We realize effects on all-cause mortality are hard to establish but this does not mean that it is not important to consider. We did not rate down (e.g. for risk of bias) studies that did not provide women with estimates of the effects on all-cause mortality because we realize how hard it is to present data while also describing the uncertainty around it. 5. We think we have made it quite clear that duration of experience with an outcome will impact its disutility, and have focused a good portion of our discussion on this.   In 2018, the task force used findings about patient preferences, especially around the consistent evidence that there will be some variability in values among patients, to a large extent when developing their recommendations. The findings of both reviews undertaken were clear that not all women will think the benefits outweigh the harms. |
| DeCoteau | The discussion around breast cancer screening continues to focus primarily on age as the risk factor to determine when screening should commence. Considerations should be given to the mounting data that supports a more sophisticated risk-stratification approach to breast screening. | Thank you for the comments. We added a comment in the discussion about the limited data on whether findings would differ based on risk. The review on effectiveness and other information will be used to inform the task force deliberations about differing risk. |
| DelBianco | An additional limitation may be the lack of HSUV studies from Canada. | We agree and added this. |
| Gordon | NOT ENOUGH ROOM, SO REPEATED BELOW, OUTSIDE THIS TABLE  1. In spite of the fact that the vast majority of the studies concluded that women put a higher value of the benefits than the harms of screening, all but a few rated “low” or “very low” by GRADE. This opens the door for the Task Force to ignore women’s true preferences.  2. Most RCTs used only breast cancer mortality as a benefit of screening, ignoring the significant benefit of the opportunity to have successful treatment with less aggressive surgery (lumpectomy vs mastectomy, sentinel node biopsy vs axillary dissection) and the opportunity to avoid chemotherapy. Had those additional benefits been included, it would undoubtedly have increased women’s willingness to accept higher frequency of recalls (“false positives”) and overdiagnosis. The studies that did include stage distribution (reduced advanced disease) and treatment burden (reduced mastectomy) vs FP biopsies were rated low on GRADE.  The Valentine study 2022 (their ref 14), is emblematic of the bias in many of the studies. It was a concerted effort to find ways to persuade patients to decline screening. They seem unable to accept that some choose to be screened, even when there is no data showing mortality reduction. They speak about improving “the quality of patient decisions,” but it’s clear that they define improvement as choosing not to be screened. They describe their discussion of the potential harms of overtreatment, but not the harms of undertreatment. They included patients aged 40-70, but don’t indicate whether they tailored the information provided based on age, or whether they told participants about the negligible incidence of overdetection in younger people.  In their experiment, they told patients that “the intended purpose of cancer screening, which is to detect cancer early with the goal of preventing cancer death,” and that mammography saved 1 life per 1000 people screened, and “harms consisted of the rates of false positives and overdetection per 1000 people screened over a decade for women.” They didn’t state where they sourced the numbers provided to the participants.  All the examples they described used mortality reduction as the only benefit. As one example, they referred to a study that claimed that “Overuse of screening tests (e.g., screening when there is no evidence that the test will benefit patient outcomes) have been reported for… breast cancer.17 It looked at mammography rates in women with severe cognitive impairment, noting that “certain subgroups with cognitive impairment are often screened despite lack of probable benefit.” They concluded that guidelines should explicitly recommend against screening these women.” In this group, the important benefit of early detection that they ignored was the opportunity to do minimal therapy to improve quality of life. Therapy in these patients can be limited to excision under local anesthetic, when cancer is detected early. If cancers are not detected until clinically apparent, it’s often because they have eroded through the skin, and become a nursing challenge: they need constant dressing changes because they ooze; they have unpleasant odor, and are vulnerable to infection.  Mehta KM, Fung KZ, Kistler CE, Chang A, Walter LC. Impact of cognitive impairment on screening mammography use in older US women. Am J Public Health. 2010;100(10):1917–23.  Finally, in “Intervention 4: Narrative,” they stated: Research has found that the most impactful narratives on screening intentions are those describing physical harms from screening, rather than emotional harms or overdetection.37 Since the primary goal of the narrative intervention was to decrease interest in the unbeneficial test, we wanted to present a narrative that described a clearly negative (albeit uncommon) experience while also remaining realistic. Thus, we crafted our narrative to maximally decrease interest for the unbeneficial test by including information about experiencing physical harm, as well as emotional and financial harms, in the context of a false-positive screening test result. The full narrative can be viewed in the supplemental materials. They did not do the same for the harms of underdiagnosis, ie the avoidable pain and suffering when cancer is not detected as early as possible.  Valentine KD, Wegier P, Shaffer VA, Scherer LD. The Impact of 4 Risk Communication Interventions on Cancer Screening Preferences and Knowledge. Medical Decision Making. 2022;42(3):387-97.  - The same applies to any research that passes judgement as to whether screening is justified, and any decision aid that uses that research: ALL the benefits must be included, and by limiting the review to RCTs, that shows how this whole review was biased.    - The outcome of studies that used “decision aids” very much depends on the information given in those decision aids. For example: was breast cancer mortality reduction based on randomized trials (RCTs) or observational studies. Lay women may not realize that data from RCTs underestimates mortality reduction because of contamination and non-compliance. An individual woman in the process of deciding whether she should participate in screening would be better served by observational data (ie potentially 50% breast cancer mortality reduction) rather than population-based, intention-to-treat data (15-20% breast cancer mortality reduction). In particular, since this review is for Canadian women, the decision aids should ideally be based on Canadian data, including the Coldman 2014 “Pan-Canadian study,” and information on recall rates from the Canadian Partnership Against Cancer.  3. Comments on specific Sections  All-cause versus BC Mortality  - Deaths from breast cancer represent a small percentage of all deaths. So even trials that show significant reduction in breast cancer mortality are unlikely to show reduction in all-cause mortality.  - so outcomes from any decision aid that relies on all-cause mortality should be excluded, since it is mathematically almost impossible for all-cause mortality to be positively affected by screening, and it is misleading to tell women that screening doesn’t impact all-cause mortality without a full explanation of the math  - Tabar et al demonstrated reduction in all-cause mortality among the women diagnosed with breast cancer. They used data from the Swedish Two-County Trial of mammographic screening for breast cancer, in which 77 080 women were randomised to an invitation to screening and 55 985 to no invitation. There was a significant 31% reduction in breast cancer mortality in the invited group (RR 0.69, 95% confidence interval (CI) 0.58-0.80; p<0.001), and a significant 19% reduction in deaths from all causes was observed among breast cancer cases in the group invited to screening (RR 0.81, 95% CI 0.72-0.90; p<0.001).  Tabar L, Duffy SW, Yen MF, Warwick J, Vitak B, Chen HH, Smith RA. All-cause mortality among breast cancer patients in a screening trial: support for breast cancer mortality as an end point. J Med Screen. 2002;9(4):159-62. doi: 10.1136/jms.9.4.159. PMID: 12518005.  BC Mortality versus Overdiagnosis  - conclusion that across all ages, at least a majority (>50%) and possibly a large majority (>75%) of patients probably accept up to 6 cases of overdiagnoses to save one death from breast cancer, and the rating by GRADE is moderate. Clearly, women put greater importance on early detection, than the small real risk of overdiagnosis (Puliti). Given that the Task Force overestimates the incidence of overdiagnosis based on the flawed CNBSS trials, it’s not clear how they will use this information.  Puliti D, Duffy SW, Miccinesi G, de Koning H, Lynge E, Zappa M, Paci E; EUROSCREEN Working Group. Overdiagnosis in mammographic screening for breast cancer in Europe: a literature review. J Med Screen. 2012;19 Suppl 1:42-56. doi: 10.1258/jms.2012.012082. PMID: 22972810.  BC Mortality versus False Positives  - women 40-49 prioritize reduction in mortality, over false positives. This is less so in women 50-59, but all studies are rated as low, so this gives the TF the opportunity to ignore women’s preferences.  BC Mortality versus FP biopsies  - For patients 40 or older, a large majority of patients may accept that between 10-15 people experience a FP biopsy to prevent one BC death over many years, but the solitary study is rated as low, so this gives the TF the opportunity to ignore women’s preferences.  Stage Distribution (reduced advanced disease) versus FPs  - For patients 40 or older, a large majority of patients may accept that at least 25 people experience a FP to prevent one advanced stage cancer, but the studies are rated as low, so this gives the TF the opportunity to ignore women’s preferences.  Stage distribution (reduced advanced disease) versus FP biopsies  - For patients 40 or older, a large majority of patients may accept that at least 4 people experience a FP biopsy to prevent one advanced stage cancer, but the studies are rated as low, so this gives the TF the opportunity to ignore women’s preferences.  Treatment burden (reduced mastectomy) versus FP biopsies  - For patients 40 or older, avoiding mastectomy may be much more important than experiencing a FP for a majority of patients, but the solitary study is rated as low, so this gives the TF the opportunity to ignore women’s preferences.  Table S3.2. Making Inferences from Attitudes, Intentions, And Behaviors, by age and judgement of net benefit presented  - For women 40-49: as expected, attitudes on benefits:harms varied based on whether the net-benefit scenarios were presented as high, moderate or low  - for 50 yr old women: a large majority of patients 50 years old probably weigh the benefits as greater than the harms from screening, whether presented with a high- or moderate net-benefit scenario.  - for women 50-69, a large majority of 50 to 69-year-old patients probably weigh the benefits as greater than the harms from screening in both high-and low net-benefit scenarios.  - for women 70+, a large majority of patients 70-71 years of age who have recently screened probably think the benefits outweigh the harms for continuing to screen in a moderate-to-low net benefit scenario, and this was based on one study rated as moderate on GRADE.  - in 3 studies of women 75-early 80s, who have recently screened, a majority but possibly not a large majority may weigh the benefits as greater than the harms for continuing to screen. It is unclear what impact life expectancy has on this preference, but these were rated low on GRADE.  Table S3.3. Direct Preference Data from Mixed Ages  The first 2 studies cited under “Weighing all-cause and BC mortality” Reder 2017 (Germany) and Davey 2005 (Australia) both included all cause mortality in their decision aid.  - Deaths from breast cancer represent a small percentage of all deaths. So even trials that show significant reduction in breast cancer mortality are unlikely to show reduction in all-cause mortality.  - so outcomes from any decision aid that relies on all-cause mortality should be excluded, since it is mathematically almost impossible for all-cause mortality to be positively affected by screening, and it is misleading to tell women that screening doesn’t impact all-cause mortality without a full explanation of the math  - Tabar et al demonstrated reduction in all-cause mortality among the women diagnosed with breast cancer. They used data from the Swedish Two-County Trial of mammographic screening for breast cancer, in which 77 080 women were randomised to an invitation to screening and 55 985 to no invitation. There was a significant 31% reduction in breast cancer mortality in the invited group (RR 0.69, 95% confidence interval (CI) 0.58-0.80; p<0.001), and a significant 19% reduction in deaths from all causes was observed among breast cancer cases in the group invited to screening (RR 0.81, 95% CI 0.72-0.90; p<0.001).  Tabar L, Duffy SW, Yen MF, Warwick J, Vitak B, Chen HH, Smith RA. All-cause mortality among breast cancer patients in a screening trial: support for breast cancer mortality as an end point. J Med Screen. 2002;9(4):159-62. doi: 10.1136/jms.9.4.159. PMID: 12518005.  Stiggelbout 2020 (The Netherlands and Australia) tested women’s understanding of the terms “false positive” and “overdiagnoses,” before asking what their tolerance was for overdiagnosis. But the information given to the women was incorrect: “Cancers like this may grow very slowly or just stay the same. Without screening, they would never be noticed or cause any trouble...doctors cannot be sure which cancers will be harmless. Therefore, treatment is recommended...”. They provided “explicit information on the nature of invasive treatments… (e.g., in mastectomy scenario 37% of women who correctly picked the overdetection description would always screen versus 52% of women who (incorrectly) picked the FP; 49% vs 58% for lumpectomy.”  The Schwartz 2000, USA is particularly troublesome. The wording in their Format & Definitions section is hugely misleading: in print survey questions; FP: “in a woman who gets mammogram annually for the next 10 years, one of her mammograms will look like she has BC even though she does not.” In fact, most recalls are for findings that are not highly suspicious. Some reporting systems allow the reporting radiologists to indicate the level of suspicion. But most tell women that most recalls are false alarms, and only a small fraction of women who are recalled are diagnosed with cancer.  Re: overdiagnosis, “We would like to ask your opinion about ductal carcinoma in situ or DCIS, a breast abnormality which can only be picked up by mammograms. Specialists are confused about DCIS because some-times it becomes invasive and sometimes it doesn’t. If DCIS does not become invasive, it will not affect how long a person will live even without treatment. Doctors don’t know which DCIS will become invasive. Nowadays, almost everyone with DCIS gets treated. Many people receive surgery, chemotherapy, or radiation who would never have gotten sick. For these people, treatment provides no physical benefit.”; no trade -offs for overdiagnosis or estimates provided  Under Relative importance for decision making, they state “Overdiagnosis: important for 60% (71% in 18-39 yr); not included in primary synthesis since no numerics provided or trade-offs elicited)” Why were women aged 18 included? Why were 25% of the women younger than 40? Overdiagnosis is vanishingly low in younger women. Was overdiagnosis explained just in the context of DCIS? Was it explained in terms of competing causes of death, like other cancers, heart disease, and other illnesses uncommon in younger women, but applicable to the elderly?  In Lewis, 2003, USA, they framed the communication piece in a manner that would increase anxiety among the participants. Women were surveyed about their beliefs about the importance of mortality reduction vs false positives. Then they were told in videos, “an abnormal mammogram when there is nothing actually wrong, but the result may require more tests or a biopsy to find out that there was no cancer; more than one third of women with a false positive continue to worry about having breast cancer.” There was no need to include the last phrase.  Women were surveyed again after watching the videos and there was no change in their beliefs. | Thank you taking the time to make these additional comments which we appreciate. We have numbered your comments into broad categories and have responded to each.   1. During our GRADE assessments we carefully interpreted the findings and are confident in our assessments. As per the study findings and our summary statements, it is not clear (and definitely not with high confidence) that across all ages and scenarios presented that “a vast majority” value the benefits as greater than the harms. In several cases the evidence suggests there will be at least some variability among individuals, even when the net benefit is portrayed as quite high. Further, as you rightly point out there are limitations across studies, such as what an how data was presented to women (all detailed in our tables) and how the potential outcomes were described conceptually and in respect of the effects from screening. Our certainty is reflective of our confidence in our summary statements, for example in how confident we were about the proportions of women that would have the same views. This does not suggest that the task force will ignore the results or consider preferences as less important. Indeed, the task force (as with any guideline panel) has to reflect on much evidence that is of low certainty and does not use this in isolation to support recommendations for or against screening. We have clarified in this manuscript that the findings of the review are one source of information on patient preferences. 2. We agree that the findings (especially for the indirect evidence used for findings in Table 4) relate to what information is provided in the decision aids or other educational/information materials. We agree that there are limitations from studies not reporting on all important outcomes or when descriptions of the outcomes are potentially biased. We had commented on the fact that few of the studies providing information about the effects of screening presented information on the possibility of lower treatment morbidity and avoiding some treatment such as chemotherapy. It is difficult to judge these studies too harsh for bias when there is limited high quality research (from comparing screened versus not screened populations rather than case series of patients with cancer) supporting these effects. We have added a comment on this limitation to the discussion but do not feel it changes our overall findings. We have interpreted the findings based on assessments of these limitations. For example, poor descriptions of overdiagnosis or only using relative effects for benefits on breast-cancer mortality deemed ratings of high risk of bias for studies, and the magnitudes of effects across the outcomes has considered when we made judgements about the net benefit of the data presented. One example is that for the Valentine study as you mention, the data from “intervention 4” (viewed as biased by only focusing on rare harms from a biopsy) was not relied upon at all when making our summary statements/conclusions. Of note, our assessments focused on the outcomes rated by the task force’s working group to be important/critical for decision making, and undertreatment from missing a cancer was included whereas all-cause mortality (even if effects are uncertain) was. Our review did not try to evaluate whether the effects presented were accurate/valid, but rather what the views were in light of the differences in the data presented. Several studies using data from observational or modelling studies indicated higher net benefit from screening and the results were compared with other studies using trial data (e.g. showing a lower net benefit) for their materials, regardless of whether we felt one set of data was more valid than the other. This is the main basis for synthesizing these findings. As indicated in our review (and GRADE guidance), the findings on the disutilities of the outcomes and from direct tradeoffs between outcomes are considered more relevant to this research questions and was weighed accordingly (for example by us rating down all evidence on attitudes/intentions from decision aids/educational materials as indirect because intentions to screen are based on a large number of factors apart from the numbers/outcomes). 3. Though we agree that all-cause mortality (within a specific timeframe) may be difficult to demonstrate (mainly due to imprecision) this does not necessarily prevent it from being an outcome that is important to assess even if to mainly comment on this uncertainty. Recommendations for screening for any age/population group is a clear indication that this outcome is not the sole factor relied upon for the overall direction of recommendations. We are confident in our assessments of the findings for the data in table 3 on trade-offs though cannot comment on how the task force will judge the effects from their review on screening effectiveness or on how much findings from this review contribute to their decision making. We recognize that the few studies that mentioned all-cause mortality did not clearly qualify this with a statement about the uncertainty. Regardless, we do not think this seriously impacts the main findings, for example that “for patients 50 or older, at least a large majority (>75%) of patients may think that reducing breast-cancer mortality is beneficial even if there is no impact on all-cause mortality.” We carefully examined the descriptions studies provided about the outcomes, and rated down when this was of serious concern, as it was for the Schwartz study. With working group input, our most serious concern for descriptions of overdiagnosis was when the diagnosis was not of a cancer but only a precancerous lesion or DCIS. We have added discussion points about the poor descriptions of false positives across several studies (most seriously when stating they are all suspicious for cancer). Because we rated our certainty in the findings (often rating down for these issues) and used broad categorizations about the findings (e.g. majority vs large majority) we are quite confident in the conclusions. As mentioned above, having to use low certainty evidence does not suggest that the task force will ignore findings or the importance of patient preferences. |
| McClure | No, thank you for allowing me to review this manuscript. Thank you for working on the updated guidelines. | Thank you. No changes requested. |
| Palmer | Blank | No changes requested. |
| Sehdev | Outstanding and comprehensive review | Thank you. No changes requested. |
| White | In 2019 I was diagnosed with locally advanced breast cancer with lymph node involvement. It was 2 weeks after my 43rd birthday, a few years earlier I asked my family doctor for a mammogram, and she refused citing the task force. I asked again but she said no.  I understand this is an academic process, that for many involved in this process it is routine, but to me this is personal. I could have received an earlier diagnosis, I could have saved myself aggressive chemotherapy that has aged me and 29 painful fractions of radiation.  The women who are included in these studies, they are not being told that this does happen in younger women. They don’t know that mammograms are mostly just uncomfortable and not painful and they certainly don’t know that a ‘false positive’ most often means returning for a quick and painless ultrasound or MRI to rule out cancer. Even if a needle biopsy is required, it is a quick outpatient procedure and not more painful than some dental visits.  I speak to friends and family and share my story and many end up struggling to access this care at 40 because the task force recommendations are held up as gold standard evidence by family doctors who don’t have time to read primary literature and who don’t realize that saving lives is being measured against transient anxiety.  Thank you for reading this statement and I hope you will include my words in the final version of the document. | Thank you very much for sharing your thoughts and experience. It is intended by the task force to make their recommendations more explicit/understandable as there was never the intent to prevent anyone in their 40s from being screened as long as they were well informed about the benefits and risks and chose to screen. With the updated recommendations the task force is preparing to undertake a fulsome evaluation through stakeholder and public comments and active usability testing with patients/public, to ensure the intent of their recommendations and related messaging is well understood.  Many of the included studies gave data on the expected rates of breast cancer for women in their age group. We have commented on the oftentimes insufficient description of false positives and agree this could definitely be improved upon in information provided to women when making decisions. Nevertheless, findings suggest quite a large number of false positives may be acceptable.  We had commented on the fact that few of the studies providing information about the effects of screening presented information on the possibility of lower treatment morbidity and avoiding some treatment such as chemotherapy. We have added a comment on this limitation to the discussion.  While we are not incorporating views from stakeholders directly into the manuscript your message from a patient perspective will be shared with task force |
| Bell | No | Thank you. No changes requested. |
| Buick | Blank | No changes requested. |
| Wittmer (peer reviewer) | One part that was not clear for me was the inclusion of articles submitted by stakeholders (literature flow). Given the fact that stakeholders may wish to know transparently what evidence was included versus not, I would find it helpful to see why stakeholder submissions were not retained (evidence already found through database searches ?), this does not seem to be commented in the paragraph on the literature flow. | Thanks for this request. We have added a comment on this to the manuscript, “All of the eligible studies submitted by stakeholders were also found in our searches.” |
