# Supplementary material for: Patient preferences for breast cancer screening: a systematic review update to inform recommendations by the Canadian Task Force on Preventive Health Care
Source: Syst Rev. 2024 May 28;13:140. doi: 10.1186/s13643-024-02539-8 (PMC11134964; doi:10.1186/s13643-024-02539-8)
Supplement: Supplementary file 2 — Supplementary Material 2. Data sets for studies reporting health-state utilities. [file 13643_2024_2539_MOESM2_ESM.docx]

**Patient preferences for breast cancer screening: a systematic review update** **to inform recommendations by the Canadian Task Force on Preventive Health Care**

Jennifer Pillay, Samantha Guitard, Sholeh Rahman, Sabrina Saba, Ashiqur Rahman, Liza Bialy, Nicole Gehring, Maria Tan, Alex Melton, Lisa Hartling; Alberta Research Centre for Health Evidence, Faculty of Medicine and Dentistry, University of Alberta, Edmonton, Alberta, Canada.

**Supplementary file 2.** **Data sets for studies reporting health-state utilities**

**CONTENTS**

Summary of Findings Tables, primary analysis (Screening states, Treatment states time 1 (≥12 months after surgery), Treatment states time 2 (≥2 years after surgery) p. 2

Time trade-off and standard gamble analysis (Screening states, Treatment states time 1, Treatment states time 2) p. 18

Study Characteristics p. 23

Summary of risk of bias assessments p. 41

Included studies p. 44

**Table S2.1. Summary of Findings: HSUVs, screening health states**

| **Number of included studies;**  **Sample size** | **Findings** | **GRADE^ꝉ^** | **What does the evidence say?** |
| --- | --- | --- | --- |
| **Healthy comparator (Before screening and negative screening results eligible) (FOR USE TO CALCULATE DISUTILITIES OF OTHER HEALTH STATES)** | | | |
| N=3 studies;  Tosteson 2014, Tran 2022, Zigman 2020  N=8,556  Alberta norms, N=17,914 | **Pooled utilities (95% CI):** 0.94 [0.93, 0.94]  *all moderate ROB  **One study (n=33) enrolled individuals attending genetic counselling testing (results not known), one negative screening result known (n=531; Utility (95% CI): 0.90 [0.89, 0.91]) and one healthy age and education matched comparators (n=7992; age matched to comparison: 46.3 (SD 9.6), 18+ years eligible)  ***No studies reported on utilities from before screening  **Alberta EQ-5D utility norms (across sexes but 45 to 74 year olds were similar between men and women for these age groups): adult females across all ages:** 0.85 (0.14)  **45-64 years:** 0.83 (0.16)  **65-74 years:** 0.82 (0.15)  **75+ years:** 0.79 (NR) | ⊕⊕⊕⊝  MODERATE  (for individuals 40-70 years, lack of consistency relying heavily on one study)  ⊕⊕⊝⊝  LOW  (for individuals 70+ years, lack of consistency and lack of directness in included studies compared with trend in Alberta norms) | The utility value for a healthy comparator, eligible for BC screening and aged 40-70 years is probably 0.94.  The utility value for a healthy comparator, eligible for BC screening and aged 70+ years may be 0.94, but there is less certainty for this value. |
| **Disutility of screening test process (after screening but before screening results)** | | | |
| No evidence | **Pooled utilities (95% CI):** no evidence | No evidence | No evidence |
| **Disutility of positive screening mammography (before diagnostic testing)** | | | |
| N=3 studies;  Domeyer 2010, Timmers 2014, Tosteson 2014  N=565 participants | **Pooled utilities (95% CI):** 0.87 [0.86, 0.89]  *Timmers 2014 (n=204) (0.86 utility) and Domeyer 2010 (n=102) (0.73) high ROB but Domeyer 2010 only contributed 8.1%  **Domeyer 2010 and Tosteson 2014 (0.90) excluded patients later diagnosed with BC; Timmers 2014 only included those with BiRADS 0 “low suspicion of cancer” which would have been communicated to the participants during informed consent.  In Tostenson, participants had enrolled in a large study of the accuracy of digital vs film mammography and consented to undergo additional mammograms thus may have been less concerned about getting positive mammograms.  **Disutility from healthy comparator (95% CI):** 0.94 [0.93, 0.94] – 0.87 [0.86, 0.89] = 0.07 [0.05, 0.09] | ⊕⊕⊕⊝  MODERATE  (some inconsistency and some risk of bias) | The disutility value for a positive screening mammography is probably 0.07. |
| **Disutility after biopsy (diagnostic results not known)** | | | |
| N=1 study;  Domeyer 2010  N=102 participants | **Pooled utilities (95% CI):** 0.79 [0.75, 0.83]  *all FP, no BC  **high ROB  **Disutility from healthy comparator (95% CI):** 0.94 [0.93, 0.94] – 0.79 [0.75, 0.83] = 0.15 [0.11, 0.19] | ⊕⊝⊝⊝  VERY LOW^b^  (single study-lack of consistency, risk of bias, and imprecision) | We are very uncertain about the disutility of receiving a biopsy, before the results are known. |
| **Disutility false positive requiring imaging only or imaging plus biopsy** | | | |
| N=2 studies;  Timmers 2014, Tosteson 2014  N=696 participants | **Pooled utilities (95% CI):** 0.90 [0.89, 0.91]  *Timmers 2014 (n=204) has high ROB  **one study is no BC: Toteson 2014, n=492 **1 yr from diagnostic workup, also has** **subgroup at baseline who received FP result soon after diagnostic workup (n=234; 0.91 (0.14)).** Other study, Timmers 2014 n=204 has some individuals that still need biopsy [had abnormal repeat mammogram] and those with normal diagnostic mammogram, so small percent likely with BC)  **Disutility from healthy comparator (95% CI):** 0.94 [0.93, 0.94] - 0.90 [0.89, 0.91] = 0.04 [0.03, 0.05] | ⊕⊕⊝⊝  LOW  (risk of bias, indirectness for concerns about applicability to duration follow-up, disutility might be slightly overestimated) | The disutility value for a false positive requiring imaging only or imaging plus biopsy may be 0.03 to 0.04. |
| **Disutility of false positive result requiring imaging plus biopsy** | | | |
| N=1 study;  Domeyer 2010  N=78 participants | **Pooled utilities (95% CI):** 0.77 [0.72, 0.82]  *all FP, no BC  **high ROB  **Disutility from healthy comparator (95% CI):** 0.94 [0.93, 0.94] – 0.77 [0.72, 0.82] = 0.17 [0.12, 0.22] | ⊕⊝⊝⊝  VERY LOW  (risk of bias, lack of consistency, imprecision) | We are very uncertain about the disutility of a false positive result after invasive testing, with results known. |
| **True positive result, before treatment** | | | |
| N=9 studies;  De Kok 2010, Haidairi 2022,  Min 2014, Miret 2023, Morgan 2021, Rautalin 2021, Ring 2021, Tran 2022, Zigman 2020  N=6,657 participants | **Pooled utilities (95% CI):** 0.86 [0.85, 0.86]  *2/9 studies are high ROB (Min 2014 [n=30] and Rautalin 2021 [n=1065]); removing high ROB studies utility: 0.85 [0.85, 0.85])  **Morgan and Ring aged 70+: 0.88 [0.87, 0.88]  ***Removing De Kok 2010 (1 day before surgery, weight 43.6%): 0.88 [0.88, 0.89]  ******All 9 studies are not specific to screen-detected cancers**  ******Of 7/9 studies that reported on stage, the range of proportions with stage IV was: 0-0.7%**  **Disutility from healthy comparator (95% CI):** 0.94 [0.93, 0.94] – 0.86 [0.85, 0.86] = 0.08 [0.07, 0.09] | ⊕⊕⊕⊝  MODERATE  (some concerns about inconsistency across studies and some indirectness based on within study age group data indicating differences across age, and no studies specific to screen-detected cancers [but few stage IV]) | The disutility of a screen-detected cancer is probably on average 0.08, but may be higher for older ages and advanced stage operable cancer. |
| Subgroup, stage 0 included:  N=3 studies;  N=1,875 participants  Subgroup, stage 0 not included:  N=2 studies;  N=3,344 participants  Subgroup, stage 0 not reported:  N=4 studies;  N=1,438 participants | **Subgroup, stage 0 included, utilities (95% CI):** 0.88 [0.87, 0.88]  *no studies high ROB  **Subgroup, stage 0 not included, utilities (95% CI):** 0.88 [0.87, 0.88]  *no studies high ROB  **Subgroup stage 0 not reported, utilities (95% CI):** 0.84 [0.84, 0.84]  *2 studies high ROB |  |  |
| Subgroup, age:  N=1 study;  N=1,200 participants (  40-65 years: n=878;  >65 years: n=322) | **Subgroup, age 40-65 years, utilities (95% CI):** 0.87 [0.86, 0.88]  **Subgroup, age >65 years, utilities (95% CI):** 0.84 [0.82, 0.86] |  |  |
| Subgroup stage 0:  N=1 study;  N=132 participants  Subgroup stage I-III:  N=3 studies;  N=4,488 | **Subgroup, stage 0, utilities (95% CI):** 0.88 [0.86, 0.90]  *study is not high ROB  **Subgroup, stage I-III, utilities (95% CI):** 0.87 [0.87, 0.88]  *no studies are high ROB  **none had stage IV |  |  |
| Subgroup stage 0-I:  N=1 study;  N=798  Subgroup stage II-III:  N=1 study;  N=478 | **Subgroup, stage 0-I, utilities (95% CI):** 0.86 [0.85, 0.87]  *study is not high ROB  **Subgroup, stage II-III, utilities (95% CI):** 0.85 [0.84, 0.86]  *study is not high ROB |  |  |
| Subgroup stage I:  N=1 study;  N=666  Subgroup stage II-III:  N= 1 study;  N=478 | **Subgroup, stage I, utilities (95% CI):** 0.86 [0.85, 0.87]  *study is not high ROB  **Subgroup, stage II-III, utilities (95% CI):** 0.85 [0.84, 0.86]  *study is not high ROB |  |  |
| Subgroup, stage 0-II:  N= 1 study;  N=1,184  Subgroup, stage III-IV:  N=2 studies;  N=124 participants | **Subgroup, stage 0-II, utilities (95% CI):** 0.86 [0.85, 0.87]  *study is not high ROB  **Subgroup, stage III-IV, utilities (95% CI):** 0.85 [0.82, 0.87]  *neither study is high ROB  **Miret 2023 (n=92) does not have stage IV |  |  |
| Subgroup, stage I-II:  N=2 studies;  N=1,233  Subgroup, stage III=IV:  N=2 studies;  N=124 participants | **Subgroup, stage I-II, utilities (95% CI):** 0.86 [0.85, 0.87]  **Subgroup, stage III-IV, utilities (95% CI):** 0.85 [0.82, 0.87] |  |  |
| **Interval cancer** | | | |
| N=1 study (using VAS);  Bonomi 2008  N=131 participants | Hypothetical scenarios in public sample found similar VAS utility scores for screen-detected new diagnoses versus an interval cancer: 45.7 (20.5) vs. 48.5 (20.7). Scenario for new diagnosis and interval cancer are quite similar, i.e. possibly more aggressive cancers.  Also see results for true positive, before treatment. | ⊕⊕⊝⊝  LOW  (some concerns about risk of bias, some concerns about inconsistency, and indirectness) | The disutility for interval cancer may be similar to a screen-detected cancer. |

ꝉ GRADE, Grading of Recommendations Assessment, Development and Evaluation. Reasons for rating down certainty: a=risk of bias, b=inconsistency/lack of consistency, c=indirectness, d=imprecision; use of capitals indicates there was very serious concern for the domain; for three exposures (positive screening mammography, true positive result, interval cancer) there was only some concern for two of the domains

BC, breast cancer; CI, confidence interval; FP, false positive; ROB, risk of bias; NR, not reported

**Table S2.2. Summary of Findings: HSUVs, treatment health states: T1≤12 months from surgery**

| **Number of included studies;**  **Sample size** | **Findings** | **GRADE^ꝉ^** | **GRADE overall** | **What does the evidence say?** |
| --- | --- | --- | --- | --- |
| **Disutility of mastectomy vs. BCS/partial mastectomy** | | | | |
| Within study:  N=3 studies;  Bromley 2019,  Moro-Valdezate 2014, Rautalin 2021  N=1,546 participants | **Pooled disutility, within study (95% CI):** 0.03 [0.02, 0.05]  *all high ROB  **studies had >20% receiving adjuvant therapies | ⊕⊕⊝⊝  Low (risk of bias, inconsistency) | ⊕⊕⊝⊝  Low (ROB and inconsistency between types of adjuvant therapy received as well as indication from direct measurements that disutility may be higher) | The disutility of a mastectomy versus a BCS/partial mastectomy (all patients receiving adjuvant treatments) may be at least 0.02 to 0.03.  We are very uncertain about the disutility from mastectomy without adjuvant treatment vs. BCS/partial mastectomy with radiation. |
|  | **Subgroup, by adjuvant therapies, disutility, within study (95% CI):**  BCS with mixed/unspecified adjuvant therapy vs. mastectomy with mixed/unspecified adjuvant therapy: 0.05 [-0.03, 0.13]  BCS with adjuvant chemotherapy vs mastectomy with adjuvant chemotherapy:  0.08 [0.05, 0.11]  BCS with adjuvant radiation vs mastectomy with mixed/unspecified adjuvant therapy (>50% receiving chemotherapy):  0.01 [-0.01, 0.03] |  |  |  |
| Between study:  BCS, N=5 studies;  Ali 2017, Bromley 2019, Moro-Valdezate 2014, Rautalin 2021, Youens 2019  N=1,682 participants  Mastectomy, N=7 studies;  Bromley 2019, Fujii 2019, Moro-Valdezate 2014, Park 2023, Rautalin 2021, Tran 2022, Velikova 2018  N=1,942 participants | **Pooled BCS utilities (95% CI):** 0.82 [0.81, 0.83]  *3/5 high ROB (Moro-Valdezate 2014 [n=364], Bromley 2019 [n=172], Rautalin 2021 [n=632])  **All receiving adjuvant therapies  **Pooled mastectomy utilities (95% CI):** 0.80 [0.79, 0.80]  *4/7 high ROB (Moro-Valdezate 2014 [n=141], Bromley 2019 [n=172], Rautalin 2021 [n=378], Velikova 2018 [n=776])  **All receiving adjuvant therapies  **Disutility, between study (95% CI):** 0.02 [0.01, 0.03] | ⊕⊕⊝⊝  Low  (inconsistency and risk of bias) |  |  |
|  | **Subgroup, by adjuvant therapies, utilities, between study (95% CI):**  BCS/partial mastectomy with adjuvant chemotherapy: 0.88 [0.86, 0.90]  *1/1 high ROB study (Moro-Valdezate 2014)  BCS/partial mastectomy with adjuvant radiation: 0.83 [0.82, 0.84]  *2/3 high ROB (Bromley 2019, Rautalin 2021)  BCS/partial mastectomy with mixed/unspecified adjuvant therapy: 0.76 [0.74, 0.77]  *1/3 high ROB (Bromley 2019)  BCS/partial mastectomy with no adjuvant therapy: no evidence  Mastectomy with adjuvant chemotherapy: 0.84 [0.83, 0.84]  *1/2 high ROB (Moro-Valdezate 2014)  Mastectomy with adjuvant radiation: 0.75 [0.73, 0.77]  *1 high ROB study (Velikova 2018)  Mastectomy with mixed/unspecified adjuvant therapy: 0.74 [0.73, 0.75]  *3/5 high ROB (Bromley 2019, Rautalin 2021, Velikova 2018)  Mastectomy with no adjuvant therapy: no evidence |  |  |  |
| **Disutility of adjuvant chemotherapy vs. none** | | | | |
| Within study:  N=2 studies;  Hall 2015, Ring 2021  N=1,011 participants | **No meta-analysis**  **Ring 2021, n=780 (95% CI):** -0.01 [-0.04, 0.02]  *high ROB  **Hall 2015, n=231 (95% CI):** 0.76 [0.73, 0.79] - 0.75 [0.71, 0.79] = 0.01 (NR)  *moderate ROB  **no SD or sample size in each arm  ***Both studies were among mixed surgery/unspecified; and were among mixed radiation (21-79%)/unspecified  ****Subgroup analysis by type of chemotherapy not possible | ⊕⊕⊝⊝  Low  (risk of bias and imprecision)  For little-to no difference in utility | ⊕⊕⊝⊝  Low (inconsistency, indirectness) | The disutility of adjuvant chemotherapy may be 0.02-0.04 among a mixed surgical population. |
|  | **Subgroup, by surgery, disutilities, within study (95% CI):** none (both studies were among mixed surgery/unspecified) |  |  |  |
|  | **Subgroup, by adjuvant radiation, disutilities, within study (95% CI):** none (both studies mixed radiation (21-79%)/unspecified) |  |  |  |
|  | **Subgroup, including stage 0, utilities, within study (95% CI):**  Adjuvant chemotherapy vs. none with stage 0 excluded: **Ring 2021, n=780 (95% CI):** 0.01 [-0.04, 0.02]  *high ROB  Adjuvant chemotherapy vs. none with stage 0 NR: **Hall 2015, n=231 (95% CI):** 0.76 [0.73, 0.79] - 0.75 [0.71, 0.79] = 0.01 (NR)  *moderate ROB |  |  |  |
|  | **Subgroup, including stage III-IV, utilities, within study (95% CI):**  Adjuvant chemotherapy vs. none with stage III-IV NR:  **Ring 2021, n=780 (95% CI):** 0.01 [-0.04, 0.02]  *high ROB  Adjuvant chemotherapy vs. none with stage III-IV NR:  **Hall 2015, n=231 (95% CI):** 0.76 [0.73, 0.79] - 0.75 [0.71, 0.79] = 0.01 (NR)  *moderate ROB |  |  |  |
| Between study:  Adjuvant chemotherapy:  N=7 studies;  Hall 2015, May 2017, Moro-Valdezate 2014, Ring 2021, Roine 2020, Tanaka 2019, Tran 2022  N=1,234 participants (1 study N=NR by arm, N=231 overall)  No adjuvant chemotherapy:  N=5 studies;  Hall 2015, Kim 2015, Morgan 2021, Rautalin 2018, Ring 2021  N=2,447 participants (1 study N=NR by arm, N=231 overall) | **Pooled adjuvant chemotherapy utilities (95% CI): 0.85 [0.84, 0.85]**  *2/7 high ROB (Moro-Valdezate 2014 [n=364], Ring 2021 [n=224]), **without high ROB studies:** 0.85 [0.84, 0.86] ROB subgroup effects: p=0.24 vs high ROB studies: 0.84 [0.83, 0.85]  **7 studies, mastectomy (2) or mixed (5) surgery  **Pooled no adjuvant chemotherapy utilities (95% CI):** 0.84 [0.83, 0.84]  *2/5 high ROB (Morgan 2021 [n=1,477], Ring 2021 [n=556]), **without high ROB studies:** **0.87 [0.86, 0.88]** ROB subgroup effects: p<0.00001 vs high ROB studies: 0.83 [0.82, 0.83]  Disutility, between study (95% CI): -0.01 [-0.02, -0.00]  **Disutility, removing high ROB studies (95% CI): 0.02 [0.01, 0.03]**  *Disutility without high ROB studies (no chemotherapy), 3 studies, n=646  **one study, Hall 2015, does not report numbers in each arm (chemo Y vs N), only gives utility+SE (no SD), n=231 overall (did not add this study sample size to the disutility calculation)  ***Subgroup analysis by type of chemotherapy not possible  **Outlier effects (lower/higher values) did not appear to relate to timepoint used in studies** | ⊕⊕⊝⊝  Low (inconsistency [unexplained by subgroups], indirectness [comparisons])  For disutility of 0.02 from chemotherapy in a mixed surgical population. Subgroup findings indicated slightly more disutility when removing effects from radiation. |  |  |
|  | **Subgroup, by surgery, utilities, between study (95% CI):**  **Chemotherapy with BCS/partial mastectomy: 0.88 [0.86, 0.90] *1 study high ROB (Moro-Valdezate 2014) vs. No chemotherapy with BCS/partial mastectomy: no evidence**  **Chemotherapy with mastectomy: 0.84 [0.83, 0.84] *1/2 studies high ROB (Moro-Valdezate 2014) vs. No chemotherapy with mastectomy: no evidence**  Chemotherapy with mixed/unspecified surgery: 0.86 [0.85, 0.87] *1/5 studies high ROB (Ring 2021) vs. No chemotherapy with mixed/unspecified surgery: 0.84 [0.83, 0.84] *2/5 studies were high ROB (Morgan 2021, Ring 2021) |  |  |  |
|  | **Subgroup, by adjuvant radiation use, utilities, between study (95% CI):**  Adjuvant chemotherapy with >80% adjuvant radiation: 0.84 [0.84, 0.85]  *1/2 studies high ROB (Moro-Valdezate 2014 [n=364] vs No adjuvant chemotherapy with >80% adjuvant radiation: no evidence  Adjuvant chemotherapy with mixed adjuvant radiation (21-79%): 0.88 [0.87, 0.89]  *no studies high ROB (2 studies) vs No adjuvant chemotherapy with mixed adjuvant radiation (21-79%): no evidence  **Adjuvant chemotherapy with no adjuvant radiation (0 to <20%): 0.82 [0.76, 0.88]**  ***1 study, is not high ROB (Tanaka 2019, n=38; mixed surgery; 0% stage 0 and 26% stage III/IV) vs. No adjuvant chemotherapy with no adjuvant radiation (0 to <20%): 0.86 [0.84, 0.88] *1 study, is not high ROB (Rautalin 2018 n=268; mixed surgery; % stage 0 or III/IV NR)**  Adjuvant chemotherapy with adjuvant radiation use NR: 0.80 [0.78, 0.82]  *1/2 studies high ROB (Ring 2021 [n=224] vs. No adjuvant chemotherapy with adjuvant radiation use NR: 0.84 [0.83, 0.84]  *2/4 studies high ROB (Morgan 2021 [n=1,477], Ring 2021 [n=556]) |  |  |  |
|  | **Subgroup, including stage 0, utilities, between study (95% CI):**  Adjuvant chemotherapy with stage 0 included (>20%): no evidence vs. No adjuvant chemotherapy with stage 0 included (>20%): no evidence  Adjuvant chemotherapy with stage 0 included (% NR): 0.84 [0.83, 0.85] *1 study, not high ROB vs. No adjuvant chemotherapy with stage 0 included (% NR): 0.89 [0.88, 0.90] *1 study, not high ROB  Adjuvant chemotherapy with stage 0 excluded (0 to <20%): 0.87 [0.86, 0.87] *2/4 studies high ROB (Moro-Valdezate 2014 [n=364], Ring 2021 [n=224]) vs. No adjuvant chemotherapy with stage 0 excluded (0 to <20%): 0.83 [0.82, 0.83]  *Both studies high ROB (Morgan 2021 [n=1,477], Ring 2021 [n=556])  Adjuvant chemotherapy with stage 0 NR: 0.77 [0.74, 0.79] *no studies high ROB (2 studies) vs. No adjuvant chemotherapy with stage 0 NR: 0.83 [0.81, 0.85]  *Neither study high ROB (2 studies) |  |  |  |
|  | **Subgroup, including stage III-IV, utilities, between study (95% CI) (stage IV <10% in all):**  **Adjuvant chemotherapy with stage III-IV included (>20%):**  **0.82 [0.76, 0.88] *1 study**, not high ROB (18.4% stage III, 7.9% stage IV) vs.  No adjuvant chemotherapy with stage III-IV included (>20%): no evidence  Adjuvant chemotherapy with stage III-IV included (% NR):  0.89 [0.88, 0.90] *1 study, not high ROB vs.  No adjuvant chemotherapy with stage III-IV included (% NR): no evidence  Adjuvant chemotherapy with stage III-IV excluded (0-<20%): 0.84 [0.84, 0.85] *1/2 studies high ROB (Moro-Valdezate 2014 [n=364]) vs No adjuvant chemotherapy with stage III-IV excluded: no evidence  **Adjuvant chemotherapy with stage III-IV NR: 0.79 [0.78, 0.81] *1/3 studies high ROB (Ring 2021 [n=224]) vs. No adjuvant chemotherapy with stage III-IV NR: 0.84 [0.83, 0.84] *2/5 studies high ROB (Morgan 2021 [n=1,477], Ring 2021 [n=556])** |  |  |  |
| **Disutility of adjuvant radiation vs. none** | | | | |
| Within study:  N=4 studies;  Bromley 2019, Hall 2015, Velikova 2018, Youens 2019  N=1,587 participants | **Disutility, within study (95% CI):** 0.01 [-0.01, 0.02]  *2/3 studies high ROB (Bromley 2019 [n=172] (0.08), Velikova 2018 [n=776]); removing high ROB  -0.01 [-0.02, 0.01] **(one study 0.08)**  **Hall 2015, n=231 (95% CI):** 0.76 [0.73, 0.79] - 0.76 [0.72, 0.80] = 0.00 (NR)  *moderate ROB | ⊕⊕⊕⊝  Moderate (inconsistency)  For little-to no difference in utility | ⊕⊕⊕⊝  Moderate (inconsistency) | There is probably little-to-no disutility from adjuvant radiation among those receiving BCS/partial mastectomy or mastectomy, where many are receiving chemotherapy. |
|  | **Subgroup, by surgery, disutility, within study (95% CI):**  Adjuvant radiation vs. none with BCS/partial mastectomy: 0.01 [-0.01, 0.03]  *1/2 studies high ROB (Bromley 2019)  Adjuvant radiation vs. none with mastectomy: 0.00 [-0.04, 0.04]  *1 study high ROB (Velikova 2018)  Adjuvant radiation vs. none with mixed surgery/unspecified:  **Hall 2015, n=231 (95% CI):** 0.76 [0.73, 0.79] - 0.76 [0.72, 0.80] = 0.00 (NR)  *moderate ROB |  |  |  |
| Between study:  Adjuvant radiation:  N=8 studies;  Ali 2017, Bromley 2019, Hall 2015, Moro-Valdezate 2014, Rautalin 2021, Tran 2022, Velikova 2018, Youens 2019  N= 2,174 participants (1 study N=NR by arm, N=231 overall)  No adjuvant radiation:  N=8 studies;  Bromley 2019, Fujii 2019, Gordon 2017, Hall 2015, Rautalin 2021, Tanaka 2019, Velikova 2018, Youens 2019  N=1,547 participants | **Pooled adjuvant radiation utilities (95% CI):** 0.83 [0.82, 0.83]  *4/8 studies high ROB (Bromley 2019 [n=172], Moro-Valdezate 2014 [n=223], Rautalin 2021 [n=632], Velikova 2018 [n=388]), removing high ROB studies: 0.83 [0.82, 0.83]  **After removing studies reporting 80% or more chemotherapy used: **0.80 [0.79, 0.81]**  **Pooled no adjuvant radiation utilities (95% CI):** 0.81 [0.80, 0.82]  *2/8 studies high ROB (Bromley 2019 [n=172], Velikova 2018 [n=388]), removing high ROB studies: 0.81 [0.80, 0.82]  **After removing studies reporting 80% or more chemotherapy used: **0.81 [0.80, 0.82]**  **Disutility, between study (95% CI): -0.02 [-0.03, -0.01]**  **Disutility, removing studies reporting 80% or more chemotherapy used (95% CI):** **0.01 [-0.00, 0.02]**  *one study, Hall 2015, does not report numbers in each arm (radiation Y vs N), only gives utility+SE (no SD), n=231 overall (did not add this study sample size to the disutility calculation)  **Of 5/8 studies reporting on adjuvant chemotherapy, ≥49.0% received it | ⊕⊕⊝⊝  Low (very serious inconsistency unexplained by type of surgery and chemotherapy) |  |  |
|  | **Subgroup, by surgery, utilities, between study (95% CI):**  Radiation with BCS/partial mastectomy: 0.82 [0.82, 0.83] *3/5 studies are high ROB (Bromley 2019, Moro-Valdezate 2014, Rautalin 2021) vs. No radiation with BCS/partial mastectomy: 0.79 [0.78, 0.81] *1/2 studies high ROB (Bromley 2019)  Radiation with mastectomy: 0.83 [0.83, 0.84] *1/2 studies high ROB (Velikova 2018) vs. No radiation with mastectomy: 0.80 [0.78, 0.82] *1/2 studies high ROB (Velikova 2018)  **Radiation with mixed/unspecified surgery: 0.76 [0.60, 0.92] *1 study moderate ROB (Hall 2015)** vs. No radiation with mixed/unspecified surgery: 0.83 [0.81, 0.84]  *no studies high ROB (4 studies) |  |  |  |
| **Disutility of ALND vs. SLND** | | | | |
| Within study:  No evidence | **Disutility, within study (95% CI):** no evidence | No evidence | No evidence | No evidence |
| Between study:  N=1 study;  Moro-Valdezate 2014  N=364 participants | **Pooled ALND utilities (95% CI):** 0.85 [0.84, 0.86]  *high ROB  **Pooled SLNB utilities (95% CI):** no evidence  **Disutility, between study (95% CI):** no evidence | No evidence |  |  |
|  | **Subgroup, by surgery, utilities, between study (95% CI) only data for those receiving ALND:**  ALND with BCS/partial mastectomy: 0.88 [0.86, 0.90]  ALND with mastectomy: 0.80 [0.78, 0.82]  ALND with mixed/unspecified surgery: no evidence  * Comparing with data BCS surgery wasn’t valid since we couldn’t assume that these patients were not receiving ALND |  |  |  |
| **Disutility of advanced stage vs. not advanced stage (Stage II-III vs. I)** | | | | |
| Within study:  N=2 studies;  Criscitiello 2021, Porciello 2020  N=1,412 participants | **Disutility, within study (95% CI):** 0.02 [0.01, 0.03]  *neither study high ROB  ****neither study includes stage 0 or IV**  ***Both studies in a mixed surgery/unspecified with mixed adjuvant therapy | ⊕⊕⊝⊝  Low (lack of consistency due to 88% weight of one study, imprecision) | | There may be a disutility of 0.02, from having stage II-III vs. I among a mixed surgical and adjuvant treatment population. |
|  | **Subgroup, by surgery and adjuvant therapy, disutilities, within study (95% CI):** none |  |  |  |
| **Disutility of advanced stage vs. not advanced stage (Stage III vs. I-II)** | | | | |
| Within study:  N=2 studies;  Criscitiello 2021, Porciello 2020  N=1,412 participants | **Disutility, within study (95% CI):** 0.03 [0.02, 0.05]  *neither study high ROB  ****neither study includes stage 0 or IV**  *** Both studies in a mixed surgery/unspecified with mixed adjuvant therapy | ⊕⊕⊝⊝  Low (lack of consistency due to 71% weight of one study, imprecision) | | There may be a disutility of 0.03, from having stage III vs. I-II among a mixed surgical and adjuvant treatment population. |
|  | **Subgroup, by surgery and adjuvant therapy, disutilities, within study (95% CI):** none |  |  |  |

ꝉ GRADE, Grading of Recommendations Assessment, Development and Evaluation.

BC, breast cancer; BCS, breast-conserving surgery; CI, confidence interval; FP, false positive; ROB, risk of bias; N, no; NR, not reported; Y, yes

**Table S2.3. Summary of Findings: HSUVs, treatment health states: T2≥24 months from surgery**

| **Number of included studies;**  **Sample size** | **Findings** | **GRADE** ^ꝉ^ | **GRADE overall** | **What does the evidence say?** |
| --- | --- | --- | --- | --- |
| **Disutility of Mastectomy vs. BCS/partial mastectomy** | | | | |
| Within study:  N=5 studies;  Hanson 2022, Kouwenberg 2020, Lagendijk 2018(a), Rautalin 2021, Swanick 2018  N=3,820 participants | **Disutility, within study (95% CI):** 0.00 [-0.01, 0.01]  *2/5 studies high ROB (Hanson 2022 [n=510], Rautalin 2021 [n=964]), removing high ROB studies: 0.00 [-0.01, 0.02]  **4/5 studies >5 years from surgery | ⊕⊕⊕⊝  Moderate (indirectness of mixed therapies)  Little-to-no disutility from mastectomy | ⊕⊕⊕⊝  Moderate (indirectness of mixed therapies) | There is probably little-to-no disutility from mastectomy vs. BCS/partial mastectomy with radiation >2 years from surgery. |
|  | **Subgroup, by adjuvant therapies, disutilities, within study (95% CI):**  BCS/partial mastectomy with adjuvant radiation vs. mastectomy with adjuvant radiation: 0.09 [0.02, 0.16]  BCS/partial mastectomy with radiation vs. mastectomy with mixed/unspecified adjuvant therapy: -0.00 [-0.01, 0.01] (5 studies) |  |  |  |
| Between study:  BCS, N=6 studies;  Hanson 2022, Kouwenberg 2020, Lagendijk 2018(a), Lagendijk 2018(b), Rautalin 2021, Swanick 2018, Tran 2022  N=2,017 participants  Mastectomy, N=6 studies;  Hanson 2022, Kouwenberg 2020, Lagendijk 2018(a), Rautalin 2021, Swanick 2018, Velikova 2018  N=2,702 participants | **Pooled BCS utilities (95% CI):** 0.89 [0.88, 0.89]  *3/6 studies high ROB (Tran 2022 [n=124], Hanson 2022 [n=288], Rautalin 2021 [n=596]), removing high ROB studies: **0.84 [0.83, 0.85]**  **5/6 studies >5 years from surgery  **Pooled mastectomy utilities (95% CI):** 0.86 [0.85, 0.86]  *2/6 studies high ROB (Hanson 2022 [n=222], Rautalin 2021 [n=368]), removing high ROB studies: **0.83 [0.82, 0.84]**  **4/6 studies >5 years from surgery  **Disutility, between study (95% CI):** 0.03 [0.02, 0.04]  **Disutility, excluding high ROB studies (95% CI):** 0.01 [-0.00, 0.02]  **Disutility without high ROB studies has 4 studies (BCS), n=1,009 vs. 4 studies (mastectomy), n=2,112 | ⊕⊕⊕⊝  Moderate (indirectness across different adjuvant treatments) |  |  |
|  | **Subgroup, by adjuvant therapies, utilities, between study (95% CI):**  BCS/partial mastectomy with adjuvant chemotherapy: 0.92 [0.91, 0.93]  *1 study high ROB (Tran 2022) vs. Mastectomy with adjuvant chemotherapy: no evidence  **BCS/partial mastectomy with adjuvant radiation: 0.86 [0.86, 0.87]**  *2/6 studies high ROB (Hanson 2022, Rautalin 2021), removing high ROB studies: 0.84 [0.83, 0.85] vs. Mastectomy with adjuvant radiation: 0.77 [0.75, 0.79] *neither study high ROB (2 studies)  BCS/partial mastectomy with mixed/unspecified adjuvant therapy: no evidence vs. **Mastectomy with mixed/unspecified adjuvant therapy: 0.86 [0.86, 0.86]** *1/6 studies high ROB (Hanson 2022)  BCS/partial mastectomy with no adjuvant therapy: 0.85 [0.80, 0.90]  *1 study high ROB (Swanick 2018) vs. Mastectomy with no adjuvant therapy: no evidence |  |  |  |
| **Disutility of adjuvant chemotherapy vs none.** | | | | |
| Within study: no evidence | **Disutility, within study (95% CI):** no evidence | No evidence | ⊕⊝⊝⊝  Very low (very serious imprecision, lack of consistency) | We are very uncertain about the disutility of adjuvant chemotherapy vs none >2 years from surgery. |
| Between study:  Adjuvant chemotherapy:  N=2 studies;  Roine 2020, Tran 2022  N=272 participants  No adjuvant chemotherapy:  N=1 study;  Swanick 2018  N=278 participants | **Pooled adjuvant chemotherapy utilities (95% CI):** 0.91 [0.91, 0.92]  *1/2 studies are high ROB (Tran 2022 [n=124])  **1/2 studies >5 years from surgery  **Pooled no adjuvant chemotherapy utilities (95% CI):** 0.86 [0.84, 0.88]  *study is not high ROB  ** The 1 study is >5 years from surgery  **Disutility, between study (95% CI):** -0.05 [-0.07, -0.03] | ⊕⊝⊝⊝  Very low (very serious imprecision, lack of consistency) |  |  |
|  | **Subgroup, by surgery, utilities, (95% CI):**  Chemotherapy with BCS/partial mastectomy: 0.92 [0.91, 0.93] *1 study high ROB (Tran 2022) vs. No chemotherapy with BCS/partial mastectomy: 0.86 [0.84, 0.88] *study is not high ROB  Chemotherapy with mastectomy: no evidence vs. No chemotherapy with mastectomy: no evidence  Chemotherapy with mixed/unspecified surgery: 0.90 [0.89, 0.91]  *study is not high ROB vs. No chemotherapy with mixed/unspecified surgery: no evidence |  |  |  |
| Subgroup, age:  N=1 study;  Roine 2020  N=342 (NR by age subgroup) | **Subgroup, by age ≤50 years, utilities (95% CI):** 0.91 [0.90, 0.92]  **Subgroup, by age >50 years, utilities (95% CI):** 0.90 [0.88, 0.92]  *study is not high ROB |  |  |  |
| **Disutility of adjuvant radiation vs. none** | | | | |
| Within study:  N=2 studies;  Swanick 2018, Velikova 2018  N=1,183 participants | **Disutility, within study (95% CI):** -0.00 [-0.03, 0.03]  *no studies high ROB  **1/2 studies are >5 years from surgery | ⊕⊕⊕⊝  Moderate (imprecision)  Little-to-no disutility from adjuvant radiation vs none. | ⊕⊕⊕⊝  Moderate (inconsistency)  Little-to-no disutility from adjuvant radiation vs none. | There is probably little-to-no disutility from adjuvant radiation vs none >2 years from surgery. |
|  | **Subgroup, by surgery, disutilities (95% CI):**  Adjuvant radiation vs. none with BCS/partial mastectomy: -0.02 [-0.08, 0.04]  Adjuvant radiation vs. none with mastectomy: 0.01 [-0.02, 0.04]  Adjuvant radiation vs. none with mixed surgery/unspecified: no evidence |  |  |  |
| Between study:  Adjuvant radiation: N=9 studies;  Hanson 2022, Kouwenberg 2020, Lagendijk 2018(a), Lagendijk 2018(b), Moshina 2022, Rautalin 2021, Swanick 2018, Tran 2022, Velikova 2018  N=5,646 participants  No adjuvant radiation: N=4 studies;  Hanson 2022, Lagendijk 2018(a), Swanick 2018, Velikova 2018  N=838 participants | **Pooled adjuvant radiation utilities (95% CI):** 0.83 [0.83, 0.83]  *3/9 high ROB (Hanson 2022 [n=288], Rautalin 2021 [n=596], Tran 2022 [n=124]), removing high ROB studies: **0.80 [0.80, 0.80]**  **6/9 studies are >5 years from surgery  **Pooled no adjuvant radiation utilities (95% CI):** 0.86 [0.86, 0.87]  *1/4 high ROB (Hanson 2022 [n=222]), removing high ROB study: **0.81 [0.79, 0.83]**  **3/4 studies are >5 years from surgery  **Disutility, between study (95% CI):** 0.03 [0.02, 0.04]  **Disutility, excluding high ROB studies:** 0.01 [-0.01, 0.03]  *Disutility without high ROB studies has 3 studies (no radiation), n=616 vs. 6 studies (radiation), n=4,638 | ⊕⊕⊕⊝  Moderate (unexplained inconsistency)  Little-to-no disutility from adjuvant radiation vs none. |  |  |
|  | **Subgroup, by surgery, utilities (95% CI):**  Radiation with BCS/partial mastectomy: 0.89 [0.88, 0.89]  *3/7 studies high ROB (Tran 2022, Hanson 2022, Rautalin 2021) vs. No radiation with BCS/partial mastectomy: 0.85 [0.80, 0.90] *study is not high ROB  Radiation with mastectomy: 0.77 [0.75, 0.79] *neither study is high ROB vs. No radiation with mastectomy: 0.86 [0.86, 0.87]  *1/4 studies high ROB (Hanson 2022)  Radiation with mixed/unspecified surgery: 0.79 [0.79, 0.79]  *study is not high ROB vs. No radiation with mixed/unspecified surgery: no evidence |  |  |  |
| **Disutility of ALND vs. SLND** | | | | |
| Within study: no evidence | **Disutility, within study (95% CI):** no evidence | No evidence | No evidence | No evidence |
| Between study:  N=1 study;  Swanick 2018  N=102 | **Pooled ALND utilities (95% CI):** 0.78 [0.73, 0.83]  *not a high ROB study  **>5 years from surgery  **Pooled SLND utilities (95% CI):** no evidence  **Disutility, between study (95% CI):** no evidence | No evidence |  |  |
|  | **Subgroup, by surgery, utilities, between study (95% CI) only data for those receiving ALND:**  ALND with mastectomy: 0.78 [0.73, 0.83] |  |  |  |
| **Disutility of Advanced stage vs. not advanced stage** | | | | |
| No evidence | **Disutility, within study (95% CI):** no evidence | No evidence | No evidence | No evidence |

ꝉ GRADE, Grading of Recommendations Assessment, Development and Evaluation. Reasons for rating down certainty: a=risk of bias, b=inconsistency/lack of consistency, c=indirectness, d=imprecision; use of capitals indicates there was very serious concern for the domain; for three exposures (positive screening mammography, true positive result, interval cancer) there was only some concern for two of the domains

ALND, axillary lymph node dissection; BC, breast cancer; CI, confidence interval; ROB, risk of bias; SLND, sentinel lymph node dissection; NR, not reported

**Table S2.4. Evidence from direct elicitation methods (Time trade-off and standard gamble), screening health states**

| **Number of included studies;**  **Sample size** | **Findings** |
| --- | --- |
| **Healthy comparator (Before screening and negative screening results eligible) (FOR USE TO CALCULATE DISUTILITIES OF OTHER HEALTH STATES)** | |
| No evidence | **Pooled utilities for SG or TTO (95% CI):** no evidence |
| **Disutility of screening test process (after screening but before screening results)** | |
| No evidence | **Pooled utilities for SG or TTO (95% CI):** no evidence |
| **Disutility of positive screening mammography (before diagnostic testing)** | |
| No evidence | **Pooled utilities for SG or TTO (95% CI):** no evidence |
| **Disutility after biopsy (diagnostic results not known)** | |
| No evidence | **Pooled utilities (95% CI):** no evidence |
| **Disutility false positive requiring imaging only or imaging plus biopsy** | |
| No evidence | **Pooled utilities (95% CI):** no evidence |
| **Disutility of false positive result requiring imaging plus biopsy** | |
| No evidence | **Pooled utilities (95% CI):** no evidence |
| **True positive result, before treatment** | |
| N=1 study;  Schleinitz 2006  N=156 | **Utility (95% CI):** 0.53 [0.52, 0.54]  **Utility, excluding stage IV (95% CI):** 0.62 [0.61, 0.63]  *SG, hypothetical  **moderate ROB  **Disutility from healthy comparator (95% CI):** no evidence |
|  | **Subgroup, stage I, utilities (95% CI):** 0.68 [0.67, 0.69]  **Subgroup, stage II/III, utilities (95% CI):** 0.59 [0.58, 0.60] |
|  | **Subgroup, stage I/II, utilities (95% CI):** 0.65 [0.64, 0.66]  **Subgroup, stage III, utilities (95% CI):** 0.56 [0.55, 0.57] |
|  | **Subgroup, age <50 years, utilities (95% CI):** 0.58 [0.57, 0.59]  **Subgroup, age ≥50 years, utilities (95% CI):** 0.49 [0.47, 0.51] |
|  | **Subgroup, white race, utilities (95% CI):** 0.59 [0.57, 0.61]  **Subgroup, black race, utilities (95% CI):** 0.47 [0.45, 0.49]  **Subgroup, other race, utilities (95% CI):** 0.56 [0.51, 0.61] |
|  | **Subgroup, family history of breast cancer, utilities (95% CI):** 0.56 [0.54, 0.58]  **Subgroup, no family history of breast cancer, utilities (95% CI):** 0.51 [0.49, 0.53] |

CI, confidence interval; ROB, risk of bias; SG, standard gamble; TTO, time trade-off

**Table S2.5. Evidence from direct elicitation methods (Time trade-off and standard gamble), treatment health states: T1≤12 months from surgery**

| **Number of included studies;**  **Sample size** | **Findings** |
| --- | --- |
| **Disutility of mastectomy vs. BCS/partial mastectomy** | |
| Within study:  N=2 studies;  Bromley 2019, Knuttel 2017  N=293 participants | **Pooled disutility, within study (95% CI):** 0.05 [0.04, 0.06]  *both high ROB  ** Bromley 2019 is SG, Knuttel 2017 is TTO |
|  | **Subgroup, by adjuvant therapies, disutility, within study (95% CI):**  BCS/partial mastectomy with adjuvant radiation vs. Mastectomy with mixed/unspecified adjuvant therapy: 0.05 [0.04, 0.06]  *both studies high ROB  BCS/partial mastectomy without radiation vs. Mastectomy with mixed/unspecified adjuvant therapy: 0.16 [0.10, 0.22]  *study is high ROB |
| Between study:  BCS, N= 3 studies;  Bromley 2019, Knuttel 2017, Songtish 2014  N= 403 participants  Mastectomy, N=2 studies;  Bromley 2019, Knuttel 2017  N=293 participants | **Pooled BCS utilities (95% CI):** 0.79 [0.78, 0.80]  *2/3 studies high ROB  **Pooled mastectomy utilities (95% CI):** 0.69 [0.67, 0.70]  *2/2 studies high ROB  **Disutility, between study (95% CI):** 0.10 [0.08, 0.12] |
|  | **Subgroup, by adjuvant therapies, utilities (95% CI):**  BCS/partial mastectomy with adjuvant chemotherapy: no evidence vs. Mastectomy with adjuvant chemotherapy: no evidence  BCS/partial mastectomy with adjuvant radiation: 0.80 [0.79, 0.81] *both studies high ROB vs. Mastectomy with adjuvant radiation: no evidence  BCS/partial mastectomy with mixed/unspecified adjuvant therapy: 0.78 [0.76, 0.79] *1/2 studies high ROB vs. Mastectomy with mixed/unspecified adjuvant therapy: 0.69 [0.67, 0.70] *both studies high ROB |
|  | **Subgroup, by stage, utility (95% CI):**  BCS stage 0/I: 0.95 [0.93, 0.97] *study is high ROB vs. Mastectomy stage 0/I: 0.88 [0.84, 0.92] *study is high ROB  BCS stage II/II/IV: 0.90 [0.86, 0.94] *study is high ROB vs. Mastectomy stage II/III/IV: 0.90 [0.86, 0.94] *study is high ROB |
|  | **Subgroup, by age, utilities (95% CI):**  BCS, age <55 years: 0.90 [0.87, 0.93] *study is high ROB vs. Mastectomy, age <55 years: 0.90 [0.87, 0.93] *study is high ROB  BCS, age ≥55 years: 0.90 [0.86, 0.94] *study is high ROB vs. Mastectomy, age ≥55 years: 0.83 [0.79, 0.87] *study is high ROB |
| **Disutility of adjuvant chemotherapy vs. none** | |
| Within study: No evidence | **Pooled disutility, within study (95% CI):** no evidence |
| Between study:  Adjuvant chemotherapy, N=1 study;  Schleinitz 2006  N=156  No adjuvant chemotherapy: no evidence | **Pooled adjuvant chemotherapy utilities (95% CI):** 0.48 [0.47, 0.49]  *moderate ROB  **Pooled no adjuvant chemotherapy utilities (95% CI):** no evidence  **Disutility, between study (95% CI):** no evidence |
|  | **Subgroup, by surgery, utilities (95% CI):**  Adjuvant chemotherapy with mixed/unspecified surgery: 0.48 [0.47, 0.49] *moderate ROB vs. No adjuvant chemotherapy with mixed/unspecified surgery: no evidence |
|  | **Subgroup, by age, utilities (95% CI):**  Adjuvant chemotherapy, age <55 years: 0.48 [0.46, 0.50] *moderate ROB  Adjuvant chemotherapy, age ≥55 years: 0.48 [0.46, 0.50] *moderate ROB |
|  | **Subgroup, by race, utilities (95% CI):**  Adjuvant chemotherapy, White race: 0.53 [0.51, 0.55] *moderate ROB  Adjuvant chemotherapy, Black race: 0.45 [0.42, 0.48] *moderate ROB  Adjuvant chemotherapy, other race: 0.40 [0.34, 0.46] *moderate ROB |
|  | **Subgroup, by family history of breast cancer, utilities (95% CI):**  Adjuvant chemotherapy, family history of breast cancer: 0.47 [0.45, 0.49] *moderate ROB  Adjuvant chemotherapy, no family history of breast cancer: 0.50 [0.48, 0.52] *moderate ROB |
| **Disutility of adjuvant radiation vs. none** | |
| Within study:  N=1 study;  Bromley 2019  N=172 | **Pooled disutility, within study (95% CI):** 0.03 [-0.03, 0.09]  *study is high ROB |
|  | **Subgroup, by surgery, disutilities (95% CI):**  Adjuvant radiation with BCS/partial mastectomy:  vs. No adjuvant radiation with BCS/partial mastectomy: 0.03 [-0.03, 0.09] *study is high ROB |
| Between study: Adjuvant radiation, N=3 studies;  Bromley 2019, Knuttel 2017, Schleinitz 2006  N=449 | **Pooled adjuvant radiation (95% CI):** 0.70 [0.69, 0.71]  *2/3 studies high ROB  **Pooled no adjuvant radiation (95% CI):** 0.78 [0.77, 0.79]  *study is high ROB  **Disutility, between study (95% CI):** 0.08 [0.07, 0.09]  *2/3 studies high ROB |
|  | **Subgroup, by surgery, utilities (95% CI):**  **Adjuvant radiation with BCS/partial mastectomy: 0.78 [0.77, 0.79] *both studies high ROB vs. No adjuvant radiation with BCS/partial mastectomy: 0.78 [0.77, 0.79] *study is high ROB**  Adjuvant radiation with mixed/unspecified surgery: 0.61 [0.60, 0.62] *moderate ROB vs. No adjuvant radiation with mixed/unspecified surgery: no evidence |
|  | **Subgroup, by age, utilities (95% CI):**  Adjuvant radiation, age <55 years (95% CI): 0.63 [0.61, 0.65] *moderate ROB  Adjuvant radiation, age ≥55 years (95% CI): 0.58 [0.56, 0.60] *moderate ROB |
|  | **Subgroup, by race, utilities (95% CI):**  Adjuvant radiation, White race: 0.68 [0.66, 0.70] *moderate ROB  Adjuvant radiation, Black race: 0.54 [0.51, 0.57] *moderate ROB  Adjuvant radiation, other race: 0.54 [0.47, 0.61] *moderate ROB |
|  | **Subgroup, by family history of breast cancer, utilities (95% CI):**  Adjuvant radiation, family history of breast cancer: 0.58 [0.56, 0.60] *moderate ROB  Adjuvant radiation, no family history of breast cancer: 0.63 [0.61, 0.65] *moderate ROB |
| **Disutility of ALND vs. SLND** | |
| Within study: No evidence | **Disutility, within study:** no evidence |
| Between study:  N=1 study;  Knuttel 2017  N=121 participants | **Pooled ALND utilities (95% CI):** no evidence  **Pooled SLNB utilities (95% CI):** 0.89 [0.87, 0.91]  *study is high ROB  **Disutility, between study (95% CI):** no evidence |
|  | **Subgroup, by surgery, utilities, between study (95% CI) only data for those receiving SLNB:**  SLNB with BCS/partial mastectomy and radiation: 0.90 [0.87, 0.93]  SLNB with mastectomy and mixed/unspecified adjuvant therapy: 0.88 [0.85, 0.91] |
| **Disutility of advanced stage vs. not advanced stage (Stage II-IV vs. 0/I)** | |
| Within study:  N=1 study;  Knuttel 2017  N=121 participants | **Disutility, within study (95% CI):** 0.02 [-0.07, 0.11]  *study is high ROB |
|  | **Subgroup, by surgery and adjuvant therapy, disutilities within study (95% CI):**  Stage 0/I vs. Stage II/III/IV with BCS/partial mastectomy and radiation: 0.05 [-0.04, 0.14]  *study is high ROB  Stage 0/I vs. Stage II/III/IV with mastectomy and mixed/unspecified adjuvant therapy: -0.02 [-0.13, 0.09] *study is high ROB |

ALND, axillary lymph node dissection; BCS, breast-conserving surgery; CI, confidence interval; ROB; risk of bias; SLNB, sentinel lymph node biopsy; SLND, sentinel lymph node dissection; vs., versus

**Table S2.6. Study Characteristics for Studies Reporting on Health State Utility Values**

| **Author, Year, Country, Data collection time**  **Funding source(s)** | **Participants**  **Recruitment pool**  **Enrolled N (analyzed N)** | **Elicitation tool(s) (country, use of vignettes/patients); range** | **Health state(s) evaluated^ⱡ^; Timing of data collected** | **Mean age (yrs)**  **Gender (other than female, %)** | **Comorbidities**  **Race/ethnicity**  **Stage/severity of cancer** |
| --- | --- | --- | --- | --- | --- |
| **Screening-specific health states** | | | | | |
| Domeyer, 2010,  Greece  NR  NR | Adult women (aged ≥18 yrs) with non-palpable mammographic lesions requiring vaacuum-assisted breast biopsy recruited from Breast Unit, First Department of Propaedeutic Surgery, Hippokratio Hospital, Medical School, University of Athens and followed up 18 mos post-biopsy, diagnosed with benign lesions.  102 (102 analyzed at pre-biopsy and immediate post-biopsy [dx results not known]; 78 analyzed at 18 mos follow-up [dx results known]) | EQ-5D-3L (Spain; patients)  Range NR | Screening positive (before dx work up); 1-2 hours before biopsy; current state  After biopsy (dx results not known); 4 days after biopsy; current state  False positive (after dx results known) from biopsy; 18 months after biopsy; current state | 51.3  NR (not reported) | NR  NR  NA |
| Tosteson, 2014  USA  2001-NR  Non-industry grant | Women (age range NR) with positive screening mammogram, defined as any mammogram where additional work-up or consultation was recommended; and a sample of women with a negative screening mammogram, subsampled from DMIST study and followed up approximately after 12 mos of mammography.  1226 (1025 analyzed after mammogram; 1019 analyzed 12 mos after mammogram) | EQ‐5D-3L (USA; patients); 0.11 to 1 | A negative screening mammogram, Baseline (after mammography, results known); & Approximately 12 mos after mammography; current state  Screening positive (before dx work up); & Approximately after 12 mos after mammography (after dx workup); current state  Screening positive (after dx workup - resolved results known); & Approximately 12 mos after mammography (after dx workup - resolved results known); current state  *A positive screening mammogram that is any mammogram where additional work-up or consultation was recommended. | NR  NR | NR  White 82%  Black 12%  Hispanic/Latina 4%  Other 2%  NA |
| Timmers, 2014  Netherlands  2010-2012  Non-industry grant | Women (aged 49-75 yrs) with a Breast Imaging Reporting and Data System (BI-RADS) 0 during breast cancer screening in the MASS trial, recalled to the GP and hospital. 4 A BI-RADS code 0 indicates that the screening radiologist needs more information to determine whether recall is necessary, for instance, in the case of possible superimposed images.  369 (204 analyzed in effectiveness analysis) | EQ‐5D-3L (Netherlands; patients)  Range NR | Screening positive (before dx workup, Intervention and Control); directly after receiving screening results before any further follow-up; current state  False positive (Not all resolved, but results combined with false positive on diagnostic mammogram and those who need a biopsy now); current state  Intervention: quick, non-invasive assessment (additional mammographic views and/or ultrasound examination)  Control: usual care of recall to the GP and assessment in hospital by the multidisciplinary breast cancer team | 57  NR | NR  NR  NA |
| **Non-screening specific health states** | | | | | |
| Ali, 2017  USA  2006-2013  Non-industry grant | Elderly women (age range NR) diagnosed with HR + early stage breast cancer recruited from 6 cohorts from the Surveillance, Epidemiology, and End Results (SEER) – Medicare Health Outcomes Survey (MHOS).  618 (592 analyzed after propensity score matching; 75 analyzed in our analysis of interest) | VR-6D (Country NR; patients); 0 to 1 | BCS; T1: 7-12 months from diagnosis; current state  BCS with radiation; T1: 7-12 months from diagnosis; current state  *BCS without radiation pooled for BCS  overall, but n<30 for separate analysis | 73.7  NR | 0 comorbidities: 10.03%  1-2 comorbidities: 40.94%  2+ comorbidities: 49.03%  White: 79.94%  Black: 9.55%  Others: 10.52%  Stage I and II and T1-T3/N0 or M0: 100% |
| Bromley, 2019  Australia  2018  Non-industry grant | Patients (aged 30+ yrs) with DCIS/early invasive breast cancer and women without breast cancer (aged 30+ yrs) were recruited through the Lifepool cohort and Breast Cancer Network Australia’s Review & Survey group.  254 (94 patients, 78 public analyzed) | EQ-5D-5L (UK; patients+public); 0 to 1  Standard Gamble (vignettes); 0 to 1 | BCS without radiation; T1: hypothetical during/after treatment  BCS with radiation; T1: hypothetical during/after treatment  Mastectomy and optional reconstruction; T1: hypothetical during/after treatment | 62.9  NR | NR  Australian  Patients:73%,  non-patients: 78%;  New Zealand/Torres Strait Islander  Patients: 4%  non-patients: 5%;  European  Patients: 12%  non-patients: 8%;  Asian  Patients: 3%  non-patients: 6%;  American  Patients: 8 %  non-patients: 0%;  Middle Eastern  Patients: 0%  non-patients: 3%  Patients:  Stage 0 (DCIS): 35%  Early invasive breast cancer 65%  Metastatic: 0% |
| Criscitiello, 2021  UK, USA, Japan, France, Germany, Italy, and Spain  2019-2019  Industry | Patients (aged ≥18 yrs) with a physician-confirmed diagnosis of stage I to III HR + /HER2–breast cancer.  1110 (1077 analyzed) | EQ-5D-5L (US, Japan, France, Germany, and Spain; patients). UK and Italy index scores were calculated by using the crosswalk values sets that map EQ-5D-5L to the EQ-5D-3L index scores.  Range NR | Mixed/unspecified surgery with mixed adjuvant treatment in advanced stage (stage III) vs not (stage I-II); T1: within 12 mos of treatment or currently on adjuvant treatment; current state | 59.2  Female sex: 99.0% | NR  NR  Stage I-III: 99.5%  Unknown: 0.5% |
| De Kok, 2010  Netherlands  2005-2007  Non-industry grant | Patients (aged 26-89 yrs), 1 day before receiving curative breast cancer surgery in four Dutch hospitals.  324 (262 analyzed) | EQ-5D-5L (Netherlands; patients)  Range NR | At diagnosis, before treatment; current state  *1 day before surgery | 55.7  NR | NR  NR  Curative BC: 100% |
| Fujii, 2019  Japan  2015-2018  Non-industry grant | Patients (aged 20+ yrs) with American Society of Anesthesiologists physical status 1 or 2 scheduled for breast cancer resection by mastectomy at the Nagoya University Hospital, Nagoya, Japan, studied between August 2015 and August 2018, randomized to serratus block or pectoral nerve-2 block during surgery). They were followed up at 6 mos post-surgery.  80 | EQ‐5D‐3L (Japan; patients)  Range NR | Mastectomy without radiation; T1: 6 mos postoperative | 58.2  NR | NR  NR  NR |
| Gordon, 2017  Australia  2006-2008 (recruitment)  Non-industry grant | Participants (aged 20 to 69 yrs) who were newly diagnosed with breast cancer and were recruited from 4 participating hospitals in Brisbane (Australia) and resided within 30 km (18.6 miles) of Brisbane central business district. Patients were randomized to receive either an exercise intervention delivered through either face‐to‐face home delivery or by telephone, or usual care in the control arm over an 8‐month period starting 6 weeks after breast surgery.  200 (198 analyzed) | EQ-5D-3L (Australia; patients); 0-1 | Mixed/unspecified surgery without radiation; T1: 6 weeks post-surgery; current state | 52.0  NR | NR  NR  Stage:  0: 4.1%  I: 30.4%  II/III: 61.9%  Unknown: 3.6% |
| Haidari, 2022  France  2006-2009  Non-industry grant | Patients (age range NR) hospitalized for the diagnosis or treatment of early BC or for a suspicion of BC in East centers of France, the cancer centers of Dijon, Nancy and in the university hospitals of Strasbourg and Reims and followed up until six months post hospitalization.  381 (304 analyzed) | EQ‐5D‐3L (France; patients)  Range NR | At diagnosis, before treatment; current state | 57.7  NR | 1+ comorbidity: 35%  NR  Stage I: 38.3%  Stage II: 23.9%  Stage III: 2.1%  Stage IV: 0.8%  Missing: 23.4% |
| Hall, 2015  UK  2010-2011 (recruitment)  Non-industry grant | Adult patients (aged 23-92 yrs) who were within 6 months of diagnosis of early breast cancer with curative intent, recruited from Leeds Teaching Hospitals NHS Trust (LTHT) and Calderdale & Huddersfield NHS Foundation Trust (CHFT).  291 (231 analyzed - by adjuvant therapy group: NR) | EQ-5D-3L (UK; patients); 0 to 1 | Mixed/unspecified surgery with chemotherapy; T1: 6 months post-diagnosis; current state  Mixed/unspecified surgery without chemotherapy; T1: 6 months post-diagnosis; current state  Mixed/unspecified surgery with radiation; T1: 6 months post-diagnosis; current state  Mixed/unspecified surgery without radiation; T1: 6 months post-diagnosis; current state | 56  Male: 0.4% | NR  NR  Early BC: 100%  Primary tumour  T1: 44.8%  T2: 20.4%  T3: 2.4%  T4: 0%  Unknown: 32.4%  Lymph node status (N stage):  N0: 50.0%  N positive: 18.8%  Unknown: 31.2% |
| Hanson, 2022  USA  2017 to 2018  Non-industry grant | Participants (aged 23-85 yrs) were from Texas Cancer Registry (TCR) with stage 0-II female breast cancer diagnosed between 2006 and 2008 who were alive in 2016 and had undergone wither breast conserving surgery or mastectomy with reconstruction.  647 (510 analyzed) | EQ-5D-3L (US; patients); -0.109 to 1 | BCS with radiation; T2: Median: 10.3 (range 8.4-12.5) yrs after diagnosis; current state  Mastectomy; T2: Median: 10.3 (range 8.4-12.5) yrs after diagnosis; current state | Median 53 (Range 23-85)  NR | NR  American Indian/Alaska Native 1.1%  Asian American/Pacific Islander 14.2%  Black 22.3%  Hispanic 23.2%  White 39.3%  Stage 0-II: 100% (inclusion criteria)  Nodal status  Uninvolved (N0): 70.8%  Involved (N+): 14.2%  Unnknown: 15.0% |
| Kim, 2015  South Korea  2012  Industry grant | Breast cancer patients (age range NR) who had surgery as a primary treatment at the ambulatory cancer center of 1 tertiary hospital ins Seoul, Korea.  1,002 (827 analyzed; 147 analyzed in analysis of interest) | EQ-5D-3L (South Korea; patients); -0.171 to 1 | Mixed/unspecified surgery with chemotherapy; T1: <12 mos from diagnosis; current state | NR  NR | NR  NR  Stage 0-II: 91.8%  Stage III: 8.2% |
| Kouwenberg, 2020  Netherlands  NR  None | Patients (age range NR) surgically treated for nonmetastatic breast cancer with breast-conserving surgery, mastectomy, autologous breast reconstruction, and implant-based breast reconstruction in the past 10 years (2008 to 2018). Patients were recruited from four hospitals in the Netherlands (one academic hospital and three general hospitals), and were invited by mail to participate in a self-administered cross-sectional online survey.  1871 (1871 analyzed; 1294.4 analyzed after propensity score matching) | EQ‐5D‐5L (Netherlands; patients), 0 to 1 | Breast conserving surgery with radiation; T2: 7.04 ± 4.56 yrs from surgery; current state  Mastectomy; T2: 6.86 ± 4.34 yrs from surgery; current state  Mastectomy (and autologous breast reconstruction); T2: 6.61 ± 4.65 yrs; current state  Mastectomy (and implant-based breast reconstruction); T2: 7.04 ± 4.95 yrs; current state  *timing is after propensity weighted adjustment | 61.4  *at time of survey  NR | NR  1+ comorbidity: 52.1%  Metastatic BC: 0% |
| Lagendijk, 2018 (a)  Netherlands  2005-2016 (recruitment)  NR | Patients (≥ 18 years of age) treated with either breast-conserving therapy (BCT), mastectomy alone (MAS), mastectomy followed by (in)direct implant reconstruction (REC I), or a mastectomy followed by (in)direct autologous reconstruction (REC A) between January 2005 and September 2016 were identified from electronic patient files using operation codes from Erasmus Medical Centre (Erasmus MC), Rotterdam, The Netherlands.  612 (586 analyzed) | EQ-5D-5L (Country NR, patients); 0.28 to 1.0 | Breast conserving surgery; T2: 5.3 (2.8–8.1) yrs from surgery; current state  Mastectomy; T2: 7.1 (3.7–9.8) yrs from surgery; current state  Mastectomy (and (in)direct implant reconstruction) without radiation; T2: 7.0 (3.7–10.4) yrs from surgery; current state  Mastectomy (and (in)direct autologous reconstruction); T2: 7.2 (4.7–9.5) yrs from surgery; current state | Median: 51.0 Range: 43.0–60.0  NR | NR  NR  T Stage:  T1:57.8%  T2: 20.9%  T3: 2.9%  CIS: 18%  N Stage:  N0: 72.2%  N1+: 27.8%  Unknown: 0% |
| Lagendijk, 2018 (b)  Netherlands  2007-2012 (recruitment)  NR | Patients (aged 50-62 yrs) treated through breast conserving therapy between 2007 and 2012 at a tertiary referral center, initially following a preoperative breast MRI.  68 (58 analyzed) | EQ-5D-5L (Country NR; patients)  Range NR | Breast conserving surgery and whole breast irradiation- (and radiation "boost"); T2: 44.0 (31-59) mos from surgery; current state | Median 54.7, Range 50-62  NR | NR  NR  T-stage:  T1: 73.5%  T2: 25.0%  T3: 1.5%  DCIS: NR  Metastatic: 0%  N-stage:  N0: 70.6%  N1: 23.5%  N2: 4.4%  Unknown: 1.5% |
| May, 2017  Netherlands  2010-2013  Non-industry grant | Patients (aged 25-75 yrs) with histological diagnosis of breast cancer <6 wks before recruitment scheduled for chemotherapy from seven outpatient clinics enrolled in an RCT on 18-week exercise program vs. usual care  204 (165 analyzed) | EQ-5D-3L (Dutch; patients)  Range NR | Mixed/unspecified surgery with chemotherapy; T1: Approximately 18 wks from diagnosis (<6 wks from diagnosis at baseline + 12 wks follow-up; exercise group); Approximately 18 wks from diagnosis (<6 wks from diagnosis at baseline + 24 wks follow-up; control group); current state | 49.7  NR | NR  NR  Stage M0: 100% |
| Min, 2014  South Korea  2013  Non-industry grant | Patients (aged 36 to 65 yrs) who were planning to receive neoadjuvant chemotherapy for breast cancer were recruited for participation in this feasibility study when they were admitted to the Breast Cancer Center, Asian Medical Center, Seoul, South Korea, for 2 nights to evaluate the disease status for preoperative chemotherapy.  38 (30 analyzed) | EQ-5D-3L (Country NR; patients)  Range NR | At diagnosis before treatment; current state  *within 4 weeks of diagnosis, before neoadjuvant chemotherapy | 45  NR | NR  NR  Lymph node metastasis: 77%  Distant metastasis: 0% |
| Miret, 2023  Spain  2013-2015 (recruitment)  Non-industry grant | Women (aged >18 yrs) diagnosed with incident breast cancer in one of the participating Spanish hospitals.  1456 (1276 analyzed) | EQ-5D-5L (Spain; patients); 0-1 | At diagnosis before treatment; current state | 58  NR | Charlson comorbidity index:  0: 79.7%  1: 12.9%  2: 5.3%  3: 1.6%  4+: 0.4%  NR  Stage 0: 10.3%  Stage I: 52.2%  Stage II: 30.3%  Stage III: 7.2% |
| Morgan, 2021  England and Wales  2013-2018  Non-industry grant | Patients (aged ≥70 yrs) recruited at the time of diagnosis and before commencement of treatment from 56 breast cancer units, with ER-positive breast cancer.  2354 (At diagnosis: 2029 analyzed; T1: 1477 analyzed) | EQ-5D-5L (Country NR; patients)  Range NR | At diagnosis before treatment; current state  Mixed/unspecified surgery without chemotherapy; T1: at 12 mos after diagnosis; current state | 76.4  NR | NR  NR  Invasive, operable BC: 100%  DCIS: 0% |
| Moro-Valdezate, 2014  Spain  2003-2007  NR | Early breast cancer patients (age range NR) that underwent oncological surgery in three institutions from Valencia, Spain.  446 (364 analyzed) | EQ-5D-5L (Spain; patients); -1 to +1 | BCS with chemotherapy, radiation and ALND; T1: at 1 year after surgery; current state  Mastectomy with chemotherapy and ALND; T1: at 1 year after surgery; current state  Mixed/unspecified surgery with chemotherapy and ALND; T1: at 1 year after surgery; current state | 59  NR | NR  White: 99.2%  Hispanic/Latina: 0.8%  Stage I: 47.5%  Stage IIa: 27.2%  Stage IIb: 11.8%  Stage IIIa: 8.8%  Stage IIIb: 4.7%  DCIS: 0% |
| Moshina, 2022  Norway  2019 to 2020  Non-industry grant | Women (aged 50-69 yrs) at invitation to screening or at histologically verified invasive breast cancer diagnosis (symptomatic, screen-detected or interval cancer) recruited from BreastScreen Norway database.  3232 | EQ-5D-5L (Danish; patients). Converted from EQ-5D-3L by cross-walking algorithm.  Range NR | Mixed/unspecified surgery with Radiation (Y); T2: Time since diagnosis: Symptomatic: 8.0 (3.4) yrs; Screen-detected: 7.6 (3.4) yrs; Interval: 7.8 (3.5) yrs; current state | 66.7  NR | NR  NR  Stage I: 45.3%  Stage II: 34.7%  Stage III: 14.7%  Stage IV: 1.4%  Missing: 3.9%  DCIS: 0% |
| Park, 2023  South Korea  2020-2021 (recruitment)  Non-industry grant | Patients from two hospitals in South Korea (aged 20-70 yrs) who underwent axillary lymph node dissection or breast reconstruction following mastectomy, with limited range of motion ≤8 wks after the operation  100 (93 analyzed) | EQ-5D-5L (Country NR; patients)  Range NR | Mastectomy; T1: ≤8 wks from surgery; current state | 44.9  NR | NR  NR  NR |
| Porciello, 2020  Italy  2016-2019 (recruitment)  Non-industry grant | Patients with breast cancer (aged ≥30 and ≤75 yrs) recruited and followed up in national cancer institutes or oncologic departments of hospitals located in Southern and Northern Italy. Patients were part of a randomized trial of dietary modification, physical activity and vitamin D supplementation.  309 (308 analyzed) | EQ-5D-3L (Italy; patients)  Range NR | Mixed/unspecified surgery, advanced stage (stage III) vs not (stage I-II; T1: <12 mos since diagnosis; current state | 52  NR | 0 comorbidities: 60.5%  1 comorbidity: 27.2%  2+ comorbidities: 12.3%  NR  Stage I: 30.1%  Stage IIA: 42.7%  Stage IIB: 12.9%  Stage IIIA: 11.7%  Stage IIIC: 2.6% |
| Rautalin, 2018  Finland  2009-2011  Industry and Non-industry grant | Patients (aged 29-90 yrs) with histologically verified breast cancer were identified from hospital records in the Helsinki and Uusimaa Hospital District.  268 (267 analyzed for EQ-5D; 265 analyzed for 15D) | EQ-5D-3L (UK; patients); -0.594 to 1.000  15D (Country NR; patients); 0 to 1 | Mixed/unspecified surgery, without chemotherapy or radiation; T1: during treatment within 6 months from diagnosis (mean 0.7 mos); current state, and  Mixed/unspecified surgery, without chemotherapy or radiation; T1: in remission 6 to 18 months from diagnosis (mean 13 mos); current state | 59.1  Male: 1.1% | NR  NR  Non-metastatic: 100% |
| Rautalin, 2021  Finland  2008-2015 (recruitment)  Non-industry grant | Patients (aged 24-89 yrs) diagnosed with primary breast cancer at Helsinki and Uusimaa Hospital District (HUS) Finland between September 2008 and September 2015 and then underwent mastectomy, breast resection, oncoplastic resection or immediate breast reconstruction.  1065 (1065 analyzed at baseline; 1010 analyzed at T1; 964 analyzed at T2) | 15D (Country NR, patients); 0 to 5 | At diagnosis, before treatment; current state  Mastectomy; T1: 3 mos after primary surgery; current status  BCS (Breast resection) with radiation; T1: 3 mos after primary surgery; current status  BCS (Oncoplastic resection) with radiation; T1: 3 mos after primary surgery; current status  Mastectomy (and immediate breast reconstruction); T1: 3 mos after primary surgery; current status  Mastectomy; T2: 24 mos after primary surgery; current status  BCS (Breast resection) with radiation; T2: 24 mos after primary surgery; current status  BCS (Oncoplastic resection) with radiation; T2: 24 mos after primary surgery; current status  Mastectomy (and immediate breast reconstruction); T2: 24 mos after primary surgery; current status | 59.7  NR | Charlson comorbidity points:  1 point: 11.1%  2 points: 8.0%  3 points: 1.3%  NR  T1: 65.8%  T2: 29.1%  T3: 3.2%  T4: 0.8%  N0: 61.3%  N1: 27.6%  N2: 8.4%  N3: 2.6%  DCIS: 3.8% |
| Ring, 2021  UK  2013-2018 (recruitment)  Non-industry grant | Patients (aged ≥70 yrs) at diagnosis of primary operable invasive breast cancer from 56 UK centres in England and Wales, followed up for 24 months. Utility analysis was restricted to early stage high-risk breast cancer patients who underwent surgery within 6 months from diagnosis.  1520 (1315 analyzed at diagnosis; 780 analyzed at follow-up) | EQ-5D-5L (Country NR; patients)  Range NR | At diagnosis before treatment; current state  Mixed/unspecified surgery, without Chemotherapy; T1: 18 months from diagnosis; current state  Mixed/unspecified surgery, with chemotherapy; T1: 18 months from diagnosis; current state | NR  NR | NR  NR  Operable, invasive BC: 100%  DCIS: 0%  Tumour size  <=20mm: 45.5%  21-50mm: 45.7%  >50mm: 6.7%  Unknown: 2.1%  Nodal status  pN0-1 mi: 68.1%  pN1: 21.8%  pN2: 5.2%  pN3: 2.8%  pNx: 2.2% |
| Roine, 2020  Finland  2005-2007 (recruitment)  Non-industry grant  (Roine 2021 – associated for age subrgroups) | Women (aged 35-68 yrs) newly diagnosed with invasive breast cancer, recently completed the adjuvant chemotherapy or started endocrine therapy and/or radiotherapy were enrolled into the BREX study between September 2005 and September 2007, who completed the 15D at five-year follow-up.  NR (182 analyzed at T1, 148 analyzed at T2) | 15D (Country NR, patients); 0 to 1 | Mixed/unspecified surgery with adjuvant chemotherapy; T1: baseline, shortly after completion of adjuvant therapy; current status  Mixed/unspecified surgery with adjuvant chemotherapy; T2: 30 months after completion of adjuvant therapy; current status | 52.6  NR | NR  NR  T1-4N0-3: 100% |
| Swanick, 2018  USA  2015-2016 (recruitment)  Non-industry grant | Women (aged ≥67 yrs) in whom nonmetastatic breast cancer was diagnosed in 2009 and were treated with 1 of the 5 local therapy strategies of interest, who were alive as of May 2015, and who had continuous fee-for-service Medicare Part A and B coverage between diagnosis and the end of 2011.  489 (466 analyzed) | EQ-5D-3L (Country NR; patients)  Range NR | BCS with radiation (whole breast irradiation or brachytherapy); T2: approximately 6 years from diagnosis  BCS without radiation or chemotherapy; T2: approximately 6 years from diagnosis  Mastectomy without radiation; T2: approximately 6 years from diagnosis  Mastectomy with radiation and ALND; T2: approximately 6 years from diagnosis | Median 72 (range 67-87)  NR | Charlson comorbidity index:  0: 67.6%  1+: 32.4%  White: 86.4%  Non-white: 13.6%  Nonmetastatic BC: 100% |
| Tanaka, 2019  Japan  2013-2015  NR | Patients (age range NR) with breast cancer who received their first course of intravenous outpatient chemotherapy at Gifu Municipal Hospital between December 2013 and November 2015.  38 | EQ-5D (Country NR; patients)  Range NR | Mixed/unspecified surgery with chemotherapy without radiation; T1: <6 months from start of adjuvant chemotherapy (before 2^nd^ course of chemotherapy); current state | 55.1  NR | NR  NR  Stage:  I: 23.7%  II: 50.0%  III: 18.4%  IV: 7.9%  DCIS: 0% |
| Tran, 2022  South Korea  2004-2020  Non-industry grant | Patients (aged ≥18 yrs) diagnosed with histologically confirmed breast cancer, having no other cancer, recruited from the National Cancer Center in Korea.  298 patients (295 analyzed at diagnosis and T1; 124 analyzed at T2); 7992 age- and education level-matched public | EQ-5D-3L (Korea; patients+public); -0.17 to 1 | At diagnosis before treatment (2-3 days after); current state  Healthy population control, age- and education level-matched; current state  Mastectomy with radiation; T1: 3 mos from diagnosis; current state  Mastectomy with radiation; T2: Median 15 years from diagnosis; current state | 46.3  NR | 34%  NR  Stage 0-II: 83%  Stage III-IV: 18% |
| Velikova, 2018  UK  2006-2013 (recruitment)  Non-industry grant | Women (aged ≥18 yrs) who had undergone mastectomy for unilateral breast cancer and, if they had intermediate risk breast cancer, an axillary staging procedure with axillary lymph node dissection. Patients were recruited from the 111 UK centers eligible for the SUPREMO trial.  989 (947 analyzed at T1 before adjuvant therapy; 776 analyzed at T1 for radiation vs no radiation; 717 analyzed at T2) | EQ-5D-3L (UK; patients); 0 to 1 | Mastectomy without chemotherapy or radiation; T1: directly after surgery before adjuvant therapy; current state  Mastectomy with radiation; T1: 12 months post-surgery; current state  Mastectomy without radiation; T1: 12 months post-surgery; current state  Mastectomy with radiation; T2: 24 months post-surgery; current state  Mastectomy without radiation; T2: 24 months post-surgery; current state | 56.1  NR | NR  NR  Tumour grade  1: 4.5%  2: 40.7%  3: 54.1%  Not specified: 0.7%  DCIS: 0% |
| Youens, 2019  Australia  2012-2015  Non-industry grant | Participants (age range NR) diagnosed with early breast cancer referred for curative radiotherapy in three sites, in Perth, Melbourne, and Adelaide.  408 | AQoL‐6D (Australia; patients)  Range NR | BCS without radiation; T1: prior to commencement of adjuvant radiation; current state  BCS with radiation; T1: within 1 week of adjuvant therapy completion; current state | 56.8  NR | Other health conditions: 52.9%  Non-immigrant: 90.2%  Early BC: 100% |
| Zigman, 2020  Croatia  2016  NR | Patients (irrespective of their age) who presented for genetic counseling at Sestre milosrdnice University Hospital Centre.  114 (81 patients; 33 public) | EQ-5D-3L (UK; patients and public); -0.594 to 1.000 | At diagnosis before treatment; current state  Healthy high-risk for breast cancer control; current state | 52.3  NR | NR  NR  Patients:  Localized stage BC: 60.5%  Advanced stage BC: 39.5% |
| **Not included for primary analysis** | | | | | |
| Bonomi, 2008  USA  NR  Non-industry grant | Subjects comprised English-speaking women aged 50–79 years randomly sampled from Group Health’s Breast Cancer Screening Program (BCSP).  137 (131 analyzed) | VAS (vignettes; public); 0 to 100 | Screening test process (after screening but before screening result); hypothetical  Negative screening (know result); hypothetical  Interval cancer (scan deemed normal but after a few months noticing a lump that results in biopsy and breast cancer diagnosis); hypothetical  Screening positive (before diagnostic workup - describes both an additional mammogram and possible biopsy); hypothetical  New diagnosis, before treatment; hypothetical  False positive (resolved after dx results - scenario describes additional mammography, ultrasound and biopsy); hypothetical | NR  NR | NR  White: 65.4%  Black: 16.7%  Asian: 16.0%  Native American: 0.7%  Other race: 2.2%  NA (previous BC: 9.9%) |
| Knuttel, 2017  Netherlands  NR  None | Patients (age range NR) who completed breast cancer treatment more than 12 months ago and also the public (healthy women) older than 40 years without a history of breast cancer. Healthy volunteers were friends or relatives of the participating patients with breast cancer.  71 (patients analyzed) 50 (public analyzed) | TTO (vignettes; patients+public)  Range NR | **Included for SLNB health states:**  Mastectomy with SLNB; T1: hypothetical at dx  Mastectomy followed by direct implant-based reconstruction and SLNB; T1: hypothetical at dx  Lumpectomy with SLNB and whole breast radiotherapy; T1: hypothetical at dx | 58.4  NR | NR  NR  Patients:  TNM stage:  0: 22.5%  I: 42.3%  II: 28.2%  III: 5.6%  IV: 1.4% |
| Schleinitz, 2006  USA  2003-2004 (recruitment)  Non-industry grant | Women (aged over 25 yrs) recruited from primary care clinics and the community.  156 | SG (vignettes; public)  TTO (vignettes; public)  Ranges NR | **Included for subgroup analyses (age, race and family hx of BC)**  SG: At diagnosis, before treatment; hypothetical  TTO:  Mixed/unspecified surgery and chemotherapy; T1: hypothetical  Mixed/unspecified surgery and radiation; T1: hypothetical | NR  NR | NR  White: 48.1%  Black: 41.0%  Asian: 1.9%  American Indian: 9.0%  Hispanic: 8.3%  Stage I-IV (hypothetical health states)  DCIS: 0% |
| Songtish, 2014  Thailand  NR  Non-industry grant | Healthy Thai women (aged 26 to 60 yrs; additional details NR)  110 | SG (vignettes; public)  Range NR | BCS; T1: hypothetical after surgery | NR  NR | NR  NR  NR |

ALND, axillary lymph node dissection; BC, breast cancer; BCS, breast-conserving surgery; DCIS, ductal carcinoma in situ; Dx, diagnosis; EQ-5D-3L, EuroQol 5 Dimensions 3 Levels; EQ-5D-5L, EQ-5D-3L, EuroQol 5 Dimensions 5 Levels; HER2, Human Epidermal Growth Factor Receptor 2; HR, hormone receptor; Mos, months; MRI, magnetic resonance imaging; NA, not applicable; NR, not reported; SG, standard gamble; SLNB, sentinel lymph node biopsy; TTO, time trade-off; VAS, visual analog scale; VR-6D, veterans RAND 6-Item Health Survey; Wks, weeks; yrs, years

**Table S2.7. Summary of Risk of Bias*, HSUV studies**

| **Study (Utility tool, as applicable)** | **Was an appropriate study sample selected from the sampling frame?** | **Was the participation rate adequate?** | **Are responders and non-responders sufficiently similar?** | **Was attrition and/or missing data sufficiently low to minimize the risk of bias? (<20% T1 and <40% T2)** | **Was the instrument used for eliciting relative importance valid and reliable?** | **Was the instrument administered in the intended way?** | **Did authors present participants with a valid representation of the health state or intervention being evaluated?** | **Did the researchers check the understanding of the instrument?** | **Were the results analyzed appropriately to avoid bias and confounding? (reporting variance measures)** | **Overall bias** |
| --- | --- | --- | --- | --- | --- | --- | --- | --- | --- | --- |
| Bromley  2019 |  |  |  |  |  |  |  |  |  |  |
| Haidari, 2022 |  |  |  |  |  |  |  |  |  |  |
| Domeyer, 2010  Screen positive before dx workup |  |  |  |  |  |  |  |  |  |  |
| Domeyer, 2010  After biopsy (FP but dx results not known) |  |  |  |  |  |  |  |  |  |  |
| Domeyer, 2010  After biopsy (FP results known) |  |  |  |  |  |  |  |  |  |  |
| Tosteson, 2014 |  |  |  |  |  |  |  |  |  |  |
| Timmers, 2014 |  |  |  |  |  |  |  |  |  |  |
| Fujii, 2019 |  |  |  |  |  |  |  |  |  |  |
| Kouwenberg, 2020 |  |  |  |  |  |  |  |  |  |  |
| Lagendijk, 2018 (a) |  |  |  |  |  |  |  |  |  |  |
| Lagendijk, 2018 (b) |  |  |  |  |  |  |  |  |  |  |
| Knuttel, 2017 |  |  |  |  |  |  |  |  |  |  |
| Rautalin, 2021  At dx |  |  |  |  |  |  |  |  |  |  |
| Rautalin, 2021  T1 |  |  |  |  |  |  |  |  |  |  |
| Rautalin, 2021  T2 |  |  |  |  |  |  |  |  |  |  |
| Roine, 2020  T1 |  |  |  |  |  |  |  |  |  |  |
| Roine, 2020  T2 |  |  |  |  |  |  |  |  |  |  |
| Criscitiello 2021 |  |  |  |  |  |  |  |  |  |  |
| Hanson, 2022 |  |  |  |  |  |  |  |  |  |  |
| May, 2017  T1 |  |  |  |  |  |  |  |  |  |  |
| Miret, 2023 |  |  |  |  |  |  |  |  |  |  |
| Morgan, 2021  At dx |  |  |  |  |  |  |  |  |  |  |
| Morgan, 2021  T1 |  |  |  |  |  |  |  |  |  |  |
| Moshina, 2022 |  |  |  |  |  |  |  |  |  |  |
| Park, 2023 |  |  |  |  |  |  |  |  |  |  |
| Porciello, 2020 |  |  |  |  |  |  |  |  |  |  |
| Rautalin, 2018 |  |  |  |  |  |  |  |  |  |  |
| Ring, 2021  At dx |  |  |  |  |  |  |  |  |  |  |
| Ring, 2021  T1 |  |  |  |  |  |  |  |  |  |  |
| Swanick, 2018 |  |  |  |  |  |  |  |  |  |  |
| Tran, 2022  At dx |  |  |  |  |  |  |  |  |  |  |
| Tran, 2022  T1 |  |  |  |  |  |  |  |  |  |  |
| Tran, 2022  T2 |  |  |  |  |  |  |  |  |  |  |
| Min, 2014 |  |  |  |  |  |  |  |  |  |  |
| Moro-Valdezate, 2014 |  |  |  |  |  |  |  |  |  |  |
| Youens, 2019 |  |  |  |  |  |  |  |  |  |  |
| Ali, 2017 |  |  |  |  |  |  |  |  |  |  |
| Schleinitz, 2006 |  |  |  |  |  |  |  |  |  |  |
| Songtish, 2014 |  |  |  |  |  |  |  |  |  |  |
| Kim, 2015 |  |  |  |  |  |  |  |  |  |  |
| Tanaka, 2019 |  |  |  |  |  |  |  |  |  |  |
| Gordon, 2017 |  |  |  |  |  |  |  |  |  |  |
| De Kok, 2010 |  |  |  |  |  |  |  |  | ≥ |  |
| Velikova, 2018  T1 |  |  |  |  |  |  |  |  |  |  |
| Velikova, 2018  T2 |  |  |  |  |  |  |  |  |  |  |
| Zigman, 2020 |  |  |  |  |  |  |  |  |  |  |
| Hall, 2015 |  |  |  |  |  |  |  |  |  |  |
| Bonomi, 2008  VAS |  |  |  |  |  |  |  |  |  |  |

At dx, At diagnosis; FP, false positive; T1, ≤12 months from surgery; T2, ≥2 years from surgery; VAS, visual analog scale

Green: low risk of bias; Yellow: moderate risk of bias; Red: high risk of bias; Grey: No information

*Our assessments were based on items stated in GRADE guidance 19 for assessing risk of bias of preference-based studies (Zhang Y et al. J Clin Epidemiol 2019, 111:94-104). See the manuscript text for more information including our modifications.

**References**

1. Ali AA, Xiao H, Tawk R, Campbell E, Semykina A, Montero AJ, Diaby V**.** Comparison of health utility weights among elderly patients receiving breast-conserving surgery plus hormonal therapy with or without radiotherapy. Curr Med Res Opin. 2017;33(2):391-400.

2. Bonomi AE, Boudreau DM, Fishman PA, Ludman E, Mohelnitzky A, Cannon EA, Seger D**.** Quality of life valuations of mammography screening. Qual Life Res. 2008;17(5):801-14.

3. Bromley HL, Mann GB, Petrie D, Nickson C, Rea D, Roberts TE**.** Valuing preferences for treating screen detected ductal carcinoma in situ. European journal of cancer (Oxford, England: 1990). 2019;123:130-7.

4. Criscitiello C, Spurden D, Piercy J, Rider A, Williams R, Mitra D, et al. Health-Related Quality of Life Among Patients With HR+/HER2- Early Breast Cancer. Clinical therapeutics. 2021;43(7):1228-44.e4.

5. de Kok M, Dirksen CD, Kessels AG, van der Weijden T, van de Velde CJ, Roukema JA, et al. Cost-effectiveness of a short stay admission programme for breast cancer surgery. Acta Oncol. 2010;49(3):338-46.

6. Domeyer PJ, Sergentanis TN, Zagouri F, Zografos GC**.** Health-related quality of life in vacuum-assisted breast biopsy: short-term effects, long-term effects and predictors. Health Qual Life Outcomes. 2010;8:11.

7. Fujii T, Shibata Y, Akane A, Aoki W, Sekiguchi A, Takahashi K, et al. A randomised controlled trial of pectoral nerve-2 (PECS 2) block vs. serratus plane block for chronic pain after mastectomy. Anaesthesia. 2019;74(12):1558-62.

8. Gordon LG, DiSipio T, Battistutta D, Yates P, Bashford J, Pyke C, et al. Cost-effectiveness of a pragmatic exercise intervention for women with breast cancer: results from a randomized controlled trial. Psycho-oncology. 2017;26(5):649-55.

9. Haidari RE, Anota A, Dabakuyo-Yonli TS, Guillemin F, Conroy T, Velten M, et al. Utility values and its time to deterioration in breast cancer patients after diagnosis and during treatments. Quality of life research : an international journal of quality of life aspects of treatment, care and rehabilitation. 2022;31(10):3077-85.

10. Hall PS, Hamilton P, Hulme CT, Meads DM, Jones H, Newsham A, et al. Costs of cancer care for use in economic evaluation: a UK analysis of patient-level routine health system data. Br J Cancer. 2015;112(5):948-56.

11. Hanson SE, Lei X, Roubaud MS, DeSnyder SM, Caudle AS, Shaitelman SF, et al. Long-term Quality of Life in Patients With Breast Cancer After Breast Conservation vs Mastectomy and Reconstruction. JAMA surgery. 2022;157(6):e220631.

12. Kim SH, Jo MW, Lee JW, Lee HJ, Kim JK**.** Validity and reliability of EQ-5D-3L for breast cancer patients in Korea. Health Qual Life Outcomes. 2015;13:203.

13. Knuttel FM, van den Bosch MAAJ, Young-Afat DA, Emaus MJ, van den Bongard DHJG, Witkamp AJ, Verkooijen HM**.** Patient Preferences for Minimally Invasive and Open Locoregional Treatment for Early-Stage Breast Cancer. Value in health : the journal of the International Society for Pharmacoeconomics and Outcomes Research. 2017;20(3):474-80.

14. Kouwenberg CAE, de Ligt KM, Kranenburg LW, Rakhorst H, de Leeuw D, Siesling S, et al. Long-Term Health-Related Quality of Life after Four Common Surgical Treatment Options for Breast Cancer and the Effect of Complications: A Retrospective Patient-Reported Survey among 1871 Patients. Plastic and reconstructive surgery. 2020;146(1):1-13.

15. Lagendijk M, van Egdom LSE, van Veen FEE, Vos EL, Mureau MAM, van Leeuwen N, et al. Patient-Reported Outcome Measures May Add Value in Breast Cancer Surgery. Annals of surgical oncology. 2018;25(12):3563-71.

16. Lagendijk M, Vos EL, Nieboer D, Verhoef C, Corten EML, Koppert LB**.** Evaluation of cosmetic outcome following breast-conserving therapy in trials: panel versus digitalized analysis and the role of PROMs. The breast journal. 2018;24(4):519-25.

17. May AM, Bosch MJC, Velthuis MJ, van der Wall E, Steins Bisschop CN, Los M, et al. Cost-effectiveness analysis of an 18-week exercise programme for patients with breast and colon cancer undergoing adjuvant chemotherapy: the randomised PACT study. BMJ open. 2017;7(3):e012187.

18. Min YH, Lee JW, Shin YW, Jo MW, Sohn G, Lee JH, et al. Daily collection of self-reporting sleep disturbance data via a smartphone app in breast cancer patients receiving chemotherapy: a feasibility study. J Med Internet Res. 2014;16(5):e135.

19. Miret C, Orive M, Sala M, Garcia-Gutierrez S, Sarasqueta C, Legarreta MJ, et al. Reference values of EORTC QLQ-C30, EORTC QLQ-BR23, and EQ-5D-5L for women with non-metastatic breast cancer at diagnosis and 2 years after. Quality of life research : an international journal of quality of life aspects of treatment, care and rehabilitation. 2023;32(4):989-1003.

20. Morgan JL, Shrestha A, Reed MWR, Herbert E, Bradburn M, Walters SJ, et al. Bridging the age gap in breast cancer: impact of omission of breast cancer surgery in older women with oestrogen receptor-positive early breast cancer on quality-of-life outcomes. The British journal of surgery. 2021;108(3):315-25.

21. Moro-Valdezate D, Buch-Villa E, Peiró S, Morales-Monsalve MD, Caballero-Gárate A, Martínez-Agulló Á, et al. Factors associated with health-related quality of life in a cohort of Spanish breast cancer patients. Breast Cancer. 2014;21(4):442-52.

22. Moshina N, Falk RS, Botteri E, Larsen M, Akslen LA, Cairns JA, Hofvind S**.** Quality of life among women with symptomatic, screen-detected, and interval breast cancer, and for women without breast cancer: a retrospective cross-sectional study from Norway. Quality of life research : an international journal of quality of life aspects of treatment, care and rehabilitation. 2022;31(4):1057-68.

23. Park H-Y, Nam KE, Lim J-Y, Yeo SM, Lee JI, Hwang JH**.** Real-Time Interactive Digital Health Care System for Postoperative Breast Cancer Patients: A Randomized Controlled Trial. Telemedicine journal and e-health : the official journal of the American Telemedicine Association. 2022.

24. Porciello G, Montagnese C, Crispo A, Grimaldi M, Libra M, Vitale S, et al. Mediterranean diet and quality of life in women treated for breast cancer: A baseline analysis of DEDiCa multicentre trial. PloS one. 2020;15(10):e0239803.

25. Rautalin M, Farkkila N, Sintonen H, Saarto T, Taari K, Jahkola T, Roine RP**.** Health-related quality of life in different states of breast cancer - comparing different instruments. Acta oncologica (Stockholm, Sweden). 2018;57(5):622-8.

26. Rautalin M, Jahkola T, Roine RP**.** Surgery and health-related quality of life - A prospective follow up study on breast cancer patients in Finland. European journal of surgical oncology : the journal of the European Society of Surgical Oncology and the British Association of Surgical Oncology. 2021;47(7):1581-7.

27. Ring A, Battisti NML, Reed MWR, Herbert E, Morgan JL, Bradburn M, et al. Bridging The Age Gap: observational cohort study of effects of chemotherapy and trastuzumab on recurrence, survival and quality of life in older women with early breast cancer. British journal of cancer. 2021;125(2):209-19.

28. Roine E, Sintonen H, Kellokumpu-Lehtinen P-L, Penttinen H, Utriainen M, Vehmanen L, et al. Long-term health-related quality of life of breast cancer survivors remains impaired compared to the age-matched general population especially in young women. Results from the prospective controlled BREX exercise study. Breast (Edinburgh, Scotland). 2021;59:110-6.

29. Roine E, Sintonen H, Kellokumpu-Lehtinen P-L, Penttinen H, Utriainen M, Vehmanen L, et al. Health-related Quality of Life of Breast Cancer Survivors Attending an Exercise Intervention Study: A Five-year Follow-up. In vivo (Athens, Greece). 2020;34(2):667-74.

30. Schleinitz MD, DePalo D, Blume J, Stein M**.** Can differences in breast cancer utilities explain disparities in breast cancer care? J Gen Intern Med. 2006;21(12):1253-60.

31. Songtish D, Praditsitthikorn N, Teerawattananon Y**.** A Cost-Utility Analysis Comparing Standard Axillary Lymph Node Dissection with Sentinel Lymph Node Biopsy in Patients with Early Stage Breast Cancer in Thailand. Value Health Reg Issues. 2014;3:59-66.

32. Swanick CW, Lei X, Xu Y, Shen Y, Goodwin NA, Smith GL, et al. Long-term Patient-Reported Outcomes in Older Breast Cancer Survivors: A Population-Based Survey Study. International journal of radiation oncology, biology, physics. 2018;100(4):882-90.

33. Tanaka K, Tachi T, Hori A, Osawa T, Nagaya K, Makino T, et al. Cost utility analysis of pharmacist counseling care for breast cancer chemotherapy outpatients. Die Pharmazie. 2019;74(7):439-42.

34. Timmers JM, Damen JA, Pijnappel RM, Verbeek AL, den Heeten GJ, Adang EM, Broeders MJ**.** Cost-effectiveness of non-invasive assessment in the Dutch breast cancer screening program versus usual care: a randomized controlled trial. Can J Public Health. 2014;105(5):e342-7.

35. Tosteson AN, Fryback DG, Hammond CS, Hanna LG, Grove MR, Brown M, et al. Consequences of false-positive screening mammograms. JAMA Intern Med. 2014;174(6):954-61.

36. Tran TXM, Jung S-Y, Lee E-G, Cho H, Cho J, Lee E, et al. Long-term trajectory of postoperative health-related quality of life in young breast cancer patients: a 15-year follow-up study. Journal of cancer survivorship : research and practice. 2022.

37. Velikova G, Williams LJ, Willis S, Dixon JM, Loncaster J, Hatton M, et al. Quality of life after postmastectomy radiotherapy in patients with intermediate-risk breast cancer (SUPREMO): 2-year follow-up results of a randomised controlled trial. The Lancet. Oncology. 2018;19(11):1516-29.

38. Youens D, Halkett G, Wright C, O'Connor M, Schofield P, Jefford M, et al. Assessing the cost-effectiveness of RT Prepare: A radiation therapist-delivered intervention for reducing psychological distress prior to radiotherapy. Psycho-oncology. 2019;28(5):1110-8.

39. Zigman T, Luksa I, Mihaljevic G, Zarkovic M, Kirac I, Vrdoljak DV, Serman L**.** Defining health-related quality of life in localized and advanced stages of breast cancer - the first step towards hereditary cancer genetic counseling. Acta clinica Croatica. 2020;59(2):209-15.
