# Supplementary material for: Patient preferences for breast cancer screening: a systematic review update to inform recommendations by the Canadian Task Force on Preventive Health Care
Source: Syst Rev. 2024 May 28;13:140. doi: 10.1186/s13643-024-02539-8 (PMC11134964; doi:10.1186/s13643-024-02539-8)
Supplement: Supplementary file 3 — Supplementary Material 3. Data sets for studies not reporting health-state utilities. [file 13643_2024_2539_MOESM3_ESM.docx]

**Patient preferences for breast cancer screening: a systematic review update** **to inform recommendations by the Canadian Task Force on Preventive Health Care**

Jennifer Pillay, Samantha Guitard, Sholeh Rahman, Sabrina Saba, Ashiqur Rahman, Liza Bialy, Nicole Gehring, Maria Tan, Alex Melton, Lisa Hartling; Alberta Research Centre for Health Evidence, Faculty of Medicine and Dentistry, University of Alberta, Edmonton, Alberta, Canada.

**Supplementary file 3. Data sets for studies not reporting health-state utilities**

**CONTENTS**

Summary of Findings Tables p. 2

Findings narratives for 50 to 69-year-olds, indirect evidence on weighing benefits and harms p. 12

Study characteristics and results data for all studies (16 newly added studies since 2018 review are highlighted in grey) p. 16

Risk of bias summary table p. 60

Included studies and associated papers p. 62

**Table S3.1 Summary of Findings: Non-HSUV Studies Providing Direct Preference Data, by outcome comparison**

| **Included studies;**  **Sample size** | **Findings** | **GRADE^ꝉ^** | **What does the evidence say?** |
| --- | --- | --- | --- |
| **All-cause versus BC Mortality** | | | |
| **Across all ages** | | | |
| 2 studies  Davey 2005, Reder 2017  N=1019 | An RCT of an online decision aid in those aged 50, at first invitation (n=913; 50 yrs, 33% previously screened) (1 fewer in 200 BC deaths over 20 years and no reduction in all-cause mortality vs. 50 FP and 1 overdiagnosed in 200 screened) (moderate ROB) and computer-assisted telephone interviews with convenience sample at primary care clinic (n=106; 45-70 yrs; 91% previous screening) with sequential presentation of four screening scenarios with first three indicating i) BC morality using relative terms (34% reduction), ii) BC mortality using absolute terms (4 vs 6 in 1000 over 10 years), and iii) all-cause mortality “screening will not increase your chance of living longer”. (none of the first 3 scenarios mentioned harms) (low ROB)   - In the RCT there were positive intentions to screen for 82% after reading about a reduction in BC but not all-cause mortality, and in the interviews, women were somewhat less willing to be screened after being presented with information on all-cause mortality (definitely: 53% and probably: 31%) after that on breast-cancer mortality (definitely: 78% and probably: 14%). - In the RCT 83% had a positive attitude about screening, and during the interviews only 16% of participants stated that the information on all-cause mortality should definitely be presented to women (40% stated probably), compared with 73% and 20% when asked about the absolute effects of BC mortality. - At 3 months, 65% of the women in the RCT had attended screening. | ⊕⊕⊝⊝  LOW  Indirectness due to one population of screeners and need to rely on intentions data in the RCT that also provided other information to women.  Imprecision around the “large majority” | For patients 50 or older, at least a large majority (>75%) of patients may think that reducing breast-cancer mortality is beneficial even if there is no impact on all-cause mortality.  The evidence was limited to relatively high rates of breast-cancer mortality reductions (2 and 5 fewer per 1000). |
| **BC Mortality versus Overdiagnosis** | | | |
| **Across all ages** | | | |
| 5 studies  Stiggelbout 2020, Hersch 2013, Sicsic 2018, Van den Bruel 2015, Wong 2015  N=2,652 (range 50-810) | Main analysis: Community samples using i) an online survey using choice sets varying by rates of overdiagnosis and its required treatments (Netherlands and Australia; n=803; ages 45-75 [mean 58.3 ± 8.9 yrs; oversampled 45-50 yrs; 69% previous screening/29% previous FP]), ii) focus groups (Australia; n=50, ages 40-79 [38% <50 yrs]; 62% previous screening), and iii) an online discrete choice experiment (DCE; France n=810, ages 40-74 [37% 40-49]; 58% screening in past 2 yrs).   - 50-57% (varying across types of treatment) would always participate in screening, even with a 1:6 ratio of breast cancer deaths avoided to cancers overdiagnosed. No associations between acceptance and age, previous experience of a FP or FP biopsy, or having a friend or relative with breast cancer. Previous screening associated with higher acceptance of overdiagnosis for all scenarios (P < 0.001). 33% correct on question asking for definition of the outcome. (low ROB) - 30% overdiagnosis (i.e., 11 among 38 cancers) was “acceptable and of limited impact” (on average 5:1); 50% overdiagnosis (i.e., 19 among 38 cancers; 10:1) thought to possibly deter some women, especially younger women, or necessitate careful consideration by others. (low ROB) - Mean 14.1 overdiagnosed cases acceptable for preventing 1 death from BC; a majority (>50%), large majority (≥75%) and almost all (≥90%) would accept <10:1, ≤6:1, and ≤4:1. Previous screening experience was not a significant predictor. (moderate ROB)   Two other studies at high risk of bias: an online survey eliciting simple trade-offs (UK; n=510; mean 46.9 yrs; 42% previously screened) and study asking about the relative importance of these outcomes when making decisions based on a decision aid (Hong Kong; n=90; mean age 54; very few previously screened [<20% heard of mammography]).   - A large majority would accept between 50 and 120 overdiagnoses per life saved (high risk of bias from up to 20% 18-35 yrs in sample). (high ROB) - 22% (BC mortality) and 5% (overdiagnosis) thought the data was important for decision making. (high ROB) | ⊕⊕⊕⊝  MODERATE  Indirectness (some limitation of understanding of this outcome) | For patients 40 or older, at least a majority (>50%) and possibly a large majority (>75%) of patients probably accept up to 6 cases of overdiagnoses to save one death from breast cancer. |
| **50 to 69-year-olds** | | | |
| 2 studies  Hersch 2015, Waller 2014  N=1,833 | RCT of community sample in Australia (n=879; 48-50 yrs; 0% screened over past 2 yrs) using decision aids with and without data on rates of overdiagnosis and survey in UK (n=954; 53-70 yrs; 91% previously screened) with same (3:1) ratio was presented in three differing formats (i.e., 3:1; 4000:1300, and 3 in 200 screened vs 1 in 200 screened). Relatively large benefit for BC mortality presented   - BC mortality (4 vs. 8 in 1000 over 20 yrs) and overdiagnosis (19 in 1000) were very important for 67% and 45% in intervention vs. 79% and 57% in control, i.e. 5:1 ratio did not appear to change the relative importance of the outcomes for decision making, but not direct trade-off (low ROB) - Intentions to probably/definitely screen with 3:1 were 92%, though there was a shift in intentions by one level (e.g. from definitely to probably) for 4.5% of women (9.1% for the simple 3:1 ratio group). 48% failed to understand that screening increases cancer diagnosis (low ROB) | ⊕⊕⊝⊝  LOW  Lack of consistency from heavy reliance on 1 study  Indirectness due to reliance on intentions and very limited understanding and lack of denominator in one study | For patients 50 and older, a large majority of women may accept at least 3 overdiagnoses to prevent one BC death though an upper limit was not examined. |
| **BC Mortality versus False Positives** | | | |
| **Across all ages** | | | |
| 3 studies  Schwartz 2000, Davey 2005, Wong 2015  N=675 (range 90-479) | - Population-based US survey (n=479; up to 20% <35 yrs): 80%, 63%, and 37% would accept 100, 500, or 10,000 or more FPs per life saved over a 10-year timeframe (scale 0, 10, 50, 100, 500, 1000, 10000) (high ROB) - Sequentially presenting information on BC mortality (2 lives saved per 1000 over 10 yrs), all-cause mortality (no life yrs gained) and FPs (50 in 1000 screened) during interviews within a primary care clinic in Australia (n=106; 45-70 yrs [28% ≤50]; 100% previously screened): neither willingness to screen or positive attitudes changed from before to after hearing about FPs (for willingness 78% vs. 79% and positive attitudes 85% vs. 79%). Based on the data presented, both BC mortality and FPs were very important or important for most (95% and 87%) (low ROB) - In decision aid with data about BC mortality (20% reduction) and FPs (10%), the information was important for decision making in 22% and 5% of participants, respectively (Hong Kong; n=90; mean age 54; very few previously screened [<20% heard of mammography]) (high ROB) | ⊕⊕⊝⊝  LOW  ROB  Imprecision about estimate of “majority” | For patients 40 or older, there may be considerable variation in preferences though almost all patients may accept that 25-50 and a majority may accept that a few hundred among 1000 experience a false positive to prevent one death from BC mortality over 10 yrs. |
| **40 to 49-year-olds** | | | |
| 2 studies  Lewis 2003, Nekhlyudov 2008  N=272 | US primary care clinic samples   - For 1 fewer BC deaths to 300 FPs per 1000 screened, 83% stated BC mortality was more (for 75% much more) important than FPs (low ROB); No effect of previous screening or high vs. under 5-year risk of cancer (n=179; 35-49 years; 75% previously screened) - 1 fewer BC deaths per 1000 screened increased intentions (previous intentions not reported) almost twice (56% vs. 29%) as often as did a FP rate of 100 per 1000; presented data on other outcomes which may have confounded intentions (moderate ROB) (n=93; 40-44 years; 0% previous screening) | ⊕⊕⊝⊝  LOW    Some (-0.5) inconsistency  Indirectness from possible confounding  Some (-0.5) imprecision | For patients in their 40s, at least a majority of patients probably accept at least 100 and may accept at least 300 FPs per life saved over 10-yrs. |
| **50 to 59-year-olds** | | | |
| 3 studies  Hersch 2015, Gyrd-Hansen 2000, Yasunaga 2007  N=1483 | - RCT in Australia (n=879; 48-50 yrs; 0% screened in past 2 years) using decision aids: BC mortality (4 vs. 8 dying in 1000 over 20 yrs) was very important for about 1.5 times (e.g. 79% vs. 52%) as many people as was the data on FPs (412 in 1000) (ratio 1:100), regardless of whether data on overdiagnosis was presented (low ROB) - DCE in community sample in Denmark (n=207; 50 yrs; starting age for screening): BC mortality was many times more influential for acceptance than FPs (preference weights 0.061 vs. -0.0003); 30% to 100% reduction in mortality may have influenced findings (low ROB) - Willingness to pay study in Japan (n=397; 50-59 yrs) for screening programs that reduced mortality by 20% with and without FPs (80 per 1000): reduced by about 25% when presented with the harms data indicating this information was an important consideration (high ROB) | ⊕⊕⊝⊝  LOW  ROB  Inconsistency | For patients 50-59 yrs of age, even in scenarios of relatively high reductions in BC mortality, FP rates of 80-120 or higher per 1000 may be important information for a large minority of patients when making decisions about screening. |
| **BC Mortality versus FP biopsies** | | | |
| **Across ages** | | | |
| 1 study  Sicsic 2018  n=812 | - DCE (community sample in France n=810, ages 40-74 [37% 40-49]; 58% screening in past 2 yrs): mean willingness-to-accept value was 47.8 FP biopsies per prevented BC death when screening until age 74; 95% would accept between 6.7 and 127.3 FP biopsies; 92% would accept 10 FP biopsies, 63% would accept 20, and 48% would accept 30 FP biopsies per life saved. Those with regular screening history were willing to accept more FPs (22% higher). (moderate ROB) | ⊕⊕⊝⊝  LOW  Lack of consistency  Imprecision around to 10-15 FP biopsies | For patients 40 or older, a large majority of patients may accept that between 10-15 people experience a FP biopsy to prevent one BC death over many yrs. This trade-off may be an overestimate for what is acceptable over a 10-year timeframe. |
| **Stage Distribution (reduced advanced disease) versus FPs** | | | |
| **Across ages** | | | |
| 3 studies  Bilger 2020, Ganott 2006, Jafri 2008  N=2,881 | - DCE among community sample in Singapore (n=400; 40-64 yrs, mean 52.1 ± 7.3; 35% previously screened) with attributes including stage distribution (i.e., BC cancer survival rates of 25%, 50%, 65%, and 90%) and FPs (5%, 15%, and 30%); when cancer survival changed from 25% to 90% (the author’s interpretation of a shift from late to early stage), 14.5% more (23% relative effects) participants would undergo screening; from 25% to 65% the change in acceptance increased by 9.9%. The number of people experiencing this change in advanced disease was not considered (may be 1 unit to many). When the FP rate was reduced from 30% to 5% (e.g. 25-unit change), uptake only increased 1.4% (2% in relative terms). In Singapore there are large out-of-pocket expenses which was noted by the authors as impacting uptake of screening. (low ROB) - Two US clinic samples (at screening visits) using same questionnaire: Willingness to accept more FPs (15% vs. 10%) in order for the chance that if cancer is diagnosed it may be detected earlier (described as 1 in 200 cancers found vs. 1 in 300) (i.e. 50 more FPs vs. 2 cancers detected earlier per 1000) (indirect outcome) - 97% White participants (n=1570; ≥40 yrs [41% 40-49 yrs]): 86% acceptable; small differences in subgroups of previously screened, previous FP or invasive procedures, age (<60 vs ≥60 yrs) and family history of breast cancer (high ROB) - Underserved and predominantly minority population (n=911; ≥ 40 yrs [32% aged 40-49]): more White than Black and Hispanic women agreed (76% vs. 54% and 59%) and fewer being unsure (11% vs. 27% and 24%) about the trade-off (high ROB at screening visit) - Poor understanding in both studies and all participants at screening visit; both high ROB | ⊕⊕⊝⊝  LOW  ROB  Indirectness for outcome | For patients 40 or older, a large majority of patients may accept that at least 25 people experience a FP to prevent one advanced stage cancer. |
| **Stage distribution (reduced advanced disease) versus FP biopsies** | | | |
| **Across ages** | | | |
| 2 studies  Ganott 2006, Jafri 2008  N=2,481 | Two US clinic samples (at screening visit) using same questionnaire: Willingness to accept more FP biopsies (1 in 60 to 1 in 40) in order for the chance that if cancer is diagnosed it may be detected earlier (described as 1 in 200 cancers found vs. 1 in 300) (i.e., 8 more FP biopsies to detect 2 cancers earlier, per 1000) (indirect outcome)   - 97% White participants (n=1570; ≥40 yrs [41% 40-49 yrs]): 82% agreed, with small differences in subgroups (see above) - Underserved and predominantly minority population (n=911; ≥ 40 yrs [32% aged 40-49]): more White than Black and Hispanic women agreed (75% vs. 53% and 65%) and fewer being unsure (11% vs. 27% and 24%) - Poor understanding in both studies and all participants at screening visit; both high ROB | ⊕⊕⊝⊝  LOW  ROB  Indirectness for outcome and whether findings apply to all ethnicities | For patients 40 or older, a large majority of patients may accept that at least 4 people experience a FP biopsy to prevent one advanced stage cancer. |
| **Treatment burden (reduced mastectomy) versus FPs** | | | |
| **Across ages** | | | |
| 1 study  Bilger 2020  N=400 | - DCE in community sample in Singapore (52.1 ± 7.3 yrs; 35% previously screened): type of surgery (3 levels: no change, changes in feel/appearance of breast, or lose an entire breast) for comparisons with FPs (5%, 15%, and 30%): compared with no change, not losing a breast increased acceptance by 4.8% (7.5% in relative terms) and not having a change in appearance increased acceptance by 2.1%, compared with the increased acceptance of 1.4% (2.1% in relative terms) with a large change in FPs from 30% to 5% (i.e. 25 units) (low ROB). | ⊕⊕⊝⊝  LOW  Lack of consistency  Imprecision about the estimate of majority | For patients 40 or older, avoiding mastectomy may be much more important than experiencing a FP for a majority of patients. |

ꝉ GRADE, Grading of Recommendations Assessment, Development and Evaluation. Reasons for rating down certainty: a=risk of bias, b=inconsistency/lack of consistency, c=indirectness, d=imprecision; use of capitals indicates there was very serious concern for the domain; for three exposures (positive screening mammography, true positive result, interval cancer) there was only some concern for two of the domains

BC, breast cancer; DCE, discrete choice experiment; FP, false positive; RCT, randomized controlled trial; ROB, risk of bias; Yrs, years

**Table S3.2. Summary of Findings: Non-HSUV Studies Providing Indirect Data from Making Inferences from** **Attitudes, Intentions, And Behaviors, by age and judgement of net benefit presented**

| **Included studies;**  **Sample size** | **Findings** | **GRADE^ꝉ^** | **What does the evidence say?** |
| --- | --- | --- | --- |
| **40 to 49-year-olds (10 studies [N=7,405] including 4 new to this update [N=3,814])** | | | |
| **Relatively high net benefit scenario** | | | |
| 6 studies  Laza-Vásquez 2022, Roberto 2020, Schonberg 2020a, Seitz 2016, Driedger 2017, Elkin 2017  N = 4,826 | 2 community samples, 3 primary care clinics, 1 using screening program lists; previously screening 40-74%. Benefits presented only using relative effects (e.g. 20% reduction) or a natural frequency that was judged high (e.g., 1 in 200 prevented BC deaths), and/or not presenting any mention or numerical information on overdiagnosis (n=3); 4 studies provided patients with their own predicted risk for BC; in 3 there was also the opportunity to discuss the information during a clinic visit (1 low ROB, 5 high ROB)   - Attitudes: 3 studies: high (88% and 92%) in two studies (N=1,388; 1 with 40-59yrs), but also positive attitudes (62.7%) towards personalized screening (e.g., limiting screening to higher-risk women in their 40s); 1 (n=168) reported that 83% of participants strongly agreed/agreed that benefits outweigh the risks - Intentions: 5 studies: in 1 study (ages 40-59) 98-99% (across 2 interventions) had positive intentions; in 3 studies (40s) fewer patients had intentions (e.g., 77% over next 6 months, 19-31% would not screen/would wait until 50s, mean score of 68 ± 40 on 0-100 scale); high (92%) for personalized screening in 1 study; in Canadian study (n=46), 21% 35-49 yrs stated age 40 was when screening should start - Attendance: 2 studies: at 16 ± 5.4 months 42% in US study (36% non-Caucasian) and 84% at an unknown follow-up 1 study (40-459 yrs) - Subgroups: Data in 3 studies by risk groups were somewhat inconsistent but at most showing small differences (e.g. n=2,918, 19-24% low risk vs. 24-31% intending not to screen in 40s | ⊕⊕⊝⊝  LOW  Indirectness  Inconsistency | In a relatively high net benefit scenario, a majority but possibly not a large majority of patients in their 40s may weigh the benefits as greater than the harms from screening. Preferences may be similar for patients with different levels of breast cancer risk. |
| **Relatively moderate net benefit scenario** | | | |
| 1 study  Valentine 2022  N=2,120 | Community US sample 49.5 ± 7.8 yrs (72% previous screening) with complex intervention having 4 stages (net benefit: 2 fewer BC deaths, 160 FPs and 20 overdiagnoses in 1000 over 11 yrs) (low ROB)   - Intentions: 88.5% of 40-49 yrs had intentions at baseline; across all participants, preferences lowered after each subsequent stage of the intervention, reducing to 53% after the first stage (didactic information with benefit/harms) then to 28-30% after all four stages (including a detailed explanation of overdiagnosis and narrative of a biopsy experience). | ⊕⊝⊝⊝  VERY LOW  Lack of consistency  Very serious indirectness (mean age 49.5)  ⊕⊕⊝⊝  LOW  Lack of consistency  Indirectness | In a relatively moderate net benefit scenario, it is unclear how patients in their 40s weigh the benefits as greater than the harms from screening.  Information on overdiagnosis may be quite important for many women. |
| **Relatively low net benefit scenario** | | | |
| 3 studies  Saver 2017, Mathieu 2010, Paul 2008  N=459 | Largely community samples provided with information in deliberative jury (New Zealand; n=12; 55% previous screening), video intervention (US; n=35; 91% previous screen/37% previous FP), and decision aid (Australia; n=412; 27% previous screening. (all moderate ROB). Jury and decision aid: among 1000 over 10 years: 2 vs 2.5 BC mortality, 12.8 vs 13.3 all-cause mortality; 239 FPs (“abnormal mammogram that requires extra testing”), 7 extra diagnoses (some of which may never affect health but be treated); comparison with 2 fewer BC deaths per 1000 in 50s.   - Attitudes: 2 studies: 10/11 voters changed their mind from *for* to *against* provision of screening for 40-49 yrs; video intervention reduced scores about the benefits being greater than the harms (-0.65 on 5-point scale; [p <0.001]) - Intentions: 2 studies: video lowered (pre: 85% intended/6% unsure vs. post: 49% intended/20% unsure) and after decision aid 39% did not intend to start screening (18% unsure); 94% had adequate knowledge after decision aid | ⊕⊕⊝⊝  LOW  Indirectness  Imprecision (around majority) | In a relatively low net benefit scenario, a majority of patients in their 40s may not weigh the benefits as greater than the harms from screening. |
| **50 to 69-year-olds (16 studies [N=27,148] including 6 new to this update [N=4,924])** | | | |
| **Relatively high net benefit scenario** | | | |
| **Focus on 50-year-olds** | | | |
| 5 studies  Berens 2015, Gummersbach 2015, Perez-Lacasta 2019 (associated paper Lo´pez-Panisello 2023), Reder 2017, Roberto 2020  N=6,904 | European studies mainly recruiting from organized screening program lists; most 1 in 200 lives saved with 1-2 overdiagnoses (1 high ROB study; low to moderate knowledge scores across studies)   - Attitudes: 4 studies: positive attitudes in 74% to 94% - Intentions: 5 studies: intentions to screen 82% to 83% in 3 studies, and in 2 positive intentions (e.g. above mid-point in scale) in 82% and 99%. 1 study found that intentions reduced at a 3-month follow-up (from 82% to 65%) - Attendance: 2 studies: 63% at 3 months and 84% at unknown timing - Subgroups: in Germany: those with previous (2%) or a family history (17%) of BC were more willing to screen (97% vs. 73%; p=0.009) (n=353); immigrants had more positive attitudes (mean scores 4.6 to 5.1 vs. 4.2 on -8 to +8 scale) but lower intentions (75%-77% vs. 83% for non-immigrants) - Mediation: knowledge directly worsened attitude towards screening (p = 0.002), but not intentions (p = 0.334) | ⊕⊕⊕⊝  MODERATE  Indirectness | In a relatively high net benefit scenario, a large majority of 50-year-old patients probably weigh the benefits as greater than the harms from screening. |
| **Ongoing screening in 50-69 yrs** | | | |
| 6 studies  Waller 2013, Lawrence 2000, Toledo-Chavarri 2017, Driedger 2017, Bourmaud 2016, Haakenson 2006  N=16,864 (1 RCT 16,000) | Patients across a range of settings and ages with previous screening histories of around 75% (46% to 99%); 3 used focus groups, 2 RCTs comparing a decision aid (n=16,000) or an informative brochure (n=668) with standard invitation letters, and 1 validated a decision aid (n=71). (3 high ROB mainly for not having any numerical data on overdiagnosis; 3 moderate ROB for either quantifying qualitative results or having an inadequate description of overdiagnosis [specific to DCIS only])   - Attitudes: 2 studies: “few” focus-group participants changed their attitudes based on information on overdiagnosis; in Canadian study 35% 50-59 yrs said screening should start at 40, 29% age 50, and 35% uncertain - Intentions: 3 studies: 93% in one US study and described in two European qualitative studies as “remaining high overall” and “a vast majority of those who had already considered screening (≥90%) would participate” - Attendance: 2 studies; 40.3% in large high ROB RCT from France (previous year 50%; no differences across ages); 98.3% attendance in US RCT (high ROB) | ⊕⊕⊕⊝  MODERATE  Indirectness  Some inconsistency from RCT in France but in context of little screening so not serious | In a relatively high net benefit scenario, a large majority of 50 to 69-year-old patients probably weigh the benefits as greater than the harms from screening. |
| **Relatively moderate net benefit scenario** | | | |
| **Focus on 50-year-olds** | | | |
| 1 study    Hersche 2015 (associated Hersche 2017 & 2021)  N=879 | RCT (n=879) among 48-50 yrs from community compared decision aids with and without data on overdiagnosis (i.e., 4 vs. 8 in 1000 dying of BC over 20 yrs vs. 412 FPs +/- 19 overdiagnoses per 1000) (low ROB; focus on results from overdiagnosis scenario)   - Attitudes: 69% and 81% positives attitude at 1 mo and 2 yrs - Intentions: 74% and 82% intentions to screen at 1 mo and 2 yrs - Attendance: 55% (self-reported) and 70% (via public records) at 2 yrs - Mediation: reduced positive intentions vs control group (87% at 1 mo) mediated by greater knowledge of overdiagnosis and the subsequent reduction in positive attitudes (adequate knowledge of overdiagnosis 55% at 1 mo) | ⊕⊕⊕⊝  MODERATE  Some concern about lack of consistency but large low ROB study so did not rate down  Indirectness | In a relatively moderate net benefit scenario, a majority and possibly a large majority of patients 50 years old probably weigh the benefits as greater than the harms from screening. |
| **Ongoing screening in 50 to 69-year-olds** | | | |
| 1 study  Baena-Canada 2018  N=20 | Citizen’s jury (n=20 enrolled with 15 attending some sessions and 13 voting) of eligible screening program participants in Spain (data: range 1 fewer BC deaths in 235 to 2 life saved in 1 or 2000; 4% fewer need for chemotherapy,5% fewer with advanced stage, 3-10% FPs, 1 in 77 to 10 in 1000 overdiagnoses) (high ROB)  Attitudes: 85% agreed that health authority should continue to offer screening to those 50-69 yrs (100% favorable at baseline) | ⊕⊝⊝⊝  VERY LOW  ROB  Lack of consistency  Indirectness | In a relatively moderate net benefit scenario, it is uncertain how 50 to 69-year-old patients weigh the benefits versus harms from screening. |
| **Relatively low net benefit scenario** | | | |
| **Focus on 50 to 59-year-olds** | | | |
| 3 studies  Henriksen 2015, Valentine 2022, Baena-Canada 2015  N=2,481 | Qualitative study in a primary care clinic in Denmark (n=6), an RCT of 4-stage intervention in the US using a public survey platform (n=2,120; 49.5 [7.8] yrs; 72% previous screening) and an RCT on screening leaflets among screening program attendees in Spain (n=355; mean 54 yrs; 100% screened [at visit]) at moderate, low and high (screening attenders; 18% well informed) ROB   - Attitudes: 1 study: 99% positive attitude (n=355) based on leaflet based on the 2008 Cochrane review risk estimates (200 FPs and 10 overdiagnoses to prevent 1 BC death in 2000 over 10 yrs) - Intentions: 3 studies: 99% intended to screen (n=355); intentions reduced in 40-59 yrs from 84% to 53% after the first stage (didactic information with benefit/harms [2 fewer BC deaths, 160 FPs and 20 overdiagnoses in 1000 over 11 years] then to 28-30% after all four stages of complex intervention with 4 stages of information; 1 of 6 reconsidered their decision to start screening when invited based on information on overdiagnosis; no improvement in numerical knowledge | ⊕⊕⊝⊝  LOW  Inconsistency  Indirectness | In a relatively low net benefit scenario, a large majority of 50 to 59-year-old patients may weigh the benefits as greater than the harms from screening. |
| **70 yrs and older (6 studies [N=1,441] including 4 new to this update [N=662])** | | | |
| **Relatively high net benefit scenario** | | | |
| **70 yrs and older** | | | |
| 2 studies  Pappadis 2018, Braithwaite 2023  N=73 | Evaluation of a tailored decision aid (n=14) and mixed-methods study (n=59) using qualitative narratives focused on overdiagnosis (10% to 30% of cancers diagnosed via figures and scenarios) (high ROB)   - Intentions: 2 studies: 1 of 11 analyzed would stop screening; 44% supported mammograms and 49% intended to continue screening (20% and 37% for those indicating good understanding of overdiagnosis) | ⊕⊝⊝⊝  VERY LOW  ROB  Indirectness  Imprecision | Under relatively high net benefit scenarios, it is uncertain how patients 70 years old and over weigh the benefits and harms. |
| **Relatively moderate-to-low net benefit scenario** | | | |
| **70 to 71-year-olds** | | | |
| 1 study  Mathieu 2007  N=734 | RCT (n=734) in Australia compared a decision aid to a standard brochure among 70 to 71-year-old recent screeners (2 lives saved per 1000 over 10 yrs vs. 135 FPs, 15 overdiagnoses and 9 interval cancers) (low ROB)   - Attitudes: 95% positive attitudes - Intentions: 86% intended (with 5% more unsure) to continue screening - Attendance: at 1 mo, 6% had participated and 76% indicated they were in the process of arranging to be screened | ⊕⊕⊕⊝  MODERATE  Indirectness  Some concern about lack of consistency but large low ROB trial so did not rate down | In a moderate-to-low net benefit scenario, a large majority of patients 70-71 years of age who have recently screened probably think the benefits outweigh the harms for continuing to screen. |
| **75 yrs and older** | | | |
| 3 studies  Schonberg 2020b, Schonberg 2014, Cadet 2021a  N=634 | One RCT (n=546; age 79.8 [3.7]) and 2 pre-post trials (N=88) among US primary care clinics measured screening intentions and, in 2, screening attendance after exposure to a decision aid for recent screeners aged 75 and older. In all there was the opportunity to use the decision aid during a clinic visit, and aids depicted a reduction of BC mortality by 1 per 1000 screened (e.g. 3 vs. 4 die in 1000) but in 2 the time horizon was 5 yrs whereas in 1 it was (n=43) it was 10 yrs. 2 mentioned 4 in 1000 would avoid a large cancer and ranges of 100-200 FPs and 11-13 overdiagnoses per 1000 (2 low and 1 moderate ROB)   - Intentions: 3 studies: intentions reduced (by ≥1 level on 15-point scale) for 24.5% (n=546); 56% intentions to continue screening (vs. 82% at pre-test) (n=45); in 18 medical records at 6 mos, 67% noted continuing screening, 22% discontinuation and 22% indecision - Attendance: 2 studies: 51% at 18 mos vs. 100% 2 yrs prior (n=546); 63% at 15-mos vs. 85% 2-yrs prior (n=45) - Subgroups: no effects on attendance by patient age, educational level, life expectancy, or breast cancer risk (≥3 vs <3% 5-yr risk) (n=546); those having <9 yrs life expectancy had lower intentions (50% vs. 63%) and attendance (52% vs. 78%)(n=45) | ⊕⊕⊝⊝  LOW  Indirectness  Imprecision | For patients aged 75 years to their early 80s who have recently screened, a majority but possibly not a large majority may weigh the benefits as greater than the harms for continuing to screen. It is unclear what impact life expectancy has on this preference. |

ꝉ GRADE, Grading of Recommendations Assessment, Development and Evaluation. Reasons for rating down certainty: a=risk of bias, b=inconsistency/lack of consistency, c=indirectness, d=imprecision; use of capitals indicates there was very serious concern for the domain; for three exposures (positive screening mammography, true positive result, interval cancer) there was only some concern for two of the domains

BC, breast cancer; DCIS, ductal carcinoma in situ; FP, false positive; Mos, months; RCT, randomized controlled trial; ROB, risk of bias; Yrs, years

**Findings Narratives for 50 to 69-year-olds indirect evidence on weighing benefits and harms**

Sixteen studies (N=27,148) were included in this update, with six studies (N=4,924) added from the update search. Additionally, one of the new studies had one associated paper and one of the original studies had two associated papers. Four studies were conducted in Spain, three in each of Germany and the USA, and one each in Italy, Canada, Australia, Denmark, the UK, and France. Studies evaluated decision aids (n=7), enhanced screening program brochures (n=5), and investigator developed materials (n=4). Eleven studies used quantitative designs and five (including one deliberative jury) used qualitative designs with quantifying data relevant for this review. Seven studies focused on patients 50 years of age whereas nine included a broader age range. Studies differed in their estimates of the benefits and harms, and our analysis accounts for this variation as well as the age(s) of focus.

**Relatively low net benefit scenario**

Focus on 50 to 59-year-olds

**3 studies (N=2,481) Henriksen 2015, Valentine 2022, Baena-Canada 2015**

Three studies (N=2,481) contributed to this synthesis; a qualitative study in a primary care clinic in Denmark (n=6), an RCT in the US using a public survey platform (n=2,120; 49.5 [7.8]) and an RCT among screening program attendees in Spain (n=355; mean age 54) at moderate, low and high risk of bias, respectively.

The RCT among screening attenders in Spain found that a leaflet based on the 2008 Cochrane review risk estimates (200 FPs and 10 overdiagnoses to prevent 1 BC death in 2000 over 10 yrs) led to 99% of participants to report a positive attitude towards continuing to screen. Intentions to screen were also very high (99%) in the study, though only 18% of the intervention arm was considered well informed. In the larger RCT, before being presented with a complex intervention with four sequential formats of data (presenting 2 fewer BC deaths, 160 FPs and 20 overdiagnoses in 1000 over 11 yrs), screening intentions were quite high (84.9%) and higher for those aged <50 versus ≥50 yrs (88.5% vs. 80.4%). Preferences for screening lowered after each subsequent stage of the intervention, reducing to 53% after the first stage (didactic information with benefit/harms) then to 28-30% after all four stages (including a detailed explanation of overdiagnosis and narrative on a patient’s experience of a biopsy). The data for 50 to 59-year-olds was not presented separately from that for 40 to 49-year-olds.

The small qualitative study discussed starting to screen once 45 to 49-year-olds turn 50. Under the scenario of 180 FPs and 10 overdiagnoses to save 1 BC death among 2000 screened over 10 yrs, one (17%) woman reconsidered her decision (to start screening when invited) based on information on overdiagnosis.

**Relatively moderate net benefit scenario**

Focus on 50-year-olds

**1 study N=879 Hersche 2015**

One low risk of bias RCT (n=879) among 48 to 50-year-olds in the community compared decision aids with and without data on overdiagnosis. The original data at ≤1-month follow-up data on attitudes and intentions has for this update been supplemented with data (including attendance) at 6 months, and 1 and 2 yrs. When providing a moderate net benefit scenario (i.e., 4 vs. 8 in 1000 dying of BC over 20 yrs vs. 412 FPs and 19 overdiagnoses per 1000), at initial follow-up 69% had positive attitudes and 74% had intentions to screen compared with 83% and 87%, respectively, in the control group. At 2 yrs, positive attitudes and intentions were still high (81% and 82%) though only half of the participance attended (55% [self-reported] and 70% [via public records]). Another associated paper describing mediation analysis found that the reduced positive intentions in the intervention group were in fact mediated by greater knowledge of overdiagnosis and the subsequent reduction in positive attitudes. Though conceptual understanding of overdiagnosis was improved by the decision aid, and remained higher than the control group over time, there was nevertheless only adequate knowledge of this outcome in 55% at 1 month and 26% at 2 yrs.

Ongoing screening in 50-69 yrs

**1 study N=20 Baena-Canada 2018**

A newly added study reported on a citizen’s jury (n=20 enrolled with 15 attending some sessions and 13 voting) of eligible screening program participants in Spain. Variable estimates for the outcomes of BC mortality, FPs and overdiagnosis were included due to provision of data from multiple sources (e.g. British review with higher net benefit [number needed to screen of 235] and Cochrane review with lower net benefit of 1 in 1000 or 20000 and there was inclusion of data on potential benefits for less aggressive treatment and lower numbers of advanced stage disease. At baseline 100% of the jury members had a favorable opinion on screening. When asked after the sessions if the health authority should continue to offer screening to those 50-69 year of age, 85% agreed. Exploration for the reasons for the decision for screening did not confirm that all participants focused on the efficacy of screening, with some referring to the “public good” and supporting “women’s freedom”.

**Relatively high net benefit scenario**

Focus on 50-year-olds

**5 studies N=6,904 Berens 2015, Gummersbach 2015, Perez-Lacasta 2019, Reder 2017, Roberto 2020**

Five European studies (N=6,904; 4 new to this update) examined preferences of patients at age 50 (45 yrs in one at first invitation in Italy) for screening programs judged as offering relatively high net benefit scenarios, mostly due to providing estimates of 1 fewer BC deaths in 200 screened usually over 20 yrs. All but one study recruited patients identified from organized screening program lists. Four studies were rated at low or moderate risk of bias, and one as high risk from high missing data and inadequate measurement of outcomes (defining positive attitudes and intentions).

Positive attitudes were reported by a large majority of patients (76% to 94%) in four studies. When asked within a month of the intervention, in three studies 82% to 83% of patients indicated their intent to screen and in another two studies there were positive intentions (e.g. above mid-point in scale) in 82 and 99% of patients. One study found that intentions reduced at a 3-month follow-up (from 82% to 65%). The two studies reported attendances of 63% at 3 months and 84% at an unreported timepoint. One study (n=353) in Germany examined subgroup effects based on risk for cancer; those with previous (2%) or a family history (17%) of BC were more willing to screen (97% vs. 73%; p=0.009). Another study (n=4,113) assessing subgroup effects based on immigration status in Germany found that immigrants more positive attitudes (mean scores 4.6 to 5.1 vs. 4.2 on -8 to +8 scale) but lower intentions (75% to 77% vs. 83% for non-immigrants).

Knowledge scores were reported across all studies, with three reporting 32%-44% of patients having adequate knowledge (e.g. ≥11 of 22 points) and two reporting roughly mid-points when scales were used (5.5 on 0-10 and 3.96 on 0-7 scales). An associated paper of one study (n=524)—analyzing direct, indirect, and moderating effects between information, knowledge and attitude or intentions—found that higher knowledge levels significantly worsened the patients’ attitude towards screening. Another study (n=4,113) assessing subgroup effects based on immigration status in Germany found that immigrants had lower knowledge scores (sufficient in 25% vs. 33%).

Ongoing screening in 50-69 yrs

**6 studies N=16,864 (1 RCT n=16,000) Waller 2013, Lawrence 2000, Toledo-Chavarri 2017, Driedger 2017, Bourmaud 2016, Haakenson 2006**

Six studies (N=16,864) from the previous review focused on patients across a range of settings and ages with previous screening histories of around 75% (46% to 99%) and presented information judged as portraying a relatively high net benefit. Three of the studies used focus groups for data collection, two were RCTs comparing a decision aid (n=16,000) or an informative brochure (n=668) with standard invitation letters, and another was a validation study of a decision aid in a US sample of European women (n=71). One study included some (23%) patients under 50 and another reported data by age for those 35-49 (as included in the 40 to 49-year data set) and 50-59 yrs. Three studies were rated at high risk of bias mainly for not having any numerical data on overdiagnosis, and three were rated as moderate risk for either quantifying qualitative results or having an inadequate description of overdiagnosis (specific to DCIS only).

Only two studies reported data relevant to attitudes. Authors of one study (77% regular and 22% not regular screeners) stating that a “few” focus-group participants changed their attitudes based on information on overdiagnosis. The other study (n=46; high risk of bias) from Canada asked at which age screening should start, with 35% of 50-59 yrs old saying age 40, 29% saying age 50, and 35% being uncertain. Intentions to screen when provided with data on overdiagnosis were 93% in one US study and described in two European qualitative studies as “remaining high overall” and “a vast majority of those who had already considered screening (≥90%) would participate”. The large RCT from France reported lower attendance in the intervention (40.3%) versus control (42.1%) group in the 12 months following the invitation (p=0.02; previous year attendance was 50%). In this trial, neither 50-59 (aOR 1.0) or 60-74 (aOR 1.02) age groups were associated with differing attendance (p=0.21), but previous attendance for screening (aOR = 15.9 [95% CI 14.2 to 17.4]) was independently associated with attendance. The decision aid did not present information on overdiagnosis. The other RCT from the US reported that only 1.7% did not attend their scheduled mammogram (1 month after their invitation); this study used relative risks (21-30% reduction) for BC mortality data, used a relatively low rate of FPs 10-20% and FP biopsies (8-10% of FPs) and no information on overdiagnosis.

**Study Characteristics and Results Data**

**Table S3.3. Direct Preference Data from Mixed Ages**

| **Study, Country**  **Study description**  **Recruitment setting**  **Sample size; Age; Insured; White (other race/ethnicities); dense breasts; other risk factors**  **Screening history**  **History of FP or breast surgery**  **Risk of bias (ROB)** | | **Study data e.g. communicated outcomes of screening, attributes and their levels**  **Format of information and definitions provided**  **Data collection**  **Other information provided** | | **Findings** | **Knowledge and understanding (including relevant subgroup findings)** |
| --- | --- | --- | --- | --- | --- |
| **Direct data: specific to relative importance between individual outcomes** | | | | | |
| **Weighing all-cause and BC mortality** | | | | | |
| Reder 2017, Germany  RCT of online decision aid and usual care vs usual care (i.e., mammography screening program brochure) age 50 at first invitation  Recruitment setting: Organized program lists  N=913; 50; >90%; NR; NR; 15% first degree relative with breast cancer  Previous screening**:** 33%  History of FP or breast surgery: NR (not reported), NR  ROB: Moderate | **Communicated Outcomes of Screening (every 2 yrs for 20 yrs)**  **Decision aid:**   - BC mortality: 4 in 200 over 50 (without mammography) - BC mortality: 3 in 200 over 50 (with mammography) - **All-cause mortality: 21 vs 21 in 200 over 50** - FP: 50 in 200 screened - Overdiagnosis: 1 in 200 screened - Interval cancers: 3 in 200 screened   **Program brochure control**:  Same as above but in textual format and no description of all-cause mortality  Format & Definition: interactive online decision aid consisting of static information with crowd figure pictograms for screening vs not screening and interactive parts based on Mathieu et al. DA including discussion of uncertainties; FP: “abnormal mammogram requiring more tests, however the tests show the woman has no BC (“false alarm”)”; overdiagnosis: “would not have noticed their breast cancer during their lifetime”; interval cancers “women diagnosed with BC, although nothing found during screening”, usual care via program brochure had same quantitative data on all outcomes (via text) except for all-cause mortality  Data collection: baseline questionnaire (T1; 60% had received an invitation to screen with program brochure); post-intervention/right after reviewing intervention but no screening appointment date passed (T2); follow-up data collection 3-months after T2 (assuming screening would have been completed if undertaken)(T3)  Other information: true positives/detection rate, negative screening results (140 told they do not have cancer), interactive personal work sheet, evaluating information in favor of or against mammography screening, evaluating importance of information, making decisions about participation in mammography screening, input window for remaining questions, downloadable PDF summarizing information and personal responses | | **Positive intention to participate over next 3 months (T1 & T2; yes/no/undecided) and self-report of completed screening (T3):**  Intervention:  T1: 87%; T2: 82%; T3: 65%  Control:  T1: 86%; T2: 82%; T3: 67%  For intention at baseline and uptake at T3 there were no significant differences between the groups. At T2 the proportion of those who did not want to participate in the screening was higher in the DA group (18.1%) than in the control group (10.0%). Women in the control group had higher odds to have a positive intention (OR 2.00, 95% CI 1.21-3.29) than women in the DA group  **Positive attitude (4 5-point questions; ≥0 on scale -8 to 8):**  Intervention:  T1: 90%; T2: 83%; T3: 84%  Control:  T1: 89%; T2: 88%; T3: 85%  **Attitude score** (5-point scale), mean (SD):  Intervention:  T1: 3.39 (2.91); T2: 2.96 (3.41); T3: 2.84 (3.51)  Control:  T1: 3.33 (2.88); T2: 3.20 (2.94); T3: 3.39 (3.48) | | **Knowledge score (7 multiple choice questions, focused on conceptual knowledge; >3 considered adequate), mean (SD):**  Intervention:  T1: 2.73 (1.41); T2: 3.96 (1.33); T3: 3.57 (1.16)  Control:  T1: 2.79 (1.34); T2: 2.92 (1.40); T3: 3.21 (1.28)  **Informed intention to screen (i.e., adequate knowledge, positive attitude and intention to screen)**  **I**ntervention: T2: 61.5%, T3 39.8%  Control: T2 28.9%, T3 30.3% |
| Davey 2005, Australia  Computer-assisted telephone interviews with convenience sample at clinic using structured questions on all four scenarios with different numerical values  Recruitment setting: Primary care clinics  N=106; 45-70 (28% ≤50; 51% 51-60; 21% 61-70); 100%, NR, NR  Previous screening: 91%  History of FP or breast surgery: NR, NR  ROB: Low | **Communicated Outcomes of Screening (4 scenarios provided in sequential order):**   1. BC mortality (relative): RRR 34% for biennial for 10 yrs 2. BC mortality (absolute): 4 vs 6 in 1000 for biennial over 10 yrs 3. **All-cause mortality: This test reduces the chances of dying from BC. However, having the test will not increase the absolute chance of living a longer life**. 4. Above information (in absolute terms) plus FP: 50 in every 1000 for biennial for 10 yrs   Format & Definitions: telephone narratives of each scenario; all-cause mortality: this test will reduce the risk for dying from BC, but having the test will not increase the absolute chance of living a longer life; FPs: “because the test is not 100% accurate, some women (50/1000) will be asked to come back for further tests because their screening test was not normal. Women will eventually be shown through further tests that they do not have breast cancer (those who don’t have breast cancer, but need further tests before they get clearance)”. However, these women might experience worry, possible discomfort and inconvenience.  Data collection: presented with statements followed by decisional preference questions during telephone interview; at the end of the interview questions were asked about self-reported participation in screening  Other information: reassurance 940 in 1000, FN 2 in 1000 | | **Relative importance of outcomes:**  Very or important: BC mortality (absolute or RRR) 95%, FP 87%, overall benefits (1. to 3. in 2^nd^ column) vs harms (p<0.01)  **Willingness to be screened (as values presented sequentially)**:  Definitely:  78% using BC mortality absolute effects  53% after all-cause mortality  79% knowing limitations (FPs and FNs)  Probably:  14% using BC mortality absolute effects  31% after all-cause mortality  11% knowing limitations (FPs and FNs)  **Attitudes/feelings:**  Positive or very positive feelings: 85% using BC mortality ARR vs. 71% after ARR for all-cause mortality vs. 79% knowing limitations (harms)  Should the information be provided to others?  Absolute effects on BC mortality: definitely 73%, probably 20%  Effects on all-cause mortality: definitely 16%, probably 40% | |  |
| **Weighing BC mortality against overdiagnosis and FPs** | | | | | |
| Stiggelbout 2020, The Netherlands and Australia  Online survey asking women their maximum acceptable ratio of overdetection, per breast cancer death avoided, for four treatment scenarios (randomized order): mastectomy; lumpectomy; lumpectomy plus radiotherapy;  lumpectomy plus radiotherapy and hormone therapy.  Recruitment setting: public survey panel  N=803; 45-75 yrs (mean 58.3 ± 8.9; oversampled 45-50 less likely to have screening experience); NR; NR; NR  Screening history: 69% (with screening program); 31% (outside of program)  History of FP or breast surgery: 29% (for Australian sample n=400) & 11% breast biopsy; NR  ROB: Low | | **Communicated Outcomes of Screening & Overdetection Attribute levels (biennially starting at age 50 over 25 yrs):**   - BC mortality: 5 fewer per 1000 over 25 yrs - FP: 464 per 1000 over 25 yrs - Overdetection (levels of attribute to assess): used 0, 5, 30, 15, 2 (ratios 0:5, 1:1, 6:1, 3:1, 2:5)   Format & definitions: explanation and pictographs of benefits and harms with natural frequencies for BC mortality and FPs; 4 scenarios for treatment. FPs and overdiagnosis: “(FPs) occur in women who do not have breast cancer. These women have an abnormal screening result, but then extra tests show they do not have cancer. By contrast, in cases of over-detection the women do have breast cancer confirmed by further tests, so they get a cancer diagnosis and treatment…screening also leads to finding some breast cancers that are not harmful... some cancers found by screening would never cause problems anyway. Cancers like this may grow very slowly or just stay the same. Without screening, they would never be  noticed or cause any trouble...doctors cannot be sure which cancers will be harmless. Therefore, treatment is  recommended…”. Provided explicit information on the nature of invasive treatments, with descriptions of important associated short- and long-term side effects  Data collection: online survey using choice sets using pictographs with depiction of no screening versus screening, having fixed BC deaths but varying the number of overdetected cases from 0 to 30 (versus 5 BC deaths overdetection i.e. 0, 5, 30, 15); number of cancers overdetected was varied in the same order for each of 4 treatment scenarios (5 choices for each treatment scenario); grouped the overdetection variables into 4 categories: 0 = always screen (our highest ratio 30:5), 1 = screen depending on level of overdetection (15:5, 5:5, and 2:5), 2 = screen only if no overdetection (0:5), and 3 = never screen | | Highest overdetection ratio at which the respondent  would accept screening:   - 50-57% (varying across treatment scenarios) women indicated they would always participate in screening, even with a 1:6 ratio of breast cancer deaths avoided to cancers overdetected - 25-30% would screen “depending” (if overdetection was 1:1, 1:3 or 2:5 combined) - 17-21% would either never screen or only screen if there was no overdetection   Based on type of treatment: multivariate analysis of variance found only a slight effect (P = 0.12) for treatment scenario, with women accepting screening at the highest levels of overdetection for lumpectomy.  Subgroups:  No associations between acceptance and age, education, health literacy, or numeracy.  Previous screening: higher acceptance of overdetection, for all scenarios (P < 0.001);  Previous FPs and risk factors: No effect for having previous FP or FP biopsy, or having a friend or relative with breast cancer. | Comprehension of overdetection using 1 choice question (for defining overdiagnosis) and 7 true/false questions (scaled to 0-100:   - 33% understood (67% chose answer describing FPs) overdetection - Other questions: 66.4 [Netherlands] and 61.7 [Australia]   Those who correctly understood overdetection showed a lower acceptance of overdetection (e.g., in mastectomy scenario 37% of women who correctly picked the overdetection description would always screen versus 52% of women who (incorrectly) picked the FP; 49% vs 58% for lumpectomy. |
| Hersch 2013, Australia  Focus groups (age-stratified) using random and purposeful sampling with presentation and discussion on data  Recruitment setting: Community  N=50; 40-49: 38%  50-69: 32%  70-79: 30%, 100%, NR, NR, NR  Previous screening: 62% (some in all age groups)  History of FP or breast surgery: NR, NR  ROB: Low | | **Communicated Outcomes of Screening (over 10 yrs):**   - BC mortality:   40-49: 2 vs 2.5 in 1000  50-59: 4 vs 6 in 1000  60-69: 5 vs 8 in 1000  70s: 6 vs 8 in 1000   - Overdiagnosis: 3 scenarios based on “at most 4” (1-10%), 11 (30%) or 19 (50%) of 38 diagnosed with BC in 1000     Format & Definitions: In-depth presentation (text, pictures, plain language) with discussions, clarifications, paraphrasing by women, and assessment of understanding; included age stratified, evidence-based information on the benefit in terms of breast cancer mortality associated with mammography screening in Australia and portrayal of uncertainty in quantifying and detecting overdiagnosis such that treatments usually given. Overdiagnosis: an abnormality that is correctly diagnosed as cancer might grow very slowly or not at all, meaning that it will never develop into anything dangerous or even noticeable in the woman’s remaining lifetime. So, if the woman had not had screening, she would never have known that the cancer was even there...generally they are all treated straight away, so “overtreated” (with diagram to indicate no benefit in life yrs.  Data collection: during each of the focus groups baseline questionnaires and final questionnaires were completed; one to 4 weeks after focus groups a random selection of 18 participants were contacted for brief telephone interview to identify whether views about overdiagnosis had changed | | **Overdiagnosis vs BC mortality**:  1-10% (≤4 in 1000) “negligible”  30% (11 in 1000) “acceptable and limited impact”  50% (19 in 1000) “extremely high” and thought some may decline, delay (especially for younger), take more care about whether and when to screen. or be less concerned or rigorous about attending  **Subgroups**: regular screenees less concerned than those with no/less experience | **Conceptual knowledge after focus groups**:  **Who is more likely to be diagnosed with breast cancer?**:  70% agree on women who are screened with mammography (20% di not agree, 10% unsure)  **What is meant by the term “overdetection”? (scoring criteria for free response question)**:  60% full marks, 24% partial, 16% incorrect |
| Davey 2005, Australia  Computer-assisted telephone interviews with convenience sample at clinic using structured questions on all four scenarios with different numerical values  Recruitment setting: Primary care clinics  N=106; 45-70 (28% ≤50; 51% 51-60; 21% 61-70); 100%, NR, NR  Previous screening: 91%  History of FP or breast surgery: NR, NR  ROB: Low | | **Communicated Outcomes of Screening (4 scenarios provided in sequential order):**   1. BC mortality (relative): RRR 34% for biennial for 10 yrs 2. BC mortality (absolute): 4 vs 6 in 1000 for biennial over 10 yrs 3. All-cause mortality: This test reduces the chances of dying from BC. However, having the test will not increase the absolute chance of living a longer life. 4. FP: 50 in every 1000 for biennial for 10 yrs   Format & Definitions: telephone narratives of each scenario; all-cause mortality: this test will reduce the risk for dying from BC, but having the test will not increase the absolute chance of living a longer life; FPs: “because the test is not 100% accurate, some women (50/1000) will be asked to come back for further tests because their screening test was not normal. Women will eventually be shown through further tests that they do not have breast cancer (those who don’t have breast cancer, but need further tests before they get clearance)”. However, these women might experience worry, possible discomfort and inconvenience.  Data collection: presented with statements followed by decisional preference questions during telephone interview; at the end of the interview questions were asked about self-reported participation in screening  Other information: reassurance 940 in 1000, FN 2 in 1000 | | **Relative importance of outcomes:**  Very or important: BC mortality (absolute or RRR) 95%, FP 87%, overall benefits (1. to 3. in 2^nd^ column) vs harms (p<0.01)  **Willingness to be screened (as values presented sequentially)**:  Definitely:  78% using BC mortality absolute effects  53% after all-cause mortality  79% knowing limitations (FPs and FNs)  Probably:  14% using BC mortality absolute effects  31% after all-cause mortality  11% knowing limitations (FPs and FNs)  **Attitudes/feelings:**  Positive or very positive feelings: 85% using BC mortality ARR vs. 71% after ARR for all-cause mortality vs. 79% knowing limitations (harms)  Should the information be provided to others?  Absolute affects on BC mortality: definitely 73%, probably 20%  Affects on all-cause mortality: definitely 16%, probably 40% |  |
| Sicsic 2018, France  Discrete choice experiment via online survey incorporating 7 screening program attributes  Recruitment setting: Community  N=812; 40 (37% 40-49) -74; 94%; NR; NR; 27% family history  Previous screening: in past 2 yrs: 58%, 2-4 yrs 14%; more than 4 yrs: 13%, never 15%  History of FP or breast surgery: NR; NR  Ages 50-74 receive invites to national (free) program and ≥40 with ≥1 risk factor (not defined) can be prescribed screening  ROB: Moderate | | **Attributes and levels used (with regular screening up to 74 yrs)**  **•** BC mortality: screening levels: 10, 15, 20, 25 vs. no screening level: 30 breast cancer deaths expected per 1000  • FP requiring investigation/biopsy: screening levels 50, 100, 150, 200 vs no screening level 0 per 1000  • Overdiagnosis (of DCIS): screening levels 10, 50, 100, 150 vs. no screening level 0 per 1000 women  Format & Definitions: through thematic analysis 7 BC screening attributes identified to include in hypothetical screening program: BC mortality, false-positive mammography (“women undergoing unnecessary investigations because of suspicious findings on the mammograms that do not result in BC diagnosis…following abnormal mammography, which does not lead to the diagnosis of breast cancer; generates an invasive examination such as a biopsy”); overdiagnosis (“unnecessary treatment [surgery, chemotherapy and/or radiotherapy] for a precancerous tumor (i.e DCIS] detected by screening, which would probably not have evolved into breast cancer”), type of screening referral, number of screening tests, time spent traveling, and out of pocket cost; with options/levels for each attribute set by Cochrane review and another decision aid. Instead of 8192 possible scenarios a main-effects D-efficient design was generated to create 16 screening choice scenarios, with each built into 2 blocks of 8 scenarios. Participants were randomized to a block with the order of the choice scenarios also randomized. In each choice set there was 2 screening scenarios (A and B) and a “no screening”/”opt-out” option.  Data collection: Sent through online survey  Other information: additional attributes included DCE   - Type of screening referral: letter sent by local screening centre or doctor (none for no screening option) - Time spent travelling to radiological centre: 10, 30, 60, 90 (0 for no screening option) - Number of screenings performed: 6, 12, 18, 24 (0 for no screening) - Out of pocket cost after insurance reimbursement: 0, 30, 60, 60 (but refunded) Euros | | **Mean preferences/trade-offs (assuming all participants make same trade-offs)**  Willing to accept (WTA) on average 14.1 overdiagnosis cases (median 9.6; 95% CI 12.9 to 15.2)) and 47.8 false-positive (with biopsy) results (median 27.2; 95 CI 24.9 to 70.8) to avoid one BC-related death.  Overall: Generic screening intercept was positive and highly significant: maximum likelihood estimate mean [95% CI]: 9.43 [7.74 to 11.10] (P=0.01); indicating that women had a propensity to choose to be screened; that is, they placed higher weights on the benefits as compared with the harms of BC screening  **After accounting for preference heterogeneity**  Individual-specific WTA:  95% credibility intervals show that 95% of women would be WTA between 2.3 and 49.2 over-diagnosis cases and between 6.7 and 127.3 false-positives (with biopsy) to save one life from BC. Less than 50% of women would be willing to accept 10 overdiagnosis cases for one BC-related death avoided.  High heterogeneity in preferences.  Simulated acceptance as a function of benefits and harms balance (based on program with 2 to 10 overdiagnoses and 10 to 50 FPs per BC death avoided):  WTA overdiagnoses of 2, 4, 6, 8, 10 per 1 BC death avoided = 98%, 90%, 73%, 58%, 49%  WTA FPs with biopsy of 10, 20, 30, 40, 50 per 1 BC death avoided = 92%, 63%, 48%, 39%, 29%  **Subgroups:**  Those with regular screening history were willing to accept more FPs (22% higher)  Executives: up to 58% (vs. 49% overall) would accept overdiagnoses to BC death ratio of 10:1 and up to 48% (vs 29% overall) would accept FP with biopsy to BC death avoided ratio of 50:1. |  |
| Van den Bruel 2015, UK  Online survey with two scenarios to elicit trade-offs in research panel representative of UK for age and sex; results specific for BC but prostate and bowel also assessed  Recruitment setting: Community  N=510 women; >18 (mean 46.9); NR (100% in ≥50 yrs); NR “less ethnically diverse”, NR, 4.5% diagnosed with BC before  Previous screening: 42% (but wide age)  History of FP or breast surgery: NR, NR  ROB: High | | **Scenario 1**: In population of 1000 with 5% incidence of cancer and 1% cancer specific mortality, *1 will not die* from cancer because of screening (10% RRR). How many women being overdetected and overtreated would you accept for 1 woman to avoid dying from BC?  **Scenario 2**: In population of 1000 with 5% incidence of cancer and 1% cancer specific mortality, *5 will not die* from cancer because of screening (50% RRR). How many women being overdetected and overtreated would you accept for 5 women to avoid dying from BC?  *These authors prefer the term overdetection to avoid implications of overdiagnosis meaning misdiagnosis  Format & Definitions: written and graphical information; overdiagnosis/overdetection: definition with consequences of unnecessary tests and treatments and their harms. Description of overdetection unclear but involved cancers unnecessarily treated (limited description of what treatments would be)  Data collection: first survey was presented with questions about screening, followed by presentation of background information and screening scenarios; immediately after scenario of benefit presentation participants were asked about overdetection  Other information: absolute number of cases per year in the UK and a description of the treatment, including its adverse effects | | **Trade-offs:**  Scenario 1: median 150 (95% CI 120 to 197) for 1 averted BC death, accept no overdetection at all 5.1% (3.4 to 7.9), accept overdetection in complete population 10.2% (7.7 to 13.2) 95% CI  Scenario 2: median 313 (250 to 364) for 5 averted deaths 95% CI, accept no overdetection at all 3.5% (2.1 to 5.6) 95% CI, accept overdetection in complete population 13.9% (11.0 to 17.2) 95% CI  Acceptability of overdetection did not increase fivefold when benefit increased fivefold (i.e. benefits favor harms)  **Subgroups (indirect because across all cancer scenarios)**: people ≥ 50 accepted less overdetection than younger respondents (OR 1.93, 1.43-2.61 for low level of acceptance <30 overdiagnosis per death saved); people with at least a degree accepted more overdetection in higher benefit scenario |  |
| Schwartz 2000, USA  Mailed survey using random selection stratified to oversample screening age  Recruitment setting: Community  N=479; 18+ (25% <40, 10% >70); NR; 90%, NR, NR  Previous screening: 76%  History of FP or breast surgery: **16%**, NR  ROB: High | | No data provided but elicited  Format & Definitions: in print survey questions; FP: “in a woman who gets mammogram annually for the next 10 years, one of her mammograms will look like she has BC even though she does not”; overdiagnosis “We would like to ask your opinion about ductal carcinoma in situ or DCIS, a breast abnormality which can only be picked up by mammograms. Specialists are confused about DCIS because some-times it becomes invasive and sometimes it doesn’t. If DCIS does not become invasive, it will not affect how long a person will live even without treatment. Doctors don’t know which DCIS will become invasive. Nowadays, almost everyone with DCIS gets treated. Many people receive surgery, chemotherapy, or radiation who would never have gotten sick. For these people, treatment provides no physical benefit.”; no trade -offs for overdiagnosis or estimates provided  Data collection: after initial mailing of survey, reminder letters were sent at two weeks to non-responders, sent a second copy of survey after four weeks, and telephone attempt to those not responding after six weeks | | **Relative importance for decision making:**  FP: important for 38%  (Overdiagnosis: important for 60% (71% in 18-39 yr); not included in primary synthesis since no numerics provided or trade-offs elicited)  **Trade-offs:**   - 63% would tolerate 500 or more FP per life saved - 37% would tolerate 10 000 or more FP per life saved   **Subgroups:** previous FP (biopsy) similar high tolerance for FPs (35% wanted to take account of; 71% tolerate 500/life and 39% tolerate 10000 or more) | From survey “fairly realistic knowledge”: median estimate of FP was 20% for over 10 yrs and RRR for BC mortality thought 33% by 25% and 50% by half; no question on benefits in actual terms |
| Wong 2015, Hong Kong  Population-based telephone survey using random-digit dialing about print-based decision aid  Recruitment setting: Community  N=90; 54±12.4, 15.5% (minimal user charge), 0% (Chinese), NR, NR  Previous screening: few (<20% had heard of mammography)  History of FP or breast surgery: NR, NR  Opportunistic mammography screening services widely available for a minimal user charge to potential clients  ROB: High | | **Communicated Outcomes of Screening (over 10 yrs):**   - BC mortality: 20% RRR - FP: 10% - Overdiagnosis: 19% to 30% of cancers   Format & Definitions: print-based decision aid (online version; no longer functioning) with mainly textual presentation; overdiagnosis are abnormalities that may never become invasive or effect a life in any way; results in further diagnostics and treatment and no gain in post-treatment life expectancy; “major potential harm of mammography”; FPs: benign results from mammography after unnecessary investigations & may lead to anxiety, worry and depression for some; “false alarms”    Data collection: DA posted to participants after baseline survey  Other information: lower BC incidence in Hong Kong (1 in 19); age-based risk for BC; USPSTF 2009 recommendations & Hong Kong recommendation of insufficient evidence (due to lower baseline rates); possibility for earlier stage & better treatment when diagnosing early, thus may have better quality-of-life after treatment | | **Relative importance for making decisions:**  BC mortality: important for 22%  FP: important for 5%  Overdiagnosis: important 5% | At follow-up, 88% thought screening prevented BC |
| **Weighing stage distribution & treatment morbidity against FPs** | | | | | |
| Bilger 2020, Singapore  DCE with 6 attributes and randomization to with vs without a gain-framed health promotion message in a nationally representative sample of working age women (Pap smear DCEs examined but not included)  Recruitment setting: Community  N= 400; 40-64 (mean [SD]: 52.1 [7.3]); NR; 74% Chinese; NR; NR  Previous screening: 35.5%  History of FP or breast surgery: NR  Screening recommended for women aged 50 to 69 every 2 yrs, and women aged 40 to 49 with higher cancer risk to screen every year, but low uptake in part due to large out-of-pocket treatment expenses.  ROB: Low | | **Attributes and levels used (biennially, duration NR):**   - Stage distribution (BC survival among cancers): 25%, 50%, 65%, and 90% chance of surviving breast cancer if diagnosed (change from 90% to 25% stated to resemble early vs late stage diagnosis) - BC morbidity: no change, changes in feel/appearance of breast, or lose an entire breast - FP: 5%, 15%, and 30% chance of false cancer diagnosis   Format & Definition: two alternative screening scenarios per choice task stating different levels of attributes; each choice task had a forced choice and question about likelihood of screening for the preferred choice; used D-efficient design to generate 72 choice tasks, which were divided into 9 blocks of 8 choice tasks to reduce the respondent burden, then randomly assigned an equal number of respondents to each block; the health promotion message for one group was: “Cancer screening according to guidelines is a WIN-WIN. A negative test result gives peace of mind and early detection saves lives.”; DCE attributes and levels were selected based on a systematic literature review, focus group interviews, and quantitative pilots to ensure sufficient degree of tradeoff between attributes. FP: “chance of false cancer diagnosis”  Data collection: Survey administered by trained interviewers  Other information: additional attributes in DCE:   - Pain/discomfort during screening (Y/N); - Out-of-pocket payment or reward for screening (free, pay $50, pay $200, receive $50) - Total out-of-pocket treatment costs ($0, $0,000, $50,000, $150,000, $250,000 | | **Changes in stated screening uptake/acceptance according to changes in attribute levels**  Stage distribution: uptake increased by 14.5 percentage points (or 22.8% relative increase), 9.9 percentage points, and 9.3 percentage points when BC survival was changed from 25% to 90% (i.e. early to late stage), 65%, and 50%, respectively  FP: uptake increased by 1.4 percentage point (or 2.1% relative increase) when FP was reduced from 30% to 5%; by 0.6 percentage points (not significant) when FP was reduced from 30% to 15%  BC morbidity: uptake increased by 4.8 percentage points (or 7.5%) if “not losing a breast”, and by 2.1 percentage points (not significant) if “change in feel and appearance” vs no change  The incremental effects of each attribute were calculated by setting all other attributes at their reference level. Note: 63.7% uptake when all attributes were set to their worst levels (i.e., 30% chance of FP, 25% survival rate, lose an entire breast, $200 screening cost, $250K out-of-pocket treatment cost)  No significant difference in the DCE attributes’ effect between groups with vs. without health promotion message | Respondents underestimated survival for screen-detected breast and cervical cancer. 94.4% of non-screeners and 87.1% of screeners reported that they believe the screen-detected breast cancer survival rate is lower than 90% (vs data showing 93% survival for screen-detected). |
| Ganott 2006, USA [from 2018 review excluded studies list]  Prospective survey of women who were attending their routine screening mammography, about preference/trade-offs for increased recall rate for invasive and noninvasive testing versus earlier detection of breast cancer  Recruitment setting: Breast imaging centre (at mammography visit)  N=1570; 41% 40-49, 34% 50-59, 22% ≥60 yrs; NR; 97% white (2% African America); NR; 15% one family member; 2% more than one family member; 1% previous breast cancer  Previous screening: 95% (81% ≥3 times)  History of FP or breast surgery: 48% previous recall (10% invasive procedure); NR  ROB: High | | **Communicated outcomes of screening:**   - FPs vs. Stage distribution/treatment morbidity (Early detection) (current ratio): 10% recall rate to detect 1 BC in 300 women screened - FPs vs. Stage distribution/treatment morbidity (Early detection): (higher ratio): 15% recall rate to detect 1 BC in 200 women screened - FP biopsies vs Stage distribution/treatment morbidity (Early detection) (current ratio): 1 in 60 to detect 1 BC in 300 screened - FP biopsies vs Stage distribution/treatment morbidity (Early detection) (higher ratio): 1 in 40 to detect 1 BC in 200 screened   Format & Definition: prospective survey with single and multiple-choice questions; FP: “being recalled after a mammogram for additional tests (invasive or non-invasive) and the final diagnosis turn out to be “no cancer”  Data collection: surveys completed by patients prior to any imaging during their visit and were returned on the same day  Other information: inconvenience of getting screened | | **Would be willing to take the chance of being called back more often (for example 15% of the time instead of 10%) for a second non-invasive procedure (such as additional mammogram views or breast ultrasound) if doing so might increase the chance (for example 1 out of 200 instead of 1 out of 300) that if I have cancer it might be detected earlier (5-point Likert):**  Strongly agree/agree: 86%  **Would be willing to take the chance of being called back more often (for example 1 out of 40 instead of 1 out of 60) for a needle or surgical biopsy) if doing so might increase the chance that if I have cancer it might be detected earlier:**  Strongly agree/agree: 82%  **FP vs FP biopsy trade-offs** P=0.006  **Subgroups (adjustments NR):**   - <60 vs ≥60 yrs: FP and FP biopsy trade-offs: 87% vs 82% P=0.009 and 83% vs 80% P=0.15, respectively - Previously screened: FP and FP biopsy trade-offs: 86% vs 81% P=0.25 and 83% vs. 79% P=0.47, respectively - Previously recalled: FP and FP biopsy trade-offs: 89% vs 84% P=0.003 and 85% vs 90% P=0.03, respectively - Previous invasive procedure: FP and FP biopsy trade-offs: 90% and 86% willing, respectively (comparison NR) - With family history of breast cancer: FP and FP biopsy trade-offs: 88% vs 86% P=0.54 and 85% vs 82% 0.20, respectively | **Knowledge of mammography sensitivity:**  46% correctly identified the sensitivity of mammography as able to detect 80%-94% of cancers; 28% chose all or 95%  **What do you think is an average woman's approximate chance of having breast cancer detected as a result *of one screening* mammogram?**  32% chose ≥10% and 38% chose 2-8% (vs 0.2-0.8% correct) |
| Jafri 2008, USA [from 2018 review excluded studies list]  Prospective survey in an underserved and predominantly minority population of women who were going to undergo mammography, about preference for increased recall rate for invasive and noninvasive testing if it meant that breast cancer could be detected earlier  Recruitment setting: Breast imaging centre (at mammography visit)  N=911; 32% aged 40-49, 33% 50-59, 18% 60-69, 7% ≥70 yrs (53.5 [10.9]); NR; 41% White (48% Black, 10% Hispanic); NR; 14% one first-degree relative; 5% >1 first-degree relative; 7% previous breast cancer  Previous screening; 91% (70% ≥3 times)  History of FP or breast surgery: 43% history of recall; NR  ROB: High | | **Trade-off scenarios:**   - FPs vs. Stage distribution/treatment morbidity (Early detection) (“current ratio”): 10% recall rate to detect 1 BC in 300 women screened - FPs vs. Stage distribution/treatment morbidity (Early detection): (“higher ratio”): 15% recall rate to detect 1 BC in 200 women screened - FP biopsies vs. Stage distribution/treatment morbidity (Early detection) (current ratio): 1 in 60 vs. 1 in 5 with recall/1 in 300 screened - FP biopsies vs Stage distribution/treatment morbidity (Early detection) (higher ratio): 1 in 40 vs. 1 in 200 screened   Format & Definition: prospective survey with single and multiple-choice questions  Data collection: surveys completed by patients prior to any imaging during their visit and were returned on the same day; FP: “recalled for additional tests (such as additional mammogram or breast ultrasound) and the final diagnosis turns out to be ‘no cancer’”  Other information: inconvenience of getting screened | | **Would be willing to take the chance of being called back more often (for example 15% of the time instead of 10%) for a second non-invasive procedure (such as additional mammogram views or breast ultrasound) if doing so might increase the chance (for example 1 out of 200 instead of 1 out of 300) that if I have cancer it might be detected earlier:**  Strongly agree/agree: 76% White, 54% Black, 59% Hispanic; P<0.0001  Unsure: 11% White, 27% Black, 24% Hispanic  **Would take the chance of being called back more often (for example 1 out of 40 instead of 1 out of 60) for a needle or surgical biopsy, if doing so might increase the chance that if I have cancer it might be detected earlier:**  Strongly agree/agree: 75% White, 53% Black, 65% Hispanic; P<0.0001  Unsure: 13% White, 26% Black, 16% Hispanic  **Intention to continue screening after receiving a false-positive result:**  Very hesitant/somewhat hesitant to continue with screening: 4%  Very likely/Likely to continue with screening: 85%  Unsure: 5%  No response: 7%  **Subgroups:**   - 2% of White, 6% of Black, and 5% of Hispanic women were “Very hesitant/somewhat hesitant to continue with screening (P>0.05) - 93% of White, 80% of Black, and 71% of Hispanic women were “Very likely/Likely to continue with screening” (P<0.0001). When results were controlled for education level and income, these differences remained significant (P= 0.0009 and 0.016, respectively) across the three ethnicities - 2% of white, 6% of Black, and 11% of Hispanic women were “Unsure” (P>0.05) - 3% of White, 8% of Black, and 13% of Hispanic women had “No response” | **Knowledge of mammography sensitivity:**  40% of White women vs 28% of Black and 28% of Hispanic women correctly identified the sensitivity of mammography as able to detect 80%-94% of cancers (P<0.0001)  **What do you think is an average woman's approximate chance of having breast cancer detected as a result of one screening mammogram?**  34% chose ≥10% and 30% chose 2-8% (vs 0.2-0.8% correct): |

ARR, absolute risk reduction; BC, breast cancer; DA, decision aid; DCE, discrete choice experiment; DCIS, ductal carcinoma in situ; FN, false negative; FP, false positive; NR, not reported; OR, odds ratio; RCT, randomized controlled trial; ROB, risk of bias; RRR, relative risk reduction; SD, standard deviation; USPSTF, The U.S. Preventive Services Task Force; Y/N, yes/no; Yrs, years

**Table S3.4. Direct and Indirect Preferences for 40 to 49-year-olds**

| **Study, Country**  **Study description**  **Recruitment setting**  **Sample size; Age; Insured; % Caucasian (other race/ethnicities); dense breasts; other risk factors**  **Screening history**  **History of FP or breast surgery** | **Study data e.g. communicated outcomes of screening**  **Format of information and definitions provided**  **Data collection**  **Other information provided** | **Findings** | **Knowledge and understanding (including relevant subgroup findings)** |
| --- | --- | --- | --- |
| **Direct data: specific to relative importance between individual outcomes** | | | |
| **Weighing BC mortality against FPs** | | | |
| Lewis 2003, USA  RCT in clinic sample with differently framed information videos (considering as pre-post one group)  Recruitment setting: Primary care clinic  N=179; 35-49, 78%, 59% (39% Black), 0%, 15% family history; 9% at high risk (Gail model; ≥1.67% 5-year risk)  Previous screening: 75%  History of FP or breast surgery: NR, NR  ROB: Low | **Communicated Outcomes of Screening (over 10 yrs):**   - BC mortality: 1 life extended in 1000 screened every year - FP: 300 experience vs 700 do not experience in 1000 screened   Format & Definitions: 5-min videos of female MD narrator with text boxes; FP: “an abnormal mammogram when there is nothing actually wrong, but the result may require more tests or a biopsy to find out that there was no cancer; more than one third of women with a false positive continue to worry about having breast cancer”  Data collection: completed baseline questionnaires before viewing the videos; before and after seeing the videos, women responded to questions about number of lives extended, number of FP expected, and about number of women who would remain upset by FP | **Relative importance of outcomes**:  Baseline: 81% BC mortality more important (75% much more, 6% somewhat more) than FPs and worry  Follow-up: 83% BC mortality more important (75% much more; 8% somewhat more) than FPs and worry  No significant difference between framing (positive, negative, or neutral)  **Subgroups**:  No effect of previous screening or high vs. under 5-year risk of cancer  Change in perceptions of the benefits/harms did not differ women compared by their 5-year breast cancer risk as calculated by the Gail model | **Knowledge at baseline:** Most (76%) women greatly overestimated the benefit, endorsing incorrect responses that 300 women or 500 women out of 1,000 would live longer because of mammography.  Subgroups: No effect of accuracy of knowledge on findings |
| Nekhlyudov 2008, USA  Mailed survey to clinic patients before first automated contact for screening  Recruitment setting: Members of health maintenance organization  N=93; 40-44, 100%, 67% (19% Black, 11% Asian), NR, 4% family history  Previous screening: 0%  History of FP or breast surgery: **0%**, 0%  ROB: Moderate | **Communicated Outcomes of Screening (during one’s 40s):**   - BC mortality: 1 fewer in 1000 screened regularly in their 40s - FP: 10 in 100 screens   Format & Definitions: statements describing the numerical estimates; BC mortality: will live longer by having regular mammograms in their 40s; FPs: “abnormal mammogram which will lead to additional tests (sometimes referred to as call-back or false alarm) and call-backs end up not being cancer”  Data collection: surveys sent 2–4 weeks prior to screening appointment, with a reminder 1–2 weeks after initial mailing  Other information: BC risk lifetime 12 in 100, over 5 yrs <1 in 100, 95 of 100 FPs not diagnosed;  FN: 20 out of 100 | **Effect on screening intentions information on outcomes:**  BC mortality: more likely 56%, same 40%, less likely 4%  FP: more likely 29%, same 69%, less likely 2%  Baseline intentions not reported | **Knowledge at baseline:** 40 (43%) were aware of a woman’s lifetime risk of developing BC; 23% were aware of the numerical benefits of screening, 13% were aware of the chances of FN and FP mammograms, (11%) of women were aware of ductal carcinoma in situ |
| **Indirect data related to relative importance of benefits versus harms (via attitudes, intentions, and/or attendance)** | | | |
| **Weighing benefits vs harms under relatively high net benefit scenarios** | | | |
| Laza-Vásquez 2022, Spain  Single-arm proof-of-concept trial of offering personalized breast cancer screening (e.g. limiting to higher risk women if <50 yrs; higher vs lower frequency based on risk)  Recruitment setting: Primary care clinic  N= 387; 40-50 yrs (median: 48.4); NR; 79% born in Spain/Catalonia; breast density: BI-RADS A 14.7%, B 30.7%, C 44.6%, and D 10%; 9.8% family history of breast cancer; 28% increased risk (>1.16% [40-44 yrs] or >1.19% [older] 5-year risk); 3 (0.9%) high-risk (>6% at 5 yrs) referred to breast unit  Previous screening: 56.3% (opportunistic screening mainly applies to women in 40-49);  History of FP or breast surgery: NR, NR  ROB: Low | **Communicated outcomes of screening (50-69 yrs old women screened biennially until the age of 80):**   - BC mortality: 1 will survive breast cancer because of screening (4 others will die) in 200 - FP: 40 in 200 - Overdiagnosis: 2 in 200   Format & Definition: informative brochure with detailed information on the benefits and harms of screening; FP: “requiring additional test and subsequently not diagnosed with cancer”; overdiagnosis (“Some types of cancer that are detected by screening mammography grow so slowly that they would never become a health problem. Some, even, would disappear spontaneously, without treatment. Currently, one cannot know which tumors would progress and which ones would not, and therefore, treatment is offered to all women diagnosed (“treated without need”); contained numerical values and infographics; provided to women on their second visit with a healthcare professional and facilitated by professional; if no screening in past year, received mammography to obtain dense breast information  Data collection: participants were asked to return the follow-up questionnaire within 2-4 weeks of their visit with a healthcare professional  Other information: personalized 5-year breast cancer risk estimation based on Breast Cancer Surveillance Consortium (BCSC) v2.0 risk model with polygenic risk score with 83 single nucleotide polymorphisms; 15 of 200 diagnosed with breast cancer, the treatments of some cancers can be less aggressive with screening; FN: much less frequent (than FPs) and occurs when the mammogram does not show any signs of the disease, even if the woman suffers it | **Attitudes towards personalized breast screening (3 5-point items):**  Median score (range: 3-15, higher score indicates more positive attitude): 12 (IQR: 11; 14)  Positive attitude (score ≥12): 62.7% (95% CI: 57.2% to 67.9%)  Were more favorable towards increasing frequency for higher risk vs. reduction for lower risk.  **Attitudes towards undergoing breast screening (without specifying personalized screening) (5 5-point scale):**  Median (range: 5-25, higher scores indicate more positive attitude): 22 (IQR: 21; 25)  Positive attitudes (scores ≥ 20): 87.8%  **Screening intention for personalized screening (5-point Likert):**  Intending to participate (definitely/likely): 91.7%  **Preference with regard to the current screening (biennial exams between 50 and 69 yrs):**  Would choose personalized screening: 66%  Would choose the current screening: 26.7%  Don’t know: 7.3%  **Relative importance of considering chances of each outcome for decision making**:  BC mortality: 84.5% very important/9.9% important  Overdiagnosis: 49.5% very important/28.1% important  FPs: 51.6% very important/25.8% important | **Knowledge:**  Adequate knowledge (combined conceptual and numerical knowledge across all subscales of BC mortality, FPs, and overdiagnosis): 1.5%  Adequate knowledge about overdiagnosis: 8.3% |
| Roberto 2020, Italy  RCT of online decision aid vs online standard brochure in women aged >45 at first invitation  Recruitment setting: organized screening program lists (for first invitation; 2 of 6 centres invite at 45 yrs)  N= 1,001; >45 (mean [SD]: 49.7 [3.2]); NR; 96% Italian; NR; NR  Previous screening: 68%  History of FP or breast surgery: NR, NR  ROB: High  **Also included in Table for 50-59 yrs** | **Communicated Outcomes of Screening**  **Decision aid (regular screening over 30 yrs):**   - BC mortality: 12 vs 20 in 1000 women - FP: 45 in 1000 screens (at each screen) (about 30% requiring biopsy); - Interval cancers (“FNs” but described as interval cancers): 1 to 2 in 1000 screened - Overdiagnosis: 4 vs. 0 in 1000   **Control program brochure (regular screening over 20 yrs):**   - BC Mortality:7-9 lives are saved per 1000 screened - FP: 4 of 100 screens - Overdiagnosis (of DCIS): 1 in 10 cancers detected   Format & Definition: online decision aid with text, images and diagrams (icon arrays of screened and not screened)  <https://www.donnainformata-mammografia.it/en/>. FP: “A doubtful result or “positive mammography” means the radiologist has seen images like nodules, masses, calcifications or others that could signal a tumor and thus call for more investigation. This may cause you anxiety and concern but it does not imply a malignant disease. If the suspicion is not confirmed by further tests it is called a ‘false positive’”; overdiagnosis: “Sometimes there is unnecessary and useless treatment (overtreatment), and if it is useless it is harmful, of tumors discovered by the screening that would resolve themselves spontaneously during the woman’s life. These malignancies look just like other tumors but either do not grow or grow very slowly. Unfortunately, so far there is no way to distinguish whether a tumor is actually not harmful, so all cases are treated and the woman will not know what her situation was”; Control group brochure had absolute effects in textual format for FPs (“need to undergo repeat exam but it turns out to be a “false alarm” as the in-depth analyses exclude the presence of a tumor”), overdiagnosis (“some anomalies, among those detected by mammography, are not destined to become invasive tumors and to compromise the woman's health (the so-called overdiagnosis)”, and morality with screening (“for every 1000 women aged between 50 and 69 yrs who regularly perform mammography, 7-9 lives are saved within 20 yrs”)  Data collection: 7-10 days after receiving information  Other information:   - True positives: 5 in 1000 women - True negative: 950 in 1000 women - Other estimates of BC mortality eg data about controversies: ~20% in women aged 40-74 yrs over 13 yrs (Cochrane review and the Independent UK Panel review); 25% reduction in women aged 50-69 (Euroscreen review) - Risks related to radiation (comparable to that absorbed in a few weeks from “background radiation”) - Other estimates of overdiagnosis: 4 in 1000 women aged 50 yrs over 30 yrs (Euroscreen review) vs others with 19-30% of cancers | **Positive intention toward screening (5-point Likert scale; NR how determine “positive”)**:  Intervention: 99%  Control: 98%  (P= 0.0230)  **Positive attitude toward screening (6 5-point scale questions but NR how determine “positive”)**:  Intervention: 92%  Control: 92%  (P= 0.0922)  **Screening attendance**:  Intervention: 84%  Control: 83%  (P = 0.6537) | **Adequate knowledge (>8/13 correct answers):**  Intervention:  Baseline: 23%; Follow-up: 44%  Control:  Baseline: 18%; Follow-up: 37 |
| Schonberg 2020a, USA  Pre-post trial of a 2-page personalized breast cancer risk report for women in 40s and their primary care providers  Recruitment setting: Primary care clinic  N=306; 40-49 (44.1 [2.9]); NR; 64% White (17% non-Hispanic Black, 7-8% each Hispanic and Asian); NR; 32% ≥1.1% & 3.9% ≥1.7% 5-year risk & 17% ≥20% lifetime risk  Previous screening: 71% (mammogram, may not have been screening)  History of FP or breast surgery: NR, 0% (excluded)  ROB: High | **Communicated Outcomes of Screening (in their 40s)**   - BC mortality: Very few women in their 40s die of breast cancer (16 to 20% of women in their 40s diagnosed with breast cancer die of breast cancer); having mammograms lowers the chance of dying from breast cancer by 15%. - FP: 50% of women in their 40s who have mammograms experience a false alarm - Overdiagnosis: 1 in 5 BC found on a mammogram   Format & Definition: report on personal risk and bullets about benefits and harms; FP: “a false alarm that may trigger additional testing (e.g., breast ultrasounds, breast MRIs, or even breast biopsies)”; overdiagnosis “cancer would not otherwise have caused problems in a woman’s lifetime (overdetection). Since doctors do not know which breast cancers will grow and which will not, all women are treated for breast cancer. Breast cancer treatment may include surgery, radiation, chemotherapy, and/or medications”; referred to online decision aid for women in their 40s with text and images (icon arrays) and references to risk calculators and guidelines; for those at increased five-year risk (≥3% for postmenopausal and ≥1.7% for premenopausal; Breast Cancer Risk Assessment Tool (BCRAT) and Breast Cancer Surveillance Consortium (BCSC) model (when breast density was available) statement that mammograms are recommended (not a personal choice) and to consider breast cancer prevention medications) and mentioned supplemental screening with MRI for those with lifetime risk >25%  Data collection: pre-visit questionnaire and risk factors completed median 19 (IQR=8-36) days before visit with primary care provider; providers emailed risk report and DA and patients received a copy by email; post-visit questionnaires completed (telephone or email) median 9 (IQR=1-23) days after visit  Other information: mammograms may find breast cancers when they are smaller and may be easier to treat; chance of BC diagnosis based on each participant’s risk factors and age; breast MRI recommendations, gene mutation testing recommendations; details on risk calculators used to estimate 5-year risk (Breast Cancer Risk Assessment Tool [BCRAT] <https://www.cancer.gov/bcrisktool/>); United States Preventive Services Task Force mammography screening guidelines | **Intentions to be screened (mean ± SD)**  Scale from 0 (will not have a mammogram in the next year) to 100 (will have a mammogram in the next year)  Overall: pretest 79±28; posttest 68±40 (RR [95% CI]: -10.3 [-14.3, -6.3]; P=<0.0001)  Subgroup:  5-year risk <1.1%: pretest 77±29; posttest 63±41 (RR [95% CI]: -13.4 [‒18.4, -8.5]; **P=<0.0001**)  5-year risk ≥1.1%: pretest 83±25; posttest: 77±37 (RR [95% CI]: -4.3 [-11.2, 2.6]; P=0.22)  5-year risk < 1.7%: pretest 79.1±27.7; posttest 67.2±40.2 (RR [95% CI]: ‒10.9 [‒15.0, ‒6.8]; **P<0.0001**)  5-year risk ≥ 1.7%: pretest 82.5±24.2; posttest 86.7±29.5 (RR [95% CI]: 4.17 [‒15.4, 23.7]; P=0.67)  **Underwent screening (using claims data supplemented by chart review)**:  Overall: pretest (in 2 yrs prior) 38%; posttest (16.1 months [SD=5.4] mean follow-up) 42%, P=0.17  Subgroup:  5-year risk <1.1%: pretest 31%; posttest: 32% P=0.70  5-year risk ≥1.1%: pretest 52%; posttest: 62% P=0.10  5-year risk < 1.7%: pretest 36%; posttest 39% P=0.31)  5-year risk ≥ 1.7%: pretest 50%; posttest 57% P=0.55  There was no significant effect modification by patients’ race/ethnicity or educational attainment. | **Knowledge (mean ± SD):**  8 True/False conceptual questions about the benefits and risks of screening mammography  Overall: pretest 5.8±1.3; posttest 6.2±1.2 (P<0.0001) |
| Seitz 2016, USA  RCT using online survey (by survey company), stratified into 2 groups based on Gail Assessment (<1.5% vs ≥1.5% 10-year risk) then each risk group randomized to 1 of 8 conditions varying according to content (brief [narrative] vs extended [numerical]) and format (expository vs untailored exemplar vs tailored exemplar), vs comparators no or basic (statements of USPSTF and American Cancer Society recommendations) information.  We report results for brief and extended versions vs. comparators for women with <1.5% risk for BC (format did not change results)  Recruitment setting: Community  N=2918; 35-49, 81.3%, 69% (11.5% African American); 75.4%, NR, 2 risk groups of <1.5% (n=1227) vs ≥1.5% (n=1691 [excluding those with previous BC and BRCA mutation]) 10-year risk (Gail Assessment)  Previous screening: 40% (low risk group), 74.1% (elevated risk group)  History of FP or breast surgery: NR, NR  ROB: High | **Brief intervention**: Individual risk for cancer and table summarizing USPSTF and ACS recommendations  **Extended intervention:**  **Communicated Outcomes of Screening (over 10 yrs, biennially between the ages of 40 and 50):**   - BC mortality: 3 in 1000 (without mammogram) - BC mortality: 2 in 1000 (with mammogram) - FP (tests, biopsies, or surgery): 239 in 1000 women - Overdiagnosis: In addition to finding breast cancer, mammograms can sometimes draw attention to suspicious cells that would never had spread or become life-threatening. Doctors cannot always tell if these will spread or not, which may lead to unnecessary treatment.   **Communicated Outcomes of Screening (over 10 yrs, biennially between the ages of 50 and 60):**   - BC mortality: 6 in 1000 (without mammogram) - BC mortality: 4 in 1000 (with mammogram) - FP: 220 in 1000 women - Interval cancers: 9 (40-50 yrs) or 10 (50-60) in 1000 women have BC that is not detected by a mammogram, because it develops between mammograms   Format & Definitions: Online decision aid with text and diagrams, participants were emailed a link to the experiment; FPs: “women do not have BC, but have extra tests, biopsies, or surgery following abnormal mammograms... abnormal results that look like cancer but are not that can lead to unnecessary biopsies, and painful surgeries”  Data collection: participants completed measures and were then randomly assigned to one of the conditions and completed additional measures  Other information: personal and general 1-year and lifetime BC risks. TP and FNs: 12 (40-50 yrs) or 23 (50-60) in 1000 have BC detected by a mammogram; statement on radiation. Women under 50 have a decision to make.  All numerical data obtained from author contact. | **Intentions to wait until age 50; Odds ratio vs no/basic information) (low risk group):**  Comparators: 7.2% and 6.7%  Brief interventions: 14-18.4% (ORs 2.09-2.89)  Extended interventions: 19.4-24.2% (ORs 3.07-4.08)  ORs vs comparator all significant for increased intentions to wait; difference not significant between brief and extended interventions  **Intentions to start or continue to have mammograms in their 40s (elevated risk group)**  Comparators: 72.6% and 73.0%  Brief interventions: 70.5-77.9% (ORs 1.13-1.64)  Extended interventions: 68.7-75.9% (ORs 0.71-1.32)  ORs vs comparator - only one intervention significant for increasing intentions to screen in their 40s; difference not significant between brief and extended. | All women overestimated their risk for breast cancer by approximately 10-15% (e.g. objective risk via Gail at 1-2.5% vs perceived by women before (17-20%) intervention.  There were no significant moderation effects of numeracy for mammography intentions. |
| Driedger 2017, Canada (Toronto and Winnipeg)  Focus groups (n=5) in 2012 with members of the public using presentations and print hand-outs; survey research company recruitment using known survey participants and random-digit dialing, with maximum variation sampling (e.g. wide variation in income and marital status but education skewed to higher) based on questionnaire. 4 of 5 focus groups stratified by age (35-49 and 45-59)  Recruitment setting: Community  N=46; 35-59 (63% <50); NR, NR, NR, NR  Previous screening: 61% (100% in >50 yrs; most <45 had not)  History of FP or breast surgery: NR, NR  **Also included in Table for 50-59 yrs**  ROB: High | **Communicated Outcomes of Screening (total of 11 yrs):**   - BC mortality:   40-49: 2108 would need to be screened to prevent 1 death  50-59: 721 would need to be screened to prevent 1 death   - FP:   40-49: 690 in 2108  50-59: 204 in 721   - Unnecessary biopsies:   40-49: 75 in 2108  50-59: 26 in 721  Format & Definitions: Presentation and print hand-outs of plain language descriptions of 2011 CTFPHC guidelines (2180- and 721-person diagrams for BC mortality, FPs, and unnecessary biopsies at 40-49 and 50-69), a summary of the relevant research evidence, and a description of the uncertainties that remained. “Some doctors think that finding a cancer through screening in this age group (40-49) – who are usually pre-menopausal, won’t change the available treatment options or the effectiveness of treatments and might expose women to unnecessary risk (including additional testing and anxiety)”  Data collection: focus groups were accompanied by hand-outs given to each participant; followed by being asked at what age they thought screening should begin  Other information: None | **Age to start screening:**  At age 40: 35-49; 21%  50-59; 35%  At age 50: 35-49; 41%  50-59; 29%  Unclear: 35-49; 38%  50-59; 35%  Note: unclear was based on authors’ interpretations because using focus group data to quantify  **Relative importance of outcomes:**  13% (most under 50) felt the potential harms of screening were too high a price to prevent one 40 -49 year old from dying |  |
| Elkin 2017, USA  Prospective, single-arm trial for development and evaluation of a web-based decision aid to help women decide when to start and how often to have mammograms  Recruitment setting: Primary care clinics  N=168; 40-49; 98%; 80% (Asian 12%, Hispanic 5%); NR, 20% family history; 0% at high risk  Previous screening: 74% at least once  History of FP or breast surgery: NR, NR  ROB: High | **Communicated Outcomes of Screening**  **Comparing BC mortality, over one’s lifetime, based on starting age and intervals (deaths averted from cited modelling paper):**   - Annually starting at 40: BC mortality: 22 vs 30 in 1000 - Biennially starting at age 40: BC mortality: 24 vs 30 in 1000 - Annually starting at age 50: BC mortality: 23 vs 30 in 1000; 977 in 1000 die of other causes - Biennially starting at age 50: BC mortality: 25 vs 30 in 1000; 975 in 1000 die of other causes - **Over 10 yrs**, annual screening cumulative FP 60%, biennial screening FP 40% - **In 1000 screens**, FP 98 vs TP 2   Format & Definition: online decision aid with text and icon arrays framed around decisions on when to start screening (40s or 50s) and how often. FPs: “do not have BC despite an abnormal mammogram; may require biopsy and be inconvenient and physically uncomfortable”. Overdiagnosis and unnecessary treatment are described qualitatively in plain language, with links to additional information. Also, “many cancers found by screening have a very small chance of causing death”.  Data collection: participants completed breast cancer risk assessment, presented with DA on subsequent screens, then asked to consider a series of 10 statements and indicate their level of agreement or disagreement  Other information: Personal risk assessment and prediction over next 5 yrs provided. FN: 1 per 1000 “BC that is missed by screening”; values clarification exercise; radiation risk “extremely low” | **Actual & intention to start or continue screening (one-month follow-up):**  30% Had a screening mammogram  18% Scheduled an appointment for screening mammogram  29% Plan to make an appointment *in the next 6 mos*.  18% No plan  5% Unsure  **Potential benefits outweigh the potential risks:**  83% strongly agree or agree  10% Neither agree or disagree  7% Strongly disagree or disagree  **Subgroups:**  There was no association between a user’s predicted breast cancer risk, based on the Gail model and given to her in BSD, and actual or intended use of screening. |  |
| **Weighing benefits vs harms under relatively moderate net benefit scenario** | | | |
| Valentine 2022, USA  RCT of a 4-stage intervention: (1) didactic information with benefit/harms; (2) document on possible consequences of overdetection; (3) Aiding Risk Information learning through Simulated Experience (ARISE) consisting of 30 grids with 100 colored squares representing a possible result of cancer screening; (4) narrative of screening experience based on symptom lists and narratives found in literature, versus a hypothetical screening test with no proven physical benefits (study also examined preferences related to prostate cancer screening in men)  Recruitment setting: Public via survey platform  N=2120; 40-70 (49.5 [7.8]); NR; 84% White (10% African American & 3% Hispanic); NR; 0% first degree relative with breast cancer  Previous screening: 72%  History of FP or breast surgery: NR; NR  ROB: Low  **Also included in Table for 50-59 yrs** | **Communicated Outcomes of Screening (≥ 40 yrs over 11 yrs)**   - - BC mortality: 7 in 1000 (with mammogram) (no benefit group)   - BC mortality: 5 in 1000 (with mammogram) (benefit group)   - BC mortality: 7 in 1000 (without mammogram)   - FP: 160 in 1000 - Overdiagnosis: 20 in 1000 (data taken from survey text and images in appendix; which differs from manuscript text for BC mortality (1 fewer per 1000)   Format & Definition: didactic information had text description and icon arrays of a hypothetical test using numeric about BC mortality, FP: “the test says that you probably have cancer but you actually don’t”, and overdetection: “test finds a cancer that is not dangerous, and you are diagnosed and treated for a cancer that would not have caused any trouble; They are diagnosed with cancer and get surgery to remove their cancer or radiation therapy to shrink the tumor. However, this treatment is unnecessary because the cancer would have never grown or spread or caused problems”. ARISE: Section with 30 grids consisting of 100 colored squares (black=death from cancer, yellow=unnecessary biopsy, red=unnecessary treatment, blue=lives saved, and grey=no benefits/harms) each and were informed that each colored square represented the result of one patient’s cancer screening test (3000 possible outcomes viewed); narrative: physical and emotional of a FP biopsy  Data collection: online; randomized to benefit vs no benefit scenarios then each presented with 4 informational interventions; (1) didactic information with explicit recommendation (in benefit scenario: recommending individual decision making for women 40–49 y old and screening every other year for women 50–74); then responded to 3 knowledge questions; (2) descriptive harms information; then recorded preferences; (3) ARISE then recorded preferences (4) narrative experience; then recorded preferences and responded to 3 knowledge questions. Each participant was randomized to either ARISE followed by narrative OR narrative followed by ARISE.  Other information: descriptions of physical, emotional and financial harms from unnecessary treatment; definitions of TPs, FNs, beneficial test scenario included US Preventive Services Task Force recommendations that benefits and harms closely balanced and between ages of 50 and 74 screening should be done every other year | **Preference for screening**  At this point would you want to get the screening test (yes/no)? baseline and after each of the 4 interventions  Baseline: 84.9% yes  **<50 vs ≥50 yrs**: 88.5% vs. 80.4%; P < 0.001  After 1st and 4^th^ stages (respectively), choice of screening in arm receiving information on beneficial test: 53% and 28-30% (from figure) vs. arm receiving information on unbeneficial test: 30% to 20% (from figure)  As each stage was completed women were less likely to prefer screening test; odds ratios compared with first intervention (95% CI)  2^nd^ stage = OR 0.29 (0.18 to 0.46)  3^rd^ stage = OR 0.12 (0.07 to 0.20)  4^th^ stage -= OR 0.10 (0.06 to 0.17)  Women were more likely to prefer the beneficial screening test compared with the unbeneficial test (OR = 5.69, 95% CI = 2.58 to 12.54, P < 0.001)  Subgroups: preference for beneficial vs unbeneficial test by age P=0.693 | 3 questions on number of lives saved, FPs, and overdiagnosis per 1000 after didactic intervention and all interventions  Recall accuracy increased from the first to the last intervention for women in the unbeneficial test condition (OR = 1.95, 95% CI = 1.64, 2.32, P < 0.001) but not for women in the beneficial test condition (P = 0.379)  No change in knowledge of the FP rates or rates of overdetection (for both groups) |
| **Weighing benefits vs harms under relative low net benefit scenarios** | | | |
| Saver 2017, USA  Randomized crossover study with a video intervention of slides used using a physician-patient interaction and numerical data and textual descriptions compared with a paper-based patient brochure without any data  Recruitment setting: Community, primary care clinics, public healthcare system (broad approach)  N=35; 40-49, NR, 60% (34% Hispanic), NR, NR  Previous screening: 91%  History of FP or breast surgery: 37%, NR  ROB: Moderate | **Communicated Outcomes of Screening (over 10 yrs, biennially between the ages of 40 and 50):**   - BC mortality: 4 in 1000 (without mammogram) - BC mortality: 3.5 in 1000 (with mammogram) - FP: 330 in 1000 women (36 biopsies) - Overdiagnosis: between 2 and 10 of 19 diagnoses in 1000 - Also mentions 1 in 200 women getting mammograms *over their lifetime* will be saved from dying from BC   Format & Definitions: Recorded vignettes of physician-patient discussion about mammography based on 2009 USPSTF recommendations. [https://youtu.be/6uGy72OCv_Q](https://youtu.be/6uGy72OCv_Q%20) FPs: ‘mammograms are not perfect (abnormal mammogram leads to extra tests like ultrasound and biopsies when there is no cancer). Waiting for results can lead to unnecessary stress.” Overdiagnosis: “Sometimes what looks like cancer under the microscope doesn’t grow or spread like cancer. So some women go through the stress, possibly surgery and sometimes radiation and chemotherapy BUT they get treatment for something that never would have hurt them.”  Data collection: participants answered questions in baseline survey and their opinion on the balance of benefits and harms of the screening test, followed by viewing intervention, and asked again to complete questions about their screening intentions  Other information: USPSTF recommends every other year and says if done every year, FEW additional lives saved BUT harms almost doubled. Other groups say mammograms should be done every year. Sometimes a biopsy shows something that isn’t cancer but has a chance it could later turn into cancer. This is called DCIS. | **Desired frequency of screening:**  Pre: 54% annually, 34% biennially  Post: 14% annually, 60% biennially  **Intend to screen:**  Pre: 85% yes and 6% unsure  Post: 49% yes, 20% unsure  Mean change in wanting mammography on 3-point scale (2=yes, 1=unsure, 0=no)  -0.06 (p=0.75) control  -0.50 (p <0.001) intervention  **Benefits>Harms (1=definitely yes, 5=definitely no):**  -0.14 (0.38) control  -0.65 (<0.001) intervention  Order of presentation did not make a difference. | Authors report that harms being unclear (anxiety from FP) or poorly understood (overdiagnosis and overtreatment) may be reason for small changes to intentions; the men in the study evaluating prostate cancer were much more likely to reduce intentions (perhaps due to easily understood harms). Also report that their qualitative work when developing the tool indicated that women are heavily socialized to value mammography. |
| Mathieu 2010, Australia  RCT of immediate vs delayed access to online decision aid for 40s  Recruitment setting: Community  N=412; 38-45 (16% <40); 100%; NR, 10% mother/sister  Previous screening**:** 27%  History of FP or breast surgery: NR, NR  ROB: Moderate | **Communicated Outcomes of Screening (biennially over 10 yrs):**   - BC mortality: 2 vs 2.5 in 1000 - All-cause mortality: 12.8 vs 13.3 in 1000 die from any cause (including BC) - FP: 239 vs 0 extra tests in 1000 - Overdiagnosis: 21 vs 14 BC diagnoses in 1000; some will never affect your health   Format & Definition: online decision aid with text and diagrams (icon arrays for screened and not screened) <http://www.mammogram.med.usyd.edu.au/>  FPs: “extra tests after an abnormal mammogram. The extra tests will show these women don't have breast cancer. Aside from the inconvenience of attending for these tests, some women will worry long after they have had them”; also in pop-up window that women in the 40s have denser breasts and more recalls than those in 50s; Overdiagnosis (link in text to pop-up window when numbers provided for extra cancers diagnosed with screening): “some extra diagnoses will lead to less death, some will just be known longer and some would never have effected your health; slow growing such as DCIS (a non-invasive form) that get treated; and not possible to predict which ones will become invasive”  Data collection: participants provided with baseline questions online, were immediately randomized and had access to DA, and then asked to complete outcome questions  Other information: 7 extra women diagnosed; 9 FNs; 740 TN (reassurance) vs without screening 986 will not get BC or be screened. Information compared screening in 50s (i.e., in 1000 over 10 yrs: FN 10.4, FP 209; BC deaths saved 2; DCIS 4.9 vs 28 invasive); background information and reason for a decision in 40s; values clarification exercise (personal worksheet) | **Intentions to start screening:**  Intervention: 43% yes, 18% undecided, 39% no  Control: 40% yes, 39% undecided, 21% no  **Relative importance/value for decision making**:  For women’s values related to the benefits and harms (Dormandy scale; closer to 6 more positive values): No difference between groups or between benefits (4.2 vs 4.1) and harms (4.5 vs 4.3). | Knowledge of benefits and risks (4 numerical and 5 concept questions; ≥6 out of 10 adequate knowledge:  Mean: 7.35; 94% adequate knowledge |
| Paul 2008, New Zealand  Deliberative jury using random sample from electoral poll for public funding of screening women in their 40s; 1.5 day with expert testimonies (conflicting and neutral) and decision aid provided, discussions and deliberations  Recruitment setting: Community  N=12; 40-49; 100% for ≥45 (55% in study had <45 when not funded); 64% European (15% Maori, 15% Asian/Pacific Islander), NR, 36% family history of BC  Previous screening**:** 55% (before 45)  History of FP or breast surgery: NR, NR  ROB: Moderate | **Communicated Outcomes of Screening (over 10 yrs biennially):**   - BC mortality: 2 vs 2.5 in 1000 - All-cause mortality: 12.8 vs 13.3 in 1000 die from any cause (including BC) - FP: 239 vs 0 extra tests in 1000 - Overdiagnosis: 21 vs 14 BC diagnoses in 1000; some will never affect your health   Results indicate there was discussion that lack of evidence that screening in this age group really does save lives  Format & Definition: presentations, decision aid with text and diagrams <http://www.mammogram.med.usyd.edu.au/> , deliberations; FPs: “extra tests after an abnormal mammogram. The extra tests will show these women don't have breast cancer. Aside from the inconvenience of attending for these tests, some women will worry long after they have had them”; also in pop-up window that women in the 40s have denser breasts and more recalls than those in 50s; overdiagnosis (link in text to pop-up window when numbers provided for extra cancers diagnosed with screening): some extra diagnoses will lead to less death, some will just be known longer and some would never have effected your health; slow growing such as DCIS (a non-invasive form) that get treated; and not possible to predict which ones will become invasive  Data collection: during first session given copies of standard criteria for assessing screening programs; at second session presentation given by experts and DA was viewed; deliberations and conclusion reached on final day  Other information: 7 extra women diagnosed; 9 FNs; 740 TN (reassurance) vs without screening 986 will not get BC or be screened. Information compared screening in 50s (i.e., in 1000 over 10 yrs: FN 10.4, FP 209; BC deaths saved 2; DCIS 4.9 vs 28 invasive) | **Voting about public provision of screening 40-49 yrs:**  10 of 11 women changed their mind from *for* to *against* public provision of screening for 40-49, but the current policy of screening from age 45 should not be changed (back to age 50)  The majority felt that mammography is not an accurate enough test for women 40 – 44 (FNs, FPs) and that lack of evidence that screening in this age group really does save lives, compared to starting screening in the 50’s. |  |

BC, breast cancer; DA, decision aid; DCE, discrete choice experiment; DCIS, ductal carcinoma in situ; FN, false negative; FP, false positive; IQR, interquartile range; NR, not reported; OR, odds ratio; RCT, randomized controlled trial; ROB, risk of bias; RR, relative risk; RRR, relative risk reduction; SD, standard deviation; TN, true negative; TP, true positive; USPSTF, The U.S. Preventive Services Task Force; Vs, versus; Y/N, yes/no; Yrs, years

**Table S3.5. Direct and Indirect Preferences for 50 to 69-year-olds**

| **Study, Country**  **Study description**  **Recruitment setting**  **Sample size; Age; Insured; White (other race/ethnicities); dense breasts; other risk factors**  **Screening history**  **History of FP or breast surgery** | **Study data e.g. communicated outcomes of screening**  **Format of information and definitions provided**  **Data collection**  **Other information provided** | **Findings** | **Knowledge and understanding (including relevant subgroup findings)** |
| --- | --- | --- | --- |
| **Direct data: specific to relative importance between individual outcomes** | | | |
| **Weighing BC mortality against FPs and/or overdiagnosis** | | | |
| Hersch 2015, Australia  RCT drawn from random cohort via electoral register comparing two decision aids +/- data on overdiagnosis  Recruitment setting: Community  N =879; 48-50; 100%, NR use of electoral register, NR; 4% (one close blood relative diagnosed ≥50 yrs)  Previous screening: NR (not past 2 yrs although 40-49 are eligible without invites in AUS)  History of FP or breast surgery: NR, NR  ROB: Low  **Also included in indirect data below (different findings)** | **Communicated Outcomes of Screening (over 20 yrs):**   - BC mortality: 4 avoid dying but 8 still die in 1000 - FP: 412 in 1000 (67 have biopsy) - Overdiagnosis: 19 of 73 (26%) diagnosed in 1000 (Intervention group only)   Format & Definitions: booklet with text and visual formats using icon arrays and schematic with screening vs no screening for overdiagnosis; overdiagnosis: “Screening leads to finding some breast cancers that are not harmful; cancers like this may grow very slowly or just stay the same. Without screening, they would never be noticed or cause any trouble. Further checks and examination, doctors cannot be sure which cancers will be harmless. Therefore, treatment is recommended. So, across all the women who have screening, some end up having treatment they do not need.” Schematic of women screened vs not screened. Details of treatments and their risks. Differences between FPs and overdiagnosis. FPs: false alarms with extra tests;” women often feel anxious while they are having the extra tests and waiting for their results, and then feel relieved when they are told there is no cancer after all. However, some women find that they keep worrying about breast cancer for a while afterwards”. Materials were posted for women to read at home, consistent with the setup of the Australian breast screening program, which is directly accessible by women without referral. No provider consultation, training or counseling was incorporated; https://ses.library.usyd.edu.au/handle/2123/16658  Data collection 1-4 wks after receiving decision aid via post about 1/3 had discussed with partner or friend but not GP; extended follow-up (Hersch 2021) at 1 and 2-yrs  Other information: 54 in 1000 women are diagnosed with BCs that is not over-detection; decision aids had additional information but same for both groups; all participants also received national screening brochure without numerical data; common chemotherapy side effects include nausea and vomiting, tiredness, hair loss, and diarrhea or constipation; common side effects of radiation include tiredness, and the skin of the breast becoming dry and red or darker in color | **Relative importance of outcomes (very important) (at ≤1 month follow-up):**  BC mortality: Intervention 67%, Control 79%  FP: Intervention 41%, Control 52%  Overdiagnosis: Intervention 45%, Control 57%  p<0.01 for all  **Women’s values related to benefits and harms (at ≤1 month follow-up):**  Lower value in Intervention vs. Control for benefits (4.0 vs 4.3) and harms (4.1 vs 4.3). (closer to 6 more positive values; both p<0.01) | Overall knowledge, mean (based on all conceptual and numerical knowledge items about BC mortality benefit, overdiagnosis, and FP; maximum 22 points):  IG: 13.5  CG: 11.8 |
| Waller 2014, UK  Home-based, computer-assisted survey with data presented in three different forms using stratified random location sampling (considered pre-post study)  Recruitment setting: Community  N=954; 53-70 (mean 62) yrs; 100%; NR, NR  (Additional sample not yet eligible for screening [25-46 yr] not used)  Previous screening: 91%  History of FP or breast surgery: NR, NR  ROB: Low | **Communicated Outcomes of Screening:**   - Version 1: 1 life saved to 3 overdiagnosis (ratio) - Version 2: Total number of overdiagnoses 4000 compared to lives saved 1300 - Version 3: For every 200 women screened for 20 yrs there are 3 overdiagnoses and 1 lives saved   Format & Definition: narrative during survey questions; overdiagnosis: “some women who have a screening mammography will be diagnosed and treated for breast cancer that would never otherwise have been found and would not have become life-threatening. This is the main risk of breast screening.” [If “yes” for needing more information] “The main risk of breast screening is that some women end up having treatment for a cancer that would not have caused them any harm (i.e. they would have died from something else). This is because we can't tell which breast cancers will be harmful and which ones won't, so all women are offered treatment.”  Data collection: during computer assisted survey participants received one of three versions, with comprehension assessed before and after information exposure | **Intentions to screen** (probably/definitely)**:**  baseline 91.4% vs follow-up 92%  **Decreased intentions** (1 level change between 5 yes definitely, yes probably, not sure, probably not, definitely not):  4.5%  No significant difference for intentions between versions of data (p=0.45; including data for sample not yet eligible for screening) | 52.5% correct for objective question on overdiagnosis understanding and 67.6% reported no subjective uncertainty in understanding, but 47.5% failed to understand that screening increased cancer diagnosis (worse understanding than with studies having more explanation, e.g. Hersch 2013) |
| Gyrd-Hansen 2000, Denmark  Discrete ranking/choice study with conjoint analysis with random sample drawn from register  Recruitment setting: Community  N=207; 50; 100%; NR drawn from national registry, NR, NR  Previous screening: NR but starting age for screening  History of FP or breast surgery: NR, NR  ROB: Low | Variables on benefits and harms to calculate preference/ utility weights:   - BC mortality: Risk of dying of BC over lifetime (30 yrs) 340 per 10,000 without screening vs. 0, 60, 90,120,130 with screening - FP: Risk for FP over lifetime 0 per 10,000 without screening vs. to 1,200, 2,300, 3,500, 4,700 with screening   Format & Definitions: personal interviews with choice cards; FPs: “being called for unnecessary clinical mammography”  Data collection: participants were presented with four options / alternatives and asked to rank and qualify choices by checking off a list of possible motivations; and were asked questions about income and education | **Preference weights/coefficients (both significant):**  BC mortality reduction 0.01642  FP over lifetime -0.000297  Both significant, i.e., both increase in utility/disutility as program gets more intensified in intervals or # total screens  **Intentions to screen**: 12.9% declined to participate in a BC screening program (in all scenarios)  **Subgroups:** number of screening tests over a lifetime had no impact on preferences (i.e., minor inconvenience); no professional training increased the utility for BC mortality reduction (e.g., lower education may increase judgement biases from media and overestimation of small probabilities) | Those (25%) with wrong answers to 4 questions on understanding or who did not rank consistently were excluded from study. |
| Yasunaga 2007, Japan  Contingent valuation study; WTP (with or without harms data) drawn from random sample of registered internet users with outcomes in two information sheets  Recruitment setting: Community  N=397; 50-59, NR, 100% Japanese, NR, NR  Previous screening: 38%  History of FP or breast surgery: NR, NR  ROB: High | **Communicated Outcomes of Screening:**   - Sheet A: BC mortality: 20% RRR - Sheet B: BC mortality: 20% RRR & FP imaging and biopsies (most fine-needle): 803 in 10000 - Cost options were $: 5, 10, 30, or 50, then 2, 5, 10, 30, 50, 70 (higher or lower based on first bid)   Format & Definitions: online survey with information sheets with lists of objective facts; FPs: additional close examinations with imaging *and* a biopsy, even if they don’t have BC; tests with details on invasive test procedures but no risks or psychosocial consequences mentioned  Data collection: after random assignment participants were presented with either sheet A or sheet B  Other information: detection rate 22 in 10,000, increasing BC and BC deaths in Japan, screening procedure, detection rates | **Willingness to pay for screening:**  Sheet A $16.82 (95% CI 14.21-19.42)  Sheet B $12.89 (95% CI 10.99-14.79)  p = 0.02  **Subgroups**: higher WTP if family history of BC; lower WTP in those without previous screening history |  |
| **Indirect data related to relative importance of benefits versus harms (via attitudes, intentions, and/or attendance)** | | | |
| **Weighing benefits vs harms under relatively high net-benefit scenarios** | | | |
| **Screening initiation/focus on 50 year-olds** | | | |
| Berens 2015, Germany  Survey questionnaire 1 mo after receiving population-based program invite plus 2010-2015 version of German leaflet  Recruitment setting: Organized screening lists  N=4113; 50; 100%; 90% (10% immigrants); NR, NR  Previous screening**:** 0%  History of FP or breast surgery: NR, NR  ROB: Low | **Communicated Outcomes of Screening (over 20 yrs, biennially):**   - BC mortality: 1 in 200 women saved - FP: 50 in 200 (10 of these with tissue samples) - Overdiagnosis: 1 additional diagnosis in 10 diagnosis or in 200 screened (1 in 3 becomes dangerous) (Also, of 10 in 200 women with breast cancer, 1 would not have known about it in their lifetime, and 8 would have been treated successfully without screening) - Interval cancers: of 190 women, 3 are also diagnosed with BC in the 20 yrs between two screening rounds   Format & Definitions: print brochure; FPs: “being called back for suspicious or unclear results that require additional test (e.g., needle biopsy) that subsequently turns out to be benign); harm if causes worry”; overdiagnosis: “tumors found and treated but would never have caused problems”  Data collection: 1 month after receiving information  Other information: incidence of breast cancer (1 of 20 during 50-69; 35 of 1000 women screening for 10 yrs), cure rate (30%), risk with age and family history, FN results (3 of 200 women over 20 yrs between 50-69; vs 10 TP from screening); harm if malignancy only extends period of having breast cancer; better treatment sometimes if earlier | **Attitude (4 5-point items; range -8 to +8; ≥0 positive):**  93.7% had positive attitude about screening; less positive scores in non-immigrant (4.18) vs. immigrant women (4.6 to 5.1 by groups) and most positive in Turkish immigrant women.  **Intentions to start screening:**  83% intended to (start to) participate in program or opportunistic screening *over next 3 months (having already received the invitation)*  **Subgroups:**  Turkish (75%) and other (77%) immigrants had lower intentions  With increasing education level, significantly larger proportions of women intended not to participate (10.5% low, 13.4% medium, and 15.5% high education group). | Sufficient knowledge (risks and benefits; scale 0-7, ≥4 sufficient):  31.5%  32.7% in non-immigrants and 5-25% among immigrant groups |
| Gummersbach 2015, Germany  RCT via survey with old vs new (2010-2015 version) German leaflet sent to women before their first invitation for screening program  Recruitment setting: Primary care clinics  N=353; 48-49; 100%; NR; NR, 17% family history of BC (2% previous diagnosis of BC)  Previous screening: 0%  History of FP or breast surgery: NR, NR  ROB: Low | **Communicated Outcomes of Screening (over 20 yrs):**   - BC mortality: 1 in 200 women saved - FP: 50 in 200 (10 of these with tissue samples) - Overdiagnosis: 1 additional diagnosis in 10 diagnosis or in 200 screened (1 in 3 becomes dangerous) (Also, of 10 in 200 women with breast cancer, 1 would not have known about it in their lifetime, and 8 would have been treated successfully without screening)   Format & Definitions: print brochure; FPs: “being called back for suspicious or unclear results that require additional test (e.g., needle biopsy) that subsequently turns out to be benign; harm if causes worry”; overdiagnosis: “tumors found and treated but would never have caused problems”  Data collection: participants were asked by letter to fill out the questionnaire after reading the leaflet and to send it back in an envelope that was also enclosed in the mailing  Other information: incidence of breast cancer (1 of 20 during 50-69; 35 of 1000 women screening for 10 yrs), cure rate (30%), risk with age and family history, false negative results (3 of 200 women over 20 yrs between 50-69; vs 10 TP from screening) | **Intentions to start screening:**  Intervention: 81.5% (95% 75.8%–87.2%)  Control: 88.6% (95% CI 83.9%–91.3%)  p=0.06  **Subgroups**:  Willingness to participate was negatively correlated with their educational level; if experience of BC in themselves or in close relatives (18.7%), receipt of the new leaflet increased the likelihood that they would be willing to be screened (96.6% versus 72.5%; difference, 24.1%; p = 0.009) | Knowledge about the material benefits and risks of screening (scale 0-10):  5.49 (95% CI: 5.18–5.80) vs 5.23 (95% CI: 4.92–5.55); p = 0.260). |
| Perez-Lacasta 2019, Spain  (associated paper Lo´pez-Panisello 2023)  Cluster RCT of DA vs standard leaflet in women aged 49-50 yrs, approaching age to receive invitations  Recruitment setting: Organized screening program lists (for those becoming eligible)  N=524; 49-50 (50.1 [0.5]); NR; NR; NR; 9% family history  Previous screening: intervention 84% vs control 76%  History of FP or breast surgery: NR, NR  ROB: Moderate | **Communicated outcomes of screening (50-69 yrs old women screened biennially until the age of 80):**   - BC mortality: 1 will survive breast cancer because of screening (4 others will die) in 200 - FP: 40 in 200 - Overdiagnosis: 2 in 200   Format & Definition: leaflet with detailed information on the benefits and harms of screening; contained numerical values and infographics; FP: “mammography results suggest a possible breast cancer that does not really exist. This entails additional explorations that would not be necessary”; overdiagnosis: “Some types of cancer that are detected by screening mammography grow so slowly that they would never become a health problem. Some, even, would disappear spontaneously, without treatment. Currently, one cannot know which tumors would progress and which ones would not, and therefore, treatment is offered to all women diagnosed (“treated without need”); standard leaflet contained recommendations to screen and no information on harms  Data collection: at 2–4 weeks after the estimated leaflet delivery date (either intervention or control); participation at 3 months  Other information: 15 of 200 diagnosed with breast cancer, the treatments of some cancers can be less aggressive with screening; FN: much less frequent (than FPs) and occurs when the mammogram does not show any signs of the disease, even if the woman suffers it | **Attitudes towards screening (5 5-level questions; range 5-25):**  Overall attitude score, mean (SD)  Intervention: 21.49 (3.63)  Control: 21.77 (3.33)  P= 0.357  **Positive attitude to screening (score ≥20):**  Intervention: 75.9%  Control: 78.7%  P= 0.544  **Screening intention (definitely will or likely will):**  Intervention: 82.8%  Control: 82.2%  P= 0.89  **Screening uptake (3 months):**  Intervention: 63.1%  Control: 65.5%  P=0.75  More than 85% of the women in both groups considered that screening participation is important or very important. | **Adequate knowledge (combined conceptual and numerical knowledge about mortality reduction, overdiagnosis, and false positives; ≥11 out of 22):**  Intervention: 33.5%  Control: 1%  PP <0.001  Specific to overdiagnosis: 54% vs. 8.1%  **Overall mean knowledge score**:  Intervention: 13.3  Control; 7.83  P <0.001  **Subgroup analysis by knowledge:**  In the intervention group, women that made an informed choice (i.e., adequate knowledge and attitudes in line with intentions), had lower participation than women without informed choice, 53.2% vs 66.0%.  Partial least squares-structural equation modelling was used by Lo´pez-Panisello 2023 to analyze direct, indirect, and moderating effects between information, knowledge and attitude or intentions.  Attitudes: using an adapted four-item scale which included acceptability and perceived benefits of the screening programme):  Knowledge level significantly worsened women’s attitude towards screening (β = -0.110, p = 0.002) (direct effect)  Intentions:  No direct effect of knowledge on intention: (β = -0.021, p = 0.334) and indirect effect of knowledge on intention was not significant (β = 0.024, p = 0.141) due to opposing impacts of (worse) attitude and (lower) decisional conflict |
| Reder 2017, Germany  RCT of online decision aid and usual care vs usual care (i.e., mammography screening program brochure) age 50 at first invitation  Recruitment setting: Organized program lists  N=913; 50; >90%; NR; NR; 15% first degree relative with breast cancer  Previous screening**:** 33%  History of FP or breast surgery: NR, NR  ROB: Moderate | **Communicated Outcomes of Screening (every 2 yrs for 20 yrs)**  **Decision aid:**   - BC mortality: 4 in 200 over 50 (without mammography) - BC mortality: 3 in 200 over 50 (with mammography) - All-cause mortality: 21 vs 21 in 200 over 50 - FP: 50 in 200 screened - Overdiagnosis: 1 in 200 screened - Interval cancers: 3 in 200 screened   **Program brochure control**:  Same as above but in textual format and no description of all-cause mortality  Format & Definition: interactive online decision aid consisting of static information with crowd figure pictograms for screening vs not screening and interactive parts based on Mathieu et al. DA including discussion of uncertainties; FP: “abnormal mammogram requiring more tests, however the tests show the woman has no BC (“false alarm”)”; overdiagnosis: “would not have noticed their breast cancer during their lifetime”; interval cancers “women diagnosed with BC, although nothing found during screening”, usual care via program brochure had same quantitative data on all outcomes (via text) except for all-cause mortality  Data collection: baseline questionnaire (T1; 60% had received an invitation to screen with program brochure); post-intervention/right after reviewing intervention but no screening appointment date passed (T2); follow-up data collection 3-months after T2 (assuming screening would have been completed if undertaken)(T3)  Other information: true positives/detection rate, negative screening results (140 told they do not have cancer), interactive personal work sheet, evaluating information in favor of or against mammography screening, evaluating importance of information, making decisions about participation in mammography screening, input window for remaining questions, downloadable PDF summarizing information and personal responses | **Positive intention to participate over next 3 months (T1 & T2; yes/no/undecided) and self-report of completed screening (T3):**  Intervention:  T1: 87%; T2: 82%; T3: 65%  Control:  T1: 86%; T2: 82%; T3: 67%  For intention at baseline and uptake at T3 there were no significant differences between the groups. At T2 the proportion of those who did not want to participate in the screening was higher in the DA group (18.1%) than in the control group (10.0%). Women in the control group had higher odds to have a positive intention (OR 2.00, 95% CI 1.21-3.29) than women in the DA group  **Positive attitude (4 5-point questions; ≥0 on scale -8 to 8):**  Intervention:  T1: 90%; T2: 83%; T3: 84%  Control:  T1: 89%; T2: 88%; T3: 85%  **Attitude score** (5-point scale), mean (SD):  Intervention:  T1: 3.39 (2.91); T2: 2.96 (3.41); T3: 2.84 (3.51)  Control:  T1: 3.33 (2.88); T2: 3.20 (2.94); T3: 3.39 (3.48) | **Knowledge score (7 multiple choice questions, focused on conceptual knowledge; >3 considered adequate), mean (SD):**  Intervention:  T1: 2.73 (1.41); T2: 3.96 (1.33); T3: 3.57 (1.16)  Control:  T1: 2.79 (1.34); T2: 2.92 (1.40); T3: 3.21 (1.28)  **Informed intention to screen (i.e., adequate knowledge, positive attitude and intention to screen)**  **I**ntervention: T2: 61.5%, T3 39.8%  Control: T2 28.9%, T3 30.3% |
| Roberto 2020, Italy  RCT of online decision aid vs online standard brochure in women aged >45 at first invitation  Recruitment setting: organized screening program lists (for first invitation; 2 of 6 centres invite at 45 yrs)  N= 1,001; >45 (mean [SD]: 49.7 [3.2]); NR; 96% Italian; NR; NR  Previous screening: 68%  History of FP or breast surgery: NR, NR  ROB: High  **Also included in Table for 40-49 yrs** | **Communicated Outcomes of Screening**  **Decision aid (regular screening over 30 yrs):**   - BC mortality: 12 vs 20 in 1000 women - FP: 45 in 1000 screens (at each screen) (about 30% requiring biopsy); - Interval cancers (“FNs” but described as interval cancers): 1 to 2 in 1000 screened - Overdiagnosis: 4 vs. 0 in 1000   **Control program brochure (regular screening over 20 yrs):**   - BC Mortality:7-9 lives are saved per 1000 screened - FP: 4 of 100 screens - Overdiagnosis (of DCIS): 1 in 10 cancers detected   Format & Definition: online decision aid with text, images and diagrams (icon arrays of screened and not screened)  <https://www.donnainformata-mammografia.it/en/>. FP: “A doubtful result or “positive mammography” means the radiologist has seen images like nodules, masses, calcifications or others that could signal a tumor and thus call for more investigation. This may cause you anxiety and concern but it does not imply a malignant disease. If the suspicion is not confirmed by further tests it is called a “false positive”.”. Overdiagnosis: “Sometimes there is unnecessary and useless treatment (overtreatment), and if it is useless it is harmful, of tumors discovered by the screening that would resolve themselves spontaneously during the woman’s life. These malignancies look just like other tumors but either do not grow or grow very slowly. Unfortunately, so far there is no way to distinguish whether a tumor is actually not harmful, so all cases are treated and the woman will not know what her situation was”; Control group brochure had absolute effects in textual format for FPs: “need to undergo repeat exam but it turns out to be a “false alarm” as the in-depth analyses exclude the presence of a tumor”; overdiagnosis: “some anomalies, among those detected by mammography, are not destined to become invasive tumors and to compromise the woman's health (the so-called overdiagnosis)”, and morality with screening (“for every 1000 women aged between 50 and 69 yrs who regularly perform mammography, 7-9 lives are saved within 20 yrs”)  Data collection: 7-10 days after receiving information  Other information:   - True positives: 5 in 1000 women - True negative: 950 in 1000 women - Other estimates of BC mortality e.g. data about controversies: ~20% in women aged 40-74 yrs over 13 yrs; 25% reduction in women aged 50-69 - Risks related to radiation (comparable to that absorbed in a few weeks from “background radiation”) - Other estimates of overdiagnosis: 4 in 1000 women aged 50 yrs over 30 yrs vs others with 19-30% of cancers | **Screening attendance:**  Intervention: 84%  Control: 83%  (P = 0.6537)  **Positive intention toward screening (5-point Likert scale; NR how determine “positive”)**:  Intervention: 99%  Control: 98%  (P= 0.0230)  **Positive attitude toward screening (6 5-point scale questions but NR how determine “positive”)**:  Intervention: 92%  Control: 92%  (P= 0.0922) | **Adequate knowledge (>8/13 correct answers):**  Intervention:  Baseline: 23%; Follow-up: 44%  Control:  Baseline: 18%; Follow-up: 37 |
| **Ongoing screening in 50 to 69-year-olds** | | | |
| Waller 2013, UK  Qualitative focus groups with purposive sampling for ethnicities, marital and socioeconomic statuses; using NHS 2011 leaflet plus description and data for overdiagnosis  Recruitment setting: Community  N=40 (6 FGs); 50-71 yrs; 100%; 67.5%; NR  Previous screening: 73% regular, 22% not regular; 5% never  History of FP or breast surgery: NR, NR  ROB: Moderate | **Communicated Outcomes of Screening:**   - BC mortality: 1 BC death prevented for every 400 women screened regularly over 10 yrs - FP: no #s - Overdiagnosis: between 1-3 of 8 diagnoses in 1000   Format & Definitions: leaflet and additional information verbally in focus groups; overdiagnosis: “screening can find cancers which are treated but which may not otherwise have been found during your lifetime” and “(they are) so slow-growing that they would not have caused any problems. But because we can’t yet tell which kind of cancer is the slow-growing kind, the woman receives the usual treatment for breast cancer (e.g., surgery). It’s very hard to know what proportion of cancers diagnosed in the screening program are of the slow-growing type and the experts disagree at the moment.” The term “slow-growing” was used rather than overdiagnosis as it is more familiar to the public and differentiate it from FP or treatment of benign conditions.  Data collection: questionnaire distributed before and after receiving overdiagnosis information    Other information in leaflet all descriptive | **Attitude:**  A few women did describe a change in attitude.  **Intentions to screen:**  Remained high overall; few women felt that they would make different decisions about breast screening in the future | Some women found the statistical information on overdiagnosis difficult to understand. The challenge of making sense of the numerical information was implicit  in some participants’ responses but was also mentioned explicitly. |
| Lawrence 2000, USA  Decision aid validation study in sample of European women  Recruitment setting: primary care clinic and community (mixed)  N=71; 50-80; 100%; European Americans for quantitative findings, European and Mexican Americans for qualitative findings; NR  Previous screening**:** 96%  History of FP or breast surgery: NR, NR  ROB: Moderate | **Communicated Outcomes of Screening (in 1000 over 10 yrs):**   - BC mortality:   50-59 yrs: 4 vs 7 deaths  60-69 yrs: 6 vs 10 deaths  18% vs 25% death rate (RRR 30%)   - FP: 5% (specificity [1- false positive rate = 95%]) - Overdiagnosis (“DCIS with treatment but 85% having no recurrence”):   50-59 yrs: 1 vs 5  60-69 yrs: 2 vs 7  Format & Definition: decision aid created with multidisciplinary team and piloted with lay people, including focus groups; FPs: description of rates and consequences provided (e.g., additional films, sonograms, possible biopsy, anxiety, occult cancer); overdiagnosis in terms of recurrence risk for DCIS and all receiving lumpectomy; reliability 100% and validity good (22/28 changed preferences after removing benefits); overdiagnosis is implied rather than explicitly stated, using rates of DCIS without recurrence  Data collection: NR  Other information: average risk information, screening process, treatment options; FNs 15% | **Intentions to screen:**  93% chose to have mammogram; 7% chose not to (similar to baseline)  Overall, 89% (22/28) changed preference as predicted when removing data on benefits (46% explicitly, 32% implicitly by decreased confidence score). |  |
| Toledo-Chavarri 2017, Spain  Qualitative study using 7 semi-structured focus groups to evaluate decision-making and acceptability of a decision aid  Recruitment setting: Screening program and primary care centres  N=39; 40-49 (23%), 50-69 (77%); 100%; NR, 10% family history (‘a few’ with previous BC diagnosis)  Previous screening: 90%  History of FP or breast surgery: 33%, NR  ROB: Moderate | **Communicated Outcomes of Screening (50 – 70 year olds, biennially until they are 80):**   - BC mortality: 1 life saved in 200 - FP: 40 in 200 - Overdiagnosis: 2 in 200   Format & Definitions: print-based decision aid with text and icon arrays for screening. FPs: “additional tests to rule out cancer that may be a false alarm”. Overdiagnosis: “The screening detects harmless cancers. Some types of cancer that are detected by screening grow so slowly that they would never have become a health problem. Some even would have disappeared spontaneously without treatment. Doctors can’t always know if an initial BC can endanger the life of a woman so they offer treatment to all the women diagnosed. This means that some women will be offered treatment they do not need.”  Data collection: DA was reviewed during the 2-hour focus groups  Other information: 15 in 200 diagnosed, with 8 living (regardless of screening), 4 dying (with screening); 1 in 9 women will have BC throughout their lives and 83% of affected women will survive this disease. Mammography does not prevent you from getting cancer. | **Intentions to screen (during one’s 50 and 60s):**  The vast majority of the women who had already considered participating expressed that they would participate. |  |
| Driedger 2017, Canada (Toronto and Winnipeg)  Focus groups (n=5) in 2012 with members of the public using presentations and print hand-outs; survey research company recruitment using known survey participants and random-digit dialing, with maximum variation sampling (e.g. wide variation in income and marital status but education skewed to higher) based on questionnaire. 4 of 5 focus groups stratified by age (35-49 and 45-59)  Recruitment setting: Community  N=46; 35-59 (63% <50); NR, NR, NR, NR  Previous screening: 61% (100% in >50 yrs; most <45 had not)  History of FP or breast surgery: NR, NR  **Also included in Table for 40-49 yrs**  ROB: High | **Communicated Outcomes of Screening (total of 11 yrs):**   - BC mortality:   40-49: 2108 would need to be screened to prevent 1 death  50-59: 721 would need to be screened to prevent 1 death   - FP:   40-49: 690 in 2108  50-59: 204 in 721   - Unnecessary biopsies:   40-49: 75 in 2108  50-59: 26 in 721  Format & Definitions: Presentation and print hand-outs of plain language descriptions of 2011 CTFPHC guidelines (2180- and 721-person diagrams for BC mortality, FPs, and unnecessary biopsies at 40-49 and 50-69), a summary of the relevant research evidence, and a description of the uncertainties that remained. “Some doctors think that finding a cancer through screening in this age group (40-49) – who are usually pre-menopausal, won’t change the available treatment options or the effectiveness of treatments and might expose women to unnecessary risk (including additional testing and anxiety)”  Data collection: focus groups were accompanied by hand-outs given to each participant; followed by being asked at what age they thought screening should begin  Other information: None | **Age to start screening:**  At age 40: 35-49; 21%  50-59; 35%  At age 50: 35-49; 41%  50-59; 29%  Unclear: 35-49; 38%  50-59; 35%  Note: unclear was based on authors’ interpretations because using focus group data to quantify  **Relative importance of outcomes:**  13% (most under 50) felt the potential harms of screening were too high a price to prevent one 40-49 year-old from dying |  |
| Bourmaud 2016, France  RCT standard leaflet vs decision aid  Recruitment setting: organized screen program  N=16,000; 50-74; 100%; random selection of those registered with national health program; NR, NR  Previous screening: 46%  History of FP or breast surgery: NR, NR  ROB: High | **Communicated Outcomes of Screening:**   - Stage distribution/BC mortality: 26 of 100 cancers die over 10 yrs in those screened biennially over 24 yrs vs 40 of 100 cancers in those not screened (with cancer rate 10 in 100) - Treatment morbidity: 30% lower rate of chemotherapy - FP: 94 in 1000 (37 more frequent imaging, some biopsies, 2 surgeries for benign anomaly)   Format & Definitions: 12-page leaflet with illustrations, text, histograms; FPs: “anomalies that are later found to be benign, 37 of 97 will need more frequent imaging, some will need biopsy, 2 will need surgery; abnormal images may lead to additional imaging, anxiety (short-term during examinations), more frequent examinations, having surgery with general anesthetic”  Data collection: women received leaflet in mail and did not know they were being studied; just provided with either intervention or control then attendance at programs measured.  Other information including FN (1-2 in 1000), cancer rate 10 in 100 | **Actual screening attendance:**  Lower attendance in intervention (40.3%) vs control (42.1%) in the 12 months following the invitation (p = 0.02). Previous year attendance in program was 50%.  **Subgroups** (ORs vs. whole sample):  Age: neither 50-59 (aOR 1.0) or 60-74 (aOR 1.02) age groups were associated with differing attendance; P=0.21  Previous attendance for screening (aOR = 15.9 [95% CI 14.2 to 17.4] was independently associated with attendance for screening. |  |
| Haakenson 2006, USA  RCT invitation letter +/- 2 informative brochures in women within large cohort study on mammography and 1 month before scheduled mammogram  Recruitment setting: Primary care clinic (Mayo Clinic)  N=668; 61.5±11; NR but all scheduled for mammograms; 98%; NR, 52% family history of BC and 2% previous BC diagnosis  Previous screening: 99%, 75% annually  History of FP or breast surgery: NR, NR  ROB: High | **Communicated Outcomes of Screening:**   - BC mortality: 21-30% RRR - FP: 10-20% total recalls (including FP and TP); 80% of 8-10% biopsies FP   Format & Definition: 2 brochures: FPs: “call backs quite common (compared with 2-4 per 1000 with diagnosis) often just meaning more imaging needed to look at suspicion area more carefully”; referred to percentage of women who need to return back for additional mammogram or who may need biopsies following a screening mammogram and the abnormality turns out to be not cancerous  Data collection: 1-2 weeks after receiving mailed brochures  Other information: tips about process; recommendations for most people to screen annually; some information on personal risk assessment | **Actual screening attendance:**  Intervention: 1.67% did not attend scheduled mammogram  Control: 4.03% did not attend scheduled mammogram  (P=0.73) | Knowledge about overall reduction in mortality due to screening mammography  (55.2% vs 8.9%; P<.001) and proportions of women who  required follow-up mammograms (35.5% vs 14.9%; P<.001) or  biopsy (59.5% vs 13.3%; P<.001). |
| **Weighing benefits vs harms under moderate net benefit scenarios** | | | |
| **Screening initiation/focus on 50-year-olds** | | | |
| Hersch 2015, Australia  (associated papers Hersch 2017 and 2021)  RCT drawn from random cohort via electoral register comparing two decision aids +/- data on overdiagnosis  Recruitment setting: Community  N at 1-4 weeks follow-up =879 (Hersh 2015); 790, 746, and 712 at 6-month, 1-, and 2-year follow-ups (Hersch 2021); 811 for mediation analysis in Hersch 2017; 48-50; 100%, NR use of electoral register, NR; 4% (one close blood relative diagnosed ≥50 yrs)  Previous screening: NR (not past 2 yrs although 40-49 are eligible without invites in AUS)  History of FP or breast surgery: NR, NR  ROB: Low  **Also included in direct data above (different findings)** | **Communicated Outcomes of Screening (over 20 yrs):**   - BC mortality: 4 avoid dying but 8 still die in 1000 - FP: 412 in 1000 (67 have biopsy) - Overdiagnosis: 19 of 73 (26%) diagnosed in 1000 (IG only)   Format & Definitions: booklet with text and visual formats using icon arrays and schematic with screening vs no screening for overdiagnosis; FPs: “false alarms with extra tests... women often feel anxious while they are having the extra tests and waiting for their results, and then feel relieved when they are told there is no cancer after all. However, some women find that they keep worrying about breast cancer for a while afterwards”. Overdiagnosis: “Screening leads to finding some breast cancers that are not harmful; cancers like this may grow very slowly or just stay the same. Without screening, they would never be noticed or cause any trouble. Further checks and examination, doctors cannot be sure which cancers will be harmless. Therefore, treatment is recommended. So, across all the women who have screening, some end up having treatment they do not need.” Schematic of women screened vs not screened. Details of treatments and their risks. Differences between FPs and overdiagnosis. Materials were posted for women to read at home, consistent with the setup of the Australian breast screening program, which is directly accessible by women without referral. No provider consultation, training or counseling was incorporated; https://ses.library.usyd.edu.au/handle/2123/16658  Data collection 1-4 wks after receiving decision aid via post about 1/3 had discussed with partner or friend but not GP; extended follow-up (Hersch 2021) at 6 months and 1 and 2-yrs  Other information: 54 in 1000 women are diagnosed with BCs that is not over-detection; decision aids had additional information but same for both groups; all participants also received national screening brochure without numerical data; common chemotherapy side effects include nausea and vomiting, tiredness, hair loss, and diarrhoea or constipation; common side effects of radiation include tiredness, and the skin of the breast becoming dry and red or darker in colour | **Positive attitude (6 5-Likert scale questions; score ≥24 of range 6-30):**  Intervention: ≤1 month follow-up 69%; 1 year follow-up 77%; 2 year follow-up 81%  Control: ≤1 month follow-up 83%; 1 year follow-up 85%; 2 year follow-up 82%  Odds ratio: 0.89, 95% CI: 0.59-1.34, P = 0.58 at 2-year follow-up  P of groupXtime interaction = 0.004  **Intention to start screening (definitely or likely) over next 2-3 yrs (at 2 yrs all participants were within screening program eligibility age).**  Intervention: Baseline: 89%; ≤1 month follow-up 74%; 2 year follow-up 82%  Control: Baseline 91%; ≤1 month follow-up 87%; 2 year follow-up 85%  Odds ratio: 0.81, 95% CI: 0.52-1.23, P = 0.35 at 2-year follow-up  P of groupXtime interaction = 0.002 (i.e effect dissipating over time)  **Screening uptake (self-reported):**  IG: 6 month follow-up 15%; 1 year follow-up 31%; 2 year follow-up 55%  CG: 6 month follow-up 20%; 1 year follow-up 30%; 2 year follow-up 56%  Odds ratio: 0.97, 95% CI: 0.73-1.29, P = 0.80 at 2-year follow-up)  P of groupXtime interaction = 0.03  **Screening uptake (using verification from public records for 59%)**:  **I**G: 6 month follow-up 22%; 1 year follow-up 43%; 2 year follow-up 70%  CG: 6 month follow-up 24%; 1 year follow-up 36%; 2 year follow-up 69%  **P** of groupXtime interaction = 0.03 | Overall knowledge, mean (based on all conceptual and numerical knowledge items about BC mortality benefit, overdiagnosis, and FP; maximum 22 points):  IG: ≤1 month follow-up 13.5; 1 year follow-up 9.7; 2 year follow-up 9.6  CG: ≤1 month follow-up 11.8; 1 year follow-up 8.9; 2 year follow-up 8.9  Between groups at all times: P<0.05 (persisting difference was entirely attributable to the intervention group’s superior understanding of the concept of overdetection).  P of groupXtime interaction = 0.004  **After 2 yrs,** conceptual knowledge was adequate in 34.4% of 358 women in the IG compared with 20.1% in CG (odds ratio 2.04, 95% confidence interval 1.46 to 2.85).  **Adequate knowledge of overdiagnosis (authors marking schema)**  Intervention: ≤1 month follow-up 55%; 1 year follow-up 26%; 2 year follow-up 26%  Control: ≤1 month follow-up 27%; 1 year follow-up 17%; 2 year follow-up 18%  P<0.001  **Mediation analysis (Hersch 2017):**  Total effect estimate: −0.2768 (SE: 0.0540; 95% CI: −0.3828 to 0.1708)  Direct effect estimate: −0.0192 (SE: 0.0501; 95% CI: −0.1175 to 0.0791)  Total indirect effect estimate: −0.2576 (SE: 0.0449; 95% CI: −0.3488 to −0.1734)  Reading the intervention DA was associated with a decrease in screening intentions as a result of all specific indirect causal sequences in the model (e.g., intervention DA led to greater knowledge about overdetection led to less positive attitude led to less positive intention).  As the direct effect was not significant, there was no evidence that the intervention affected intentions independently of its influence on the mediators modelled. |
| **Ongoing screening in 50 to 69-year-olds** | | | |
| Baena-Canada 2018, Spain  Qualitative citizens’ jury presented with general information about screening; expert arguments for and against screening; information about screening program; and recommendations for deliberation  Recruitment setting: Organized program lists    N=20; 50-69; NR; 100% Andalusian; NR; 31% family history  Previous screening: 92%  History of FP or breast surgery: NR; NR  ROB: Moderate | **Communicated Outcomes of Screening (timing varied across sources)**   - BC mortality: reduced by 20% (RR 0.80; based on 4 systematic reviews); number needed to screen to prevent one BC death ranges from 235 (British review) to at least 1000 or 2000 (Cochrane review) - Treatment morbidity: Need for aggressive treatments reduced, such as chemotherapy (RR 0.63; 5% vs 9% of cancers) and hormone therapy (RR 0.81), whereas need for radiotherapy increased (RR 1.24) (Cochrane review) - Stage distribution: 4% vs 6% (of cancers) stage III and 4% vs 7% stage IV - FP: 3.36% (British review) to 10% (Cochrane review), with 30% requiring a biopsy - Overdiagnosis: ranges from 1 for every 77 screened (British review) to 1 for every 200 screened/10 of every 2000 screened (Cochrane review)   Format & Definition: PowerPoint presentation with evidence presented from RCTs and systematic reviews; detailed document with explanation of relative versus absolute effects and about all outcomes; FP: “mammogram result determines that cancer is present when actually it is not (“false alarm”), some women require additional follow-up (i.e., additional imaging, surgery) to find out if they actually have BC”; overdiagnosis (“cancer would have remitted spontaneously or would have never manifested over the woman’s life. As a result, all the therapeutic actions applied on the basis of this mammography result would be overtreatment, since they would have been unnecessary and would not have been beneficial for the woman’s health –actually, they would be harmful”); icon arrays for BC mortality and overdiagnosis;  Data collection: jury met on three afternoons for four hours; day 1: introductions, study description and access to key benefits and harms information; day 2: experts presented for and against arguments, and jury could ask questions via neutral moderator; day 3: jury discussed the presentations and program without experts or moderator and reached a conclusion; process supervised by bioethicist  Other information: types of tests administered based on FP; screening causing secondary effects such as pain and exposure to radiation (slight discomfort or pain during the mammographic screening, a bit of anxiety for some women while waiting for the results, a small possibility of developing cancer induced by the radiation from mammographies [3-6 cancers out of every 10,000 screened women aged 47-73 every 3 yrs; but lower now with digital mammography], false sensation of security after learning of a negative result for cancer, which leads her to disregard symptoms or signs in the breasts and not to make consultations upon their occurrence (no numerical information provided)cost of programs, also given link to health authority early detection program information | **Should health authority continue to offer screening to women aged 50-69 yrs**  Yes = 85% (11/13)  No = 15% (2/13)  At baseline 100% had a previous favorable opinion on screening.  Reasons for ‘Yes’ votes:   - Mortality reduction - Prevents ‘greater harm’ - Few negative consequences - Diagnostic test - Absence of alternatives - High efficacy - Public good - Women’s freedom - Capacity and right decision to make   Reasons for ‘No’ votes:   - Lack of efficacy - High cost | From the analysis of the texts, it is clear that the participants improved their knowledge about the screening program. Although there is no direct comparison with the degree of knowledge before the citizen jury, it is obvious that their knowledge was better than before; they were able to position themselves for or against, to comment on the universal offering or the demand for mammography and to express their opinion on its efficacy in terms of reducing mortality, its cost and different aspects of overdiagnosis. There was some indication at least 1 participant thought screening could prevent cancer. |
| **Weighing benefits vs harms under low net benefit scenario** | | | |
| **Screening initiation/focus on 50 to 59-year-olds** | | | |
| Valentine 2022, USA  RCT of a 4-stage intervention: (1) didactic information with benefit/harms; (2) document on possible consequences of overdetection; (3) Aiding Risk Information learning through Simulated Experience (ARISE) consisting of 30 grids with 100 colored squares representing a possible result of cancer screening; (4) narrative of screening experience based on symptom lists and narratives found in literature, versus a hypothetical screening test with no proven physical benefits (study also examined preferences related to prostate cancer screening in men)  Recruitment setting: Public via survey platform  N=2120; 40-70 (49.5 [7.8]); NR; 84% White (10% African American & 3% Hispanic); NR; 0% first degree relative with breast cancer  Previous screening: 72%  History of FP or breast surgery: NR; NR  ROB: Low  **Also included in Table for 40-49 yrs** | **Communicated Outcomes of Screening (≥ 40 yrs over 11 yrs)**   - - BC mortality: 7 in 1000 (with mammogram) (no benefit group)   - BC mortality: 5 in 1000 (with mammogram) (benefit group)   - BC mortality: 7 in 1000 (without mammogram)   - FP: 160 in 1000 - Overdiagnosis: 20 in 1000 (data taken from survey text and images in appendix; which differs from manuscript text for BC mortality [1 fewer per 1000])   Format & Definition: didactic information had text description and icon arrays of a hypothetical test using numeric about BC mortality, FP: ‘false alarms and unnecessarily have additional testing or tissue removed [biopsy]”; and overdetection: “test finds a cancer that is not dangerous, and you are diagnosed and treated for a cancer that would not have caused any trouble; They are diagnosed with cancer and get surgery to remove their cancer or radiation therapy to shrink the tumor. However, this treatment is unnecessary because the cancer would have never grown or spread or caused problems.” ARISE: Section with 30 grids consisting of 100 colored squares (black=death from cancer, yellow=unnecessary biopsy, red=unnecessary treatment, blue=lives saved, and grey=no benefits/harms) each and were informed that each colored square represented the result of one patient’s cancer screening test (3000 possible outcomes viewed); narrative: physical and emotional of a FP biopsy  Data collection: online; randomized to benefit vs no benefit scenarios then each presented with 4 informational interventions; (1) didactic information with explicit recommendation (in benefit scenario: recommending individual decision making for women 40–49 y old and screening every other year for women 50–74); then responded to 3 knowledge questions; (2) descriptive harms information about unnecessary treatment; then recorded preferences; (3) ARISE then recorded preferences (4) narrative experience of physical effects from FP biopsy; then recorded preferences and responded to 3 knowledge questions. Each participant was randomized to either ARISE followed by narrative OR narrative followed by ARISE.  Other information: descriptions of physical, emotional and financial harms from unnecessary treatment; definitions of TPs, FNs, beneficial test scenario included US Preventive Services Task Force recommendations that benefits and harms closely balanced and between ages of 50 and 74 screening should be done every other year | **Preference for screening**  At this point would you want to get the screening test (yes/no)? baseline and after each of the 4 interventions  Baseline: 84.9% yes  <50 vs ≥50 yrs: 88.5% vs. 80.4%; P < 0.001  After 1st and 4^th^ stages (respectively), choice of screening in arm receiving information on beneficial test: 53% and 28-30% (from figure) vs. arm receiving information on unbeneficial test: 30% to 20% (from figure)  As each stage was completed women were less likely to prefer screening test; odds ratios compared with first intervention (95% CI)  2^nd^ stage = OR 0.29 (0.18 to 0.46)  3^rd^ stage = OR 0.12 (0.07 to 0.20)  4^th^ stage -= OR 0.10 (0.06 to 0.17)  Women were more likely to prefer the beneficial screening test compared with the unbeneficial test (OR = 5.69, 95% CI = 2.58 to 12.54, P < 0.001)  **Subgroups: preference for beneficial vs unbeneficial test by age P=0.693** | 3 questions on number of lives saved, FPs, and overdiagnosis per 1000 after didactic intervention and all interventions  Recall accuracy increased from the first to the last intervention for women in the unbeneficial test condition (OR = 1.95, 95% CI = 1.64, 2.32, P < 0.001) but not for women in the beneficial test condition (P = 0.379)  No change in knowledge of the FP rates or rates of overdetection (for both groups) |
| Henriksen 2015, Denmark  Qualitative interviews on official information and leaflet provided  Recruitment setting: Primary care clinics  N=6; 45-49; 100%, NR; NR, NR  Previous screening: 0%  History of FP or breast surgery: NR, NR  ROB: Moderate | **Communicated Outcomes of Screening (over 10 yrs):**   - BC mortality: 4.4 vs 4.8 in 1000; 15% reduction in risk for dying; 2000 screened over 10 yr to save 1 life - All-cause: no difference in length of life if screened - FP: 180 vs 0 in 2000 - Overdiagnosis: 10 in 2000 have diagnosis that will be over-treated; 25% of BC diagnoses; 1 in 4 diagnoses is a sleeping cancer, 33% more women have surgery than actually have cancer   Format & Definitions: print leaflet and interview guide with different formats for numbers with probes and explanations by interviewer; FPs: “unspecified findings”, but all women talked about anxiety and fear this would cause; overdiagnosis: have a cancer diagnosis “sleeping cancer that may or may not waken” that is over-treated; identified as cancer patients and offered surgery despite their cancer being non-progressive  Data collection: interviews lasted 90 minutes and included questions on attitudes towards screening after having read the information leaflet  Other information: 50 out of 1000 women will develop cancer (and positive frame 950 women out of 1000 will never develop cancer). | **Intentions to start screening (in their 50s):**  None of the women expressed a wish to seek out more facts, and after being provided with more information, one woman reconsidered her decision (to start screening when invited) based on information on overdiagnosis. |  |
| Baena-Canada 2015, Spain  RCT standard vs Cochrane 2008 leaflet provided and explained to women right after a mammogram in national program  Recruitment setting: Screening program attendees  N=355; 45-67 (mean 54); 100%; NR; NR, 14% direct family history and 20% indirect family history of BC  Previous screening: 100% just completed  History of FP or breast surgery: 15%, NR  ROB: High | **Communicated Outcomes of Screening (over 10 yrs):**   - BC mortality: 1 death from BC avoided in 2000 - FP: 200 in 2000 experience important psychological distress from FPs - Overdiagnosis: 10 in 2000 (30% of diagnosed) screened are diagnosed and treated unnecessarily   Format & Definitions: Cochrane 2008 leaflet (translated by Spanish speaker with back translation) provided and verbally explained; FPs: “psychological strain until it is known whether or not there is a cancer, can be severe”; overdiagnosis: “healthy women become cancer patients and will be treated unnecessarily with surgery and usually other treatments” (authors mention limited understanding)  Data collection: 1-month after receiving leaflets  Other information: Possible risks from radiation, pain, false insecurity | **Attitude (4 6-point questions; higher worse; <13=positive attitude):**  Intervention: 99.40% positive attitude  Control: 98.9% positive attitude  (p = 1.000)  **Intentions to continue screening:**  Intervention: 175 (98.90%) yes, 2 (1.10%) undecided  Control: 178 women (100%) yes  (P = 0.240) | **Well informed (7 questions, range 0-10; ≥5 “well informed”):**  Intervention: 18.1%  Control: 8.4%  P = 0.008  Mean scores from first to second interview increased from 2.96 (SD 1.23) to 3.95 (SD 1.782.97 (SD 1.16) in the IG and from to 3.43 (SD 1.39) in the CG  P = 0.002  **Subgroup:**  Participants with a higher educational level (secondary and university) also acquired a higher level of knowledge than those with a lower educational level (primary or none). |

aOR, adjusted odds ratio; BC, breast cancer; CG, control group; DA, decision aid; DCE, discrete choice experiment; DCIS, ductal carcinoma in situ; FN, false negative; FP, false positive; IG, intervention group; IQR, interquartile range; NR, not reported; OR, odds ratio; RCT, randomized controlled trial; ROB, risk of bias; RR, relative risk; RRR, relative risk reduction; SD, standard deviation; TN, true negative; TP, true positive; USPSTF, The U.S. Preventive Services Task Force; Vs, versus; Y/N, yes/no; Yrs, years

**Table S3.6. Direct and Indirect Preferences for 70 years and older**

| **Study, Country**  **Study description**  **Recruitment setting**  **Sample size; Age; Insured; White (other race/ethnicities); dense breasts; other risk factors**  **Screening history**  **History of FP or breast surgery** | **Study data e.g. communicated outcomes of screening**  **Format of information and definitions provided**  **Data collection**  **Other information provided** | **Findings** | **Knowledge and understanding (including relevant subgroup findings)** |
| --- | --- | --- | --- |
| **Direct data: specific to relative importance between individual outcomes** | | | |
| No data |  |  |  |
| **Indirect data related to relative importance of benefits versus harms (via attitudes, intentions, and/or attendance)** | | | |
| **Weighing benefits and harms under relatively high net benefit scenarios** | | | |
| **70 yrs and older** | | | |
| Braithwaite 2023, USA  Mixed-method survey conducted before and after receiving a tailored decision-aid for those 75 yrs and older  Recruitment setting: Primary care network  N= 14 at baseline, 11 at post-intervention; 75-84 (79.1 [3.0]); NR; 57.1% White (43% Black or African American); NR; 36% ≥1 first-degree relative with breast cancer  Previous screening: 100% (in past 2 yrs)  History of FP or breast surgery: NR (29% prior breast biopsy); NR  ROB: High | **Communicated outcomes of screening (over 10 yrs):**   - BC mortality: Deaths from BC avoided after 10 yrs: calculated based on participants’ self-reported information (e.g., 0.01%) - All-cause mortality: Deaths from other causes after 10 yrs, calculated based on participants’ self-reported information (e.g., 20%) - Potential harms from mammogram in the first year calculated based on participants’ self-reported information (e.g., 10%)   Format & Definition: tailored infographic booklet created from baseline data inputs into NCI’s Breast Cancer Risk Assessment Tool (BCRAT; <https://bcrisktool.cancer.gov>), to show current and future lifetime breast cancer risk. 100-person diagrams used together with percentages. The Lee-Schonberg Index within the ePrognosis breast cancer screening module ([www.cancerscreening.eprognosis.org](http://www.cancerscreening.eprognosis.org)) was used to calculate the potential screening benefits and harms. Included information on: potential harms from mammogram in the first year (“brief”: anxiety or pain from mammogram; “harms that can stay with us for a long time”: tests we don’t need, treatment that won’t help us live longer, tests and treatment can make us sick e.g. infections after surgery).  Data collection: post-intervention data collected 2 weeks after baseline  Other information: personalized breast cancer risk (5-year and lifetime) were estimated based on self-reported information: e.g. 5-year risk of 2.7%, and life-time risk of 5.7% | **Intention to continue screening:**  1/11 (9.0%) would stop screening; 5/11 (45.5%) were not sure, and the other 5 (45.5%) would continue (all 5 women reporting a family history of BC) | **Understanding (post-intervention survey):**  Learned new information from the booklet: 72.7% |
| Pappadis 2018, USA  Mixed-methods study, qualitative narratives and clustering technique to assess patterns on perceptions of BC overdetection among women ≥70 yrs. Purposive sampling based on race, education and age.  Recruitment setting: Community settings (most) and primary care  N=59; 70-92 (mean [SD]: 77.5 [6.7]); NR; 41% (36% non-Hispanic Black & 24% Hispanic); NR; NR  Previous screening: 92%  History of FP or breast surgery: NR; NR  ROB: High | **Communicated Outcomes of Screening (from 70 yrs)**   - **Mortality benefit/life yrs saved:** screening allowing cancer to be cured and saving 6 yrs of life (dying at 86 from heart disease vs. dying at 80 from BC if not screened) - **Overdiagnosis**: scenario describing proportion of overdetection between 10% and 30% of cancers, with same date of death in screened and unscreened   Format & Definition: research assistant conducted face-to-face interviews with participants using scenarios with figures (as used by Hersch 2013 with change in age from 64 to 74) on overdetection: “abnormality that is correctly diagnosed as cancer might grow very slowly or not at all, meaning that it will never develop into anything dangerous or even noticeable in the woman’s remaining lifetime; when cancers are found we cannot tell which ones are dangerous and which ones will ultimately be harmless, so generally they are all treated straight away ‘unneeded’”; interviews lasted 60 minutes with overdetection portion 10-15 minutes  Data collection: interviews, scenarios and visuals all done in same session  Other information: description of overtreatment, examples of overdetection with different scenarios/percentages | **Data quantified based on qualitative responses during interviews**  **Support regular mammograms**  Across sample: 44%  Understood overdetection: 20%  Did not understand overdetection: 69%  **Women should not undergo mammograms in the absence of symptoms:**  Across sample: 57%  Understood overdetection: 80%  Did not understand overdetection: 35%  **Initial intention to continue screening:**  Continue: 49%  Discontinue: 32%  Depends on physician: 10%  Unsure: 3%  Never had mammogram: 5%  **Desire to continue screening after interview:**  Across sample: 49%  Understood overdetection: 37%  Did not understand overdetection: 62%  P=0.045  Five (8.5%) women stated that information about overdetection influenced their decision to receive a mammogram in the future, with all five stating they were less likely to screen.  Remaining women stated that the information on overdetection would not influence their decisions about mammograms.  **Subgroups:**  There were no systematic differences on theme usage by age (r=0.02, p=0.19), ethnicity (r=0.01, p=0.42), educational level (r=−0.01, p=0.43) or screening preference (r=0.06, p=0.09). | **Understanding of overdetection (via interview transcripts)**  After the scenarios were presented, 29 (49%) women still demonstrated a lack of understanding of the concept of overdetection. Many women expressed suspicion of the concept (skepticism and mistrust), equating it to rationing.  See column 3 for findings based on understanding overdetection |
| **Weighing benefits and harms under relatively moderate-to-low net benefit scenario** | | | |
| **Continuing to screen among patients 70 to 71 yrs old** | | | |
| Mathieu 2007, Australia  RCT standard brochure vs print-based decision aid for women in their 70s using random sample from BC screening program  Recruitment setting: Screening program attendees  N=734; 70-71; 100%; NR; NR  Previous screening: 100% twice in past 5 yrs  History of FP or breast surgery: NR, NR  ROB: Low | **Communicated Outcomes of Screening (over 10 yrs biennially):**   - BC mortality: 6 vs 8 in 1000 - All-cause mortality: 204 vs 206 die (including from breast cancer) - FP: 135 vs 0 in 1000 - Overdiagnosis: 41 vs 26 diagnoses in 1000; defined with example in appendix - Interval cancers: 9 vs 0 in 1000   Format & Definitions: print-based decision aid with text and icon arrays for screening and no screening  <http://www.psych.usyd.edu.au/cemped/com_decision_aids.shtml> ; FPs: “extra tests after an abnormal mammogram. The extra tests will show these women don't have breast cancer. Aside from the inconvenience of attending for these tests, some women will worry long after they have had them”; overdiagnosis (in appendix): “some of these cancers would never be found without screening (see page 19 for more information, “more breast cancers due to screening because some women die of something else first (because so slow growing), and if not screened would not have had treatment (lumpectomy and radiotherapy)”); interval cancers: develop symptoms and are diagnosed with breast cancer between mammograms  Data collection: mailed DA was self-administered; follow-up data were collected by a self-completed, mailed questionnaire  Other information on why choice to be made, risk factors, competing death risks, FNs (9), reassurance 824 vs 974 with symptoms/diagnosis if not screened, worksheets and examples of other women’s sheets; 1 breast cancer may be caused by the radiation from mammograms if 10,000 women aged 70 have 5 screening mammograms over the next 10 yrs | **Attitude:**  95% of all women remained positive toward screening  **Intentions to continue screening:**  Intervention: baseline 77.4% yes, 16% unsure, 6.3% no vs. follow-up mailed questionnaire 85.7% yes, 4.9% unsure, 9.5% no  Control: baseline 77.7% yes, 12% unsure, 10.4% no vs. follow-up mailed questionnaire 80.6% yes, 10.1% unsure, 9.3% no  OR for stopping 1.28 [95% CI, 0.63-2.61]; P=.50)  **Actual re-attendance:**  1-month phone call: no difference in participation between groups (Intervention 5.9% vs Control 7.0%). Most indicated that they were in the process of arranging to be screened (IG: 75.7% vs CG: 74.7%). | **Knowledge (4 concept questions and 5 numeric questions; range 0-10 and % more than 50%):**  Baseline: 4.88 (1.6) vs. after intervention increase by 2.62 (with 77% >50% correct) |
| **75 yrs and older** | | | |
| Schonberg 2020b, USA  (associated paper Cadet 2021b)  RCT of paper-based decision aid for women ≥75 yrs (used during visit) vs pamphlet (i.e., American Geriatrics Society Health in Aging Foundation’s 2-page pamphlet on home safety) scheduled for a routine visit or annual with their primary care provider  Recruitment setting: Primary care attendees  N=546 (282 in decision aid group in Cadet with subgroup data for educational attainment on screening intentions); 75-89 (79.8 [3.7]); NR; 78% (18.4% non-Hispanic Black); NR; 19.8% ≥1 first-degree relative with breast cancer (35% had <10 yr life expectancy)  Previous screening: 100% (eligibility within 24 months but not 6 months)  History of FP or breast surgery: NR; NR  ROB: Low | **Communicated Outcomes of Screening (over 5 yrs)**  **Doctors do not know if mammograms benefit women age 75 or older**  75 to 84 yrs   - BC mortality: 4 in 1000 (without mammogram) - BC mortality: 3 in 1000 (with mammogram)   ≥85 yrs   - BC mortality: 3 in 1000 (without mammogram) - BC mortality: 2 in 1000 (with mammogram)   75 to 84 yrs:   - Stage distribution: 4 breast cancer that has spread outside the breast (without mammography) - Stage distribution: 2 breast cancer that has spread outside the breast (with mammography)   ≥85 yrs   - Stage distribution: 3 breast cancer that has spread outside the breast (without mammography) - Stage distribution: 2 breast cancer that has spread outside the breast (with mammography)   ≥75 yrs   - FP: 100 of 1000 screened   75 to 84 yrs   - Overdiagnosis: 20 in 1000 screened will be diagnosed with small BC and 4 with a pre-cancer vs 12 diagnosed with small cancer without screening   ≥85 yrs   - Overdiagnosis: 17 in 1000 screened will be diagnosed with small BC and 3 with a pre-cancer vs. 9 diagnosed with a small cancer     Format & Definition: paper-based decision aid with text and diagrams (icon arrays for screened and not screened; FP: “these women have an abnormal mammogram but additional tests do not show BC. Most women find this experience causes anxiety (false alarms)”; overdiagnosis “some of these (small or pre) cancers would never have caused problems but these women get treatment”  Data collection: pre-visit questionnaire administered a median of 34 days (IQR 34) before primary provider visit when decision aid or pamphlet received; post-visit questionnaire administered right after visit; attendance follow-up at 18 months  Other information: health calculator to assess whether a mammography would help live longer; causes of death among women; number dx with pre-cancer, small breast cancer and spread for screened and not screened; summary of treatment options; information on breast cancer risk factors, life expectancy by age, competing mortality risks | **Actual screening attendance within 18 mos**  Intervention: 51%  Control: 60%  RR (95% CI): 0.84 (0.75 to 0.95) (P=0.006)  **Intentions to be screened in future (1=intends, 15=does not intend; changes towards and away)**:  Intervention: baseline 3.0 (4.3); lower intentions at follow-up in 24.5%; increased intentions 8%  Control: baseline: 2.9 (4.2); lower intentions in 15.3%; increase NR  **Subgroup findings**:  No apparent effect modification on receipt of screening was found by patient age, educational level (i.e., adjusted %: 48.9% of women with lower education vs 46.0% of women with college degrees (adjusted risk ratio [95% CI]: 0.94 (0.79, 1.13), p = 0.52)) (Cadet 2021b, life expectancy, or breast cancer risk (≥3 vs <3% 5-year).  Women with lower educational attainment were less likely to lower their screening intentions (adjusted %: 11.4% vs. 19.4%, adjusted risk ratio [95% CI]: 1.70, [1.13, 2.56], p = 0.01) after receiving the DA than women with college degrees (n=283 in Cadet 2021b) (adjusted for patient race, marital status, MacArthur Scale of Subjective Social Status, life expectancy, and 5-year Gail model risk). | **Knowledge (least squares mean ± SE)**  11 questions (2 multiple choice and 9 true/false)  Intervention: 7.9 ± 0.1  Control: 6.3 ± 0.1  Adjusted risk difference, mean (95%CI):  1.6 (1.3-1.9)  Women in the DA arm were more knowledgeable about the benefits and harms of mammography than those in the control arm (25.5%] vs 11.7%])(NR how defined) |
| Schonberg 2014, USA  Pre-post trial of decision aid in women attending clinics  Recruitment setting: Primary care attendees  N=45; 75-89; NR (but clinic attendees); 69%; NR  Previous screening: 100% within 3 yrs  History of FP or breast surgery: NR, NR  ROB: Low | **Communicated Outcomes of Screening (over 5 yrs):**   - BC mortality: 3 vs 4 in 1000 women age 75 or older - FP: 100 vs 0 in 1000 women age 75 or older - Overdiagnosis: 4 vs 0 and 20 vs 12 early stage in 1000   Format & Definition: decision aid textual and icon arrays for screening vs no screening; FPs: “abnormal mammogram but additional tests do not show breast cancer. Some women find this experience causes anxiety and lists of testing with mammograms, ultrasound or biopsies (no numerical data)”; overdiagnosis: “some small breast cancers (pre-cancer or early stage) found on an older woman’s mammogram would not have caused problems for at least 5 or 10 yrs. Some of the cancers may never have caused problems.”  Data collection: pretest survey administered then women reviewed DA at routine primary care visit; after the visit the posttest was administered  Other information: BC risk, life expectancy, benefits and harms, competing mortality risks, values clarification exercise, treatments and AEs; few women age 75 or older are treated with chemotherapy; 2 out of 1,000 who do not get a mammogram are diagnosed with late stage breast cancer | **Intention to continue screening:**  82% pretest and 56% posttest intend to get a mammogram (p=0.004)  **Screening attendance** (pre-test [2 yrs priori] vs. post-test [15-month follow-up]) (n=43):  Overall” 85% vs. 63%  ≤9 year life expectancy (n=25): 80% vs. 52%  >9 life expectancy (n=18): 89% vs. 78%  **Subgroups:**  A significant difference in screening intentions was only seen for those with <9 yrs life expectancy (85 vs 50%) vs >9 yrs life expectancy (79 vs 63%). | **f**  Knowledge about mammography (10 questions on risks and benefits of mammography) (range 0-10):  Pre-test: 6.3 (1.3) vs post-test 7.3 (1.4) |
| Cadet 2021a, USA  Pre-post trial of decision aid in women ≥75 yrs at risk for lower health literacy (i.e, women who identified at risk for lower health literacy with a response of “somewhat to not at all confident” to the validated health literacy question on confidence in filling out medical forms by yourself & completion of some college or less)  Recruitment setting: Primary care attendees  N= 43 (18 for intentions data); 75-89 (78 [3.74]); NR; 40% White (57% non-Hispanic Black); NR (63% had <10 yrs estimated life expectancy)  Previous screening: 100% received mammography in the past 3 yrs but not past 6 months  History of FP or breast surgery: NR, NR  ROB: Moderate | **Communicated Outcomes of Screening (75 and older over 10 yrs):**  **Doctors do not know if mammograms are good for women age 75 or older.**   - BC mortality: 1 prevented in 1000 women - Stage distribution: 4 in 1000 women “avoid having a large cancer found” - FP: 200 in 1000 women - Overdiagnosis: 13 in 1000 women   Format & Definition: paper-based DA (as per Schonberg 2020) modified for use in older women with lower health literacy based on interviews; FP: “women who continue having mammograms experience a false alarm. These women have an abnormal mammogram but additional tests do not show breast cancer. Most women find that this experience causes anxiety”; overdiagnosis: “told that they have breast cancer but their breast cancers are growing so slowly that they would never have caused problems. But, once a breast cancer is found on a mammogram nearly all women are treated. Treatment for breast cancer can be hard (overdetection)”; the DA was provided right before their visit with their primary care physician: <https://eprognosis.ucsf.edu/decision_aids/Manmography_LOW_LIT_75-84.pdf>  Data collection: knowledge: after the scheduled visit with their primary care provider, intent to continue screening: 6-month chart review of records indicating a discussion with provider  Other information: deaths from other individual causes in women aged 75 or older (e.g., heart disease: 240 in 1000, other cancers: 220 in 1000, chro(3)nic lung disease: 80 in 1000, etc.) | **Continuing mammography (among those with a documented note about discussion with physician, N=18):**   - Will continue: 67% (12/18) - Will not continue: 22% (4/18) - Undecided: 11% (2/18) | **Knowledge about mammography (assessed with 7 true/false questions):**  pre-test mean [SD]: 3.75 [1.05]), post-test mean [SD]: 4.42 [1.19]; p: 0.03), but post-test scores were worse on concept of overdiagnosis |

aOR, adjusted odds ratio; BC, breast cancer; CG, control group; DA, decision aid; DCE, discrete choice experiment; DCIS, ductal carcinoma in situ; FN, false negative; FP, false positive; IG, intervention group; IQR, interquartile range; NR, not reported; OR, odds ratio; RCT, randomized controlled trial; ROB, risk of bias; RR, relative risk; RRR, relative risk reduction; SD, standard deviation; SE, standard error; TN, true negative; TP, true positive; USPSTF, The U.S. Preventive Services Task Force; Vs, versus; Y/N, yes/no; Yrs, years

**Tables S3.7. Summary of Rias of Bias* Across Studies**

|  | **Domain 1** | **Domain 2** | | | **Domain 3** | | | | **Domain 4** |  |
| --- | --- | --- | --- | --- | --- | --- | --- | --- | --- | --- |
| **Author, year** | **1.1 Was an appropriate sample selected from the sampling frame? Is the sample possibly biased for this review question? (e.g. recruitment of those attending for screening, including people <35 yrs)** | **2.1 Is the response rate acceptable? (i.e. ≥50% of eligible)** | **2.2 Are responders and non-responders sufficiently similar?** | **2.3 Were missing data and attrition sufficiently low to minimize the risk of bias? (i.e., ≥80% of those enrolled)** | **3.1 Was the instrument used for eliciting relative importance of health states valid and reliable? Were most/any of the outcomes adequately measured and defined (e.g. positive attitudes)?** | **3.2 Was the instrument administered in the intended way?** | **3.3 Was a valid representation of health state(s) presented? (i.e., absolute values presented/used and [for indirect studies] was overdiagnosis included). Was overdiagnosis defined adequately?** | **3.4 Did the researchers check understanding of the instrument? (e.g., piloted intervention)** | **4.1 Were the results analyzed appropriately to avoid bias and confounding?** | **Overall bias** |
| **New studies** | | | | | | | | | | |
| Baena-Canada 2018 |  |  |  |  |  |  |  |  |  |  |
| Bilger 2020 |  |  |  |  |  |  |  |  |  |  |
| Braithwaite 2023 |  |  |  |  |  |  |  |  |  |  |
| Cadet 2021a |  |  |  |  |  |  |  |  |  |  |
| Ganott 2006 |  |  |  |  |  |  |  |  |  |  |
| Jafri 2008 |  |  |  |  |  |  |  |  |  |  |
| Laza-Vásquez 2022 |  |  |  |  |  |  |  |  |  |  |
| Pappadis 2018 |  |  |  |  |  |  |  |  |  |  |
| Perez-Lacasta 2019 |  |  |  |  |  |  |  |  |  |  |
| Reder 2017 |  |  |  |  |  |  |  |  |  |  |
| Roberto 2020 |  |  |  |  |  |  |  |  |  |  |
| Schonberg 2020a |  |  |  |  |  |  |  |  |  |  |
| Schonberg 2020b |  |  |  |  |  |  |  |  |  |  |
| Sicsic 2018 |  |  |  |  |  |  |  |  |  |  |
| Stiggelbout 2020 |  |  |  |  |  |  |  |  |  |  |
| Valentine 2022 |  |  |  |  |  |  |  |  |  |  |
| **Old studies** | | | | | | | | | | |
| Baena-Canada 2015 |  |  |  |  |  |  |  |  |  |  |
| Berens 2015 |  |  |  |  |  |  |  |  |  |  |
| Bourmaud 2016 |  |  |  |  |  |  |  |  |  |  |
| Davey 2005 |  |  |  |  |  |  | . |  |  |  |
| Driedger 2017 |  |  |  |  |  |  |  |  |  |  |
| Elkin 2017 |  |  |  |  |  |  |  |  |  |  |
| Gummersbach 2015 |  |  |  |  |  |  |  |  |  |  |
| Gyrd-Hansen 2000 |  |  |  |  |  |  |  |  |  |  |
| Haakenson 2006 |  |  |  |  |  |  |  |  |  |  |
| Henriksen 2015 |  |  |  |  |  |  |  |  |  |  |
| Hersch 2013 |  |  |  |  |  |  |  |  |  |  |
| Lawrence 2000 |  |  |  |  |  |  |  |  |  |  |
| Lewis 2003 |  |  |  |  |  |  |  |  |  |  |
| Mathieu 2007 |  |  |  |  |  |  |  |  |  |  |
| Mathieu 2010 |  |  |  |  |  |  |  |  |  |  |
| Nekhlyudov 2008 |  |  |  |  |  |  |  |  |  |  |
| Paul 2008 |  |  |  |  |  |  |  |  |  |  |
| Saver 2017 |  |  |  |  |  |  |  |  |  |  |
| Schonberg 2014 |  |  |  |  |  |  |  |  |  |  |
| Schwartz 2000 |  |  |  |  |  |  |  |  |  |  |
| Seitz 2016 |  |  |  |  |  |  |  |  |  |  |
| Toledo-Chavarri 2017 |  |  |  |  |  |  |  |  |  |  |
| Van den Bruel 2015 |  |  |  |  |  |  |  |  |  |  |
| Waller 2014 |  |  |  |  |  |  |  |  |  |  |
| Waller 2013 |  |  |  |  |  |  |  |  |  |  |
| Wong 2015 |  |  |  |  |  |  |  |  |  |  |
| Yasunaga 2007 |  |  |  |  |  |  |  |  |  |  |

Green: low; Yellow: moderate/some concerns; Red: High; Grey: No information

*Our assessments were based on items stated in GRADE guidance 19 for assessing risk of bias of preference-based studies (Zhang Y et al. J Clin Epidemiol 2019, 111:94-104). See the manuscript text for more information including our modifications.

**Included studies**

**New primary publications**

From search update

1. Baena-Canada JM, Luque-Ribelles V, Quilez-Cutillas A, Rosado-Varela P, Benitez-Rodriguez E, Marquez-Calderon S, Rivera-Bautista JM. How a deliberative approach includes women in the decisions of screening mammography: a citizens' jury feasibility study in Andalusia, Spain. BMJ open. 2018;8(5):e019852.

2. Bilger M, Ozdemir S, Finkelstein EA. Demand for Cancer Screening Services: Results From Randomized Controlled Discrete Choice Experiments. Value in Health. 2020;23(9):1246-55.

3. Braithwaite D, Chicaiza A, Lopez K, Lin KW, Mishori R, Karanth SD, et al. Clinician and patient perspectives on screening mammography among women age 75 and older: A pilot study of a novel decision aid. PEC innovation. 2023;2.

4. Cadet T, Aliberti G, Karamourtopoulos M, Jacobson A, Gilliam EA, Primeau S, et al. Evaluation of a mammography decision aid for women 75 and older at risk for lower health literacy in a pretest-posttest trial. Patient education and counseling. 2021;104(9):2344-50.

5. Laza-Vasquez C, Martinez-Alonso M, Forne-Izquierdo C, Vilaplana-Mayoral J, Cruz-Esteve I, Sanchez-Lopez I, et al. Feasibility and Acceptability of Personalized Breast Cancer Screening (DECIDO Study): A Single-Arm Proof-of-Concept Trial. International journal of environmental research and public health. 2022;19(16).

6. Pappadis MR, Volk RJ, Krishnan S, Weller SC, Jaramillo E, Hoover DS, et al. Perceptions of overdetection of breast cancer among women 70 yrs of age and older in the USA: a mixed-methods analysis. BMJ open. 2018;8(6):e022138.

7. Perez-Lacasta MJ, Martinez-Alonso M, Garcia M, Sala M, Perestelo-Perez L, Vidal C, et al. Effect of information about the benefits and harms of mammography on women's decision making: The InforMa randomised controlled trial. PloS one. 2019;14(3):e0214057.

8. Reder M, Kolip P. Does a decision aid improve informed choice in mammography screening? Results from a randomised controlled trial. PloS one. 2017;12(12):e0189148.

9. Roberto A, Colombo C, Candiani G, Satolli R, Giordano L, Jaramillo L, et al. A dynamic web-based decision aid to improve informed choice in organised breast cancer screening. A pragmatic randomised trial in Italy. British journal of cancer. 2020;123(5):714-21.

10. Schonberg MA, Davis RB, Karamourtopoulos MC, Pinheiro A, Sternberg SB, Jacobson AR, et al. A Pre-Test-Post-Test Trial of a Breast Cancer Risk Report for Women in Their 40s. American journal of preventive medicine. 2020;59(3):343-54.

11. Schonberg MA, Kistler CE, Pinheiro A, Jacobson AR, Aliberti GM, Karamourtopoulos M, et al. Effect of a Mammography Screening Decision Aid for Women 75 Yrs and Older: A Cluster Randomized Clinical Trial. JAMA internal medicine. 2020;180(6):831-42.

12. Sicsic J, Pelletier-Fleury N, Moumjid N. Women's Benefits and Harms Trade-Offs in Breast Cancer Screening: Results from a Discrete-Choice Experiment. Value in health : the journal of the International Society for Pharmacoeconomics and Outcomes Research. 2018;21(1):78-88.

13. Stiggelbout A, Copp T, Jacklyn G, Jansen J, Liefers G, McCaffery K, Hersch J. Women’s Acceptance of Overdetection in Breast Cancer Screening: Can We Assess Harm-Benefit Tradeoffs? Med Decis Making. 2020;40(1):42-51.

14. Valentine KD, Wegier P, Shaffer VA, Scherer LD. The Impact of 4 Risk Communication Interventions on Cancer Screening Preferences and Knowledge. Medical Decision Making. 2022;42(3):387-97.

From 2018 review excludes

1. Ganott MA, Sumkin JH, King JL, Klym AH, Catullo VJ, Cohen CS, Gur D. Screening mammography: do women prefer a higher recall rate given the possibility of earlier detection of cancer? Radiology. 2006;238(3):793-800.

2. Jafri NF, Ayyala RS, Ozonoff A, Jordan-Gray J, Slanetz PJ. Screening mammography: does ethnicity influence patient preferences for higher recall rates given the potential for earlier detection of breast cancer? Radiology. 2008;249(3):785-91.

**Associated papers**

1. Cadet T, Pinheiro A, Karamourtopoulos M, Jacobson AR, Aliberti GM, Kistler CE, et al. Effects by educational attainment of a mammography screening patient decision aid for women aged 75 yrs and older. Cancer. 2021;127(23):4455-63.

2. Hersch J, Barratt A, McGeechan K, Jansen J, Houssami N, Dhillon H, et al. Informing Women About Overdetection in Breast Cancer Screening: Two-Year Outcomes From a Randomized Trial. Journal of the National Cancer Institute. 2021;113(11):1523-30.

3. Hersch J, McGeechan K, Barratt A, Jansen J, Irwig L, Jacklyn G, et al. How information about overdetection changes breast cancer screening decisions: a mediation analysis within a randomised controlled trial. BMJ open. 2017;7(10):e016246.

4. Lopez-Panisello MB, Perez-Lacasta MJ, Rue M, Carles-Lavila M. Factors influencing intention to participate in breast cancer screening. An exploratory structural model. PloS one. 2023;18(2):e0281454.

**Previous review studies carried forward**

1. Baena-Cañada JM, Rosado-Varela P, Expósito-Álvarez I, González-Guerrero M, Nieto-Vera J, Benítez-Rodríguez E. Using an informed consent in mammography screening: a randomized trial. Cancer Med. 2015;4(12):1923-32.

2. Berens EM, Reder M, Razum O, Kolip P, Spallek J. Informed Choice in the German Mammography Screening Program by Education and Migrant Status: Survey among First-Time Invitees. PLoS One. 2015;10(11):e0142316.

3. Bourmaud A, Soler-Michel P, Oriol M, Regnier V, Tinquaut F, Nourissat A, et al. Decision aid on breast cancer screening reduces attendance rate: results of a large-scale, randomized, controlled study by the DECIDEO group. Oncotarget. 2016;7(11):12885-92.

4. Davey C, White V, Gattellari M, Ward JE. Reconciling population benefits and women's individual autonomy in mammographic screening: in-depth interviews to explore women's views about 'informed choice'. Aust N Z J Public Health. 2005;29(1):69-77.

5. Driedger SM, Annable G, Brouwers M, Turner D, Maier R. Can you un-ring the bell? A qualitative study of how affect influences cancer screening decisions. BMC Cancer. 2017;17(1):647.

6. Elkin EB, Pocus VH, Mushlin AI, Cigler T, Atoria CL, Polaneczky MM. Facilitating informed decisions about breast cancer screening: development and evaluation of a web-based decision aid for women in their 40s. BMC Med Inform Decis Mak. 2017;17(1):29.

7. Gummersbach E, in der Schmitten J, Mortsiefer A, Abholz HH, Wegscheider K, Pentzek M. Willingness to participate in mammography screening: a randomized controlled questionnaire study of responses to two patient information leaflets with different factual content. Dtsch Arztebl Int. 2015;112(5):61-8.

8. Gyrd-Hansen D. Cost-benefit analysis of mammography screening in Denmark based on discrete ranking data. Int J Technol Assess Health Care. 2000;16(3):811-21.

9. Haakenson CP, Vickers KS, Cha SS, Vachon CM, Thielen JM, Kircher KJ, Pruthi S. Efficacy of a simple, low-cost educational intervention in improving knowledge about risks and benefits of screening mammography. Mayo Clin Proc. 2006;81(6):783-91.

10. Henriksen MJ, Guassora AD, Brodersen J. Preconceptions influence women's perceptions of information on breast cancer screening: a qualitative study. BMC Res Notes. 2015;8:404.

11. Hersch J, Barratt A, Jansen J, Irwig L, McGeechan K, Jacklyn G, et al. Use of a decision aid including information on overdetection to support informed choice about breast cancer screening: a randomised controlled trial. Lancet. 2015;385(9978):1642-52.

12. Hersch J, Jansen J, Barratt A, Irwig L, Houssami N, Howard K, et al. Women's views on overdiagnosis in breast cancer screening: a qualitative study. Bmj. 2013;346:f158.

13. Waller J, Elaine D, Katriina LW, Jane W. Women’s responses to information about overdiagnosis in the UK breast cancer screening programme: a qualitative study. BMJ Open. 2013;3(4):e002703.

14. Lawrence VA, Streiner D, Hazuda HP, Naylor R, Levine M, Gafni A. A cross-cultural consumer-based decision aid for screening mammography. Prev Med. 2000;30(3):200-8.

15. Lewis CL, Pignone MP, Sheridan SL, Downs SM, Kinsinger LS. A randomized trial of three videos that differ in the framing of information about mammography in women 40 to 49 yrs old. J Gen Intern Med. 2003;18(11):875-83.

16. Mathieu E, Barratt A, Davey HM, McGeechan K, Howard K, Houssami N. Informed choice in mammography screening: a randomized trial of a decision aid for 70-year-old women. Arch Intern Med. 2007;167(19):2039-46.

17. Mathieu E, Barratt AL, McGeechan K, Davey HM, Howard K, Houssami N. Helping women make choices about mammography screening: an online randomized trial of a decision aid for 40-year-old women. Patient Educ Couns. 2010;81(1):63-72.

18. Nekhlyudov L, Li R, Fletcher SW. Informed decision making before initiating screening mammography: does it occur and does it make a difference? Health Expect. 2008;11(4):366-75.

19. Paul C, Nicholls R, Priest P, McGee R. Making policy decisions about population screening for breast cancer: the role of citizens' deliberation. Health Policy. 2008;85(3):314-20.

20. Saver BG, Mazor KM, Luckmann R, Cutrona SL, Hayes M, Gorodetsky T, et al. Persuasive Interventions for Controversial Cancer Screening Recommendations: Testing a Novel Approach to Help Patients Make Evidence-Based Decisions. Ann Fam Med. 2017;15(1):48-55.

21. Schonberg MA, Hamel MB, Davis RB, Griggs MC, Wee CC, Fagerlin A, Marcantonio ER. Development and evaluation of a decision aid on mammography screening for women 75 yrs and older. JAMA Intern Med. 2014;174(3):417-24.

22. Schwartz LM, Woloshin S, Sox HC, Fischhoff B, Welch HG. US women's attitudes to false-positive mammography results and detection of ductal carcinoma in situ: cross-sectional survey. West J Med. 2000;173(5):307-12.

23. Seitz HH, Gibson L, Skubisz C, Forquer H, Mello S, Schapira MM, et al. Effects of a risk-based online mammography intervention on accuracy of perceived risk and mammography intentions. Patient Educ Couns. 2016;99(10):1647-56.

24. Toledo-Chávarri A, Rué M, Codern-Bové N, Carles-Lavila M, Perestelo-Pérez L, Pérez-Lacasta MJ, Feijoo-Cid M. A qualitative study on a decision aid for breast cancer screening: Views from women and health professionals. Eur J Cancer Care (Engl). 2017;26(3).

25. Van den Bruel A, Jones C, Yang Y, Oke J, Hewitson P. People's willingness to accept overdetection in cancer screening: population survey. BMJ. 2015;350:h980.

26. Waller J, Whitaker KL, Winstanley K, Power E, Wardle J. A survey study of women's responses to information about overdiagnosis in breast cancer screening in Britain. Br J Cancer. 2014;111(9):1831-5.

27. Wong IO, Lam WW, Wong CN, Cowling BJ, Leung GM, Fielding R. Towards informed decisions on breast cancer screening: Development and pilot testing of a decision aid for Chinese women. Patient Educ Couns. 2015;98(8):961-9.

28. Yasunaga H, Ide H, Imamura T, Ohe K. Women's anxieties caused by false positives in mammography screening: a contingent valuation survey. Breast Cancer Res Treat. 2007;101(1):59-64.
